# Upregulation of *Mir342* in Diet-Induced Obesity Mouse and the Hypothalamic Appetite Control

**DOI:** 10.3389/fendo.2021.727915

**Published:** 2021-08-30

**Authors:** Dongxiao Zhang, Satoshi Yamaguchi, Xinhao Zhang, Boxuan Yang, Naoko Kurooka, Ryosuke Sugawara, Haya Hamed H. Albuayjan, Atsuko Nakatsuka, Jun Eguchi, Takeshi Y. Hiyama, Atsunori Kamiya, Jun Wada

**Affiliations:** ^1^ Department of Nephrology, Rheumatology, Endocrinology and Metabolism, Okayama University Graduate School of Medicine, Dentistry and Pharmaceutical Sciences, Okayama, Japan; ^2^ Department of Cellular Physiology, Okayama University Graduate School of Medicine, Dentistry and Pharmaceutical Sciences, Okayama, Japan

**Keywords:** abdominal obesity, non-coding RNAs, adipose tissues, appetite regulation, hypothalamus

## Abstract

In obesity and type 2 diabetes, numerous genes are differentially expressed, and microRNAs are involved in transcriptional regulation of target mRNAs, but miRNAs critically involved in the appetite control are not known. Here, we identified upregulation of miR-342-3p and its host gene *Evl* in brain and adipose tissues in C57BL/6 mice fed with high fat-high sucrose (HFHS) chow by RNA sequencing. *Mir342* (-/-) mice fed with HFHS chow were protected from obesity and diabetes. The hypothalamic arcuate nucleus neurons co-express *Mir342* and EVL. The percentage of activated NPY^+^pSTAT3^+^ neurons were reduced, while POMC^+^pSTAT3^+^ neurons increased in *Mir342* (-/-) mice, and they demonstrated the reduction of food intake and amelioration of metabolic phenotypes. *Snap25* was identified as a major target gene of miR-342-3p and the reduced expression of *Snap25* may link to functional impairment hypothalamic neurons and excess of food intake. The inhibition of miR-342-3p may be a potential candidate for miRNA-based therapy.

## Introduction

microRNAs are non-coding RNAs with the length of 21-25 nucleic acids and repress the expression of hundreds of target mRNAs by the binding to complete or 1- or 2-bp mismatched complementary sequences on 3’-untranslated regions (UTR), mRNA cleavage, mRNA deadenylation, and subsequent translational repression (1). Numerous studies demonstrated that miRNAs play critical roles in fine-tuning of gene expression in various physiological and pathological states (2). Furthermore, miRNAs themselves are transcriptionally regulated, however, little is known about the structural features of miRNA promoters (3) and the accurate miRNA promotor identification is underway (2). In the disease states of obesity and diabetes, differential expression of miRNAs associated with regulation of target mRNAs would be critically involved in the pathogenesis and they are candidates for biomarkers and therapeutic targets. In fact, initial attempts were made to survey miRNA expression profile in pancreatic β cells caused by obesity, hyperglycemia, and dyslipidemia. In the islets of healthy and type 2 diabetes (T2D) organ donors, miR-7a, miR-130a/b, miR-152, and miR-184 were differentially expressed (4). The expression of microRNA is regulated by environmental, genetic, and epigenetic factors and their disturbance is critically involved in the pathogenesis of diabetes and its complications (5, 6). The promoter CpG islands of maternally expressed *MEG3* and miRNA cluster was hypomethylated in T2D organ donors, and reduction of miRNAs caused upregulation of their target genes such as *TP53INP1*, which induced the apoptosis of pancreatic β cells (7). T2D loci were recently identified at clusters of miRNAs maternally expressed *MEG3* and paternally expressed *DLK1* (8). *DLK1* is known to inhibit adipocyte differentiation and protect against obesity (9).

The further attempts were made to identify the miRNAs related to insulin resistance in obesity and T2D, and miRNAs profiling studies were extensively performed in adipose tissues, liver and muscle (6). Notably, miRNAs can be packaged in the extracellular vesicle such as exosomes, which transfer miRNAs between cells and mediate the interorgan crosstalk. Thus, the profile of circulating miRNAs was also vigorously performed (10). In our attempts to identify new therapeutic target of miRNAs, we surveyed expression profile in liver, muscle, white adipose tissues, and sera of C57BL/6JJcl mice fed with standard (STD) and high fat-high sucrose (HFHS) chow by RNA sequencing (GSE61959) (11). We identified unique miRNA gene, *Mir342*, and it is highly upregulated in brain and white adipose tissues by the feeding of HFHS chow in C57BL/6JJcl mice. Here, we report the benefits of the deletion of *Mir342* gene in C57BL/6JJcl mice fed with HFHS chow, *i.e*., amelioration of obesity and T2D. *Mir342* (-/-) mice fed with HFHS chow were characterized with reduced chow intake and reduced activation of neuropeptide Y (NPY) neurons in arcuate nuclei. We also investigated the expression of *Evl*, host gene of *Mir342*, and identified *Snap25* as a target gene of miR-342-3p.

## Materials And Methods

### Animal Models

We obtained Sanger MirKO ES cell line *Mir342* (*Mir342* ^tm1Wtsi^) from MMRRC (Mutant Mouse Resource & Research Centers, University of California, Davis, USA). The insertion of the PGK_EM7_PuDtk_bGHpA cassette created a deletion of size 196 bp (109,896,794–109,896,990 of Chromosome 12 in NC_000078.5 Chromosome 12 Reference MGSCv37 C57BL/6J; 108,624,843-108,624,915 in NC_000078.7 Chromosome 12 Reference GRCm39 C57BL/6J). This deletion eliminates the DNA sequence for this microRNA. The cassette is composed of a loxP site followed by an F3 site followed by a PGK-puromycin-delta-tk cassette, a loxP site and finally an FRT site (12). The germline chimeric mice were prepared under the background of C57BL/6N. Targeted *Mir342* was confirmed by 2 sets of primer pairs, 5’ common rev 5′-ATAGCATACATTATACGAAGTTATCACTGG-3′ and 5’ gene specific fwd (LR1) 5′-AGCTCACTTACATTTTATTTATTTTCTCCT-3′ (5,739 bp); 3’ common fwd 5′-TCTAGAAAGTATAGGAACTTCCATGGTC-3′ and 3’ gene specific rev (LR4) 5′-GTAGGCAAGAAGACATAATACAGAAAAG-3′ (3,232 bp). Wild-type *Mir342* was detected by primers, LR1 and LR4 (8,789 bp). The male chimeric mice were mated with C57BL/6JJcl (CLEA Japan, Tokyo, Japan) to generate heterozygous *Mir342* (+/-). By crossing *Mir342* (+/-) C57BL/6JJcl mice, we generated male homozygous *Mir342* (-/-) and wild-type *Mir342* (+/+) littermates. Five-week-old mice were randomly assigned to standard diet (STD) group (MF, Oriental Yeast, Japan) or high fat high sucrose diet (HFHS) group (D12331, Research Diets, New Brunswick, NJ). At 24 weeks of age, we obtained various organs and they were subjected to following experiments.

### Human Serum Samples

Human serum samples were collected from 65 patients with type 2 diabetes (T2D) in Okayama University Hospital and 45 subjects with normal fasting glucose (NFG, fasting glucose < 110 mg/dL). The patients with malignancies, treatment with steroids and immunosuppressants, and total pancreas resection were excluded. It was approved by Okayama University Graduate School of Medicine, Dentistry and Pharmaceutical Sciences and Okayama University Hospital, Ethics Committee (#1708-045).

### 3T3-L1 Cell Cultures

3T3-L1 pre-adipocytes were cultured in Dulbecco’s modified eagle’s medium (DMEM, 124951, Gibco). On day 0, the media were changed to the differentiation media of the DMEM supplemented with 10% FBS, 10 µg/ml insulin (I1882, Sigma), 1 µM DEX (D2915, Sigma) and 0.5 mM IBMX (I5879, Sigma). On day 2, the media were changed to DMEM supplemented with 10 µg/ml insulin and 10% FBS. The media were changed every day. Total RNA was isolated from 3T3-L1 cells during differentiation from day 1 to day 10 using RNeasy Mini kit (74106, Qiagen) and subjected to RT-qPCR.

### Insulin Tolerance Test and Glucose Tolerance Test (ITT and GTT)

The 20-week-old mice (n=4 in each experimental group) were fasted for 16 hours in GTT and for 3 hours in ITT. They were then intraperitoneally injected with glucose solution (1 mg/g body weight) and human insulin (1 unit/kg in HFHS groups and 0.75 unit/kg in STD groups) for GTT and ITT, respectively. Serum Insulin and leptin levels were measured (Skylight Biotech, Tokyo, Japan).

### Food Intake, Locomotor Activity, and Basal Metabolic Rate

At 16 weeks of age, daily food intake was measured and calculated; daily food intake [g/day/body weight (BW)] = [initial food weight (g) – leftover food weight (g)]/measurement period (days)/BW (g). The locomotor activity was recorded for 24 hours by the frequency of interrupting an infrared sensor (ACTIMO-100, Shinfactory, Fukuoka, Japan). O_2_/CO_2_ metabolism measuring system (MK-5000, Muromachi Kikai, Tokyo, Japan) were used to quantify oxygen consumption rate and carbon dioxide production for the estimation of V̇O2 and respiratory quotient (RQ). Four mice in each experimental group were examined.

### Pair-Feeding Study

*Mir342* (+/+) and *Mir342* (-/-) mice (n=3 for free-feeding and n=5 for pair-feeding) were individually housed and fed with HFHS chow, body weight was measured every week. Food intake of free-fed mice were measured every 3 days. The equal amount of chow consumed by free-fed *Mir342* (-/-) mice for 3 days was given to the pair-fed *Mir342* (+/+) and *Mir342* (-/-) mice.

### Reverse Transcription-Quantitative Polymerase Chain Reaction

RNAs were extracted from frozen tissues and cultured 3T3-L1 cells with RNeasy Mini Kit (Qiagen). For brain tissues, the hypothalamus and ventral midbrain region were removed by free-hand dissection. The QIAamp Circulating Nucleic Acid Kit and the exoRNeasy Serum/Plasma Midi Kit (Qiagen) were used for the isolation of total RNAs from serum and exosomes. For gene expression analyses, cDNAs were prepared with High-Capacity RNA-to-cDNA Kit (Thermo Fisher Scientific). TaqMan gene expression primers, *Evl* (Mm00468405_m1), *Snap25* (Mm01276449_m1), *Cidea* (Mm00432554_m1), *Cox7a1* (Mm00438297_g1), *Pparg* (Mm00440940_m1), *Il6* (Mm00446190_m1), *Il1b* (Mm00434228_m1), *Tnf* (Mm00443258_m1), *Ifng* (Mm01168134_m1), *Lpl* (Mm00434764_m1), *Adipoq* (Mm00456425_m1), *Nhlh2* (Mm01959164_u1), *Msi1* (Mm01203522_m1), *Fat2* (Mm01295775_m1), *Rplp0* (Mm00725448_s1), and *Rn18s* (Mm03928990_g1) were employed. For miRNA expression studies, cDNAs were prepared from total RNAs by TaqMan MicroRNA Reverse Transcription Kit (Life Technologies). MicroRNA primers, hsa-miR-342p (002260), snoRNA202 (001232), snoRNA234 (001234), and cel-miR-39 (000200) were used. *Rplp0*, *Rn18s*, snoRNA202, snoRNA234, and cel-miR-39 were served as the invariant controls. The RT-qPCR was performed using TaqMan Universal PCR Master mix II (no UNG) at a StepOne Plus Real-Time PCR system. The quantification was performed by the 2^−ΔΔCT^ analysis method.

### Western Blot Analysis

The brain and hypothalamic tissues from 24-week-old mice (n=3-4 in each experimental group) were homogenized in RIPA lysis buffer (radioimmunoprecipitation buffer) plus protease inhibitors. The samples were boiled in SDS-PAGE loading buffer, separated on 12% Mini-PROTEAN TGX Precast Protein Gels (Bio-Rad), and transferred to a PVDF Blotting Membrane (cytiva). After blocking with 5% nonfat milk for 1 hour at room temperature (RT), the blots were incubated with rabbit Anti-SNAP25 antibody (ab5666, Abcam, 1:1000), rabbit Anti-EVL antibody (ab204835, Abcam, 1:1000) overnight at 4°C. Rabbit anti-β-Actin antibody (4967S, Cell Signaling Technology) was used as a loading control. After washing three times with Tris-buffered saline (TBS), the blots were incubated with ECL Donkey Anti-Rabbit IgG, HRP-Conjugated Antibodies (NA934V, GE healthcare Life science, 1:10000) at RT for 1 hour. The blots were developed with Pierce ECL Western Blotting Substrate (TE261327, Thermo Fisher Scientific). The chemiluminescence was analyzed using ImageQuant LAS-4000 mini (FUJIFILM).

### Morphometric Analysis for Adipocyte Size

Epidydimal and subdermal adipose tissues were fixed by 10% formalin, embedded with paraffin. The 5-μm paraffin sections were prepared and stained with PAS. The images were captured using an Olympus BX51 microscope. The size of the adipocytes was analyzed by the ImageJ software (National Institutes of Health). Epidydimal and subdermal adipose tissue were taken from 4 individual animals from each experimental group.

### *In Situ* Hybridization

*In situ* hybridization for miRNA was performed using miRCURY LNA miRNA ISH Optimization Kit (FFPE) 4 (Qiagen) on formalin-fixed paraffin embedded (FFPE) tissue samples. The sections were deparaffinized in xylene, hydrated in a series of graded alcohols until water at RT, and followed by washing three times with phosphate-buffered saline (PBS). The slides were incubated for 30 minutes with 3 μg/ml of proteinase K at 37°C. After washing twice with PBS and dehydrated, the sections were hybridized for 2 hours with a gene specific probe (40 nM for double-DIG LNA *Mir342* probe) and LNA *Scramble-miR* probe (double DIG labeled) at 55°C. LNA *U6* snRNA probe (5’ DIG labeled) and LNA *Scramble-miR* probe was used for endogenous control. The sections were washed in stringent condition with 5×SSC, 1×SSC and 0.2× SSC for 10 minutes at 55°C. After the wash with PBS, the sections were blocked with Antibody blocking solution (PBS, 0.1% Tween, 2% Sheep serum, and 1% BSA) and incubated with Anti-Digoxigenin-AP Fab fragments (11093274910, Roche, 1:800) in Antibody Dilutant solution (PBS, 0.05% Tween, 1% Sheep serum, and 1% BSA) for 1 hour at RT. After washing by PBS, the sections were incubated with AP substrate, *i.e.* NBT/BCIP ready-to-use tablet (ROCHE) in 10 ml Milli-Q water and 0.2 mM Levamisole for 2 hours at 30°C. They were then incubated in KTBT buffer (50 mM Tris-Cl, 150 mM NaCl, 10 mM KCl, and 1% Triton X-100) twice for 5 min each to stop the reaction. Finally, the slides were counterstained, dehydrated, and mounted. The processed sections were visualized using an Olympus BX51 microscope.

### Immunofluorescence

The mice (n=4-5 in each experimental group) were fasted for 16 hours and euthanized. The whole brain was taken after systemic perfusion with 4% paraformaldehyde (PFA). For STAT3 activation study, the intraperitoneal injection of mouse recombinant leptin (181030-10-4, FUJIFILM) (1 mg/kg body weight) was given 1 hour before euthanasia. The frozen coronal brain sections (-1.10 mm to -1.90 mm from bregma) were cut at 20-μm thick and fixed in 4% PFA for 15 min. For adipose tissues, 5-μm paraffin sections were first deparaffinized in xylene and hydrated in a series of graded alcohols until water. After antigen retrieval in HistoVT One (nacalai tesque) at 90°C for 15 min, the sections were first incubated in the avidin-biotin blocking kit and incubated with primary antibodies, rabbit Anti-EVL antibody (ab204835, Abcam, 1:250), rabbit Anti-pSTAT3 Antibody (9131, Cell Signaling Technology, 1:500), mouse Anti-NPY antibody (GTX60971, Gene Tex, 1:2000), goat Anti-POMC antibody (ab32893, Abcam, 1:4000), mouse Anti-Neun antibody (ab104224, Abcam,1:500), chicken Anti-GFAP antibody (ab4674, Abcam, 1:500), mouse Anti-IBA1 antibody (sc-32725, Santa Cruz, 1:500), mouse Anti-Myelin Basic Protein antibody (ab62631, Abcam, 1:500), chicken Anti-Tyrosine Hydroxylase antibody (ab76442, Abcam, 1:500) and rat Anti-F4/80 Antibody (ab6640, Abcam, 1:100) for 3 days at 4°C. After 3 times washing by PBS, the tissue sections were then incubated with secondary antibodies, Alexa 647-conjugated goat Anti-Rabbit IgG (ab150083, Abcam, 1:500), Alexa 488-conjugated goat Anti-Rat (ab150165, Abcam, 1:500), Alexa 488-conjugated goat Anti-Mouse (ab150117, Abcam, 1:500), Alexa 488-conjugated donkey Anti-Goat IgG (ab150129, Abcam, 1:500), Alexa 488-conjugated goat Anti-Chicken IgY (ab150173, Abcam, 1:500), Biotin-conjugated donkey Anti-Rabbit/Goat IgG (711-065-152, 705-065-147, Jackson ImmunoResearch, 1:500) for 1 hour at RT. Next, the sections were incubated with fluorescent streptavidin (SA-1200, Vector) for 1 hour at RT. Finally, the sections were mounted using ProLong Gold Antifade Mountant with DAPI (P36931, Thermo Fisher) and observed by an Olympus BX51 microscope. Images covering the whole arcuate nucleus of brain sections (438.6 μm x 330.2 μm = 0.14 mm^2^) were subjected to count cell numbers using Image J software. For quantification, the average cell count from 3 sections per animal was obtained.

### Isolation of Stromal Vascular Fraction From White Adipose Tissues

SVF was isolated from epididymal adipose tissue of 24-week-old mice. Briefly, fresh mouse epididymal fat pads were minced and digested with collagenase type 1 (CLSS1, Worthington) in HBSS containing 10% FBS for 45 minutes at 37°C. The mixture was filtered through a nylon mesh (100 μm), then centrifuged at 400 g for 1 minute. The adipocyte fraction was obtained from the supernatant and the SVF from the pellet.

### Identification of *Mir342* Target mRNAs

The mRNA microarray was performed by GeneChip Mouse Gene 2.0 array using total RNA of epidydimal fat obtained from 16-week-old mice (3 individual animals from each group) and analyzed by Filgen (Nagoya, Japan). The raw data are available in Gene Expression Omnibus (GEO; https://www.ncbi.nlm.nih.gov/geo/) (GSE163880). TargetScan (http://www.targetscan.org/vert_72/), miRDB (http://www.mirdb.org/), Pictar (https://pictar.mdc-berlin.de/) and DIANA-microT v5.0 (https://bio.tools/DIANA-microT) and were used to identify potential target genes for *Mir342*.

### Luciferase Reporter Assay

To quantitatively evaluate miRNA activity on cloned miRNA target sequence from 3’-untranslated region (3’-UTR) of *Snap25*, pmirGLO dual luciferase miRNA Target expression vector (E1330, Promega) was used. Firstly, the pmirGLO plasmid was linearized by double digestion with *Xho*I and *Sac*I. The cDNAs of *Snap25* wild type (WT) 3′-UTR and *Snap25* mutant (MT) 3’-UTR were amplified by PCR and ligated with CIP treated pmirGLO Vector. The primers are Forward *Xho*I *Snap25*: 5’-GGGGGGCTCGAGACAAAGATGCTGGGAAGTGG-3′, Reverse *Sac*I *Snap25*: 5’-GGGGGGGAGCTCCAAACCAACAGAGGAGACAG-3′, Reverse *Sac*I mutant *Snap25*: 5’-GGGGGGGAGCTCCATGCTGTAATGATATTTAGCGCACAGTTTATC-3′. The seed sequence “TCTCACA” was mutated to “GCGCACA”. After transformation to *E. coli* JM109 cells, pmirGLO*-Snap25* WT 3’-UTR, pmirGLO-*Snap25* MT 3’-UTR, and pmirGLO no-insert control plasmids were isolated with EndoFree Plasmid Maxi Kit (12362, Qiagen). HEK293T cells were seeded at a density of 120,000 cells/mL, then co-transfected with *Mir342* mimic (MSY0000590, Qiagen), *Mir342* inhibitor (MIN0000590, Qiagen), negative control *siRNA* (1027280, Qiagen), inhibitor negative control (1027271, Qiagen), pmirGLO*-Snap25* WT 3’-UTR, pmirGLO-*Snap25* MT 3’-UTR, and pmirGLO no-insert control plasmids. Twenty-four hours after transfection, the cells were analyzed to measure luciferase activities using the Dual-Glo Luciferase Assay System and a GloMax 20/20 luminometer (Promega).

### DNA Methylation Analysis

The methylation status of *Evl* and *Mir342* genes was investigated (EpigenDx, http://www.epigendx.com/). NGS (next-generation sequencing) methylation assays were designed to interrogate the DNA methylation status of 103 CpG sites in the 5’ upstream to 3’UTR regions of the Mouse *Evl* gene. The CpG loci location or coordinates are based on Ensembl Gene ID ENSMUSG00000021262 and GRCm39 genomic build. Genomic DNA extracted from brain tissues of *Mir342* (+/+) mice fed with HFHS or STD chow (n=3) were subjected to NGS methylation analysis. Bisulfite modification was carried out using EZ DNA Methylation-Direct Kit (D5020) according to the manufacturer’s protocol (Zymo Research). PCRs included 0.5 units of HotStarTaq (203205, Qiagen), 0.2 μM primers, and 3 μL of bisulfite-treated 200-500 ng DNA in a 20 μL reaction. All PCR products were verified using the Qiagen QIAxcel Advanced System (v1.0.6). Prior to library preparation, PCR products from the same sample were pooled and then purified using the QIAquick PCR Purification Kit columns or plates (28106, Qiagen). Libraries were prepared using a custom Library Preparation method created by EpigenDX. Next, libraries were purified using Agencourt AMPure XP beads (A63882, Beckman Coulter). Barcoded samples were then pooled in an equimolar fashion before template preparation and enrichment were performed in the Ion Chef system using Ion 520 & Ion 530 ExT Chef reagents (A30670, Thermo Fisher). Following this, enriched, template-positive libraries were sequenced on the Ion S5 sequencer using an Ion 530 sequencing chip (A27764). FASTQ files from the Ion Torrent S5 server were aligned to the local reference database using open-source Bismark Bisulfite Read Mapper with the Bowtie2 alignment algorithm. Methylation levels were calculated in Bismark by dividing the number of methylated reads by the total number of reads.

### Statistical Analysis

All values were represented as the mean ± standard deviation (SD). Statistical analyses were conducted using IBM SPSS Statistics 23 and GraphPad Prism (version 8.0). Independent *t*-test, one-way ANOVA with Tukey test and two-way ANOVA with Bonferroni tests were used to determine the differences. For DNA methylation analysis, Fisher’s exact test was applied at each CpG site, and Mann–Whitney *U* test was used by NGS methylation assay. For correlation, non-parametric Spearman r coefficient was used. p<0.05 was considered statistically significant.

## Results

### *Mir342* (-/-) Mice Are Resistant to Diet-Induced Obesity and Diabetes

**Figure 1:**
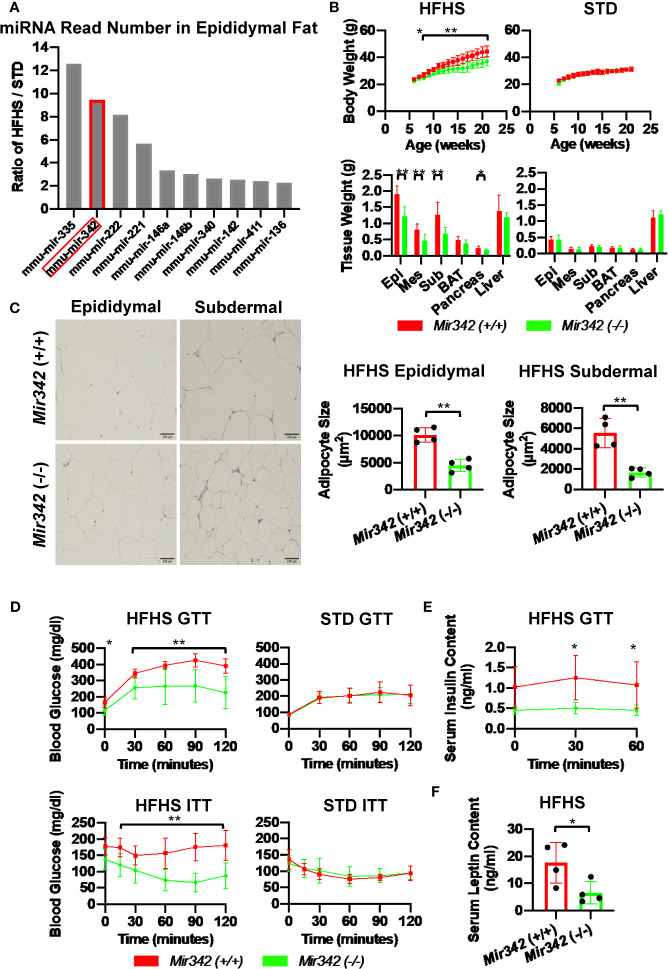
The metabolic phenotypes of *Mir342* (-/-) and *Mir342* (+/+) mice fed with high fat-high sucrose (HFHS) or standard (STD) chow. **(A)** The HFHS/STD ratio of miRNA read numbers in epididymal fat tissues. **(B)** Body and tissue weight of *Mir342* (+/+) and *Mir342* (-/-) mice fed with HFHS (n=7) or STD chow (n=8). **(C)** Adipocyte area in epididymal and subdermal adipose tissues (n=4). Quantitative analyses were carried out on PAS-stained paraffin sections. **(D)** Glucose tolerance test (GTT) and insulin tolerance test (ITT) of mice fed with HFHS (n=7) or STD chow (n=8). **(E)** Serum insulin levels in *Mir342* (+/+) and *Mir342* (-/-) mice fed with HFHS chow during GTT (n=4). **(F)** Fasting serum leptin levels in *Mir342* (+/+) and *Mir342* (-/-) mice fed with HFHS mice (n=4). Data shown as mean ± SD and analyzed by independent *t*-test or two-way ANOVA with Bonferroni tests (*p < 0.05; **p < 0.01).

**Figure 2:**
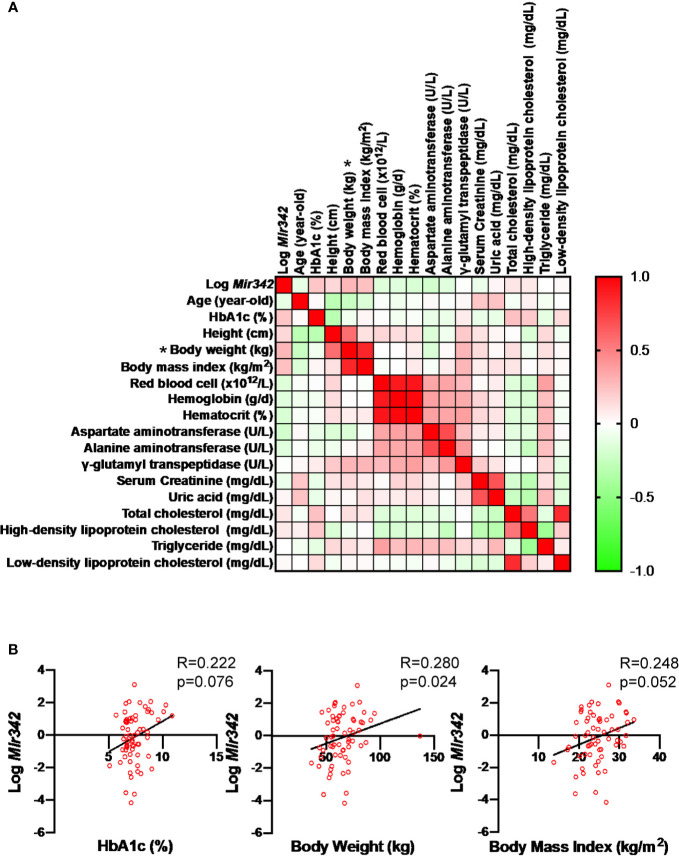
Correlation of serum mmu-mir-342-3p levels (*Log Mir342*) with various clinical parameters in the patients with type 2 diabetes (n=65). **(A)** In correlation matrix, Spearman’s rank correlation coefficient is shown. *P < 0.05. **(B)** The correlations between Log miR-342-3p (Log *Mir342*) with HbA1c (R=0.222, p=0.076), body weight (R=0.280, p=0.024) and body mass index (R=0.248, p=0.052).

To identify miRNAs which are critically involved in the disease process of metabolic syndrome and type 2 diabetes (T2D), we performed miRNA profiling of serum, liver and epididymal fat tissues in C57BL/6JJcl mice fed with standard (STD) and high fat-high sucrose (HFHS) chow (11). The Illumina RNA sequencing data are available in the Gene Expression Omnibus (GEO) under the accession number GSE61959. The miRNA genes with read number more than 2,000 were sorted by HFHS/STD ratios and we identified that *Mir342* was ranked second with 9.5-fold up-regulation in epididymal adipose tissues of HFHS group (**Figure 1A** and **Supplementary Table 1**). In the patients with T2D (n=65), we found that serum concentrations of miR-342-3p showed positive and significant correlation with body weight (**Figure 2**). However, in NFG (n=45) and NFG + T2D (n=110), there were no significant correlations between miR-342-3p and body weight (**Supplementary Figure 1** **)**. There were no significant differences of miR-342-3p levels in T2D with and without metformin (**Supplementary Table 2**). To further give a new insight and investigate the role of *Mir342* in obesity and diabetes, we obtained Sanger MirKO ES cell line *Mir342* (*Mir342* ^tm1Wtsi^) from MMRRC (Mutant Mouse Resource & Research Centers) and generated *Mir342* knockout mice [*Mir342* (-/-)].

**Figure 3:**
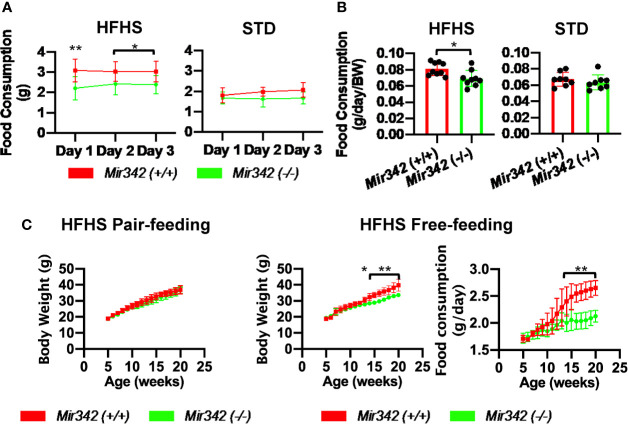
Food intake of *Mir342* (-/-) and *Mir342* (+/+) mice fed with high fat-high sucrose (HFHS) or standard (STD) chow. **(A)** Daily food intake over 3 consecutive days of mice fed with HFHS (n=9) or STD chow (n=8) at 18 weeks of age. **(B)** Average food intake over 3 days per g body weight of mice fed with HFHS (n=9) or STD chow (n=8). **(C)** Pair-feeding studies in *Mir342* (+/+) and *Mir342* (-/-) mice fed with HFHS chow (n=5). Free-feeding mice (n=3 in each group) were used as controls to match the food intake. Data shown as mean ± SD and analyzed by independent *t*-test or two-way ANOVA with Bonferroni tests (*p < 0.05; **p < 0.01).

Body weight of *Mir342* (-/-) mice fed with HFHS chow was significantly reduced compared with *Mir342* (+/+) mice. The weight of epididymal, subdermal and brown fat was also reduced in *Mir342* (-/-) mice. The *Mir342* (-/-) and *Mir342* (+/+) mice fed with STD chow demonstrated no significant differences in their body and tissue weight (**Figure 1B**). The size of adipocytes in epididymal and subdermal adipose tissues derived from *Mir342* (-/-) mice fed with HFHS chow was smaller compared with *Mir342* (+/+) mice (**Figure 1C**). To investigate glucose homeostasis, we performed insulin tolerance test (ITT) and glucose tolerance test (GTT). The blood glucose levels of *Mir342* (-/-) mice fed with HFHS chow were significantly reduced both in ITT and GTT (**Figure 1D**). In GTT, *Mir342* (-/-) mice exhibited significantly lower serum insulin level at 30 and 60 minutes after the peritoneal injection of glucose solution (**Figure 1E**), indicating that insulin sensitivity was significantly improved in *Mir342* (-/-) mice fed with HFHS chow. The levels of fasting serum leptin were also decreased in *Mir342* (-/-) mice, suggesting improved leptin sensitivity (**Figure 1F**). To investigate whether reduced adiposity in *Mir342* (-/-) mice was due to changes in energy intake or energy expenditure, we measured food intake, locomotor activity, and basal metabolic rates. *Mir342* (-/-) mice at 16 weeks of age demonstrated reduced daily food intake under HFHS chow whereas no changes in STD chow (**Figures 3A** **, B**). To prove that increased food intake is the main cause of obesity, we performed pair-feeding experiments in mice fed with HFHS chow. The pair-fed *Mir342* (+/+) mice demonstrated similar body weight with *Mir342* (-/-) mice whereas significantly higher body weight and food consumption were demonstrated in free-fed *Mir342* (+/+) mice after 12 weeks old (**Figure 3C**). The locomotor activity was recorded for over 24 hours, most of the activities were observed during the dark phase in all groups. The significantly increased activity was observed in *Mir342* (-/-) mice under HFHS chow during 23:00-24:00; however, there were no significant differences during whole dark period (**Supplementary Figures 2A, B** **)**. Dark-period oxygen consumption rate, V̇O2, was increased, while daily respiratory quotient (RQ) was reduced in *Mir342* (-/-) mice fed with HFHS chow compared with *Mir342* (+/+) mice (**Supplementary Figures 2C, D** **)**. Since the changes in V̇O2 and RQ were rather mild, the data suggested that the reduction of food intake mainly contributed to the resistance to diet-induced obesity and diabetes in *Mir342* (-/-) mice. There was no difference in the concentrations of miR-342-3p in both isolated exosome and total serum between *Mir342* (+/+) fed with HFHS and STD chow (**Supplementary Figure 3A**). The gene expression of *Cidea*, *Cox7a1*, and *Pparg* was down regulated in brown adipose tissue of *Mir342* (-/-) mice fed compared with *Mir342* (+/+) mice fed with HFHS chow, while they were not altered in epididymal adipose tissue (**Supplementary Figure 3B**).

### *Mir342* and Its Host Gene *Evl* Are Highly Expressed in Neurons Under HFHS Chow

**Figure 4:**
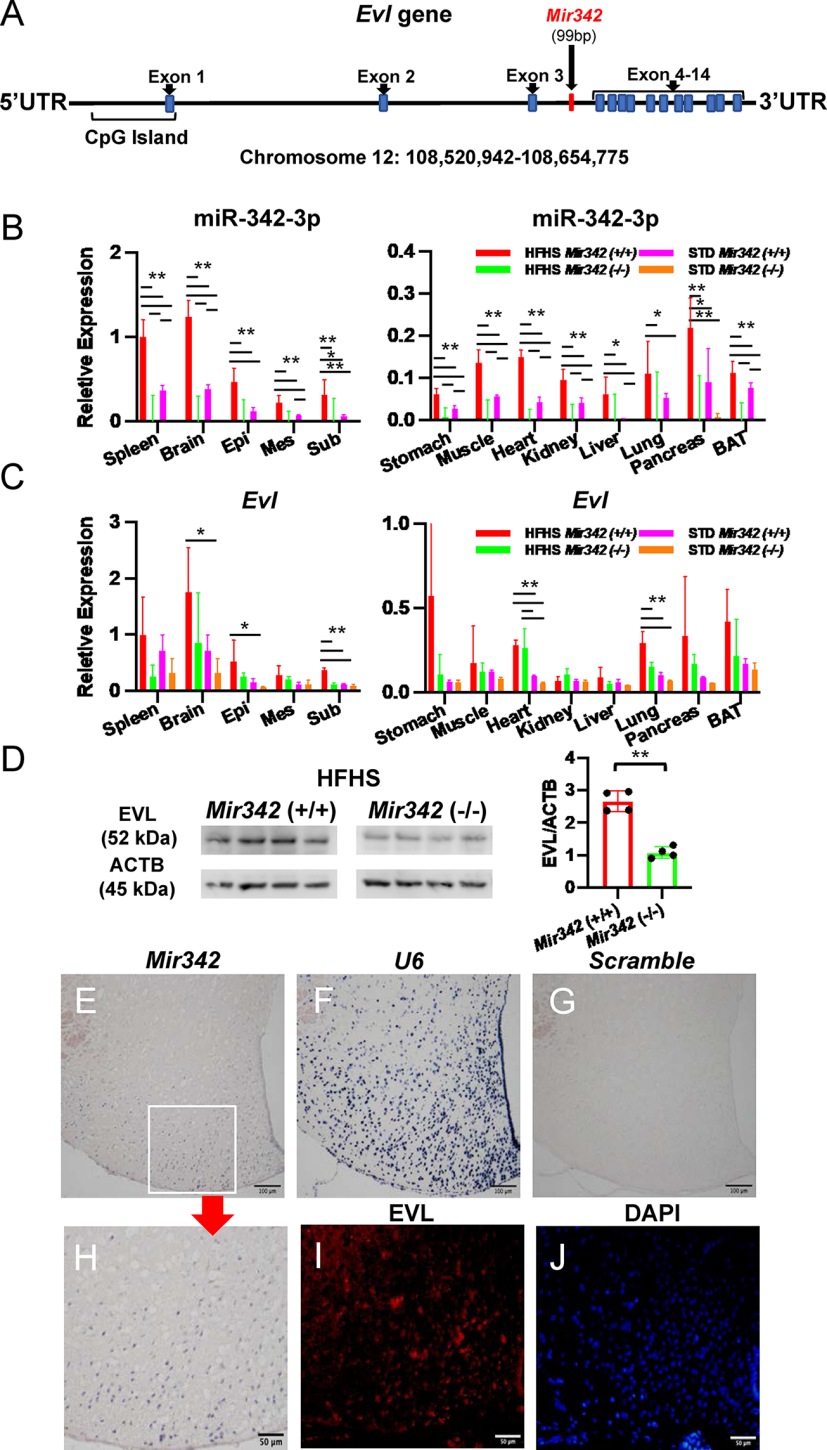
Expression of *Mir342* and its host gene *Evl*. **(A)** *Mir342* is an intronic miRNA in *Evl* (Enabled/Vasodilator-stimulated phosphoprotein) gene. **(B, C)** In various tissues, the expression of miR-342-3p is normalized by snoRNA202 and snoRNA234, while *Evl* is normalized by *Rplp0* and *Rn18s*. HFHS *Mir342* (+/+) (n=4), HFHS *Mir342* (-/-) (n=3), STD *Mir342* (+/+) (n=4) and STD *Mir342* (-/-) mice (n=4). Bar=100 μm. Data are analyzed by one-way ANOVA with a Tukey test. **(D)** Western blot analyses and quantification of EVL protein levels of brain tissue normalized by β-actin (ACTB). Data are analyzed by independent *t*-test. **(E–H)** In *in situ* hybridization, the sections of hypothalamus from *Mir342* (+/+) mice were hybridized with *Mir342* probe (**E**; *Mir342*), *U6* snRNA probe (**F**; *U6*), and *Scramble-miR* probe (**G**; *Scramble*). The inset in **(E)** is shown in panel **(H)**. Immunostaining of EVL (red) in hypothalamus of *Mir342* (+/+) mice **(I)** and nuclear staining of DAPI (blue) **(J)** are shown. Bars are 100 and 50 μm in panels **(E, H)**, respectively. Data presented as means ± SD (*p < 0.05; **p < 0.01).

*Mir342* is located within an intron of the *Evl* (Enabled/vasodilator-stimulated phosphoprotein-like) gene, thus *Evl* is regarded as a host gene of *Mir342* (**Figure 4A**). We further investigated *Mir342* and *Evl* expression in various organs. miR-342-3p was abundantly expressed in spleen, brain and white adipose tissues and they were significantly upregulated in *Mir342* (+/+) fed with HFHS compared with STD chow (**Figure 4B**). We further investigated the distribution of miR-342-3p in hypothalamus and ventral midbrain region, including ventral tegmental area and substantial nigra (**Supplementary Figure 3C**). miR-342-3p was upregulated by HFHS chow in both brain areas, and it was highly expressed in hypothalamus compared with midbrain region. The similar tissue distributions of *Evl* were observed and it was also significantly upregulated in brain and white adipose tissues in *Mir342* (+/+) mice fed with HFHS chow (**Figure 4C**). Both expressions of *Mir342* and *Evl* were regulated in parallel and rather accentuated in brain tissues compared with adipose tissues. In *Mir342* (-/-) mice, the expression of miR-342-3p was absent in all tissues and mRNA expression of *Evl* was reduced in brain and adipose tissues compared with *Mir342* (+/+) mice fed with HFHS chow without statistical significance (**Figures 4B, C** **)**. The reduction of protein levels of EVL were confirmed by Western blot analyses using brain samples in *Mir342* (-/-) mice fed with HFHS chow (**Figure 4D** and **Supplementary Figure 4**).

The expression of *Evl* is mainly regulated by the methylation status of CpG islands (13) and miRNA biogenesis is enhanced by DNA methylation in the regions flanking the miRNA coding sequence (14), we examined the DNA methylation status of 103 CpG sites ranging from the 5’ upstream to 3’UTR regions of the mouse *Evl* gene. The flanking regions of *Mir342* (103,758-103,874 and 115,467-115,543 from TTS of *Evl* gene) were highly methylated. The immediate upstream (103,758-103,874 from TTS) of *Mir342* demonstrated slightly increased methylation by HFHS chow (**Supplementary Figure 5** and **Supplementary Data**) and it may be responsible for the upregulation of *Mir342* by HFHS chow. In contrast, all samples of both STD and HFHS groups were demethylated throughout the CpG island located at 293-585 from TTS of *Evl* gene. The methylation status of CpG island was not responsible for the upregulation of *Evl* gene by HFHS chow.

Next, we investigated the localization of *Mir342* in the cell fractions of epididymal adipose tissues. miR-342-3p was predominantly expressed in stromal vascular fraction (SVF), but lower in mature adipocytes (**Supplementary Figure 6A**). 3T3-L1 pre-adipocytes were induced to differentiate for 10 days, while *Mir342* expression was continuously declined during differentiation (**Supplementary Figure 6B**). Double immunostaining demonstrated that EVL was colocalized with F4/80, indicating that adipose tissue macrophages express EVL (**Supplementary Figures 6C–F**). In cerebral cortex, EVL was colocalized with neuron marker (NeuN, neuronal nuclei), but EVL-positive cells were negative for the markers of dopaminergic neurons (TH, tyrosine hydroxylase), astrocytes (GFAP, glial fibrillary acidic protein), microglia (IBA1, ionized calcium binding adaptor molecule 1), and oligodendrocytes (MBP, myeline basic protein) (**Supplementary Figure 7**). *In situ* hybridization of *Mir342* and immunostaining of EVL demonstrated that they showed similar distribution and their expressions were accentuated in arcuate nuclei in hypothalamus (**Figures 4E** **–J**). The data indicated that EVL and its intronic miRNA, *Mir342*, colocalized in cells and tissues, especially neurons in central nervous system, and their transcriptional activities were coregulated in a parallel manner.

### NPY^+^EVL^+^ and NPY^+^pSTAT3^+^ Neurons Are Reduced in *Mir342 (-/-)* Mice

**Figure 5:**
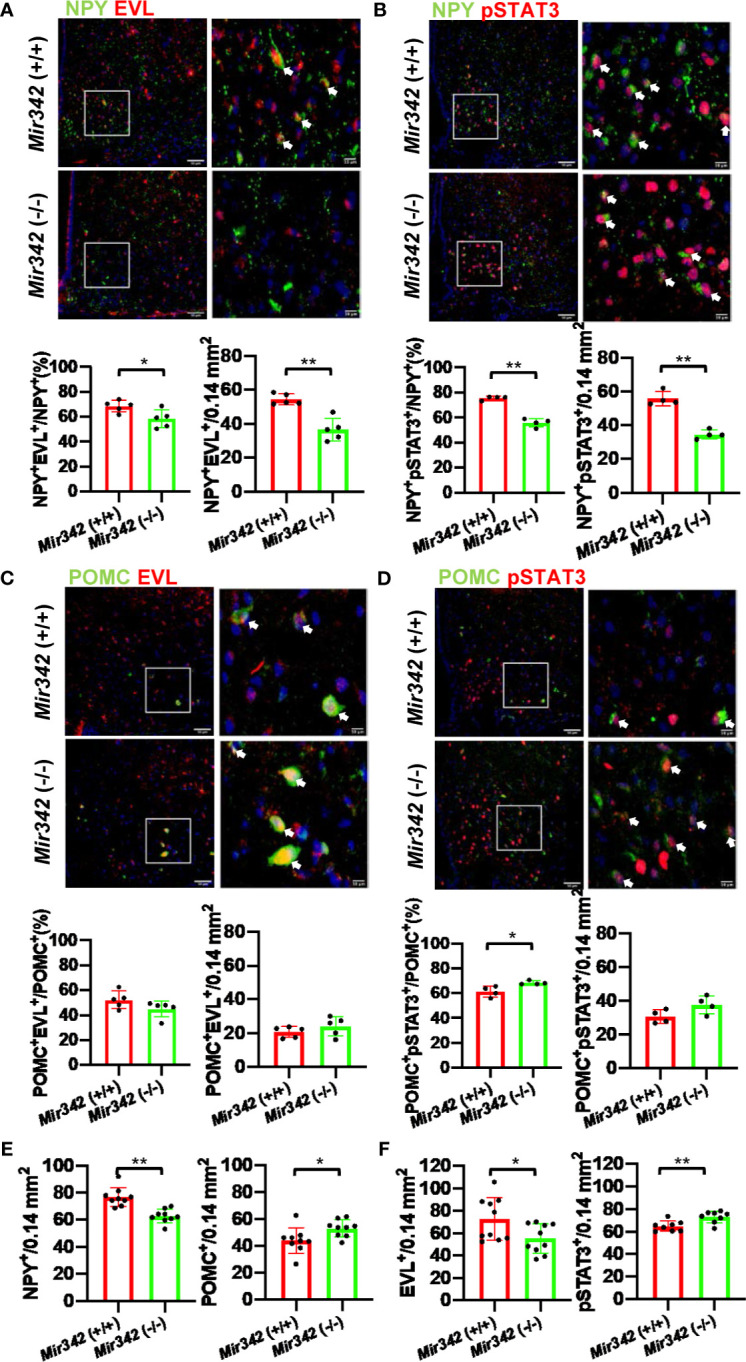
The activation of neuropeptide Y (NPY) and proopiomelanocortin (POMC) neurons by leptin injection. **(A)** Representative photographs of NPY (Green) and EVL (Red) double staining in arcuate nuclei from *Mir342* (+/+) and *Mir342* (-/-) mice fed with HFHS chow (n=5 each). The arrows indicate double-positive cells. The percentage and total numbers of NPY^+^EVL^+^ cells are shown. **(B)** NPY (Green) and pSTAT3 (Red) double staining in the mice fed with HFHS (n=4) after intraperitoneal injection of leptin (1 mg/kg body weight). The percentage and total numbers of NPY^+^pSTAT3^+^ cells are shown. **(C)** Double staining with POMC (Green) and EVL (Red) in *Mir342* (+/+) and *Mir342* (-/-) mice fed with HFHS chow (n=5). The percentage and total numbers of POMC^+^EVL^+^ cells are shown. **(D)** POMC (Green) and pSTAT3 (Red) double staining in the mice fed with HFHS (n=4) after intraperitoneal injection of leptin (1 mg/kg body weight). The percentage and total numbers of POMC^+^pSTAT3^+^ cells are shown. **(E)** Average cell numbers of NPY^+^ (n=9) and POMC^+^ (n=9) cells detected in hypothalamus of the mice fed with HFHS. **(F)** Average cell numbers of EVL^+^ (n=10) and pSTAT3^+^ (n=8) cells detected in hypothalamus of the mice fed with HFHS. Data shown as mean ± SD and analyzed by independent *t*-test (*p < 0.05; **p < 0.01).

An important function of the hypothalamus is to control appetite and satiety. Neuropeptide Y (NPY) and proopiomelanocortin (POMC) neurons are main target of leptin and distribute in hypothalamus. The activation of POMC neurons decreases food intake whereas the activation of NPY neurons increases food intake. The activation of NPY and POMC neurons was investigated after the injection of leptin by double immunostainings of NPY, POMC and phosphorylated signal transducer and activator of transcription 3 (pSTAT3). We identified EVL-positive and EVL-negative NPY and POMC neuron populations in hypothalamus. The percentage of NPY^+^EVL^+^ cells in NPY^+^ neurons were significantly reduced in *Mir342* (-/-) mice compared with *Mir342* (+/+) mice in both STD and HFHS chow (**Figure 5A** and **Supplementary Figures 8A, 9A**). The total number of NPY^+^EVL^+^ neurons were significantly reduced in *Mir342* (-/-) mice fed with HFHS chow (**Figure 5A** **)**. Both percentage and number of activated NPY^+^pSTAT3^+^ neurons were significantly reduced in *Mir342* (-/-) mice fed with HFHS chow (**Figure 5B** and **Supplementary Figure 8B**). No significant difference was obtained in the group of STD chow (**Supplementary Figure 9B**). In contrast, both percentage and total number of POMC^+^EVL^+^ were comparable in the two genotypes, and slight elevation was detected in percentage of POMC^+^pSTAT3^+^ neurons in *Mir342* (-/-) mice (**Figures 5C, D** and **Supplementary Figures 8C, D**). However, there were no significant differences in POMC^+^EVL^+^ and POMC^+^pSTAT3^+^ neurons under STD chow (**Supplementary Figures 9C, D**). In *Mir342* (-/-) mice fed with both STD and HFHS chow, total NPY^+^ neurons were reduced compared with *Mir342* (+/+) mice, while POMC^+^ neurons were increased in HFHS chow (**Figure 5E** and **Supplementary Figure 9E**). Similar to the results of Western blot, EVL-positive cells were reduced in STD and HFHS chow, total pSTAT3^+^ cells were increased in *Mir342* (-/-) mice in HFHS chow (**Figure 5F** and **Supplementary Figure 9F**). The results suggested that deficiency of *Mir342* links to the reduced population and blunted activation of NPY orexigenic neurons, which result in reduced food intake and amelioration of obesity and diabetes under HFHS chow.

### *Snap25* Is a Target of miR-342-3p

**Figure 6:**
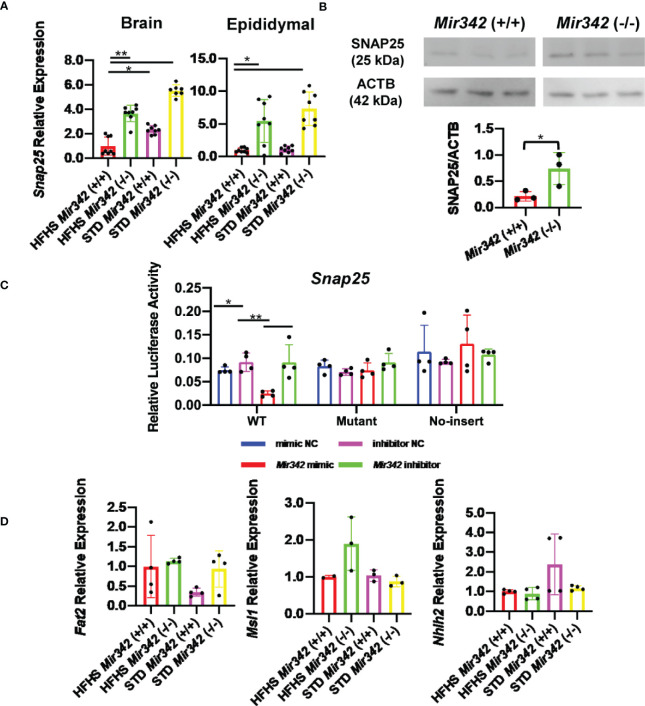
The expression and reporter assay of Snap25 (synaptosomal-associated protein, 25kDa). (A) Relative mRNA expression of Snap25 normalized by Rplp0 and Rn18s in brain and epididymal fat tissues detected by RT-qPCR. (B) Western blot analyses and quantification of SNAP25 protein levels in hypothalamus. (C) Dual-luciferase reporter assay. pmirGLO-Snap25 WT 3’-UTR, pmirGLO-Snap25 MT 3’-UTR, and pmirGLO no-insert control plasmids were cotransfected with Mir342 mimic, Mir342 inhibitor, negative control siRNA (mimic NC), inhibitor negative control (inhibitor NC) into HEK293T cells, respectively. (D) The expression of predicted target genes (Fat2, Msi1 and Nhlh2) in brain. Data are analyzed by independent t-test or one-way ANOVA with a Tukey test. All data are presented as mean ± SD (*p < 0.05; **p < 0.01).

We further performed mRNA profiling by DNA microarray using total RNAs derived from epididymal fat tissues to identify the target genes (GSE163880). We compared 4 groups of *Mir342* (+/+) and *Mir342* (-/-) mice fed with STD and HFHS chow (**Supplementary Tables 3–5**). We selected the predicted target genes of miR-342-3p from TargetScan, miRDB, Pictar and DIANA-microT v5.0 (**Supplementary Table 6**). The results of gene chip demonstrated that *Snap25* (synaptosomal-associated protein, 25kDa) was ranked as top among the genes upregulated in *Mir342* (-/-) mice fed with both STD and HFHS chow (**Supplementary Table 7**). We performed RT-qPCR of top-ranked 3 mRNAs including *Snap25*, *Fat2*, and *Msi1*. In addition, we also check the expression of *Nhlh2*, since it was reported as hypothalamic basic helix-loop-helix transcription factor and the deletion of *Nhlh2* in mice displays adult-onset obesity (15) (**Figure 6**). We confirmed that *Snap25* mRNA increased in both brain and epididymal adipose tissues in *Mir342 (-/-)* mice fed with STD and HFHS chow (**Figure 6A**), while other 3 genes were not altered by the deletion of *Mir342* in brain tissues (**Figure 6D**). Western blot analyses further confirmed that protein expression of SNAP25 was reduced in hypothalamus (**Figure 6B** and **Supplementary Figure 4**). Finally, luciferase reporter assay demonstrated that the transfection of *Mir342* mimic reduced the luciferase activity of pmirGLO*-Snap25* WT 3’-UTR. The luciferase activity of pmirGLO-*Snap25* MT 3’-UTR was not altered by the co-transfection of *Mir342* mimic (**Figure 6C**).

## Discussion

*Mir342* was highly upregulated by HFHS chow in brain in mice, and the striking feature of *Mir342* (-/-) mice fed with HFHS is that they were resistant to development of obesity and T2D. The food intake was reduced in *Mir342* (-/-) mice fed with HFHS and the STAT3 activation of NPY orexigenic neurons was suppressed. By high throughput sequencing, arcuate and paraventricular nuclei were defined by abundant expression of *Mirlet7*, *Mir7*, *Mir9*, and *Mir30* gene families (16), while *Mirlet7a*, *Mir9*, *Mir30b*, *Mir100a*, and *Mir145* were altered by caloric restriction and high-fat diet in hypothalamus (17). The specific knockdown of *Mir7* and knockout of *Mir17-Mir92* in POMC neurons aggravated diet-induced obesity in females and males, respectively (18). In current investigation, we firstly demonstrate the expression of *Mir342* in neurons in arcuate nucleus by *in situ* hybridization and functional relevance in control of appetite and satiety. Intriguingly, STD chow intake was not altered, while HFHS chow intake was reduced by the genetic deletion of *Mir342*. It suggested the roles of *Mir342* and *Evl* in the appetite control for the lipid and sugar rich diet by the alter the development and activation of NPY/POMC progenitors (**Supplementary Figure 10**). We performed RT-qPCR for miR-342-3p in hypothalamus and ventral midbrain region including ventral tegmental area and substantial nigra; however, we did not check the status of dopaminergic neurons in ventral midbrain region in *Mir342* (-/-) mice fed with HFHS. To further confirm whether the inhibition of miR-342-3p is a new therapeutic modality to control appetite and satiety in obesity, the experiments with neuron specific *Mir342* knockout and transgenic mice, and direct injection of miR-342-3p mimic and antagomir are required.

Another major site of *Mir342* expression is adipose tissue. The upregulated expression of *Mir342* in white adipose tissue was reported in diet-induced obese mice (19) and ob/ob mice (20) and also in the patients with HIV-induced lipodystrophy (21). miR-342-3p promotes the adipogenesis in mesenchymal stem cells by suppressing CtBP2 and releasing C/EBPα from CtBP2 binding (22). In the female patients with T2D and impaired fasting glucose (IFG), miRNAs including miR-342-3p were upregulated and *in silico* enrichment analyses suggested the 11 top differentially expressed miRNAs possibly involved in oxidative stress, inflammation and insulin signaling (23). In our study, miR-342-3p was prominently expressed in SVF of epididymal adipose tissue and we were interested in the status of inflammation. However, the gene expression of *Il6*, *Il1b* and *Tnf* were not altered in epididymal adipose tissues.

Epigenetic silencing of *MIR342* and its host gene *EVL* by DNA methylation was reported in colorectal cancer (13, 24, 25), multiple myeloma (26), and B cell lymphoma (27) from the patients. CpG island is located at the promotor region of *EVL* gene, while the expression of *EVL* and intronic miR-342-3p is coregulated in parallel. The DNA methylation of CpG island causes the reduction of miR-342-3p, which is resulted in failure to operate tumor suppressor function by inhibiting pro-survival autophagy by targeting *MAP1LC3B* and *DNMT1* in B cell lymphoma (27). In various tissues of *Mir342* (+/+) mice fed with HFHS chow, the expression of *Evl* and miR-342-3p upregulated in parallel and both genes demonstrated tight co-expression. *EVL* gene is suppressed in colon cancer cells and associated with a dense methylation of CpG island in the 5’-UTR region of *EVL*, which is known as tumor suppressor gene (13). In contrast to previous studies, genomic DNAs in *Mir342* (+/+) mice fed with STD and HFHS were demethylated throughout the CpG island located at 293-585 from TTS of *Evl* gene. However, the flanking regions of *Mir342* were highly methylated. It has been reported that miRNA biogenesis is enhanced by DNA methylation in the regions flanking the miRNA coding sequence (14). It suggested that transcriptional activity of *Evl* and *Mir342* under HFHS chow are differentially regulated by site-specific DNA methylation in the brain. EVL also involves in the actin cytoskeleton formation and multiple actin-dependent process such as axonal morphogenesis and neurites formation (28). One can speculate that the upregulation of *Evl* in neurons and adipose tissue macrophages may alter the neuronal function and motility of macrophages by facilitating the actin cytoskeletal formation, respectively. However, in current investigation, the *Evl* expression was maintained in brain and adipose tissues from *Mir342* (-/-) mice fed with HFHS chow, the roles of *Evl* in obesity and T2D remains unexplored.

In current investigation, we identified *Snap25* as one of the major target genes of miR-342-3p. *Snap25* is an important component of the soluble N-ethylmaleimide-sensitive factor attachment protein (SNARE) complex, contributes formation of 2 out of 4 α helices of the complex, and regulates the membrane fusion during the process of exocytosis (29). The SNARE-mediated fusion such as exocytic fusion and synaptic transmission involved vesicle-associated membrane protein-2 (VAMP2), SNAP25, and syntaxin-1, which are sufficient to fuse membranes *in vitro* experiments (30). *Snap25* is developmentally regulated in neuroendocrine cells and SNAP25a precedes SNAP25b in the development of mouse brain, and SNAP25b becomes major splicing variant at the 2^nd^ postnatal week (31). SNAP25b containing SNARE complexes demonstrate a higher degree of stability associated with increased numbers of pooled and primed vesicles (32). *Snap25b* deficient mice fed with HFHS diet demonstrated exacerbated hyperglycemia, liver steatosis, adipocyte hypertrophy, and reduced expression of pSTAT3 in hypothalamic samples (33), suggesting reduction of SNARE complex stability tightly linked to the obesity and diabetes phenotypes (29). In human studies, *SNAP25* gene single nucleotide polymorphism (SNP, rs362551) associated with severity of metabolic syndrome and type 2 diabetes (34). In addition, SNAP25 interacting protein such as syntaxin-1 SNPs were also associated with obesity (35) and type 2 diabetes (36). Prominent upregulation of miR-342-3p and subsequent reduction of *Snap25* expression in neurons in hypothalamus may link to the instability of SNARE complexes and impairment of neurotransmission.

Taken together, upregulation of *Mir342* and its host gene *Evl* in brain and adipose tissues tightly links to the metabolic syndrome phenotype of HFHS chow induced obesity mice. Percentage activated NPY^+^pSTAT3^+^ neurons were reduced while POMC^+^pSTAT3^+^ neurons increased in *Mir342* (-/-) mice, and they demonstrated the reduction of food intake and amelioration of metabolic phenotypes. We also identified that the major target gene of miR-342-3p is *Snap25* and the functional impairment of SNARE complexes in arcuate nucleus neurons may link to the excess of food intake under HFHS chow. The future studies are necessary to validate the beneficial effects of miR-342-3p antagomir by proof of concept (POC) study using the disease animal models.

### Limitation of Study

In this study, we demonstrated *Mir342* and *Evl* are co-expressed in the central nervous system and adipose tissues, and they were highly upregulated by HFHS chow in C57BL/6JJcl mice. The functional roles of *Mir342* in obesity were demonstrated in the study by investigating *Mir342* (-/-) mice, however, the expression of *Evl* was maintained in *Mir342* (-/-) mice and role of *Evl* in obesity and T2D remains elusive. We examined the expression of miR-342-3p in the sera, exosomes, and various tissues, however, inter-organ communication was not clearly demonstrated, since we did not inject the labeled miR-342-3p into the animal model. The transcriptional regulation of miRNAs is not fully understood, and we did not find out the transcription factors which regulated the expression of *Mir342* and *Evl*. We mainly investigated the major mature form of *Mir342*, *i.e.*, miR-342-3p, however, minor mature form of miR-342-5p may have a role in obesity and T2D.

## Data Availability Statement

The raw data supporting the conclusions of this article will be made available by the authors, without undue reservation. RNA sequencing and mRNA microarray data generated in this study is available at GEO: GSE61959 and GSE163880.

## Ethics Statement

The observational clinical study was approved by Okayama University Graduate School of Medicine, Dentistry and Pharmaceutical Sciences and Okayama University Hospital, Ethics Committee (#1708-045). The patients/participants provided their written informed consent to participate in this study. All animal experiments were approved by the Animal Care and Use Committee of the Department of Animal Resources, Advanced Science Research Center, Okayama University (OKU-2015547, 2016030, 2016203, 2018477, and 2018480).

## Author Contributions

DZ, SY, TH, AK, and JW designed the project and experiments and wrote the manuscript. DZ, SY, XZ, BY, NK, RS, and HA performed animal experiments and analyzed and interpreted data. AN and JE performed culture experiments and molecular biology experiments. TH and AK performed immunohistochemistry of brain tissue. SY, NK, AN, JE, and JW designed clinical study using human serum samples. All authors contributed to the article and approved the submitted version.

## Funding

This work was supported by a Grant-in-Aid for Scientific Research (B) 19H03675, Japan Agency for Medical Research and Development (AMED, grant no: 17ek0210095h0001, 20ek0109445h0001).

## Conflict of Interest

JW receives speaker honoraria from Astra Zeneca, Daiichi Sankyo, MSD, Novartis, Tanabe Mitsubishi, Taisho Toyama and receives grant support from Baxter, Chugai, Dainippon Sumitomo, Ono, Teijin.

The remaining authors declare that the research was conducted in the absence of any commercial or financial relationships that could be construed as a potential conflict of interest.

## Publisher’s Note

All claims expressed in this article are solely those of the authors and do not necessarily represent those of their affiliated organizations, or those of the publisher, the editors and the reviewers. Any product that may be evaluated in this article, or claim that may be made by its manufacturer, is not guaranteed or endorsed by the publisher.
