# Corrigendum: Upregulation of *Mir342* in Diet-Induced Obesity Mouse and the Hypothalamic Appetite Control

**DOI:** 10.3389/fendo.2021.811765

**Published:** 2021-12-10

**Authors:** Dongxiao Zhang, Satoshi Yamaguchi, Xinhao Zhang, Boxuan Yang, Naoko Kurooka, Ryosuke Sugawara, Haya Hamed H. Albuayjan, Atsuko Nakatsuka, Jun Eguchi, Takeshi Y. Hiyama, Atsunori Kamiya, Jun Wada

**Affiliations:** ^1^ Department of Nephrology, Rheumatology, Endocrinology and Metabolism, Okayama University Graduate School of Medicine, Dentistry and Pharmaceutical Sciences, Okayama, Japan; ^2^ Department of Cellular Physiology, Okayama University Graduate School of Medicine, Dentistry and Pharmaceutical Sciences, Okayama, Japan

**Keywords:** abdominal obesity, non-coding RNAs, adipose tissues, appetite regulation, hypothalamus

In the original article, there was a mistake in **Figure 6B** as published. Molecular weight of ACTB was mislabeled as 45 kDa instead of 42 kDa. The corrected **Figure 6B** appears below.

**Figure 6:**
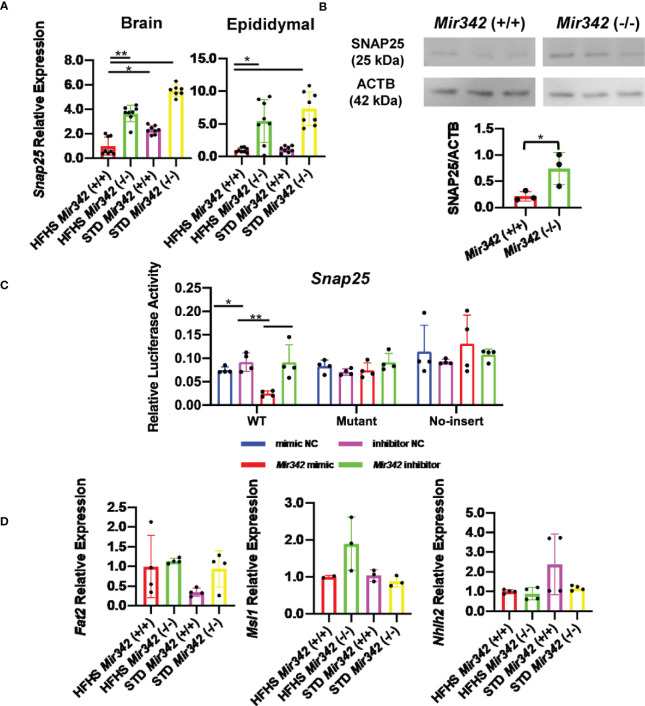
The expression and reporter assay of Snap25 (synaptosomal-associated protein, 25kDa). **(A)** Relative mRNA expression of Snap25 normalized by Rplp0 and Rn18s in brain and epididymal fat tissues detected by RT-qPCR. **(B)** Western blot analyses and quantification of SNAP25 protein levels in hypothalamus. **(C)** Dual-luciferase reporter assay. pmirGLO-Snap25 WT 3’-UTR, pmirGLO-Snap25 MT 3’-UTR, and pmirGLO no-insert control plasmids were cotransfected with Mir342 mimic, Mir342 inhibitor, negative control siRNA (mimic NC), inhibitor negative control (inhibitor NC) into HEK293T cells, respectively. **(D)** The expression of predicted target genes (Fat2, Msi1 and Nhlh2) in brain. Data are analyzed by independent t-test or one-way ANOVA with a Tukey test. All data are presented as mean ± SD (*p < 0.05; **p < 0.01).

The authors apologize for this error and state that this does not change the scientific conclusions of the article in any way. The original article has been updated.

## Publisher’s Note

All claims expressed in this article are solely those of the authors and do not necessarily represent those of their affiliated organizations, or those of the publisher, the editors and the reviewers. Any product that may be evaluated in this article, or claim that may be made by its manufacturer, is not guaranteed or endorsed by the publisher.

