# Supplementary material for: Upregulation of Mir342 in Diet-Induced Obesity Mouse and the Hypothalamic Appetite Control
Source: Front Endocrinol (Lausanne). 2021 Aug 30;12:727915. doi: 10.3389/fendo.2021.727915 (PMC8437242; doi:10.3389/fendo.2021.727915)
Supplement: Supplementary file 2 [file Presentation_1.pdf]

Supplementary Figure 1

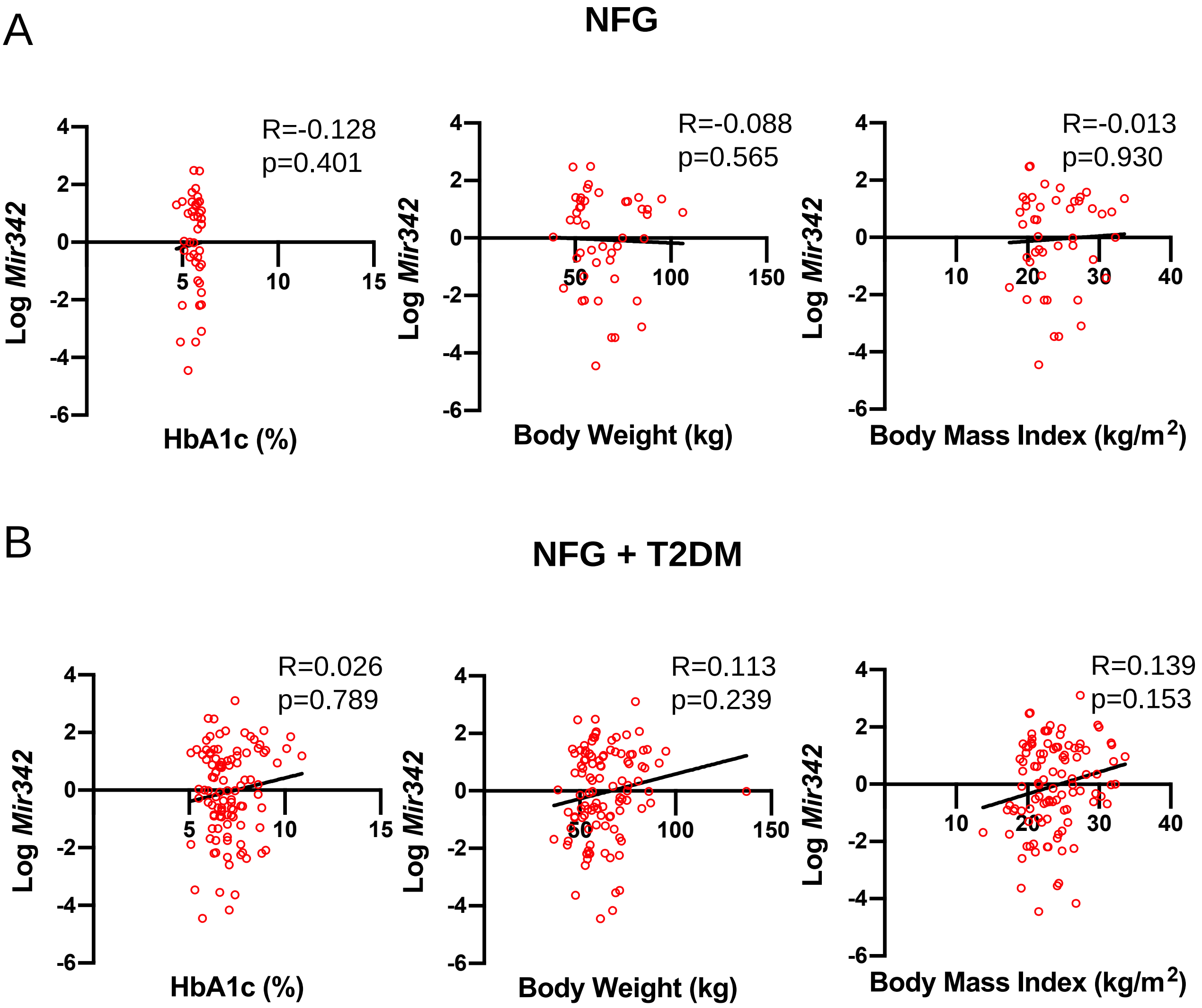

Supplementary Figure 1. (**A**) The correlations between Log *Mir342* with HbA1c (R=-0.128, p=0.401), body weight (R=-0.088, p=0.565) and body mass index (R=0.013, p=0.930) in normal fasting glucose (NFG) (n=45). (**B**) The correlations between Log miR-342-3p (Log *Mir342*) with HbA1c (R=0.026, p=0.789), body weight (R=0.113, p=0.239) and body mass index (R=0.139, p=0.153) in NFG and the patients with type 2 diabetes (T2D) (n=110).

Supplementary Figure 2

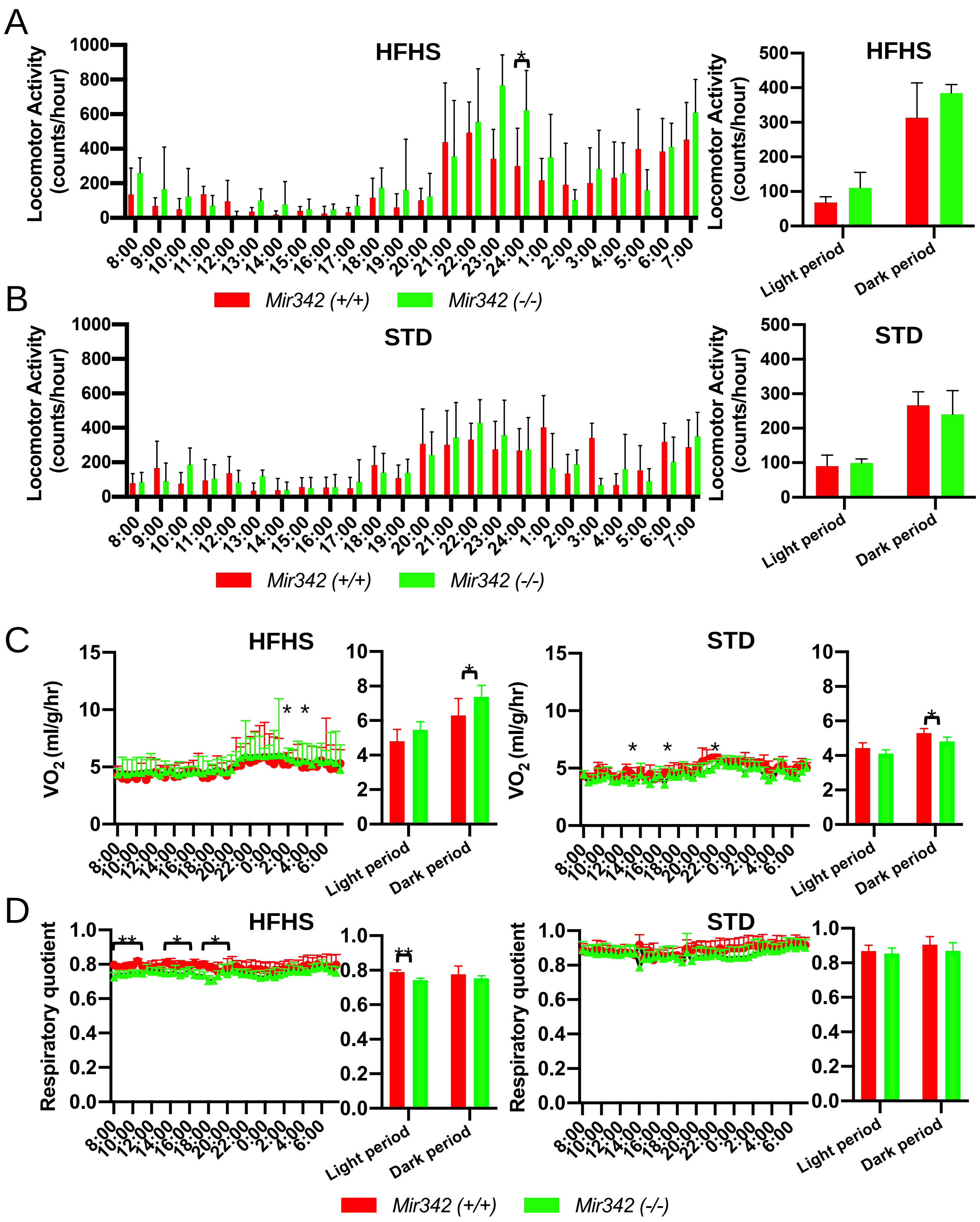

Supplementary Figure 2. Locomotor activity and basal metabolic rate. (**A**) Locomotor activity in *Mir342* (+/+) and *Mir342* (-/-) mice fed with high fat-high sucrose (HFHS) chow (n=4). (**B**) Locomotor activity in *Mir342* (+/+) and *Mir342* (-/-) mice fed with standard (STD) chow (n=4). (**C**) Oxygen consumption rate (VO2) in *Mir342* (+/+) and *Mir342* (-/-) mice fed with HFHS and STD chow (n=4). (**D**) Respiratory quotient (RQ) *Mir342* (+/+) and *Mir342* (-/-) mice fed with HFHS and STD chow (n=4). Data shown as mean ± SD and analyzed by independent *t*-test (*p<0.05, **p<0.01).

Supplementary Figure 3

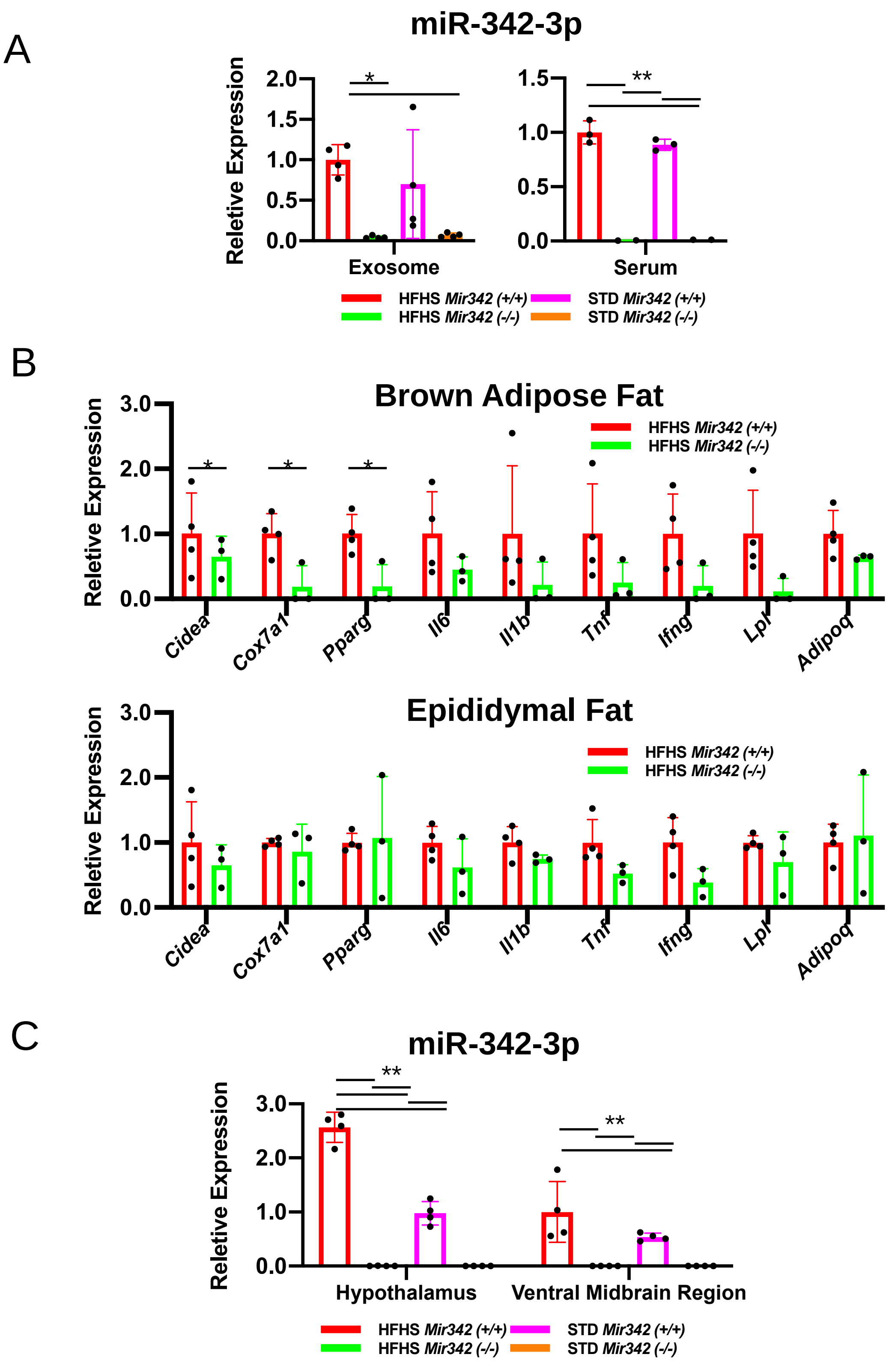

Supplementary Figure 3. Quantitative RT-PCR in serum and various tissues. (**A**) Concentration of miR-342-3p in exosome and total serum (normalized to snoRNA202 and snoRNA234 or cel-mir-39). Data analyzed by one-way ANOVA with a Tukey test. (**B**) Relative gene expression of various genes in brown adipose and epididymal adipose tissues (normalized to *Rplp0* and *Rn18s*) in HFHS chow. (**C**) Concentration of miR-342-3p in hypothalamus and ventral midbrain region (normalized to snoRNA202 and snoRNA234). Data shown as mean ± SD and analyzed by independent *t*-test (*p<0.05, **p<0.01).

Supplemental Figure 4

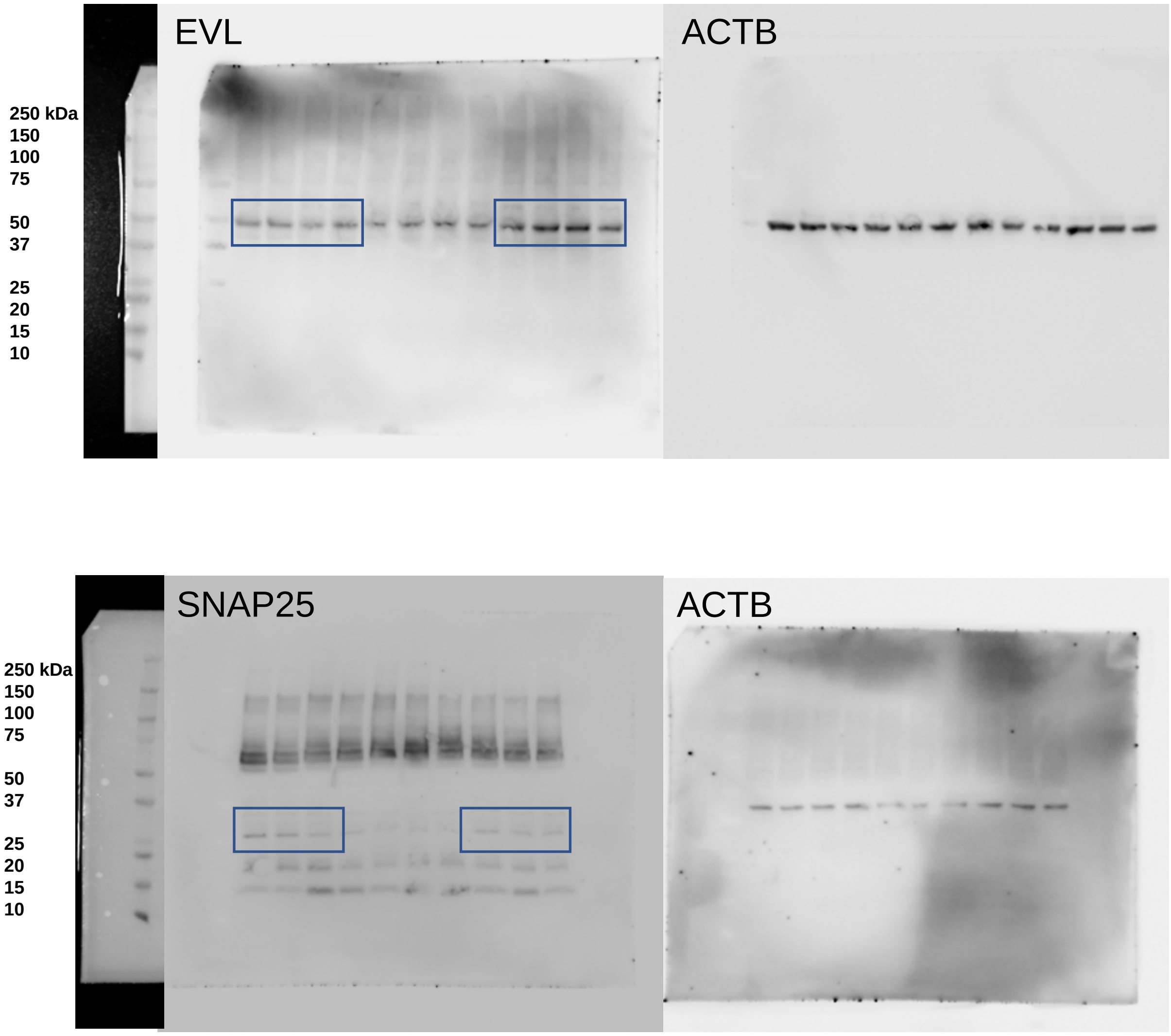

Supplementary Figure 4. Uncropped Western blot images of Enabled / Vasodilator-stimulated phosphoprotein (EVL), β-actin (ACTB) and synaptosomal-associated protein, 25kDa (SNAP25).

Supplementary Figure 5

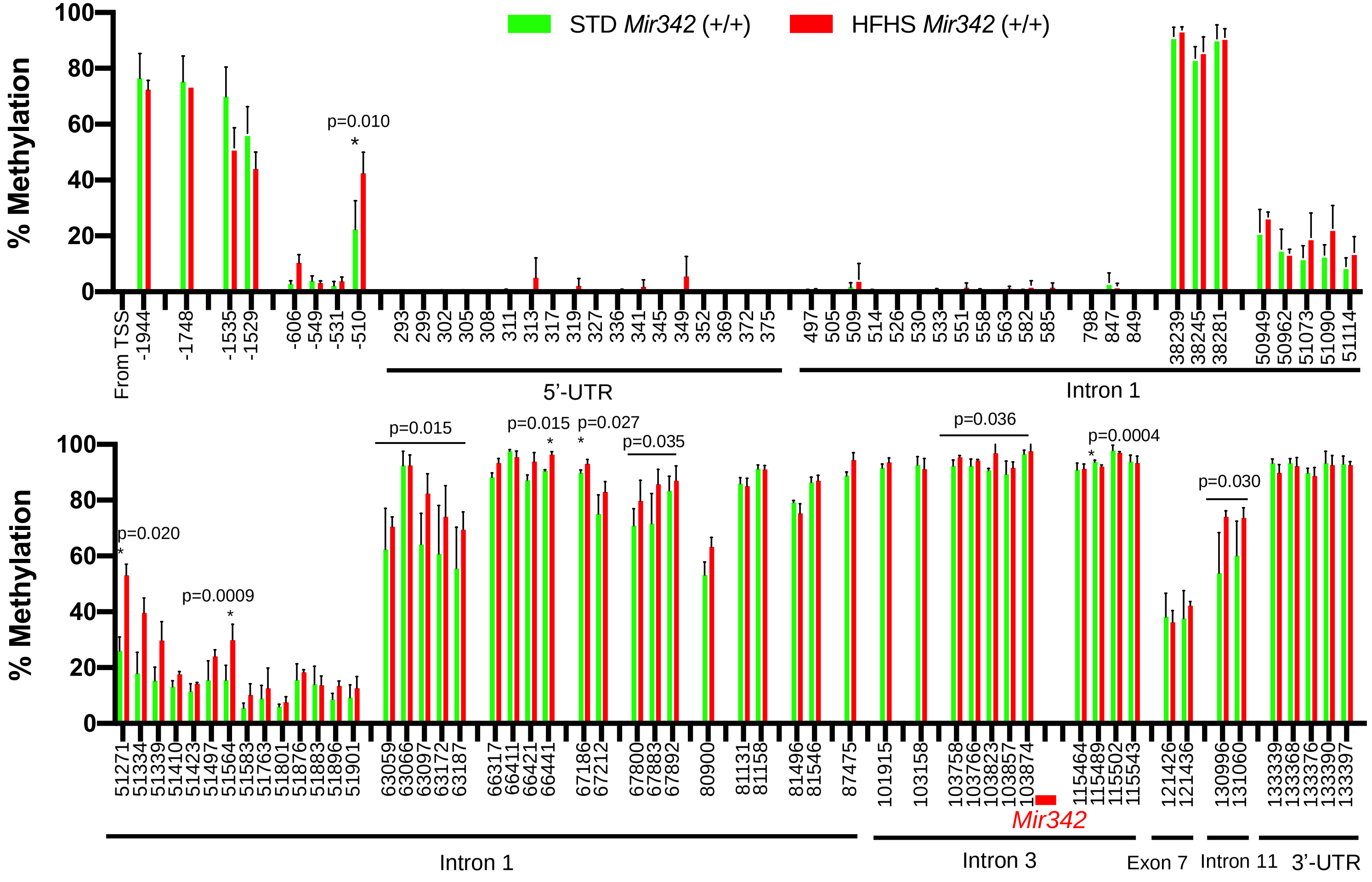

Supplementary Figure 5. Methylation status of *Evl* gene. Data shown as mean ± SD. Fisher's exact test was applied at each CpG site, and Mann–Whitney *U* test was used by NGS methylation assay (*p<0.05).

Supplementary Figure 6

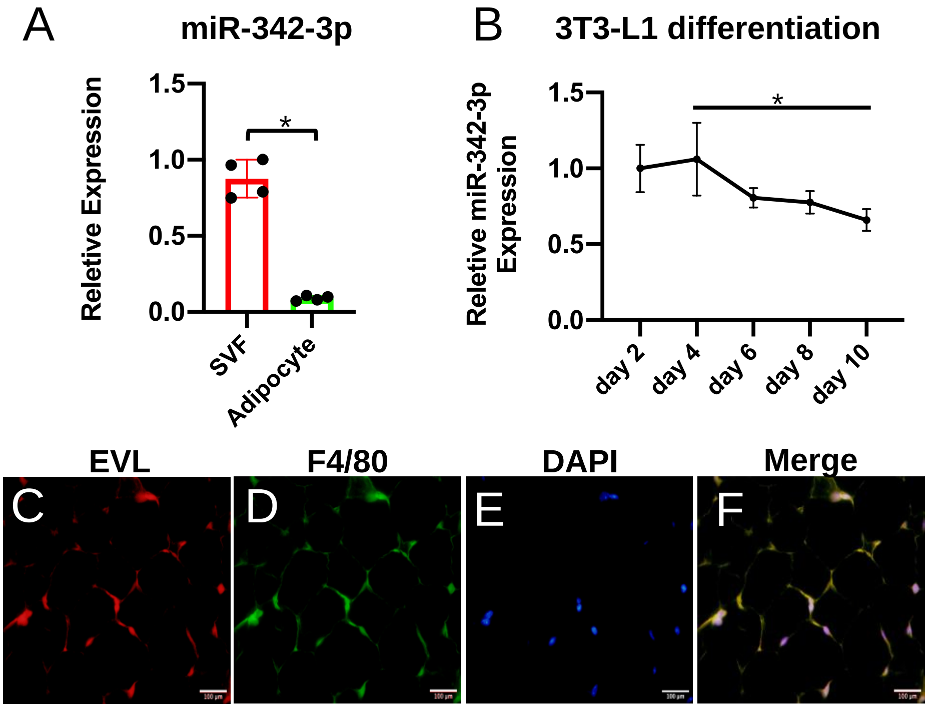

Supplementary Figure 6. Expression of *Mir342* in adipose tissues. (**A**) Levels of mmu-miR-342-3p in stromal vascular fraction (SVF) and mature adipocyte (n=4). (**B**) Expression of mmu-miR-342-3p declined during the differentiation of 3T3-L1 cells. (**C-F**) Triple immunostainings of EVL (C, red), F4/80 (D, green) and DAPI (E, blue) in epididymal adipose tissue derived from *Mir342 (+/+)* mice are shown. Data shown as mean ± SD and analyzed by independent *t*-test (*p<0.05).

Supplementary Figure 7

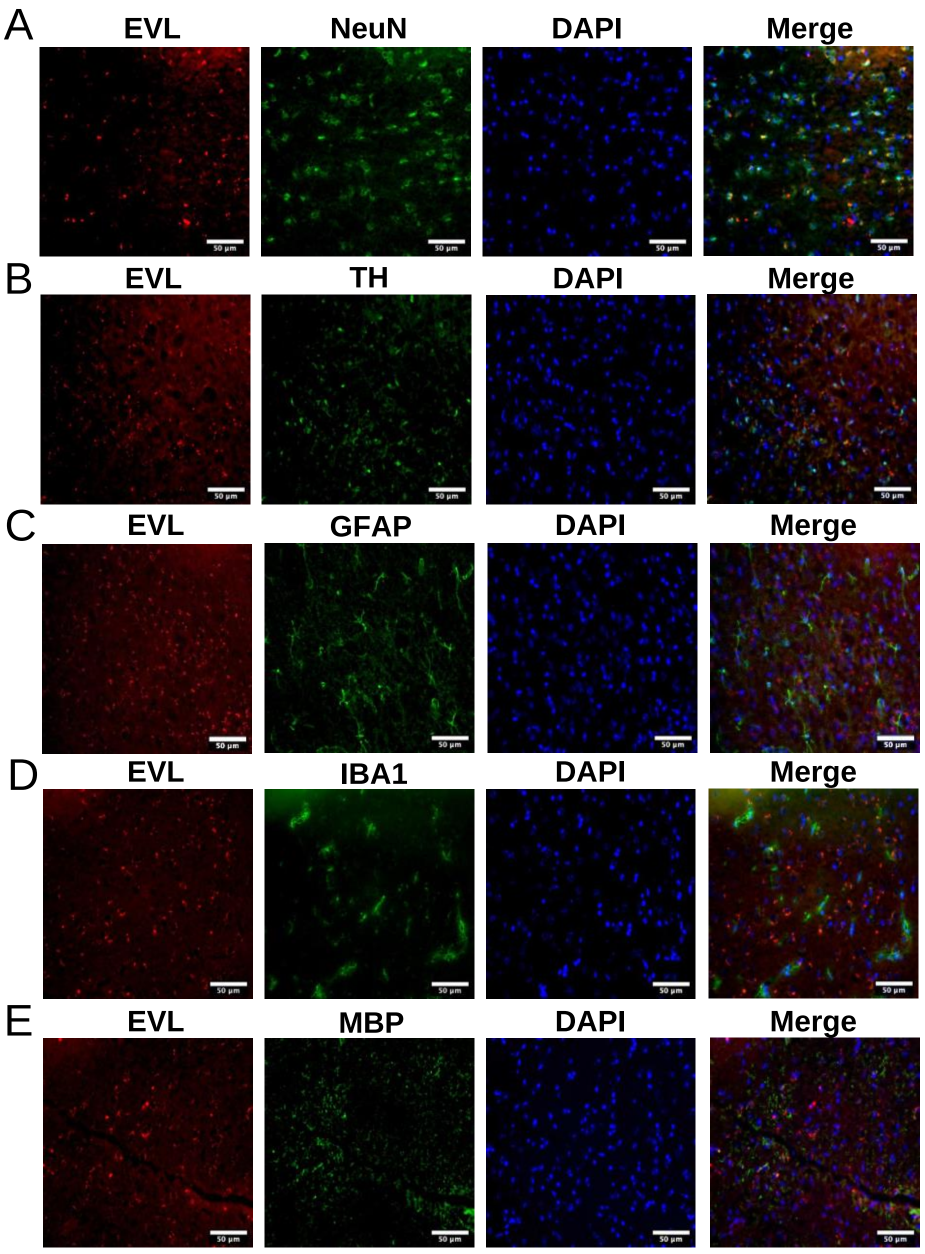

Supplementary Figure 7. Double immunostaining for EVL with various neuron and glial cell markers in cerebral cortex in *Mir342* (+/+) mice. EVL (red) staining with (**A**) NeuN (neuronal nuclei), (**B**) TH (tyrosine hydroxylase), (**C**) GFAP (glial fibrillary acidic protein), (**D**) IBA1 (ionized calcium binding adaptor molecule 1), (**E**) MBP (myeline basic protein), and DAPI (blue).

Supplementary Figure 8

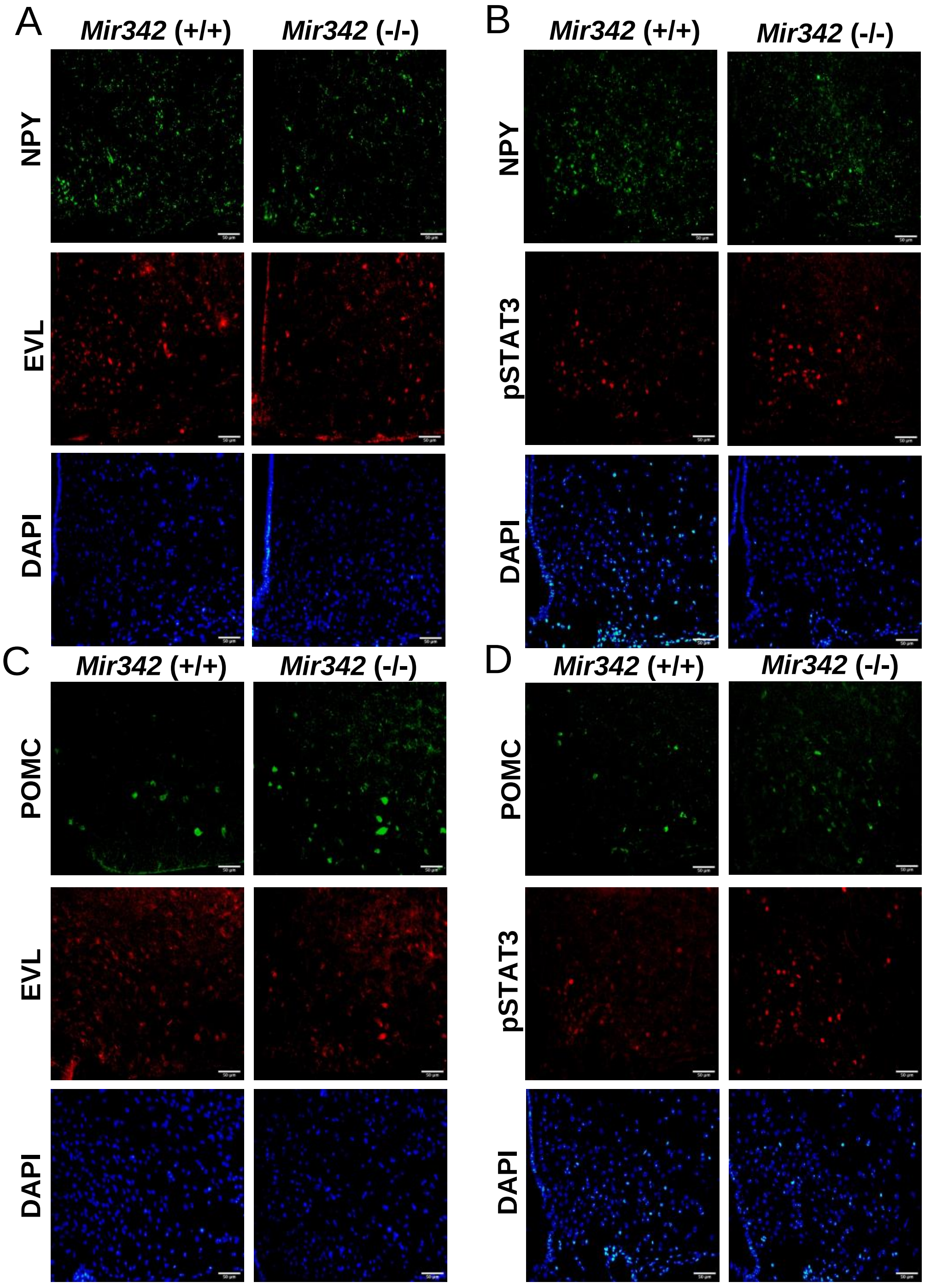

Supplementary Figure 8. Immunostaining of EVL, Neuropeptide Y (NPY), proopiomelanocortin (POMC), and phosphorylated signal transducer and activator of transcription 3 (pSTAT3) in hypothalamus derived from *Mir342* (+/+) and *Mir342* (-/-) mice fed with HFHS chow. (**A**) Immunostaining of EVL (red), NPY (green), and DAPI (blue). (**B**) Immunostaining of pSTAT3 (red), NPY (green), and DAPI (blue). (**C**) Immunostaining of EVL (red), POMC (green), and DAPI (blue). (**D**) Immunostaining of pSTAT3 (red), POMC (green), and DAPI (blue).

Supplementary Figure 9

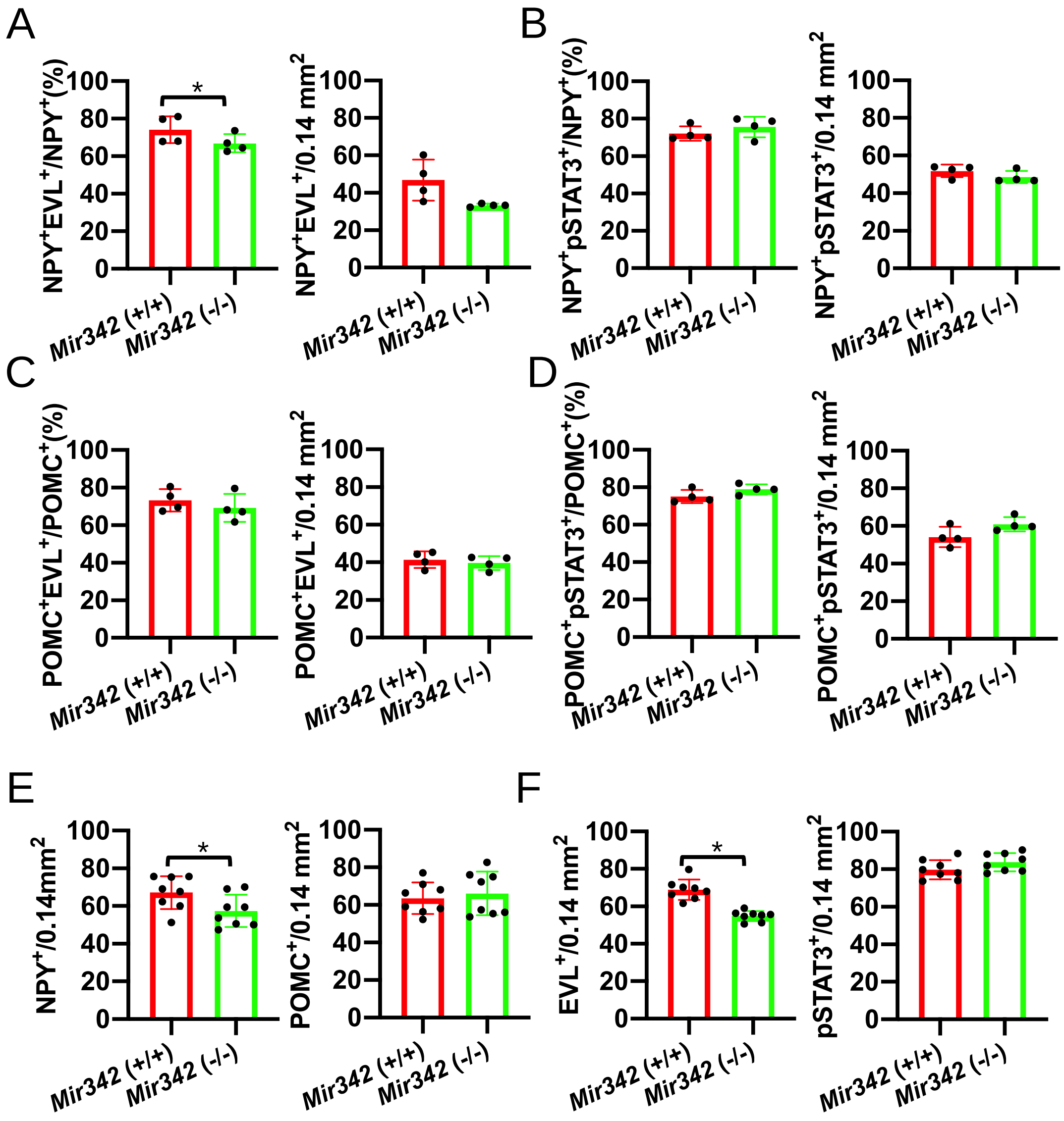

Supplementary Figure 9. The activation of NPY and POMC neurons by leptin injection in mice fed with STD chow. (**A**) The percentage and total numbers of NPY^+EVL^+ cells are shown. (**B**) The percentage and total numbers of $NPY^+pSTAT3^+$cells are shown. (**C**) The percentage and total numbers of $POMC^+EVL^+$ cells are shown. (**D**) The percentage and total numbers of $POMC^+pSTAT3^+$ cells are shown. (**E**) Average cell numbers of NPY^+ (n=8) and $POMC^+$ (n=8) cells detected in hypothalamus. (**F**) Average cell numbers of EVL^+ (n=8) and $pSTAT3^+$(n=8) cells detected in hypothalamus. Data shown as mean ± SD and analyzed by independent t-test (*$p<0.05$).

Supplementary Figure 10

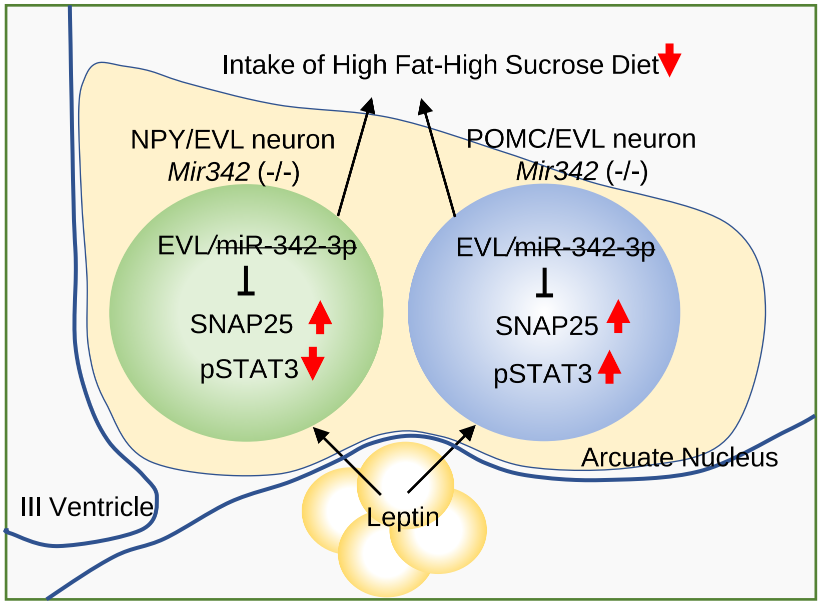

Supplementary Figure 10. Schematic drawing of mechanism of reduced food intake in *Mir342* (-/-) mice. miR-342-3p and its host gene *Evl* are highly upregulated in arcuate nucleus neurons. *Mir342* knockout mice showed reduced NPY+pSTAT3+ and increased POMC+pSTAT3+ hypothalamic neurons and were protected from diet-induced obesity and diabetes. The inhibition of miR-342-3p may be beneficial in appetite control in obesity.
