# Supplementary material for: Upregulation of Mir342 in Diet-Induced Obesity Mouse and the Hypothalamic Appetite Control
Source: Front Endocrinol (Lausanne). 2021 Aug 30;12:727915. doi: 10.3389/fendo.2021.727915 (PMC8437242; doi:10.3389/fendo.2021.727915)
Supplement: Supplementary file 3 [file Table_1.pdf]

Supplementary Table 1. RNA sequencing data of sera, liver and epididymalfat tissues in C57BL/6J mice fed with standard (STD) and high fat-high sucrose chow (HFHS).

miRBase Name	miRBase Accession	LINK	s1(Serum-High fat high sucrose chow)	s2(Liver-High fat high sucrose chow)	s3 (Epididymal fat-High fat high sucrose chow)	s5 (Serum-Normal chow)	s6 (Liver-Normal chow)	s7 (Epididymal fat-Normal chow)	Total	s1/s5	s2/s6	s3/s7
mmu-mir-22	MI0000570	miRBase_LINK	338,851	3,965,596	4,079,202	420,007	4,183,928	2,763,563	15,751,147	0.807	0.948	1.476
mmu-mir-10b	MI0000221	miRBase_LINK	54,948	1,404	2,277,812	74,813	2,390	2,626,307	5,037,674	0.734	0.587	0.867
mmu-mir-143	MI0000257	miRBase_LINK	26,388	278,402	2,360,422	31,475	452,685	2,327,301	5,476,673	0.838	0.615	1.014
mmu-mir-30a	MI0000144	miRBase_LINK	138,290	1,092,020	1,250,491	195,513	1,174,404	1,551,606	5,402,324	0.707	0.930	0.806
mmu-mir-10a	MI0000685	miRBase_LINK	43,250	244,759	1,008,625	60,325	314,615	935,762	2,607,336	0.717	0.778	1.078
mmu-mir-26a-2	MI0000706	miRBase_LINK	20,875	307,684	793,505	25,269	366,153	780,703	2,294,189	0.826	0.840	1.016
mmu-mir-26a-1	MI0000573	miRBase_LINK	20,871	307,545	793,290	25,267	365,994	780,582	2,293,549	0.826	0.840	1.016
mmu-let-7f-2	MI0000563	miRBase_LINK	16,610	684,828	778,838	25,149	593,665	604,641	2,703,731	0.660	1.154	1.288
mmu-let-7f-1	MI0000562	miRBase_LINK	11,642	621,181	731,371	17,964	543,185	572,647	2,497,990	0.648	1.144	1.277
mmu-mir-21	MI0000569	miRBase_LINK	33,913	675,905	833,457	57,300	872,282	459,536	2,932,393	0.592	0.775	1.814
mmu-mir-181a-1	MI0000697	miRBase_LINK	50,781	54,617	675,748	91,806	53,250	380,583	1,306,785	0.553	1.026	1.776
mmu-mir-181a-2	MI0000223	miRBase_LINK	50,960	54,780	672,973	92,313	53,412	380,437	1,304,875	0.552	1.026	1.769
mmu-mir-378	MI0000795	miRBase_LINK	37,243	237,812	234,917	55,688	171,521	366,144	1,103,325	0.669	1.386	0.642
mmu-let-7c-2	MI0000560	miRBase_LINK	72,776	164,719	345,608	118,692	170,389	313,353	1,185,537	0.613	0.967	1.103
mmu-let-7c-1	MI0000559	miRBase_LINK	72,525	161,513	342,882	118,216	167,212	311,496	1,173,844	0.613	0.966	1.101
mmu-mir-126	MI0000153	miRBase_LINK	13,581	210,617	507,382	15,175	201,422	301,182	1,249,359	0.895	1.046	1.685
mmu-mir-148a	MI0000550	miRBase_LINK	10,609	903,788	326,358	11,294	998,441	270,135	2,520,625	0.939	0.905	1.208
mmu-let-7a-1	MI0000556	miRBase_LINK	22,232	229,736	311,658	32,557	244,268	263,340	1,103,791	0.683	0.941	1.183
mmu-let-7a-2	MI0000557	miRBase_LINK	22,147	227,308	310,107	32,488	242,059	262,321	1,096,430	0.682	0.939	1.182
mmu-mir-30d	MI0000549	miRBase_LINK	86,140	170,538	352,452	146,223	200,896	251,104	1,207,353	0.589	0.849	1.404
mmu-mir-30c-2	MI0000548	miRBase_LINK	45,632	194,924	210,330	53,221	169,668	214,907	888,682	0.857	1.149	0.979
mmu-mir-30c-1	MI0000547	miRBase_LINK	45,487	191,433	207,508	53,007	166,348	209,769	873,552	0.858	1.151	0.989
mmu-mir-27b	MI0000142	miRBase_LINK	22,994	253,279	390,605	29,301	245,960	193,911	1,136,050	0.785	1.030	2.014
mmu-mir-191	MI0000233	miRBase_LINK	65,738	70,411	197,169	98,887	77,767	166,952	676,924	0.665	0.905	1.181
mmu-mir-103-2	MI0000588	miRBase_LINK	27,499	42,040	142,351	29,023	28,914	164,768	434,595	0.947	1.454	0.864
mmu-mir-103-1	MI0000587	miRBase_LINK	27,496	41,808	140,803	29,020	28,764	163,598	431,489	0.947	1.453	0.861
mmu-mir-486	MI0003493	miRBase_LINK	1,715,391	31,647	92,856	2,085,838	21,114	157,644	4,104,490	0.822	1.499	0.589
mmu-mir-16-1	MI0000565	miRBase_LINK	270,173	109,350	189,081	274,399	112,265	113,203	1,068,471	0.985	0.974	1.670
mmu-mir-16-2	MI0000566	miRBase_LINK	270,034	108,238	187,705	274,225	111,159	112,639	1,064,000	0.985	0.974	1.666
mmu-let-7i	MI0000138	miRBase_LINK	36,717	37,261	191,131	43,314	35,448	103,687	447,558	0.848	1.051	1.843
mmu-mir-29a	MI0000576	miRBase_LINK	10,752	77,404	98,142	13,359	82,945	99,229	381,831	0.805	0.933	0.989
mmu-let-7g	MI0000137	miRBase_LINK	24,376	121,676	109,960	28,263	143,036	88,635	515,946	0.862	0.851	1.241
mmu-mir-30e	MI0000259	miRBase_LINK	61,653	170,748	125,889	80,846	193,521	86,176	718,833	0.763	0.882	1.461
mmu-mir-125a	MI0000151	miRBase_LINK	4,192	10,692	72,514	6,653	16,382	84,101	194,534	0.630	0.653	0.862
mmu-mir-151	MI0000173	miRBase_LINK	9,486	31,203	95,030	15,283	27,328	78,781	257,111	0.621	1.142	1.206
mmu-mir-130a	MI0000156	miRBase_LINK	4,735	34,984	61,132	6,012	27,528	71,682	206,073	0.788	1.271	0.853
mmu-mir-26b	MI0000575	miRBase_LINK	4,225	87,418	89,191	4,349	85,187	70,989	341,359	0.971	1.026	1.256
mmu-mir-92a-1	MI0000719	miRBase_LINK	261,995	104,687	72,981	307,148	106,440	66,478	919,729	0.853	0.984	1.098
mmu-let-7b	MI0000558	miRBase_LINK	8,013	34,832	68,099	13,088	33,239	66,095	223,366	0.612	1.048	1.030
mmu-let-7d	MI0000405	miRBase_LINK	19,639	51,637	74,003	28,501	42,117	64,215	280,112	0.689	1.226	1.152
mmu-mir-199a-2	MI0000713	miRBase_LINK	4,297	18,289	111,987	9,449	25,297	55,364	224,683	0.455	0.723	2.023
mmu-mir-199a-1	MI0000241	miRBase_LINK	4,296	18,270	111,847	9,449	25,278	55,294	224,434	0.455	0.723	2.023
mmu-mir-199b	MI0000714	miRBase_LINK	4,178	17,125	107,930	9,223	24,025	54,220	216,701	0.453	0.713	1.991
mmu-mir-451	MI0001730	miRBase_LINK	175,527	26,684	63,120	155,992	15,663	52,887	489,873	1.125	1.704	1.193
mmu-mir-150	MI0000172	miRBase_LINK	36,117	2,616	40,846	54,152	1,326	50,744	185,801	0.667	1.973	0.805
mmu-mir-25	MI0000689	miRBase_LINK	195,450	19,655	47,352	192,381	19,725	49,959	524,522	1.016	0.996	0.948
mmu-mir-30b	MI0000145	miRBase_LINK	13,651	52,808	70,119	16,874	43,695	46,626	243,773	0.809	1.209	1.504
mmu-mir-497	MI0004636	miRBase_LINK	905	5,291	62,720	1,108	5,683	44,269	119,976	0.817	0.931	1.417
mmu-mir-101a	MI0000148	miRBase_LINK	1,277	88,769	58,100	1,312	74,119	43,132	266,709	0.973	1.198	1.347
mmu-mir-27a	MI0000578	miRBase_LINK	2,777	8,389	65,837	3,388	8,827	42,164	131,382	0.820	0.950	1.561
mmu-mir-145	MI0000169	miRBase_LINK	527	4,783	34,032	739	6,085	40,619	86,785	0.713	0.786	0.838
mmu-mir-127	MI0000154	miRBase_LINK	1,751	714	68,083	3,850	1,120	38,800	114,318	0.455	0.638	1.755
mmu-mir-125b-2	MI0000152	miRBase_LINK	1,701	6,853	31,682	3,133	7,909	37,227	88,505	0.543	0.866	0.851
mmu-mir-125b-1	MI0000725	miRBase_LINK	1,654	5,705	27,240	3,087	6,755	33,021	77,462	0.536	0.845	0.825
mmu-mir-186	MI0000228	miRBase_LINK	20,174	17,459	51,457	22,453	16,000	31,850	159,393	0.898	1.091	1.616
mmu-mir-193	MI0000235	miRBase_LINK	60	20,655	24,669	55	13,998	30,595	90,032	1.091	1.476	0.806
mmu-mir-23a	MI0000571	miRBase_LINK	4,046	3,914	44,281	7,353	5,152	28,227	92,973	0.550	0.760	1.569
mmu-mir-93	MI0000581	miRBase_LINK	42,293	14,738	27,822	42,227	14,053	26,003	167,136	1.002	1.049	1.070
mmu-let-7e	MI0000561	miRBase_LINK	534	3,481	28,628	1,146	5,389	23,560	62,738	0.466	0.646	1.215
mmu-mir-142	MI0000167	miRBase_LINK	37,026	17,590	58,187	39,676	9,631	22,754	184,864	0.933	1.826	2.557
mmu-mir-99b	MI0000147	miRBase_LINK	1,551	2,466	21,502	2,364	3,736	21,904	53,523	0.656	0.660	0.982
mmu-mir-107	MI0000684	miRBase_LINK	21,491	29,102	18,384	21,360	16,679	21,326	128,342	1.006	1.745	0.862
mmu-mir-181b-2	MI0000823	miRBase_LINK	3,670	2,242	28,133	6,614	2,261	19,128	62,048	0.555	0.992	1.471
mmu-mir-101b	MI0000649	miRBase_LINK	1,894	215,325	19,259	1,459	154,713	18,731	411,381	1.298	1.392	1.028
mmu-mir-181b-1	MI0000723	miRBase_LINK	3,625	2,174	27,208	6,530	2,188	18,561	60,286	0.555	0.994	1.466
mmu-mir-181c	MI0000724	miRBase_LINK	5,818	5,782	34,967	8,210	10,013	18,384	83,174	0.709	0.577	1.902
mmu-mir-434	MI0001526	miRBase_LINK	2,057	403	37,349	5,758	670	18,279	64,516	0.357	0.601	2.043
mmu-mir-195	MI0000237	miRBase_LINK	245	2,198	23,070	321	2,860	16,523	45,217	0.763	0.769	1.396
mmu-mir-140	MI0000165	miRBase_LINK	47,426	11,017	18,802	58,767	7,978	16,400	160,390	0.807	1.381	1.146
mmu-mir-24-2	MI0000572	miRBase_LINK	790	3,781	25,120	1,311	4,695	15,056	50,753	0.603	0.805	1.668
mmu-mir-411	MI0001163	miRBase_LINK	136	350	35,343	272	498	14,732	51,331	0.500	0.703	2.399
mmu-mir-192	MI0000551	miRBase_LINK	55,437	2,530,616	20,099	55,249	2,670,751	14,550	5,346,702	1.003	0.948	1.381
mmu-mir-423	MI0004637	miRBase_LINK	55,077	14,248	16,921	89,556	11,061	13,761	200,624	0.615	1.288	1.230
mmu-mir-99a	MI0000146	miRBase_LINK	275	3,886	10,906	526	4,188	13,606	33,387	0.523	0.928	0.802
mmu-mir-100	MI0000692	miRBase_LINK	269	1,910	12,916	476	2,406	12,493	30,470	0.565	0.794	1.034
mmu-mir-98	MI0000586	miRBase_LINK	1,262	8,984	18,009	1,521	8,447	11,921	50,144	0.830	1.064	1.511
mmu-mir-92a-2	MI0000580	miRBase_LINK	99,452	9,614	12,718	113,997	8,089	11,624	255,494	0.872	1.189	1.094
mmu-mir-203	MI0000246	miRBase_LINK	1,701	23,074	11,688	4,293	23,155	11,380	75,291	0.396	0.997	1.027
mmu-mir-15a	MI0000564	miRBase_LINK	13,121	19,585	20,015	12,934	17,333	11,210	94,198	1.014	1.130	1.785
mmu-mir-29c	MI0000577	miRBase_LINK	808	23,420	10,962	867	17,393	10,512	63,962	0.932	1.347	1.043
mmu-mir-322	MI0000590	miRBase_LINK	281	1,774	18,296	320	1,581	9,847	32,099	0.878	1.122	1.858
mmu-mir-532	MI0003206	miRBase_LINK	3,030	3,678	13,418	4,082	3,515	9,271	36,994	0.742	1.046	1.447
mmu-mir-365-2	MI0001645	miRBase_LINK	192	2,938	9,649	192	2,696	8,997	24,664	1.000	1.090	1.072
mmu-mir-365-1	MI0000768	miRBase_LINK	184	2,901	9,541	186	2,657	8,918	24,387	0.989	1.092	1.070
mmu-mir-335	MI0000817	miRBase_LINK	137	2,673	110,365	54	1,504	8,778	123,511	2.537	1.777	12.573
mmu-mir-23b	MI0000141	miRBase_LINK	485	8,475	16,917	745	8,883	8,549	44,054	0.651	0.954	1.979
mmu-mir-24-1	MI0000231	miRBase_LINK	335	2,267	13,600	604	2,923	7,887	27,616	0.555	0.776	1.724
mmu-mir-541	MI0003521	miRBase_LINK	769	207	15,707	1,541	288	7,593	26,105	0.499	0.719	2.069
mmu-mir-708	MI0004692	miRBase_LINK	1	8	11,079	7	0	7,466	18,561	0.143	#DIV/0!	1.484
mmu-mir-28	MI0000690	miRBase_LINK	691	7,653	8,991	1,027	5,588	7,377	31,327	0.673	1.370	1.219
mmu-mir-340	MI0000623	miRBase_LINK	1,859	27,006	19,256	2,661	22,296	7,227	80,305	0.699	1.211	2.664
mmu-mir-425	MI0001447	miRBase_LINK	5,323	4,145	8,496	7,343	3,756	6,993	36,056	0.725	1.104	1.215
mmu-mir-689-2			17,105	10,236	8,756	32,701	9,330	6,780	84,908	0.523	1.097	1.291
mmu-mir-221	MI0000709	miRBase_LINK	7,180	6,724	38,010	9,165	7,541	6,710	75,330	0.783	0.892	5.665

mmu-mir-146b	MI0004665	miRBase_LINK	663	676	20,505	1,766	859	6,707	31,176	0.375	0.787	3.057
mmu-mir-320	MI0000704	miRBase_LINK	6,202	2,127	6,702	8,368	2,369	6,222	31,990	0.741	0.898	1.077
mmu-mir-805			66	34,480	8,415	105	50,488	6,215	99,769	0.629	0.683	1.354
mmu-mir-152	MI0000174	miRBase_LINK	183	2,975	10,113	298	2,440	6,141	22,150	0.614	1.219	1.647
mmu-mir-139	MI0000693	miRBase_LINK	339	2,056	9,577	382	1,835	6,106	20,295	0.887	1.120	1.568
mmu-mir-872	MI0005549	miRBase_LINK	680	3,287	8,002	1,134	3,115	5,662	21,880	0.600	1.055	1.413
mmu-mir-144	MI0000168	miRBase_LINK	1,799	4,959	6,557	2,217	2,337	5,264	23,133	0.811	2.122	1.246
mmu-mir-676	MI0005003	miRBase_LINK	3,001	1,716	5,549	4,209	1,221	5,249	20,945	0.713	1.405	1.057
mmu-mir-133a-1	MI0000159	miRBase_LINK	41,161	90	2,675	79,581	146	4,555	128,208	0.517	0.616	0.587
mmu-mir-133a-2	MI0000820	miRBase_LINK	41,106	89	2,673	79,491	146	4,553	128,058	0.517	0.610	0.587
mmu-mir-193b	MI0005484	miRBase_LINK	168	120	2,000	265	111	4,108	6,772	0.634	1.081	0.487
mmu-mir-744	MI0004124	miRBase_LINK	5,312	3,379	5,628	8,151	2,647	4,085	29,202	0.652	1.277	1.378
mmu-mir-484	MI0003491	miRBase_LINK	4,496	1,844	4,994	5,858	1,315	4,045	22,552	0.767	1.402	1.235
mmu-mir-351	MI0000643	miRBase_LINK	272	2,552	5,554	463	2,370	3,998	15,209	0.587	1.077	1.389
mmu-mir-34c	MI0000403	miRBase_LINK	230	153	7,548	295	357	3,585	12,168	0.780	0.429	2.105
mmu-mir-136	MI0000162	miRBase_LINK	182	185	8,157	428	288	3,543	12,783	0.425	0.642	2.302
mmu-mir-339	MI0000621	miRBase_LINK	312	1,102	2,563	491	1,106	3,206	8,780	0.635	0.996	0.799
mmu-mir-223	MI0000703	miRBase_LINK	1,534	857	7,087	2,369	671	3,189	15,707	0.648	1.277	2.222
mmu-mir-328	MI0000603	miRBase_LINK	4,943	730	2,213	6,840	696	3,091	18,513	0.723	1.049	0.716
mmu-mir-15b	MI0000140	miRBase_LINK	3,219	1,918	5,791	3,407	1,569	3,074	18,978	0.945	1.222	1.884
mmu-mir-34a	MI0000584	miRBase_LINK	31	784	5,181	63	427	2,838	9,324	0.492	1.836	1.826
mmu-mir-146a	MI0000170	miRBase_LINK	2,113	2,851	9,532	4,266	4,742	2,822	26,326	0.495	0.601	3.378
mmu-mir-222	MI0000710	miRBase_LINK	1,039	1,763	22,488	2,231	1,578	2,759	31,858	0.466	1.117	8.151
mmu-mir-204	MI0000247	miRBase_LINK	242	1	2,752	376	0	2,661	6,032	0.644	#DIV/0!	1.034
mmu-mir-301a	MI0000401	miRBase_LINK	1,103	1,619	4,351	1,210	1,203	2,629	12,115	0.912	1.346	1.655
mmu-mir-381	MI0000798	miRBase_LINK	155	59	4,680	340	85	2,600	7,919	0.456	0.694	1.800
mmu-mir-574	MI0005518	miRBase_LINK	1,276	1,495	3,443	2,826	1,397	2,512	12,949	0.452	1.070	1.371
mmu-mir-29b-1	MI0000143	miRBase_LINK	74	3,496	2,779	103	3,291	2,396	12,139	0.718	1.062	1.160
mmu-mir-29b-2	MI0000712	miRBase_LINK	82	3,524	2,730	111	3,061	2,393	11,901	0.739	1.151	1.141
mmu-mir-410	MI0001161	miRBase_LINK	149	51	5,073	477	40	2,383	8,173	0.312	1.275	2.129
mmu-mir-342	MI0000627	miRBase_LINK	2,027	1,124	19,320	3,427	707	2,040	28,645	0.591	1.590	9.471
mmu-mir-148b	MI0000617	miRBase_LINK	513	3,255	3,523	540	2,943	1,992	12,766	0.950	1.106	1.769
mmu-mir-106b	MI0000407	miRBase_LINK	15,968	493	1,681	16,387	347	1,948	36,824	0.974	1.421	0.863
mmu-mir-361	MI0000761	miRBase_LINK	636	1,706	2,271	802	1,446	1,938	8,799	0.793	1.180	1.172
mmu-mir-215	MI0000974	miRBase_LINK	32,484	494	245	50,380	685	1,930	86,218	0.645	0.721	0.127
mmu-mir-669a-1	MI0004523	miRBase_LINK	165	512	4,545	668	416	1,880	8,186	0.247	1.231	2.418
mmu-mir-149	MI0000171	miRBase_LINK	1,128	2,485	2,990	1,681	598	1,635	10,517	0.671	4.156	1.829
mmu-mir-19b-1	MI0000718	miRBase_LINK	1,366	5,002	2,546	1,374	5,222	1,633	17,143	0.994	0.958	1.559
mmu-mir-19b-2	MI0000546	miRBase_LINK	1,368	5,031	2,540	1,375	5,241	1,632	17,187	0.995	0.960	1.556
mmu-mir-350	MI0000640	miRBase_LINK	340	422	1,977	429	427	1,619	5,214	0.793	0.988	1.221
mmu-mir-218-1	MI0000700	miRBase_LINK	30	19	1,432	41	23	1,488	3,033	0.732	0.826	0.962
mmu-mir-300	MI0000400	miRBase_LINK	65	41	2,666	179	53	1,469	4,473	0.363	0.774	1.815
mmu-mir-218-2	MI0000701	miRBase_LINK	22	19	1,392	34	23	1,458	2,948	0.647	0.826	0.955
mmu-mir-194-1	MI0000236	miRBase_LINK	646	159,842	2,109	615	142,468	1,435	307,115	1.050	1.122	1.470
mmu-mir-181d	MI0005450	miRBase_LINK	1,317	353	2,840	2,036	506	1,429	8,481	0.647	0.698	1.987
mmu-mir-194-2	MI0000733	miRBase_LINK	636	160,390	2,112	566	142,987	1,393	308,084	1.124	1.122	1.516
mmu-mir-685			728	1,949	1,744	1,116	1,585	1,382	8,504	0.652	1.230	1.262
mmu-mir-689-1			1,919	2,643	2,029	2,160	2,465	1,349	12,565	0.888	1.072	1.504
mmu-mir-17	MI0000687	miRBase_LINK	1,299	1,972	2,333	1,142	1,540	1,340	9,626	1.137	1.281	1.741
mmu-mir-326	MI0000598	miRBase_LINK	212	287	2,204	313	417	1,335	4,768	0.677	0.688	1.651
mmu-mir-214	MI0000698	miRBase_LINK	170	682	3,121	250	655	1,245	6,123	0.680	1.041	2.507
mmu-mir-615	MI0005004	miRBase_LINK	18	1	1,077	55	3	1,222	2,376	0.327	0.333	0.881
mmu-mir-20a	MI0000568	miRBase_LINK	239	2,168	1,656	306	1,918	1,114	7,401	0.781	1.130	1.487
mmu-mir-345	MI0000632	miRBase_LINK	202	830	660	238	935	1,106	3,971	0.849	0.888	0.597
mmu-mir-671	MI0004133	miRBase_LINK	149	1,253	987	220	993	1,060	4,662	0.677	1.262	0.931
mmu-mir-138-2	MI0000164	miRBase_LINK	271	98	1,608	488	60	1,059	3,584	0.555	1.633	1.518
mmu-mir-210	MI0000695	miRBase_LINK	296	369	709	278	348	1,038	3,038	1.065	1.060	0.683
mmu-mir-132	MI0000158	miRBase_LINK	471	396	1,704	621	356	1,010	4,558	0.758	1.112	1.687
mmu-mir-338	MI0000619	miRBase_LINK	58	581	1,375	64	681	910	3,669	0.906	0.853	1.511
mmu-mir-450a-1	MI0001653	miRBase_LINK	22	514	1,665	16	547	903	3,667	1.375	0.940	1.844
mmu-mir-450a-2	MI0003537	miRBase_LINK	22	512	1,654	17	549	901	3,655	1.294	0.933	1.836
mmu-mir-182	MI0000224	miRBase_LINK	1,221	16,422	1,562	1,891	12,825	888	34,809	0.646	1.280	1.759
mmu-mir-409	MI0001160	miRBase_LINK	125	25	1,891	374	40	857	3,312	0.334	0.625	2.207
mmu-mir-674	MI0004611	miRBase_LINK	417	377	1,656	623	267	836	4,176	0.669	1.412	1.981
mmu-mir-324	MI0000595	miRBase_LINK	372	297	956	432	198	775	3,030	0.861	1.500	1.234
mmu-mir-467a			8	170	1,476	8	171	716	2,549	1.000	0.994	2.061
mmu-mir-421	MI0005496	miRBase_LINK	2,166	285	1,204	2,543	263	694	7,155	0.852	1.084	1.735
mmu-mir-128-1	MI0000155	miRBase_LINK	3,329	596	1,171	5,477	450	688	11,711	0.608	1.324	1.702
mmu-mir-450b	MI0004705	miRBase_LINK	35	278	1,238	37	257	670	2,515	0.946	1.082	1.848
mmu-mir-224	MI0000711	miRBase_LINK	86	57	723	145	74	664	1,749	0.593	0.770	1.089
mmu-mir-32	MI0000691	miRBase_LINK	22	1,000	1,235	7	656	650	3,570	3.143	1.524	1.900
mmu-mir-374	MI0004125	miRBase_LINK	124	744	892	138	797	622	3,317	0.899	0.934	1.434
mmu-mir-652	MI0004965	miRBase_LINK	1,121	348	1,394	1,288	265	616	5,032	0.870	1.313	2.263
mmu-mir-431	MI0001524	miRBase_LINK	41	11	1,110	158	9	579	1,908	0.259	1.222	1.917
mmu-mir-337	MI0000615	miRBase_LINK	53	11	1,263	115	21	574	2,037	0.461	0.524	2.200
mmu-mir-155	MI0000177	miRBase_LINK	123	122	1,336	251	105	542	2,479	0.490	1.162	2.465
mmu-mir-128-2	MI0000726	miRBase_LINK	2,971	452	944	4,817	322	540	10,046	0.617	1.404	1.748
mmu-mir-330	MI0000607	miRBase_LINK	57	128	505	118	111	524	1,443	0.483	1.153	0.964
mmu-mir-331	MI0000609	miRBase_LINK	59	313	543	66	246	497	1,724	0.894	1.272	1.093
mmu-mir-185	MI0000227	miRBase_LINK	91	1,001	765	91	901	462	3,311	1.000	1.111	1.656
mmu-mir-1198	MI0006306	miRBase_LINK	1,567	320	489	1,670	268	456	4,770	0.938	1.194	1.072
mmu-mir-511	MI0005554	miRBase_LINK	332	663	985	683	687	439	3,789	0.486	0.965	2.244
mmu-mir-329	MI0000605	miRBase_LINK	21	10	888	72	14	424	1,429	0.292	0.714	2.094
mmu-mir-341	MI0000625	miRBase_LINK	25	8	662	69	5	397	1,166	0.362	1.600	1.668
mmu-mir-362	MI0000763	miRBase_LINK	19	379	709	16	348	392	1,863	1.188	1.089	1.809
mmu-mir-379	MI0000796	miRBase_LINK	1	10	784	11	25	356	1,187	0.091	0.400	2.202
mmu-mir-582	MI0006127	miRBase_LINK	108	360	465	268	84	346	1,631	0.403	4.286	1.344
mmu-mir-500	MI0004702	miRBase_LINK	34	141	627	74	162	337	1,375	0.459	0.870	1.861
mmu-mir-187	MI0000229	miRBase_LINK	83	383	315	125	526	326	1,758	0.664	0.728	0.966
mmu-mir-455	MI0004679	miRBase_LINK	54	4,687	882	59	5,597	324	11,603	0.915	0.837	2.722
mmu-mir-299	MI0000399	miRBase_LINK	15	4	735	31	17	310	1,112	0.484	0.235	2.371
mmu-mir-92b	MI0005521	miRBase_LINK	382	43	341	684	119	309	1,878	0.558	0.361	1.104
mmu-mir-369	MI0003535	miRBase_LINK	4	11	718	29	12	291	1,065	0.138	0.917	2.467
mmu-mir-673	MI0004601	miRBase_LINK	58	7	453	168	4	277	967	0.345	1.750	1.635
mmu-mir-34b	MI0000404	miRBase_LINK	36	11	547	20	30	273	917	1.800	0.367	2.004
mmu-mir-130b	MI0000408	miRBase_LINK	4,382	91	614	5,414	88	260	10,849	0.809	1.034	2.362
mmu-mir-542	MI0003522	miRBase_LINK	17	224	534	31	185	259	1,250	0.548	1.211	2.062
mmu-mir-298	MI0000398	miRBase_LINK	188	28	3,175	379	30	249	4,049	0.496	0.933	12.751
mmu-mir-466b-1	MI0005502	miRBase_LINK	48	54	438	48	53	234	875	1.000	1.019	1.872
mmu-mir-503	MI0003538	miRBase_LINK	24	67	302	41	66	234	734	0.585	1.015	1.291

mmu-mir-190	MI0000232	miRBase_LINK	1	485	713	0	325	231	1,755	#DIV/0!	1.492	3.087
mmu-mir-540	MI0003518	miRBase_LINK	55	6	497	147	8	222	935	0.374	0.750	2.239
mmu-mir-7a-1	MI0000728	miRBase_LINK	51	465	333	33	492	217	1,591	1.545	0.945	1.535
mmu-mir-429	MI0001642	miRBase_LINK	421	833	177	887	1,335	215	3,868	0.475	0.624	0.823
mmu-mir-33	MI0000707	miRBase_LINK	40	725	244	44	431	212	1,696	0.909	1.682	1.151
mmu-mir-196b	MI0001151	miRBase_LINK	12	4	122	19	6	211	374	0.632	0.667	0.578
mmu-mir-154	MI0000176	miRBase_LINK	7	6	294	14	14	206	541	0.500	0.429	1.427
mmu-mir-212	MI0000696	miRBase_LINK	42	76	326	75	64	196	779	0.560	1.188	1.663
mmu-mir-200b	MI0000243	miRBase_LINK	69	965	216	171	1,558	188	3,167	0.404	0.619	1.149
mmu-mir-18a	MI0000567	miRBase_LINK	754	261	410	823	214	181	2,643	0.916	1.220	2.265
mmu-mir-669a-2	MI0004667	miRBase_LINK	15	49	439	62	41	180	786	0.242	1.195	2.439
mmu-mir-700	MI0004684	miRBase_LINK	27	52	258	23	72	163	595	1.174	0.722	1.583
mmu-mir-485	MI0003492	miRBase_LINK	48	7	338	157	3	160	713	0.306	2.333	2.113
mmu-mir-200a	MI0000554	miRBase_LINK	230	718	156	332	1,377	159	2,972	0.693	0.521	0.981
mmu-mir-699			8	122	195	30	144	158	657	0.267	0.847	1.234
mmu-mir-382	MI0000799	miRBase_LINK	7	4	333	38	8	150	540	0.184	0.500	2.220
mmu-mir-188	MI0000230	miRBase_LINK	10	58	218	32	49	148	515	0.313	1.184	1.473
mmu-mir-466c			24	33	276	21	45	144	543	1.143	0.733	1.917
mmu-mir-196a-2	MI0000553	miRBase_LINK	6	4	208	15	0	142	375	0.400	#DIV/0!	1.465
mmu-mir-501	MI0004703	miRBase_LINK	60	65	342	106	66	141	780	0.566	0.985	2.426
mmu-mir-376b	MI0001162	miRBase_LINK	3	9	362	11	14	136	535	0.273	0.643	2.662
mmu-mir-668	MI0004134	miRBase_LINK	27	0	224	96	2	136	485	0.281	0.000	1.647
mmu-mir-677	MI0004634	miRBase_LINK	5	364	237	22	363	135	1,126	0.227	1.003	1.756
mmu-mir-467d	MI0005513	miRBase_LINK	14	27	182	19	10	133	385	0.737	2.700	1.368
mmu-mir-133b	MI0000821	miRBase_LINK	525	2	99	844	5	133	1,608	0.622	0.400	0.744
mmu-mir-376a	MI0000793	miRBase_LINK	7	7	366	0	10	132	522	#DIV/0!	0.700	2.773
mmu-mir-877	MI0005553	miRBase_LINK	141	41	169	245	25	128	749	0.576	1.640	1.320
mmu-mir-134	MI0000160	miRBase_LINK	16	5	239	101	9	124	494	0.158	0.556	1.927
mmu-mir-376c	MI0003533	miRBase_LINK	2	8	249	6	3	120	388	0.333	2.667	2.075
mmu-mir-598	MI0005556	miRBase_LINK	12	162	264	5	176	118	737	2.400	0.920	2.237
mmu-mir-9-3	MI0000721	miRBase_LINK	3	25	259	9	6	116	418	0.333	4.167	2.233
mmu-mir-9-1	MI0000720	miRBase_LINK	3	25	259	9	6	115	417	0.333	4.167	2.252
mmu-mir-9-2	MI0000157	miRBase_LINK	3	25	259	9	6	115	417	0.333	4.167	2.252
mmu-mir-196a-1	MI0000552	miRBase_LINK	1	4	166	10	0	115	296	0.100	#DIV/0!	1.443
mmu-mir-495	MI0004639	miRBase_LINK	0	8	220	9	8	114	359	0.000	1.000	1.930
mmu-mir-377	MI0000794	miRBase_LINK	11	2	271	11	3	103	401	1.000	0.667	2.631
mmu-mir-7a-2	MI0000729	miRBase_LINK	3	335	171	5	328	99	941	0.600	1.021	1.727
mmu-mir-496	MI0004589	miRBase_LINK	2	1	262	11	6	97	379	0.182	0.167	2.701
mmu-mir-19a	MI0000688	miRBase_LINK	151	444	149	147	382	93	1,366	1.027	1.162	1.602
mmu-mir-874	MI0005479	miRBase_LINK	46	243	85	46	114	90	624	1.000	2.132	0.944
mmu-mir-669a-3	MI0004668	miRBase_LINK	15	23	157	48	6	82	331	0.313	3.833	1.915
mmu-mir-1-1			30	3	77	38	6	81	235	0.789	0.500	0.951
mmu-mir-1-2			25	3	75	32	6	81	222	0.781	0.500	0.926
mmu-mir-466e	MI0005506	miRBase_LINK	16	16	142	16	18	78	286	1.000	0.889	1.821
mmu-mir-487b	MI0003534	miRBase_LINK	15	1	208	26	0	77	327	0.577	#DIV/0!	2.701
mmu-mir-433	MI0001525	miRBase_LINK	21	0	191	45	2	76	335	0.467	0.000	2.513
mmu-mir-667	MI0004196	miRBase_LINK	18	1	123	51	1	76	270	0.353	1.000	1.618
mmu-mir-380	MI0000797	miRBase_LINK	1	6	207	5	6	68	293	0.200	1.000	3.044
mmu-mir-494	MI0003532	miRBase_LINK	4	3	252	29	5	67	360	0.138	0.600	3.761
mmu-mir-122	MI0000256	miRBase_LINK	723	392,484	409	494	367,471	65	761,646	1.464	1.068	6.292
mmu-mir-1191	MI0006296	miRBase_LINK	8	54	77	3	50	64	256	2.667	1.080	1.203
mmu-mir-296	MI0000394	miRBase_LINK	287	10	454	387	20	63	1,221	0.742	0.500	7.206
mmu-mir-666	MI0004553	miRBase_LINK	2	1	108	6	0	62	179	0.333	#DIV/0!	1.742
mmu-mir-219-1	MI0000702	miRBase_LINK	77	119	62	76	112	62	508	1.013	1.063	1.000
mmu-mir-344-1	MI0000630	miRBase_LINK	3	2	45	19	1	62	132	0.158	2.000	0.726
mmu-mir-344-2	MI0005495	miRBase_LINK	3	2	45	19	1	62	132	0.158	2.000	0.726
mmu-mir-505	MI0004706	miRBase_LINK	28	79	112	27	58	59	363	1.037	1.362	1.898
mmu-mir-467b	MI0004671	miRBase_LINK	1	16	104	1	17	58	197	1.000	0.941	1.793
mmu-mir-190b	MI0005478	miRBase_LINK	8	37	48	14	49	58	214	0.571	0.755	0.828
mmu-mir-184	MI0000226	miRBase_LINK	20	15	98	73	50	57	313	0.274	0.300	1.719
mmu-mir-669c	MI0004673	miRBase_LINK	1	18	91	10	7	57	184	0.100	2.571	1.596
mmu-mir-467e	MI0006128	miRBase_LINK	3	9	72	2	14	56	156	1.500	0.643	1.286
mmu-mir-31	MI0000579	miRBase_LINK	297	8,935	189	382	11,471	55	21,329	0.777	0.779	3.436
mmu-mir-183	MI0000225	miRBase_LINK	128	655	75	334	426	55	1,673	0.383	1.538	1.364
mmu-mir-363	MI0000765	miRBase_LINK	21	5	110	71	3	52	262	0.296	1.667	2.115
mmu-mir-141	MI0000166	miRBase_LINK	273	404	88	370	272	42	1,449	0.738	1.485	2.095
mmu-mir-543	MI0003519	miRBase_LINK	0	3	102	21	3	41	170	0.000	1.000	2.488
mmu-mir-466b-2	MI0005503	miRBase_LINK	8	9	72	8	9	39	145	1.000	1.000	1.846
mmu-mir-466a	MI0002401	miRBase_LINK	8	8	71	7	10	38	142	1.143	0.800	1.868
mmu-mir-449a	MI0001649	miRBase_LINK	16	2	45	9	2	35	109	1.778	1.000	1.286
mmu-mir-205	MI0000248	miRBase_LINK	2,127	108	71	6,337	43	32	8,718	0.336	2.512	2.219
mmu-mir-702	MI0004686	miRBase_LINK	10	10	38	19	2	30	109	0.526	5.000	1.267
mmu-mir-504	MI0005515	miRBase_LINK	5	0	1,177	5	0	29	1,216	1.000	#DIV/0!	40.586
mmu-mir-147	MI0005482	miRBase_LINK	4	4	96	7	6	29	146	0.571	0.667	3.310
mmu-mir-679	MI0004638	miRBase_LINK	3	1	90	7	1	29	131	0.429	1.000	3.103
mmu-mir-466b-3	MI0005504	miRBase_LINK	7	6	54	8	6	27	108	0.875	1.000	2.000
mmu-mir-692-2	MI0004661	miRBase_LINK	10	328	66	56	254	26	740	0.179	1.291	2.538
mmu-mir-467c	MI0005512	miRBase_LINK	1	5	43	0	4	26	79	#DIV/0!	1.250	1.654
mmu-mir-665	MI0004171	miRBase_LINK	1	0	42	10	0	25	78	0.100	#DIV/0!	1.680
mmu-mir-483	MI0003484	miRBase_LINK	6	1	5	21	0	24	57	0.286	#DIV/0!	0.208
mmu-mir-499	MI0004676	miRBase_LINK	56	9	82	109	5	23	284	0.514	1.800	3.565
mmu-mir-452	MI0001734	miRBase_LINK	0	3	36	6	4	22	71	0.000	0.750	1.636
mmu-mir-370	MI0001165	miRBase_LINK	7	0	53	6	0	20	86	1.167	#DIV/0!	2.650
mmu-mir-672	MI0004258	miRBase_LINK	25	2	29	48	3	20	127	0.521	0.667	1.450
mmu-mir-669b	MI0004666	miRBase_LINK	0	6	29	0	0	20	55	#DIV/0!	#DIV/0!	1.450
mmu-mir-20b	MI0003536	miRBase_LINK	5	6	57	10	6	19	103	0.500	1.000	3.000
mmu-mir-323	MI0000592	miRBase_LINK	1	1	39	14	0	18	73	0.071	#DIV/0!	2.167
mmu-mir-297a-4	MI0005489	miRBase_LINK	0	8	36	10	1	17	72	0.000	8.000	2.118
mmu-mir-297a-3	MI0005488	miRBase_LINK	0	7	36	10	1	17	71	0.000	7.000	2.118
mmu-mir-301b	MI0004122	miRBase_LINK	80	7	91	107	4	16	305	0.748	1.750	5.688
mmu-mir-701	MI0004685	miRBase_LINK	0	0	15	0	2	16	33	#DIV/0!	0.000	0.938
mmu-mir-217	MI0000731	miRBase_LINK	5	6	7	8	0	15	41	0.625	#DIV/0!	0.467
mmu-mir-466h	MI0005511	miRBase_LINK	1	3	16	8	3	14	45	0.125	1.000	1.143
mmu-mir-682	MI0004644	miRBase_LINK	26	76	14	33	102	14	265	0.788	0.745	1.000
mmu-mir-297b	MI0004674	miRBase_LINK	1	9	30	0	0	13	53	#DIV/0!	#DIV/0!	2.308
mmu-mir-466d	MI0005546	miRBase_LINK	2	4	28	11	5	13	63	0.182	0.800	2.154
mmu-mir-129-2	MI0000585	miRBase_LINK	26	17	19	32	23	13	130	0.813	0.739	1.462
mmu-mir-758	MI0004129	miRBase_LINK	0	0	16	0	0	13	29	#DIV/0!	#DIV/0!	1.231
mmu-mir-297c	MI0005492	miRBase_LINK	0	7	26	0	0	12	45	#DIV/0!	#DIV/0!	2.167
mmu-mir-449c	MI0004645	miRBase_LINK	1	0	25	0	0	11	37	#DIV/0!	#DIV/0!	2.273

mmu-mir-200c	MI0000694	miRBase_LINK	72	54	22	119	40	11	318	0.605	1.350	2.000
mmu-mir-491	MI0004680	miRBase_LINK	0	5	13	0	5	10	33	#DIV/0!	1.000	1.300
mmu-mir-493	MI0005514	miRBase_LINK	0	1	11	8	1	9	30	0.000	1.000	1.222
mmu-mir-384	MI0001146	miRBase_LINK	3	1	3	0	0	9	16	#DIV/0!	#DIV/0!	0.333
mmu-mir-297a-6	MI0005491	miRBase_LINK	0	0	18	20	2	8	48	0.000	0.000	2.250
mmu-mir-669f	MI0006287	miRBase_LINK	0	4	12	0	2	8	26	#DIV/0!	2.000	1.500
mmu-mir-490	MI0005002	miRBase_LINK	99	6	8	194	16	8	331	0.510	0.375	1.000
mmu-mir-216a	MI0000699	miRBase_LINK	14	11	6	9	1	8	49	1.556	11.000	0.750
mmu-mir-1199	MI0006307	miRBase_LINK	8	8	22	9	4	7	58	0.889	2.000	3.143
mmu-mir-106a	MI0000406	miRBase_LINK	3	2	16	3	1	7	32	1.000	2.000	2.286
mmu-mir-539	MI0003520	miRBase_LINK	2	0	14	0	0	7	23	#DIV/0!	#DIV/0!	2.000
mmu-mir-297a-5	MI0005490	miRBase_LINK	0	0	12	10	1	7	30	0.000	0.000	1.714
mmu-mir-129-1	MI0000222	miRBase_LINK	22	42	10	23	47	7	151	0.957	0.894	1.429
mmu-mir-694	MI0004664	miRBase_LINK	0	5	8	6	4	7	30	0.000	1.250	1.143
mmu-mir-715			24	7	6	43	3	7	90	0.558	2.333	0.857
mmu-mir-138-1	MI0000722	miRBase_LINK	7	0	6	23	1	7	44	0.304	0.000	0.857
mmu-mir-592	MI0004127	miRBase_LINK	27	66	25	24	348	6	496	1.125	0.190	4.167
mmu-mir-375	MI0000792	miRBase_LINK	8,270	137	12	4,163	133	6	12,721	1.987	1.030	2.000
mmu-mir-879	MI0005472	miRBase_LINK	0	56	9	3	34	6	108	0.000	1.647	1.500
mmu-mir-466f-2	MI0005508	miRBase_LINK	2	0	9	4	0	6	21	0.500	#DIV/0!	1.500
mmu-mir-135b	MI0000646	miRBase_LINK	0	0	4	0	0	6	10	#DIV/0!	#DIV/0!	0.667
mmu-mir-802	MI0004249	miRBase_LINK	167	33,519	25	64	7,020	5	40,800	2.609	4.775	5.000
mmu-mir-692-1	MI0004660	miRBase_LINK	3	76	16	7	63	5	170	0.429	1.206	3.200
mmu-mir-1197	MI0006305	miRBase_LINK	0	0	7	0	0	5	12	#DIV/0!	#DIV/0!	1.400
mmu-mir-219-2	MI0000741	miRBase_LINK	5	21	2	1	13	5	47	5.000	1.615	0.400
mmu-mir-1193	MI0006298	miRBase_LINK	0	0	26	0	1	4	31	#DIV/0!	0.000	6.500
mmu-mir-297a-1	MI0000395	miRBase_LINK	0	0	9	10	1	4	24	0.000	0.000	2.250
mmu-mir-297a-2	MI0000397	miRBase_LINK	0	0	9	10	1	4	24	0.000	0.000	2.250
mmu-mir-201	MI0000244	miRBase_LINK	4	58	7	3	52	4	128	1.333	1.115	1.750
mmu-mir-466f-3	MI0005509	miRBase_LINK	2	0	6	0	1	4	13	#DIV/0!	0.000	1.500
mmu-mir-135a-2	MI0000715	miRBase_LINK	3	0	1	3	0	4	11	1.000	#DIV/0!	0.250
mmu-mir-135a-1	MI0000161	miRBase_LINK	1	0	1	2	0	4	8	0.500	#DIV/0!	0.250
mmu-mir-713	MI0004698	miRBase_LINK	0	0	0	0	0	4	4	#DIV/0!	#DIV/0!	0.000
mmu-mir-551b	MI0004131	miRBase_LINK	3	116	8	1	27	3	158	3.000	4.296	2.667
mmu-mir-1-2-as			0	2	5	0	2	3	12	#DIV/0!	1.000	1.667
mmu-mir-466f-1	MI0005507	miRBase_LINK	2	0	4	0	0	3	9	#DIV/0!	#DIV/0!	1.333
mmu-mir-670	MI0004295	miRBase_LINK	0	0	1	0	0	3	4	#DIV/0!	#DIV/0!	0.333
mmu-mir-1192	MI0006297	miRBase_LINK	0	9	0	0	5	3	17	#DIV/0!	1.800	0.000
mmu-mir-669d	MI0006281	miRBase_LINK	0	0	12	0	0	2	14	#DIV/0!	#DIV/0!	6.000
mmu-mir-684-1	MI0004647	miRBase_LINK	1	3	6	0	10	2	22	#DIV/0!	0.300	3.000
mmu-mir-684-2	MI0004648	miRBase_LINK	1	3	6	0	10	2	22	#DIV/0!	0.300	3.000
mmu-mir-325	MI0000597	miRBase_LINK	0	0	3	0	0	2	5	#DIV/0!	#DIV/0!	1.500
mmu-mir-653	MI0005557	miRBase_LINK	4	0	2	6	1	2	15	0.667	0.000	1.000
mmu-mir-466f-4	MI0006291	miRBase_LINK	2	1	2	0	0	2	7	#DIV/0!	#DIV/0!	1.000
mmu-mir-7b	MI0000730	miRBase_LINK	0	0	2	0	0	2	4	#DIV/0!	#DIV/0!	1.000
mmu-mir-208b	MI0005552	miRBase_LINK	11	0	1	35	1	2	50	0.314	0.000	0.500
mmu-mir-703	MI0004687	miRBase_LINK	1	112	13	4	170	1	301	0.250	0.659	13.000
mmu-mir-211	MI0000708	miRBase_LINK	17	18	7	23	9	1	75	0.739	2.000	7.000
mmu-mir-18b	MI0005483	miRBase_LINK	4	3	7	7	0	1	22	0.571	#DIV/0!	7.000
mmu-mir-412	MI0001164	miRBase_LINK	0	0	4	0	0	1	5	#DIV/0!	#DIV/0!	4.000
mmu-mir-704	MI0004688	miRBase_LINK	1	2	3	2	8	1	17	0.500	0.250	3.000
mmu-mir-669e	MI0006300	miRBase_LINK	0	0	3	0	0	1	4	#DIV/0!	#DIV/0!	3.000
mmu-mir-547	MI0003523	miRBase_LINK	0	1	2	0	10	1	14	#DIV/0!	0.100	2.000
mmu-mir-669h	MI0006289	miRBase_LINK	0	0	2	0	0	1	3	#DIV/0!	#DIV/0!	2.000
mmu-mir-137	MI0000163	miRBase_LINK	0	273	1	0	179	1	454	#DIV/0!	1.525	1.000
mmu-mir-153	MI0000175	miRBase_LINK	6	1	1	0	4	1	13	#DIV/0!	0.250	1.000
mmu-mir-292	MI0000390	miRBase_LINK	0	6	1	0	0	1	8	#DIV/0!	#DIV/0!	1.000
mmu-mir-686	MI0004650	miRBase_LINK	0	2	1	0	1	1	5	#DIV/0!	2.000	1.000
mmu-mir-693	MI0004662	miRBase_LINK	1	1	1	0	0	1	4	#DIV/0!	#DIV/0!	1.000
mmu-mir-124-1	MI0000716	miRBase_LINK	0	0	1	2	0	1	4	0.000	#DIV/0!	1.000
mmu-mir-124-2	MI0000717	miRBase_LINK	0	0	1	2	0	1	4	0.000	#DIV/0!	1.000
mmu-mir-124-3	MI0000150	miRBase_LINK	0	0	1	2	0	1	4	0.000	#DIV/0!	1.000
mmu-mir-873	MI0005550	miRBase_LINK	0	1	1	0	0	1	3	#DIV/0!	#DIV/0!	1.000
mmu-mir-871	MI0005471	miRBase_LINK	9	70	0	5	99	1	184	1.800	0.707	0.000
mmu-mir-216b	MI0004126	miRBase_LINK	37	10	0	8	3	1	59	4.625	3.333	0.000
mmu-mir-206	MI0000249	miRBase_LINK	0	1	0	10	0	1	12	0.000	#DIV/0!	0.000
mmu-mir-720	MI0004678	miRBase_LINK	5	2	0	0	0	1	8	#DIV/0!	#DIV/0!	0.000
mmu-mir-207	MI0000250	miRBase_LINK	0	0	0	0	0	1	1	#DIV/0!	#DIV/0!	0.000

Supplementary Table 2.

A. Baseline clinical parameters.

Clinical parameters	T2D (n=65)	NFG (n=45)	*p*-value
miR342-3p	2.1 ± 3.4	2.2 ± 2.2	0.815
Log miR342-3p	0.7 ± 1.0	0.8 ± 0.68	0.875
Age (year-old)	65.8 ± 9.9	46.0 ± 8.2	4.15×10^{-13}**
Male (%)	63.1	46.7	
Height (cm)	162.4 ± 8.0	163.6 ± 10.2	0.831
Body weight (kg)	65.3 ± 11.6	64.6 ± 15.7	0.818
Body mass index (kg/m^2)	24.7 ± 3.7	23.9 ± 4.3	0.435
HbA1c (%)	7.4 ± 1.0	5.7 ± 0.3	9.63×10^{-19}**
Serum creatinine (mg/dL)	0.9 ± 0.4	0.7 ± 0.1	0.007*
Uric acid (mg/dl)	5.1 ± 1.2	5.2 ± 1.3	0.861
Total cholesterol (mg/dl)	190.0 ± 44.1	207.4 ± 28.5	0.006**
HDL cholesterol (mg/dl)	61.8 ± 18.5	78.1 ± 23.6	5.40×10^{-5}**
Triglyceride (mg/dl)	130.1 ± 59.8	86.8 ± 53.6	0.001**
LDL cholesterol (mg/dl)	109.4 ± 30.6	123.1 ± 28.0	0.013*
Hypertension (%)	52.3	-	
OHA/Insulin (%)	63/37	-	
Medications (SU/TZD/BG/αGI/GLP1, %)	26/17/34/35/6	-	

B. miR-342-3p level in T2D patients with or without medications.

	miR342-3p		
	T2D with medication	T2D without medication	*p*-value
SU	2.1 ± 2.0	2.2 ± 3.6	0.842
TZD	2.2 ± 2.3	2.2 ± 3.5	0.967
BG	2.2 ± 2.0	2.2 ± 3.8	0.985
αGI	2.4 ± 1.9	2.1 ± 3.8	0.791
GLP1	2.1 ± 3.9	2.2 ± 3.3	0.930

Abbreviations

T2D, type 2 diabetes; NFG, normal fasting glucose; OHA, oral hypoglycemic agents; Insulin, insulin therapy including basal-supported oral therapy; SU, sulfonylureas; TZD, thiazolidinediones; BG, biguanide (Metformin); αGI, alpha-glucosidase inhibitors; GLP1, glucagon-like peptide 1 receptor agonists. Hypertension was defined as blood pressure ≥ 140/90 mmHg or any antihypertensive drug treatment. *p*-value was determined by independent *t*-test (*$p < 0.05$; **$p < 0.01$).

Supplementary Table 3. The comparison of global mRNA expression in epididymal adipose tissues of *Mir342* (-/-) and *Mir342* (+/+) mice fed with standard (STD) chow.

	Sample name	Negative control (average)
Test	STD *Mir342* (-/-) 668	46.24
Control	STD *Mir342* (+/+) 669	50.72

2Up	Ratio ≧ 2 and Expression value of test sample ≧ Negative control (average)
2Down	Ratio ≦ 0.5 and Expression value of test sample ≧ Negative control (average)

Feature ID	Normalized data				Flag			Annotation																	Keyword
Probe Set ID	Expression v	Expression v	Ratio	Log2Ratio	Flag	category	Ratio flag	Gene Acces	Gene Symb	Gene Descr	mRNA Acce	mRNA Sou	mRna - Des	mRNA - xhy	EntrezGene	UniGene ID	SwissProt a	Chromosom	GO Biologic	GO Biologic	GO Cellular	GO Cellular	GO Molecul	GO Molecul	miR342 target gene
17248747	20.12932	11.61673	1.73278711	0.79309442	0	main		NM_173384	Sox30	SRY (sex de	NM_173384	RefSeq	Mus muscul	FALSE	214105	Mm.66708	Q8CGW4	chr11	GO:000635	transcription	GO:000563	nucleus // n	GO:000367	DNA binding	○
17385840	42.12201	27.06227	1.55648473	0.63829142	0	main		NM_001267	Fign	fidgetin	NM_001267	RefSeq	Mus muscul	FALSE	60344	Mm.130769	Q9ERZ6	chr2	GO:000704	cell cycle //	GO:000563	nucleus // c	GO:000016	nucleotide b	○
17338872	50.24272	32.30096	1.55545594	0.63733753	0	main		NM_027244	Ndufa11	NADH dehy	NM_027244	RefSeq	Mus muscul	FALSE	69875	Mm.279823	Q9D8B4	chr17	GO:000681	transport //	GO:000573	mitochondri	GO:000367	molecular_fu	○
17401036	15.54496	10.82743	1.43570173	0.52175606	0	main		NM_178777	Nhlh2	nescient hel	NM_178777	RefSeq	Mus muscul	FALSE	18072	Mm.137286	Q64221	chr3	GO:000635	transcription	GO:000563	nucleus // n	GO:000097	RNA polyme	○
17325671	26.69333	19.78366	1.34926146	0.43216994	0	main		NM_175548	Lsamp	limbic syster	NM_175548	RefSeq	Mus muscul	FALSE	268890	Mm.310524	Q8BLK3	chr16	GO:000715	cell adhesio	GO:000588	plasma men	---	---	○
17331918	36.80741	27.3346	1.34655016	0.42926797	0	main		NM_001145	Tiam1	T cell lymph	NM_001145	RefSeq	Mus muscul	FALSE	21844	Mm.310902	Q60610	chr16	GO:000330	cardiac mus	GO:000563	nucleus // c	GO:000505	receptor sig	○
17509315	35.72089	26.58824	1.34348456	0.42597974	0	main		NM_001145	Tenm3	teneurin trar	NM_001145	RefSeq	Mus muscul	FALSE	23965	Mm.42191	Q9WTS6	chr8	GO:000715	cell adhesio	GO:000588	integral com	GO:004280	protein hom	○
17428501	31.27386	23.34089	1.33987436	0.42209772	0	main		NM_001025	Dmbx1	diencephalo	NM_001025	RefSeq	Mus muscul	FALSE	140477	Mm.213206	Q91ZK4	chr4	GO:000012	negative reg	GO:000563	nucleus // tr	GO:000097	RNA polyme	○
17536501	50.00889	37.42121	1.33637822	0.41832837	0	main		NM_001177	Eda	ectodysplas	NM_001177	RefSeq	Mus muscul	FALSE	13607	Mm.328086	O54693	chrX	GO:000194	hair follicle c	GO:000557	extracellular	GO:000516	tumor necro	○
17544446	24.5099	18.3815	1.33340043	0.4151101	0	main		NM_183319	Xkrx	Kell blood gr	NM_183319	RefSeq	Mus muscul	FALSE	331524	Mm.235055	Q5GH68	chrX	GO:000815	biological_p	GO:001602	membrane	GO:000367	molecular_fu	○
17438903	34.9096	26.33271	1.32571239	0.40676782	0	main		NM_007423	Afp	alpha fetopr	NM_007423	RefSeq	Mus muscul	FALSE	11576	Mm.358570	P02772	chr5	GO:000154	ovulation fro	GO:000557	extracellular	GO:000456	beta-glucuro	○
17400521	28.69007	21.90077	1.3100028	0.38956989	0	main		NM_022030	Sv2a	synaptic ves	NM_022030	RefSeq	Mus muscul	FALSE	64051	Mm.491135	Q9JIS5	chr3	GO:000681	transport //	GO:000573	cytoplasm /	GO:000487	receptor act	○
17525198	32.71565	25.15817	1.30039864	0.37895395	0	main		NM_172290	Ntm	neurotrimin	NM_172290	RefSeq	Mus muscul	FALSE	235106	Mm.283138	Q99PJ0	chr9	GO:000715	cell adhesio	GO:000561	extracellular	---	---	○
17442507	378.3245	292.572	1.29309879	0.3708325	0	main		NM_001310	Setd8	SET domain	NM_001310	RefSeq	Mus muscul	FALSE	67956	Mm.137966	Q2YDW7	chr5	GO:000012	negative reg	GO:000563	nucleus // c	GO:000017	rRNA (aden	○
17359769	29.77205	23.11076	1.28823327	0.36539386	0	main		NM_011976	Sema4g	sema domai	NM_011976	RefSeq	Mus muscul	FALSE	26456	Mm.34404	Q9WUH7	chr19	GO:000175	neural crest	GO:000561	extracellular	GO:000551	protein bindi	○
17439741	324.3863	253.4995	1.2796329	0.35572999	0	main		NM_001080	Aff1	AF4/FMR2 f	NM_001080	RefSeq	Mus muscul	FALSE	17355	Mm.6949	O88573	chr5	GO:000635	transcription	GO:000563	nucleus // tr	GO:000370	transcription	○
17382496	56.70056	44.44615	1.27571364	0.35130453	0	main		NM_001177	Grin1	glutamate re	NM_001177	RefSeq	Mus muscul	FALSE	14810	Mm.278672	P35438	chr2	GO:000166	conditioned	GO:000573	cytoplasm /	GO:000487	receptor act	○
17520292	15.98714	12.60399	1.26841897	0.34303136	0	main		NM_009576	Zic4	zinc finger p	NM_009576	RefSeq	Mus muscul	FALSE	22774	Mm.8062 //	Q61467	chr9	---	---	GO:000563	nucleus // n	GO:000367	nucleic acid	○
17294187	34.91095	27.52689	1.26824897	0.34283799	0	main		NM_172053	Adamts16	a disintegrin	NM_172053	RefSeq	Mus muscul	FALSE	271127	Mm.115970	Q69Z28	chr13	GO:000165	branching in	GO:000557	extracellular	GO:000422	metalloendo	○
17392529	34.25529	27.02555	1.267515	0.34200282	0	main		NM_001077	Nkx2-2	NK2 homeo	NM_001077	RefSeq	Mus muscul	FALSE	18088	Mm.330639	P42586	chr2	GO:000332	type B panc	GO:000562	intracellular	GO:000098	RNA polyme	○
17455801	542.1521	431.3169	1.2569693	0.32994941	0	main		NM_007743	Col1a2	collagen, typ	NM_007743	RefSeq	Mus muscul	FALSE	12843	Mm.277792	Q01149	chr6	GO:000150	skeletal syst	GO:000557	extracellular	GO:000520	extracellular	○
17287066	537.094	427.7617	1.25559161	0.32836729	0	main		NM_001170	Rnf144b	ring finger p	NM_001170	RefSeq	Mus muscul	FALSE	218215	Mm.287609	Q8BKD6	chr13	GO:000020	protein poly	GO:000015	ubiquitin liga	GO:000484	ubiquitin-pro	○
17253215	40.44884	32.62972	1.23963184	0.30991172	0	main		NM_010484	Slc6a4	solute carrie	NM_010484	RefSeq	Mus muscul	FALSE	15567	Mm.300318	Q60857	chr11	GO:000681	transport //	GO:000576	endosome /	GO:000532	neurotransm	○
17490995	28.19445	22.82328	1.23533734	0.30490506	0	main		NM_008172	Grin2d	glutamate re	NM_008172	RefSeq	Mus muscul	FALSE	14814	Mm.322594	Q03391	chr7	GO:000196	startle respo	GO:000588	plasma men	GO:000487	receptor act	○
17453573	334.0362	270.5324	1.23473639	0.30420307	0	main		NM_146001	Hip1	huntingtin in	NM_146001	RefSeq	Mus muscul	FALSE	215114	Mm.280805	Q8VD75	chr5	GO:000635	transcription	GO:000563	nucleus // c	GO:000377	actin binding	○
17431910	50.59619	41.01265	1.23367278	0.30295979	0	main		NM_011039	Pax7	paired box 7	NM_011039	RefSeq	Mus muscul	FALSE	18509	Mm.218760	P47239	chr4	GO:000633	chromatin re	GO:000563	nucleus // tr	GO:000098	transcription	○
17321202	89.27699	72.38771	1.23331695	0.30254361	0	main		NM_173769	Zfp641	zinc finger p	NM_173769	RefSeq	Mus muscul	FALSE	239652	Mm.207453	Q8BZ34	chr15	GO:000635	transcription	GO:000562	intracellular	GO:000098	RNA polyme	○
17367216	700.7217	570.6381	1.22796165	0.2962655	0	main		NM_026162	Plxdc2	plexin doma	NM_026162	RefSeq	Mus muscul	FALSE	67448	Mm.313938	Q9DC11	chr2	---	---	GO:000588	plasma men	---	---	○
17394024	43.00315	35.08982	1.2255164	0.29338979	0	main		XM_006500	Oser1	oxidative str	XM_006500	RefSeq	PREDICTED	FALSE	66680	Mm.25119	Q9D722	chr2	GO:007030	cellular resp	GO:000557	cellular_com	GO:000367	molecular_fu	○
17424880	22.42256	18.37914	1.2200005	0.28688174	0	main		XM_006537	Pax5	paired box 5	XM_006537	RefSeq	PREDICTED	FALSE	18507	Mm.439659	Q02650	chr4	GO:000012	negative reg	GO:000563	nucleus // tr	GO:000097	RNA polyme	○
17419305	20.38057	16.80467	1.21279204	0.27833219	0	main		NM_010769	Matn1	matrilin 1, ca	NM_010769	RefSeq	Mus muscul	FALSE	17180	Mm.271796	P51942	chr4	GO:000206	chondrocyte	GO:000557	extracellular	GO:000551	protein bindi	○
17494868	26.38733	21.79678	1.2106068	0.27573036	0	main		NM_001170	Trim66	tripartite mo	NM_001170	RefSeq	Mus muscul	FALSE	330627	Mm.183528	Q924W6	chr7	GO:004589	negative reg	GO:000562	intracellular	GO:000368	chromatin bi	○
17465332	14.73537	12.25005	1.20288244	0.26649565	0	main		NM_138682	Lrrc4	leucine rich	NM_138682	RefSeq	Mus muscul	FALSE	192198	Mm.443660	Q99PH1	chr6	GO:005080	regulation o	GO:000588	plasma men	GO:000551	protein bindi	○
17465272	10.61706	8.866076	1.19749255	0.26001669	0	main		NM_001311	Grm8	glutamate re	NM_001311	RefSeq	Mus muscul	FALSE	14823	Mm.320732	P47743	chr6	GO:000716	signal trans	GO:000588	plasma men	GO:000164	group III me	○
17545121	25.10733	21.15036	1.1870876	0.2474264	0	main		NM_001110	Dcx	doublecortin	NM_001110	RefSeq	Mus muscul	FALSE	13193	Mm.12871	O88809	chrX	GO:000176	neuron migr	GO:000573	cytoplasm /	GO:000468	calmodulin-	○
17441134	14.48534	12.20723	1.18661973	0.24685768	0	main		XM_006530	Msi1	musashi RN	XM_006530	RefSeq	PREDICTED	FALSE	17690	Mm.5077	Q61474	chr5	GO:000972	response to	GO:000563	nucleus // c	GO:000016	nucleotide b	○
17438246	430.4402	363.4018	1.1844746	0.24424726	0	main		NM_001083	Pdgfra	platelet deri	NM_001083	RefSeq	Mus muscul	FALSE	18595	Mm.221403	P26618	chr5	GO:000155	luteinization	GO:000563	nucleus // n	GO:000016	nucleotide b	○
17402787	23.25531	19.70089	1.18041926	0.23929937	0	main		NM_021382	Tacr3	tachykinin re	NM_021382	RefSeq	Mus muscul	FALSE	21338	Mm.103810	P47937	chr3	GO:000716	signal trans	GO:000563	nucleus // c	GO:000487	signal trans	○
17427164	41.02165	34.92921	1.1744225	0.23195151	0	main		NM_001177	Elavl2	ELAV (embr	NM_001177	RefSeq	Mus muscul	FALSE	15569	Mm.318042	Q60899	chr4	---	---	---	---	GO:000016	nucleotide b	○
17224551	23.35521	19.93151	1.17177324	0.22869341	0	main		NM_008985	Ptprn	protein tyros	NM_008985	RefSeq	Mus muscul	FALSE	19275	Mm.2902	Q60673	chr1	GO:000030	response to	GO:000576	endosome /	GO:000472	protein tyros	○
17537643	34.45277	29.60971	1.16356324	0.21854962	0	main		NM_026239	Tmem35	transmembr	NM_026239	RefSeq	Mus muscul	FALSE	67564	Mm.41489	Q9D328	chrX	GO:000815	biological_p	GO:000577	peroxisome	GO:000367	molecular_fu	○
17456627	75.02679	64.621	1.16102799	0.21540276	0	main		NM_001081	Flnc	filamin C, ga	NM_001081	RefSeq	Mus muscul	FALSE	68794	Mm.39046	Q8VHX6	chr6	GO:003002	actin filamer	GO:000573	cytoplasm /	GO:000377	actin binding	○
17269921	18.21216	15.69282	1.16054094	0.21479741	0	main		NM_009764	Brca1	breast cance	NM_009764	RefSeq	Mus muscul	FALSE	12189	Mm.244975	P48754	chr11	GO:000072	double-strar	GO:000079	condensed	GO:000367	DNA binding	○
17542047	24.02346	20.70273	1.16040058	0.21462293	0	main		NM_178740	Slitrk4	SLIT and NT	NM_178740	RefSeq	Mus muscul	FALSE	245446	Mm.101707	Q810B8	chrX	GO:000740	axonogenes	GO:001602	membrane	GO:000367	molecular_fu	○
17336086	97.13081	84.18594	1.15376522	0.20634969	0	main		NM_172619	Adamts10	a disintegrin	NM_172619	RefSeq	Mus muscul	FALSE	224697	Mm.29304	P58459	chr17	GO:000650	proteolysis	GO:000152	microfibril //	GO:000422	metalloendo	○
17240670	8.819125	7.713967	1.14326714	0.19316255	0	main		NM_001031	Lin28b	lin-28 homol	NM_001031	RefSeq	Mus muscul	FALSE	380669	Mm.440328	Q45KJ6	chr10	GO:000635	regulation o	GO:000563	nucleus // n	GO:000367	nucleic acid	○
17396765	23.00042	20.128	1.14270767	0.19245638	0	main		NM_001013	Usp13	ubiquitin spe	NM_001013	RefSeq	Mus muscul	FALSE	72607	Mm.316153	Q5BKP2	chr3	GO:000635	regulation o	GO:000576	lysosome //	GO:000419	cysteine-typ	○
17219612	53.81225	47.09312	1.14267753	0.19241832	0	main		NM_177723	Vsig8	V-set and im	NM_177723	RefSeq	Mus muscul	FALSE	240916	Mm.71940	Q6P3A4	chr1	GO:000815	biological_p	GO:000562	intracellular	GO:004482	poly(A) RNA	○
17309001	32.20497	28.25089	1.13996302	0.18898703	0	main		NM_178685	Pcdh20	protocadher	NM_178685	RefSeq	Mus muscul	FALSE	219257	Mm.128861	Q8BIZ0	chr14	GO:000715	cell adhesio	GO:000588	plasma men	GO:000550	calcium ion	○
17484952	118.5918	104.0389	1.13987941	0.18888121	0	main		NM_001252	Tspan4	tetraspanin 4	NM_001252	RefSeq	Mus muscul	FALSE	64540	Mm.489619	Q9DCK3	chr7	GO:000646	protein com	GO:000588	plasma men	GO:000382	antigen bind	○
17492861	106.3442	93.37978	1.13883541	0.18755926	0	main		NM_013886	Hdgfrp3	hepatoma-d	NM_013886	RefSeq	Mus muscul	FALSE	29877	Mm.392934	Q9JMG7	chr7	GO:000828	cell prolifera	GO:000563	nucleus // n	GO:000368	chromatin bi	○
17495340	110.7166	97.46153	1.1360031	0.18396677	0	main		NM_001025	Sox6	SRY (sex de	NM_001025	RefSeq	Mus muscul	FALSE	20679	Mm.323365	P40645	chr7	GO:000012	negative reg	GO:000563	nucleus // n	GO:000367	DNA binding	○
17517780	117.8388	104.2109	1.13077231	0.17730846	0	main		NM_153799	Edc3	enhancer of	NM_153799	RefSeq	Mus muscul	FALSE	353190	Mm.268104	Q8K2D3	chr9	GO:009050	RNA phosph	GO:000573	cytoplasm /	GO:000372	RNA binding	○
17527735	200.2881	177.1278	1.13075474	0.17728605	0	main		NM_001042	Neo1	neogenin	NM_001042	RefSeq	Mus muscul	FALSE	18007	Mm.42249	P97798	chr9	GO:000635	regulation o	GO:000588	plasma men	GO:000487	receptor act	○
17414009	55.76183	49.41135	1.1285227	0.17443544	0	main		NM_001145	Msantd3	Myb/SANT-l	NM_001145	RefSeq	Mus muscul	FALSE	66665	Mm.140749	Q9CR78	chr4	GO:000815	biological_p	GO:000557	cellular_com	GO:004280	identical pro	○
17531154	33.20391	29.54562	1.12381835	0.16840887	0	main		NM_007567	Bsn	bassoon	NM_007567	RefSeq	Mus muscul	FALSE	12217	Mm.20425	O88737	chr9	GO:000741	synapse ass	GO:000573	cytoplasm /	GO:000551	protein bindi	○
17360260	44.50254	39.65516	1.12223832	0.16637908	0	main		NM_025696	Sorcs3	sortilin-relate	NM_025696	RefSeq	Mus muscul	FALSE	66673	Mm.70980	Q8VI51	chr19	GO:000761	learning // r	GO:001602	membrane	GO:000551	protein bindi	○
17370487	55.9489	50.10674	1.11659429	0.15910509	0	main		NM_172854	Olfml2a	olfactomedir	NM_172854	RefSeq	Mus muscul	FALSE	241327	Mm.103491	Q8BHP7	chr2	GO:003019	extracellular	GO:000557	extracellular	GO:004280	protein hom	○
17442970	292.0267	262.8956	1.11080863	0.15161028	0	main		NM_001001	Vkorc1l1	vitamin K ep	NM_001001	RefSeq	Mus muscul	FALSE	69568	Mm.288718	Q6TEK5	chr5	GO:001718	peptidyl-glut	GO:000578	endoplasmic	GO:001649	oxidoreducta	○
17414913	9.904814	8.924139	1.10989015	0.1504169	0	main		NM_001163	Frmd3	FERM doma	NM_001163	RefSeq	Mus muscul	FALSE	242506	Mm.221597	Q8BHD4	chr4	GO:000815	biological_p	GO:000573	cytoplasm /	GO:000809	cytoskeletal	○
17381475	306.3583	276.614	1.10752999	0.14734576	0	main		NM_001110	Celf2	CUGBP, Ela	NM_001110	RefSeq	Mus muscul	FALSE	14007	Mm.398543	Q9Z0H4	chr2	GO:000637	mRNA splic	GO:000563	nucleus // n	GO:000016	nucleotide b	○
17483577	138.7015	126.2055	1.09901312	0.13620861	0	main		NM_001082	Itgam	integrin alph	NM_001082	RefSeq	Mus muscul	FALSE	16409	Mm.262106	P05555	chr7	GO:000252	leukocyte m	GO:000561	extracellular	GO:000184	opsonin bind	○
17461888	38.73437	35.33884	1.09608493	0.13235959	0	main		NM_001252	Hrh1	histamine re	NM_001252	RefSeq	Mus muscul	FALSE	15465	Mm.333327	P70174	chr6	GO:000695	inflammator	GO:000565	nucleoplasm	GO:000487	signal trans	○
17365106	63.95678	58.36703	1.09576896	0.13194365	0	main		NM_001033	Sec31b	Sec31 homo	NM_001033	RefSeq	Mus muscul	FALSE	240667	Mm.329873	Q3TZ89	chr19	GO:000681	transport //	GO:000573	cytoplasm /	GO:000367	molecular_fu	○
17282570	68.45801	62.56913	1.09411798	0.12976831	0	main		NM_013589	Ltbp2	latent transfo	NM_013589	RefSeq	Mus muscul	FALSE	16997	Mm.3900	O08999	chr12	GO:004320	extracellular	GO:000557	extracellular	GO:000550	calcium ion	○
17424884	20.86971	19.1464	1.090007	0.1243374	0	main		NM_008782	Pax5	paired box 5	NM_008782	RefSeq	Mus muscul	FALSE	18507	Mm.439659	Q02650	chr4	GO:000012	negative reg	GO:000563	nucleus // tr	GO:000097	RNA polyme	○
17321094	49.44227	45.45639	1.0876858	0.12126187	0	main		NM_001113	Col2a1	collagen, typ	NM_001113	RefSeq	Mus muscul	FALSE	12824	Mm.2423	P28481	chr15	GO:000150	skeletal syst	GO:000557	extracellular	GO:000520	extracellular	○
17451536	103.1459	95.00095	1.08573546	0.11867263	0	main		NM_029956	Mmab	methylmalon	NM_029956	RefSeq	Mus muscul	FALSE	77697	Mm.105182	Q9D273	chr5	GO:000923	cobalamin m	GO:000573	mitochondri	GO:000016	nucleotide b	○
17397344	150.8627	139.1975	1.08380323	0.11610285	0	main		NM_001130	Jade1	jade family P	NM_001130	RefSeq	Mus muscul	FALSE	269424	Mm.286285	Q6ZPI0	chr3	GO:000635	transcription	GO:000012	histone acet	GO:000110	RNA polyme	○
17383858	95.34297	88.12531	1.08190224	0.11357014	0	main		NM_001301	Dnm1	dynamin 1	NM_001301	RefSeq	Mus muscul	FALSE	13429	Mm.44736	P39053	chr2	GO:000203	G-protein co	GO:000191	photorecept	GO:000016	nucleotide b	○
17500263	53.72115	49.6639	1.08169415	0.11329263	0	main		NM_199042	Thap1	THAP doma	NM_199042	RefSeq	Mus muscul	FALSE	73754	Mm.383241	Q8CHW1	chr8	GO:000193	endothelial	GO:000563	nucleus // n	GO:000367	nucleic acid	○
17410130	74.96661	69.52476	1.07827211	0.10872131	0	main		NM_001034	Ank2	ankyrin 2, br	NM_001034	RefSeq	Mus muscul	FALSE	109676	Mm.220242	Q8C8R3	chr3	GO:000202	regulation o	GO:000562	intracellular	GO:000551	protein bindi	○
17452311	46.23802	42.98427	1.0756963	0.10527081	0	main		NM_001312	Cux2	cut-like hom	NM_001312	RefSeq	Mus muscul	FALSE	13048	Mm.332661	P70298	chr5	GO:000012	negative reg	GO:000563	nucleus // n	GO:000097	RNA polyme	○
17529481	44.14587	41.07187	1.07484441	0.10412784	0	main		NM_023814	Tbx18	T-box18	NM_023814	RefSeq	Mus muscul	FALSE	76365	Mm.158789	Q9EPZ6	chr9	GO:000012	negative reg	GO:000563	nucleus // T	GO:000097	transcription	○
17395985	110.5083	102.8577	1.07438043	0.10350493	0	main		NM_030708	Zfhx4	zinc finger h	NM_030708	RefSeq	Mus muscul	FALSE	80892	Mm.41522	Q9JJN2	chr3	GO:000635	transcription	GO:000563	nucleus // n	GO:000367	nucleic acid	○
17416540	48.49289	45.24218	1.07185131	0.10010479	0	main		NM_001080	Lrp8	low density l	NM_001080	RefSeq	Mus muscul	FALSE	16975	Mm.442134	Q924X6	chr4	GO:000012	negative reg	GO:000557	extracellular	GO:000194	glycoprotein	○
17465296	9.196845	8.636144	1.06492492	0.09075173	0	main		NR_030420	Mir592	microRNA 5	NR_030420	RefSeq	Mus muscul	FALSE	735266	---	P47743	chr6	---	---	---	---	---	---	○
17415943	83.40235	78.60956	1.06096956	0.08538326	0	main		NM_001164	Dnajc6	DnaJ (Hsp4	NM_001164	RefSeq	Mus muscul	FALSE	72685	Mm.76494	Q80TZ3	chr4	GO:007231	clathrin coat	GO:004520	synapse // r	GO:000472	phosphopro	○
17266452	27.85989	26.29961	1.05932712	0.08314816	0	main		NM_001168	Sarm1	sterile alpha	NM_001168	RefSeq	Mus muscul	FALSE	237868	Mm.210332	Q6PDS3	chr11	GO:000237	immune sys	GO:000573	cytoplasm /	GO:000551	protein bindi	○
17269695	167.9479	158.7477	1.05795486	0.08127807	0	main		NM_001113	Stat5b	signal transc	NM_001113	RefSeq	Mus muscul	FALSE	20851	Mm.34064	P42232	chr11	GO:000025	allantoin me	GO:000563	nucleus // n	GO:000097	RNA polyme	○
17372066	22.19318	21.01071	1.05627939	0.07899149	0	main		NM_013555	Hoxd9	homeobox D	NM_013555	RefSeq	Mus muscul	FALSE	15438	Mm.26544	P28357	chr2	GO:000012	negative reg	GO:000563	nucleus // n	GO:000097	RNA polyme	○
17518616	116.5696	110.4689	1.0552255	0.07755133	0	main		NM_028030	Rbpms2	RNA binding	NM_028030	RefSeq	Mus muscul	FALSE	71973	Mm.29148	Q8VC52	chr9	GO:000815	biological_p	GO:000557	cellular_com	GO:000016	nucleotide b	○
17531939	68.77133	65.39586	1.05161596	0.07260794	0	main		NM_175380	Gpd1l	glycerol-3-pl	NM_175380	RefSeq	Mus muscul	FALSE	333433	Mm.38198	Q3ULJ0	chr9	GO:000202	regulation o	GO:000573	cytoplasm /	GO:000436	glycerol-3-pl	○
17441140	30.27419	28.80253	1.05109482	0.07189282	0	main		NM_008629	Msi1	musashi RN	NM_008629	RefSeq	Mus muscul	FALSE	17690	Mm.5077	Q61474	chr5	GO:000972	response to	GO:000563	nucleus // c	GO:000016	nucleotide b	○
17400072	36.69894	35.14649	1.04417084	0.06235778	0	main		NM_028307	Tdrkh	tudor and KI	NM_028307	RefSeq	Mus muscul	FALSE	72634	Mm.331198	Q80VL1	chr3	GO:000714	male meiosi	GO:000573	cytoplasm /	GO:000367	nucleic acid	○
17323659	104.351	99.98786	1.0436367	0.06161958	0	main		NM_001080	Trmt2a	TRM2 tRNA	NM_001080	RefSeq	Mus muscul	FALSE	15547	Mm.271798	Q8BNV1	chr16	GO:000639	RNA proces	---	---	GO:000016	nucleotide b	○
17328792	164.4592	157.7459	1.04255768	0.06012721	0	main		NM_001109	Dgcr2	DiGeorge sy	NM_001109	RefSeq	Mus muscul	FALSE	13356	Mm.254515	P98154	chr16	GO:000715	cell adhesio	GO:001602	membrane	GO:003024	carbohydrat	○
17294267	26.40076	25.37928	1.04024858	0.05692832	0	main		NM_028878	Slc6a19	solute carrie	NM_028878	RefSeq	Mus muscul	FALSE	74338	Mm.271635	Q9D687	chr13	GO:000681	transport //	GO:000588	plasma men	GO:000532	neurotransm	○
17223985	52.83086	51.07743	1.03432886	0.04869496	0	main		NM_011770	Ikzf2	IKAROS fan	NM_011770	RefSeq	Mus muscul	FALSE	22779	Mm.106343	P81183	chr1	GO:000635	transcription	GO:000563	nucleus // n	GO:000367	nucleic acid	○
17246577	70.80473	68.46188	1.03422123	0.04854483	0	main		NM_178149	Pik3ip1	phosphoinos	NM_178149	RefSeq	Mus muscul	FALSE	216505	Mm.390323	Q7TMJ8	chr11	GO:001406	negative reg	GO:000588	plasma men	GO:003631	phosphatidy	○
17392578	35.80934	34.67097	1.03283352	0.04660773	0	main		NM_019632	Napb	N-ethylmale	NM_019632	RefSeq	Mus muscul	FALSE	17957	Mm.274308	P28663	chr2	GO:000681	transport //	GO:000577	vacuolar me	GO:000548	soluble NSF	○
17226676	114.7111	111.1392	1.03213898	0.04563724	0	main		NM_001162	Pfkfb2	6-phosphofr	NM_001162	RefSeq	Mus muscul	FALSE	18640	Mm.249861	P70265	chr1	GO:000600	glucose cata	GO:000573	cytoplasm	GO:000387	6-phosphofr	○
17423701	15.61336	15.14345	1.03103058	0.04408712	0	main		NM_010243	Fut9	fucosyltrans	NM_010243	RefSeq	Mus muscul	FALSE	14348	Mm.39101	O88819	chr4	GO:000648	protein glyc	GO:000579	Golgi appar	GO:000000	alpha-1,6-m	○
17384951	260.0061	252.7078	1.02888039	0.04107528	0	main		NM_001289	Zeb2	zinc finger E	NM_001289	RefSeq	Mus muscul	FALSE	24136	Mm.440702	Q9R0G7	chr2	GO:000012	negative reg	GO:000563	nucleus // n	GO:000120	transcription	○
17547634	449.3186	441.4713	1.01777533	0.02541913	0	main		NM_008379	Kpnb1	karyopherin	NM_008379	RefSeq	Mus muscul	FALSE	16211	Mm.251013	P70168	chr11	GO:000005	protein impo	GO:000563	nucleus // n	GO:000551	protein bindi	○
17430775	8.289083	8.16085	1.01571319	0.02249308	0	main		NR_036572	Rps15a-ps4	ribosomal pr	NR_036572	RefSeq	Mus muscul	FALSE	664903	Mm.490762	Q9JL60	chr4	---	---	---	---	---	---	○
17448693	18.46791	18.25478	1.0116753	0.01674632	0	main		NM_001122	Txk	TXK tyrosine	NM_001122	RefSeq	Mus muscul	FALSE	22165	Mm.3264	P42682	chr5	GO:000181	cytokine pro	GO:000563	nucleus // c	GO:000016	nucleotide b	○
17276642	301.7333	299.0916	1.00883241	0.01268653	0	main		NM_001252	Fut8	fucosyltrans	NM_001252	RefSeq	Mus muscul	FALSE	53618	Mm.35628	Q9WTS2	chr12	GO:000648	protein N-lin	GO:000573	cytoplasm /	GO:000842	glycoprotein	○
17261470	179.0967	177.5807	1.00853696	0.01226396	0	main		NM_001291	Rhbdf1	rhomboid fa	NM_001291	RefSeq	Mus muscul	FALSE	13650	Mm.11545	Q6PIX5	chr11	GO:000650	proteolysis	GO:000013	Golgi memb	GO:000425	serine-type	○
17449526	508.7817	504.9713	1.00754578	0.01084539	0	main		NM_030886	Ankrd17	ankyrin repe	NM_030886	RefSeq	Mus muscul	FALSE	81702	Mm.245522	Q99NH0	chr5	GO:000195	blood vesse	GO:000078	chromatin /	GO:000367	nucleic acid	○
17290111	74.83317	74.27445	1.00752237	0.01081187	0	main		NM_008002	Fgf10	fibroblast gr	NM_008002	RefSeq	Mus muscul	FALSE	14165	Mm.317323	O35565	chr13	GO:000013	establishme	GO:000557	extracellular	GO:000510	receptor bin	○
17393160	61.79236	61.38258	1.00667584	0.00959919	0	main		NM_001291	E2f1	E2F transcri	NM_001291	RefSeq	Mus muscul	FALSE	13555	Mm.18036	Q61501	chr2	GO:000007	DNA damag	GO:000079	nuclear chro	GO:000104	core promot	○
17333222	52.77758	52.51259	1.00504622	0.00726185	0	main		NM_026644	Agpat4	1-acylglycer	NM_026644	RefSeq	Mus muscul	FALSE	68262	Mm.258300	Q8K4X7	chr17	GO:000662	lipid metabo	GO:001602	membrane	GO:000384	1-acylglycer	○
17429810	44.33787	44.11662	1.00501512	0.0072172	0	main		NM_023423	Akirin1	akirin 1	NM_023423	RefSeq	Mus muscul	FALSE	68050	Mm.486258	Q99LF1	chr4	GO:000815	biological_p	GO:000563	nucleus // n	GO:000367	molecular_fu	○
17427746	162.2631	161.5786	1.00423633	0.00609882	0	main		NM_178143	Prkaa2	protein kinas	NM_178143	RefSeq	Mus muscul	FALSE	108079	Mm.48638	Q8BRK8	chr4	GO:000635	transcription	GO:000563	nucleus // n	GO:000016	nucleotide b	○
17320693	43.75328	43.58567	1.00384553	0.00553729	0	main		NM_001033	Gxylt1	glucoside xy	NM_001033	RefSeq	Mus muscul	FALSE	223827	Mm.22819	Q3UHH8	chr15	GO:001626	O-glycan pr	GO:001602	membrane	GO:001674	transferase	○
17472089	31.72949	31.64303	1.0027323	0.00393658	0	main		NM_008171	Grin2b	glutamate re	NM_008171	RefSeq	Mus muscul	FALSE	14812	Mm.436649	Q01097	chr6	GO:000150	action poten	GO:000588	plasma men	GO:000487	receptor act	○
17409075	162.5052	162.8043	0.99816282	-0.0026529	0	main		NM_001113	Csf1	colony stimu	NM_001113	RefSeq	Mus muscul	FALSE	12977	Mm.795	P07141	chr3	GO:000150	ossification	GO:000557	extracellular	GO:000512	cytokine act	○
17343735	170.3582	171.1077	0.99561972	-0.0063333	0	main		NM_013543	H2-Ke6	H2-K region	NM_013543	RefSeq	Mus muscul	FALSE	14979	Mm.275452	P50171	chr17	GO:000662	lipid metabo	GO:000573	mitochondri	GO:000025	C-3 sterol d	○
17349465	147.9494	148.6045	0.99559165	-0.006374	0	main		NM_175104	Fam53c	family with s	NM_175104	RefSeq	Mus muscul	FALSE	66306	Mm.29424	Q8BXQ8	chr18	GO:000815	biological_p	GO:000557	cellular_com	GO:000367	molecular_fu	○
17528714	182.1496	183.0559	0.99504905	-0.0071604	0	main		NM_001253	Tcf12	transcription	NM_001253	RefSeq	Mus muscul	FALSE	21406	Mm.171615	Q61286	chr9	GO:000635	transcription	GO:000079	nuclear chro	GO:000097	RNA polyme	○
17496237	72.30498	72.6756	0.99490035	-0.0073761	0	main		NM_001081	Sh2b1	SH2B adapt	NM_001081	RefSeq	Mus muscul	FALSE	20399	Mm.8538	Q91ZM2	chr7	GO:000692	movement c	GO:000172	ruffle // nucl	GO:000487	signal trans	○
17482021	1906.224	1920.469	0.99258254	-0.010741	0	main		NM_011055	Pde3b	phosphodies	NM_011055	RefSeq	Mus muscul	FALSE	18576	Mm.430730	Q61409	chr7	GO:000152	angiogenesi	GO:000578	endoplasmic	GO:000411	3',5'-cyclic-n	○
17289372	386.2276	389.7773	0.990893	-0.0131988	0	main		NM_007930	Enc1	ectodermal-	NM_007930	RefSeq	Mus muscul	FALSE	13803	Mm.241073	O35709	chr13	GO:000727	multicellular	GO:000079	nuclear chro	GO:000377	actin binding	○
17339108	641.3323	648.3241	0.98921558	-0.0156431	0	main		NM_008549	Man2a1	mannosidas	NM_008549	RefSeq	Mus muscul	FALSE	17158	Mm.2433	P27046	chr17	GO:000170	in utero emb	GO:000013	Golgi memb	GO:000382	catalytic acti	○
17539888	51.19691	51.92461	0.98598545	-0.0203617	0	main		NM_018832	Magix	MAGI family	NM_018832	RefSeq	Mus muscul	FALSE	54634	Mm.67912	Q4KL35	chrX	GO:000815	biological_p	GO:000557	cellular_com	GO:000367	molecular_fu	○
17273700	71.17143	72.34348	0.98379882	-0.0235648	0	main		NM_153082	Dnajc27	DnaJ (Hsp4	NM_153082	RefSeq	Mus muscul	FALSE	217378	Mm.271656	Q8CFP6	chr12	GO:000688	intracellular	GO:000563	nucleus // n	GO:000016	nucleotide b	○
17491788	95.98657	97.62957	0.98317108	-0.0244856	0	main		NM_021366	Klf13	Kruppel-like	NM_021366	RefSeq	Mus muscul	FALSE	50794	Mm.240473	Q9JJZ6	chr7	GO:000635	transcription	GO:000563	nucleus // n	GO:000097	RNA polyme	○
17245334	170.1966	173.4036	0.98150557	-0.0269316	0	main		NM_001014	Dyrk2	dual-specific	NM_001014	RefSeq	Mus muscul	FALSE	69181	Mm.458174	Q5U4C9	chr10	GO:000646	protein phos	GO:000015	ubiquitin liga	GO:000016	nucleotide b	○
17415035	41.32409	42.14399	0.98054527	-0.0283439	0	main		NM_001081	Ccdc171	coiled-coil d	NM_001081	RefSeq	Mus muscul	FALSE	320226	Mm.180032	E9Q1U1	chr4	GO:000815	biological_p	GO:000557	cellular_com	GO:000367	molecular_fu	○
17247448	143.0667	145.9767	0.98006531	-0.0290502	0	main		NM_024459	Ppp3r1	protein phos	NM_024459	RefSeq	Mus muscul	FALSE	19058	Mm.41840	Q63810	chr11	GO:000156	patterning o	GO:000573	cytoplasm /	GO:000472	phosphopro	○
17530782	32.02332	32.76868	0.97725389	-0.0331947	0	main		NM_029103	Manf	mesenceph	NM_029103	RefSeq	Mus muscul	FALSE	74840	Mm.29778	Q9CXI5	chr9	GO:000201	vasoconstric	GO:000557	extracellular	GO:000808	growth facto	○
17223793	59.61285	61.05568	0.97636862	-0.0345022	0	main		NM_001042	Fzd5	frizzled hom	NM_001042	RefSeq	Mus muscul	FALSE	14367	Mm.150813	Q9EQD0	chr1	GO:000057	embryonic a	GO:000576	early endos	GO:000487	signal trans	○
17347281	218.0242	223.8336	0.9740459	-0.0379383	0	main		NM_011500	Strn	striatin, calm	NM_011500	RefSeq	Mus muscul	FALSE	268980	Mm.391038	O55106	chr17	GO:000762	locomotory	GO:000015	protein phos	GO:000551	calmodulin b	○
17219079	46.29466	47.64585	0.97164097	-0.0415048	0	main		NM_001160	Fam78b	family with s	NM_001160	RefSeq	Mus muscul	FALSE	226610	Mm.389217	Q8BQN5	chr1	GO:000815	biological_p	GO:000557	cellular_com	GO:000367	molecular_fu	○
17223412	149.1088	153.7217	0.96999187	-0.0439554	0	main		NM_001310	Fam126b	family with s	NM_001310	RefSeq	Mus muscul	FALSE	213056	Mm.26272	Q8C729	chr1	GO:000815	biological_p	GO:000562	intracellular	GO:000367	molecular_fu	○
17268469	282.2452	291.0156	0.96986278	-0.0441474	0	main		NM_008379	Kpnb1	karyopherin	NM_008379	RefSeq	Mus muscul	FALSE	16211	Mm.251013	P70168	chr11	GO:000005	protein impo	GO:000563	nucleus // n	GO:000551	protein bindi	○
17439481	338.4068	350.084	0.96664458	-0.0489426	0	main		NM_080708	Bmp2k	BMP2 induc	NM_080708	RefSeq	Mus muscul	FALSE	140780	Mm.281490	Q91Z96	chr5	GO:000646	protein phos	GO:000563	nucleus // n	GO:000016	nucleotide b	○
17261494	96.16077	100.2062	0.95962895	-0.0594514	0	main		NM_001284	Nprl3	nitrogen per	NM_001284	RefSeq	Mus muscul	FALSE	17168	---	Q8VIJ8	chr11	GO:000328	ventricular s	GO:199013	Iml1 comple	GO:000509	GTPase act	○
17286595	347.3542	362.054	0.95939887	-0.0597973	0	main		NM_001039	Rreb1	ras responsi	NM_001039	RefSeq	Mus muscul	FALSE	68750	Mm.491109	Q3UH06	chr13	GO:000012	negative reg	GO:000563	nucleus // n	GO:000097	RNA polyme	○
17270648	419.0868	437.1094	0.95876867	-0.0607453	0	main		NM_001285	Cdc27	cell division	NM_001285	RefSeq	Mus muscul	FALSE	217232	Mm.89845	A2A6Q5	chr11	GO:000709	metaphase/a	GO:000563	nucleus // n	GO:001990	protein phos	○
17513297	84.0304	87.64886	0.95871641	-0.060824	0	main		NM_029441	Cdyl2	chromodom	NM_029441	RefSeq	Mus muscul	FALSE	75796	Mm.159575	Q9D5D8	chr8	GO:000815	metabolic pr	GO:000563	nucleus // n	GO:000382	catalytic acti	○
17413384	154.3287	161.1458	0.95769607	-0.0623602	0	main		NM_172866	Rgp1	RGP1 retrog	NM_172866	RefSeq	Mus muscul	FALSE	242406	Mm.259311	Q8BHT7	chr4	GO:004214	retrograde tr	GO:000573	cytoplasm /	GO:000508	guanyl-nucle	○
17268493	265.7139	277.6746	0.95692548	-0.0635215	0	main		NM_008942	Npepps	aminopeptid	NM_008942	RefSeq	Mus muscul	FALSE	19155	Mm.29824	Q11011	chr11	GO:000650	proteolysis	GO:000563	nucleus // c	GO:000417	aminopeptid	○
17217127	265.7737	277.89	0.95639893	-0.0643156	0	main		NM_001145	Nucks1	nuclear case	NM_001145	RefSeq	Mus muscul	FALSE	98415	Mm.246869	Q80XU3	chr1	GO:004259	glucose hom	GO:000563	nucleus // n	GO:000107	transcription	○
17502754	272.9834	286.0241	0.95440699	-0.0673235	0	main		NM_001081	Otud4	OTU domain	NM_001081	RefSeq	Mus muscul	FALSE	73945	Mm.34348	B2RRE7	chr8	GO:000650	proteolysis	GO:000557	cellular_com	GO:000484	ubiquitin-spe	○
17221317	198.1384	207.8641	0.95321126	-0.0691321	0	main		NM_001302	Ncoa2	nuclear rece	NM_001302	RefSeq	Mus muscul	FALSE	17978	Mm.2537	Q61026	chr1	GO:000012	negative reg	GO:000563	nucleus // n	GO:000097	RNA polyme	○
17313284	376.3059	395.5622	0.95131916	-0.0719987	0	main		NM_177821	Ep300	E1A binding	NM_177821	RefSeq	Mus muscul	FALSE	328572	Mm.258397	B2RWS6	chr15	GO:000012	negative reg	GO:000012	histone acet	GO:000097	RNA polyme	○
17255877	98.68607	103.8069	0.95066966	-0.072984	0	main		NM_010688	Lasp1	LIM and SH	NM_010688	RefSeq	Mus muscul	FALSE	16796	Mm.271967	Q61792	chr11	GO:000681	transport //	GO:000573	cytoplasm /	GO:000377	actin binding	○
17405142	140.7131	148.8133	0.94556804	-0.0807468	0	main		NM_080793	Setd7	SET domain	NM_080793	RefSeq	Mus muscul	FALSE	73251	Mm.192111	Q8VHL1	chr3	GO:000635	transcription	GO:000563	nucleus // c	GO:000203	p53 binding	○
17414301	316.3206	334.686	0.94512648	-0.0814207	0	main		NM_009011	Rad23b	RAD23b hor	NM_009011	RefSeq	Mus muscul	FALSE	19359	Mm.196846	P54728	chr4	GO:000071	nucleotide-e	GO:000050	proteasome	GO:000368	damaged DI	○
17243525	53.96336	57.12748	0.944613	-0.0822047	0	main		NM_177388	Slc41a2	solute carrie	NM_177388	RefSeq	Mus muscul	FALSE	338365	Mm.67073	Q8BYR8	chr10	GO:000681	transport //	GO:000588	plasma men	GO:000832	cation trans	○
17281388	52.08414	55.14585	0.94447977	-0.0824082	0	main		NM_001033	Fbxo33	F-box protei	NM_001033	RefSeq	Mus muscul	FALSE	70611	Mm.311026	Q8VE08	chr12	GO:000815	biological_p	GO:000557	cellular_com	GO:000367	molecular_fu	○
17466982	72.94467	77.23427	0.94445989	-0.0824386	0	main		NM_027268	Scrn1	secernin 1	NM_027268	RefSeq	Mus muscul	FALSE	69938	Mm.41557	Q9CZC8	chr6	GO:000650	proteolysis	GO:000563	nucleus // c	GO:001680	dipeptidase	○
17447510	191.2134	202.6133	0.94373568	-0.0835452	0	main		NM_026242	Mrfap1	Morf4 family	NM_026242	RefSeq	Mus muscul	FALSE	67568	Mm.490257	Q9CQL7	chr5	GO:000815	biological_p	GO:000563	nucleus // n	GO:000551	protein bindi	○
17371148	12.22191	12.96029	0.94302751	-0.0846282	0	main		NM_009322	Tbr1	T-box brain	NM_009322	RefSeq	Mus muscul	FALSE	21375	Mm.308525	Q64336	chr2	GO:000166	conditioned	GO:000563	nucleus // n	GO:000097	RNA polyme	○
17349774	65.7645	69.77926	0.94246485	-0.0854893	0	main		NM_001305	Hars2	histidyl-tRNA	NM_001305	RefSeq	Mus muscul	FALSE	70791	Mm.282700	Q99KK9	chr18	GO:000641	translation /	GO:000573	cytoplasm /	GO:000016	nucleotide b	○
17420761	75.40842	80.15652	0.94076464	-0.0880943	0	main		NM_001205	Iffo2	intermediate	NM_001205	RefSeq	Mus muscul	FALSE	212632	Mm.30464	Q8R2V2	chr4	GO:000815	biological_p	GO:000588	intermediate	GO:000519	structural m	○
17355962	175.6245	186.7519	0.94041613	-0.0886288	0	main		NM_001167	Suv420h1	suppressor	NM_001167	RefSeq	Mus muscul	FALSE	225888	Mm.278578	Q3U8K7	chr19	GO:000635	transcription	GO:000078	condensed	GO:000816	methyltransf	○
17291559	32.365	34.43858	0.93978904	-0.0895912	0	main		NM_001289	E2f3	E2F transcri	NM_001289	RefSeq	Mus muscul	FALSE	13557	Mm.268356	O35261	chr13	GO:000635	transcription	GO:000563	nucleus // n	GO:000104	core promot	○
17324774	240.7356	256.5869	0.93822249	-0.091998	0	main		NM_177633	Ubxn7	UBX domain	NM_177633	RefSeq	Mus muscul	FALSE	224111	Mm.31275	Q6P5G6	chr16	GO:000815	biological_p	GO:000563	nucleus // n	GO:000813	transcription	○
17321326	102.5543	109.3113	0.93818571	-0.0920546	0	main		NM_007478	Arf3	ADP-ribosyl	NM_007478	RefSeq	Mus muscul	FALSE	11842	Mm.221298	P61205	chr15	GO:000681	transport //	GO:000562	intracellular	GO:000016	nucleotide b	○
17454157	78.6429	83.99852	0.9362415	-0.0950474	0	main		NM_144913	Mepce	methylphos	NM_144913	RefSeq	Mus muscul	FALSE	231803	Mm.61193	Q8K3A9	chr5	GO:000012	negative reg	GO:000557	cellular_com	GO:000017	rRNA (aden	○
17265657	61.51756	65.81596	0.93469061	-0.0974392	0	main		NM_177776	Smtnl2	smoothelin-l	NM_177776	RefSeq	Mus muscul	FALSE	276829	Mm.46732	Q8CI12	chr11	GO:000815	biological_p	GO:000557	cellular_com	GO:000367	molecular_fu	○
17377126	74.99049	80.23766	0.93460465	-0.0975719	0	main		NM_025314	Dtd1	D-tyrosyl-tR	NM_025314	RefSeq	Mus muscul	FALSE	66044	Mm.28109	Q9DD18	chr2	GO:000626	DNA replica	GO:000563	nucleus // c	GO:000216	aminoacyl-tR	○
17260786	40.18278	43.36451	0.92662825	-0.1099374	0	main		NM_026576	Etaa1	Ewing tumor	NM_026576	RefSeq	Mus muscul	FALSE	68145	Mm.178684	Q5SVT3	chr11	GO:000815	biological_p	GO:000573	cytoplasm	GO:000367	molecular_fu	○
17252113	145.5254	157.0524	0.92660411	-0.109975	0	main		NM_001045	Mink1	misshapen-l	NM_001045	RefSeq	Mus muscul	FALSE	50932	Mm.42967	Q9JM52	chr11	GO:000016	MAPK casca	GO:000573	cytoplasm /	GO:000016	nucleotide b	○
17378640	100.3843	108.5014	0.92518898	-0.11218	0	main		NM_001042	Dlgap4	discs, large	NM_001042	RefSeq	Mus muscul	FALSE	228836	Mm.22094	B1AZP2	chr2	GO:002305	signaling //	GO:000588	plasma men	GO:000551	protein bindi	○
17479548	198.3587	214.7377	0.92372555	-0.1144638	0	main		NM_178070	Vps33b	vacuolar pro	NM_178070	RefSeq	Mus muscul	FALSE	233405	Mm.322870	P59016	chr7	GO:000681	transport //	GO:000573	cytoplasm /	GO:000551	protein bindi	○
17489707	220.3913	238.7547	0.92308675	-0.1154619	0	main		NM_009884	Cebpg	CCAAT/enh	NM_009884	RefSeq	Mus muscul	FALSE	12611	Mm.273090	P53568	chr7	GO:000633	nucleosome	GO:000563	nucleus // n	GO:000097	RNA polyme	○
17284839	49.36682	53.58744	0.92123863	-0.1183532	0	main		NM_177290	Itgb8	integrin beta	NM_177290	RefSeq	Mus muscul	FALSE	320910	Mm.217000	Q0VBD0	chr12	GO:000157	ganglioside	GO:000998	cell surface	GO:000510	receptor bin	○
17511868	164.9837	179.3222	0.92004058	-0.1202306	0	main		NM_028221	Fam192a	family with s	NM_028221	RefSeq	Mus muscul	FALSE	102122	Mm.250425	Q91WE2	chr8	GO:000815	biological_p	GO:000563	nucleus // n	GO:000367	molecular_fu	○
17452456	73.18999	79.79966	0.9171717	-0.1247363	0	main		NM_001199	Camkk2	calcium/calm	NM_001199	RefSeq	Mus muscul	FALSE	207565	Mm.289237	Q8C078	chr5	GO:000646	protein phos	GO:000563	nucleus // c	GO:000016	nucleotide b	○
17285000	474.5063	517.7437	0.9164888	-0.1258109	0	main		NM_011803	Klf6	Kruppel-like	NM_011803	RefSeq	Mus muscul	FALSE	23849	Mm.275036	O08584	chr13	GO:000635	transcription	GO:000563	nucleus // n	GO:000367	nucleic acid	○
17211774	101.451	110.7301	0.91620074	-0.1262644	0	main		NM_145516	Plekhb2	pleckstrin ho	NM_145516	RefSeq	Mus muscul	FALSE	226971	Mm.292751	Q9QZC7	chr1	GO:000815	biological_p	GO:000576	endosome /	GO:000367	molecular_fu	○
17337919	32.74117	35.74555	0.91595094	-0.1266578	0	main		NM_013688	Tcte1	t-complex-as	NM_013688	RefSeq	Mus muscul	FALSE	21645	Mm.491193	A6H639	chr17	GO:000815	biological_p	GO:000557	cellular_com	GO:000367	molecular_fu	○
17297196	163.8526	178.9966	0.91539504	-0.1275336	0	main		NM_139227	Atxn7	ataxin 7	NM_139227	RefSeq	Mus muscul	FALSE	246103	Mm.133625	Q8R4I1	chr14	GO:000022	microtubule	GO:000563	nucleus // n	GO:000368	chromatin bi	○
17309161	247.533	270.8407	0.91394314	-0.1298237	0	main		NM_015822	Fbxl3	F-box and le	NM_015822	RefSeq	Mus muscul	FALSE	50789	Mm.214746	Q8C4V4	chr14	GO:001656	protein ubiq	GO:000563	nucleus // n	GO:000484	ubiquitin-pro	○
17341681	77.83955	85.18712	0.91374788	-0.1301319	0	main		NM_027008	Kctd5	potassium c	NM_027008	RefSeq	Mus muscul	FALSE	69259	Mm.28171	Q8VC57	chr17	GO:005126	protein hom	GO:000573	cytoplasm /	GO:003240	protein com	○
17220724	249.7359	273.7484	0.91228259	-0.1324473	0	main		NM_008976	Ptpn14	protein tyros	NM_008976	RefSeq	Mus muscul	FALSE	19250	Mm.4498	Q62130	chr1	GO:000194	lymphangio	GO:000563	nucleus // n	GO:000371	transcription	○
17322642	102.5988	112.5266	0.91177375	-0.1332522	0	main		NM_026666	Ubn1	ubinuclein 1	NM_026666	RefSeq	Mus muscul	FALSE	170644	Mm.66356	Q4G0F8	chr16	GO:000633	DNA replica	GO:000563	nucleus // b	GO:000367	DNA binding	○
17379582	153.6045	169.101	0.9083595	-0.1386647	0	main		NM_146129	Pcif1	PDX1 C-terr	NM_146129	RefSeq	Mus muscul	FALSE	228866	Mm.241134	P59114	chr2	GO:001092	negative reg	GO:000563	nucleus // n	GO:000367	molecular_fu	○
17418268	17.87395	19.6862	0.90794313	-0.1393262	0	main		NM_011141	Pou3f1	POU domain	NM_011141	RefSeq	Mus muscul	FALSE	18991	Mm.297371	P21952	chr4	GO:000635	transcription	GO:000563	nucleus // tr	GO:000110	RNA polyme	○
17465212	330.2569	364.1455	0.90693665	-0.1409263	0	main		NM_001167	Wasl	Wiskott-Aldr	NM_001167	RefSeq	Mus muscul	FALSE	73178	Mm.1574	Q91YD9	chr6	GO:000635	transcription	GO:000013	Golgi memb	GO:000377	actin binding	○

17239090	291.9802	321.9822	0.90682094	-0.1411104	0	main		NM_138667	Tab2	TGF-beta ac	NM_138667	RefSeq	Mus muscul	FALSE	68652	Mm.193041	Q99K90	chr10	GO:000750	heart develo	GO:000563	nucleus // c	GO:000551	protein bindi	O
17466798	30.94766	34.13578	0.90660474	-0.1414544	0	main		NM_001311	Hoxa1	homeobox A	NM_001311	RefSeq	Mus muscul	FALSE	15394	Mm.197	P09022	chr6	GO:000635	transcription	GO:000563	nucleus	GO:000367	DNA binding	O
17414802	45.32561	50.01162	0.90630158	-0.1419369	0	main		NM_021362	Pappa	pregnancy-a	NM_021362	RefSeq	Mus muscul	FALSE	18491	Mm.317854	Q8R4K8	chr4	GO:000650	proteolysis	GO:000557	extracellular	GO:000417	endopeptida	O
17285438	24.98438	27.60077	0.90520591	-0.1436821	0	main		NM_008380	Inhba	inhibin beta-	NM_008380	RefSeq	Mus muscul	FALSE	16323	Mm.8042	Q04998	chr13	GO:000008	G1/S transit	GO:000557	extracellular	GO:000510	receptor bin	O
17523852	158.6068	175.4357	0.90407369	-0.1454877	0	main		NM_001291	Birc2	baculoviral I	NM_001291	RefSeq	Mus muscul	FALSE	11797	Mm.335659	Q62210	chr9	GO:000020	protein poly	GO:000174	XY body // r	GO:000371	transcription	O
17440974	43.94994	48.6448	0.90348691	-0.1464244	0	main		NM_001004	Fam222a	family with s	NM_001004	RefSeq	Mus muscul	FALSE	433940	Mm.194212	Q6PGH4	chr5	GO:000815	biological_p	GO:000557	cellular_com	GO:000367	molecular_fu	O
17339630	424.9077	471.4305	0.90131568	-0.1498956	0	main		NM_007566	Birc6	baculoviral I	NM_007566	RefSeq	Mus muscul	FALSE	12211	Mm.442564	O88738	chr17	GO:000189	placenta de	GO:000092	spindle pole	GO:000484	ubiquitin-pro	O
17448467	57.61747	64.08509	0.89907762	-0.1534824	0	main		NM_001164	Bend4	BEN domain	NM_001164	RefSeq	Mus muscul	FALSE	666938	Mm.101339	P86174	chr5	GO:000815	biological_p	GO:000557	cellular_com	GO:000367	molecular_fu	O
17470060	123.5399	137.5229	0.89832239	-0.1546948	0	main		NM_178045	Rassf4	Ras associa	NM_178045	RefSeq	Mus muscul	FALSE	213391	Mm.257931	Q8CB96	chr6	GO:000704	cell cycle //	GO:000557	cellular_com	GO:000367	molecular_fu	O
17218233	26.62391	29.63811	0.89829986	-0.154731	0	main		NM_177756	Colgalt2	collagen bet	NM_177756	RefSeq	Mus muscul	FALSE	269132	Mm.23782	Q6NVG7	chr1	GO:000815	biological_p	GO:000578	endoplasmic	GO:001674	transferase	O
17417437	225.2395	251.1756	0.89674116	-0.1572365	0	main		NM_029868	Gpbp1l1	GC-rich pro	NM_029868	RefSeq	Mus muscul	FALSE	77110	Mm.41715	Q6NZP2	chr4	GO:000635	transcription	GO:000563	nucleus // n	GO:000367	DNA binding	O
17457892	111.3178	124.1933	0.89632694	-0.157903	0	main		NM_007610	Casp2	caspase 2	NM_007610	RefSeq	Mus muscul	FALSE	12366	Mm.3921	P29594	chr6	GO:000650	proteolysis	GO:000563	nucleus // c	GO:000419	cysteine-typ	O
17548736	27.1242	30.27794	0.89584034	-0.1586865	0	main		XM_006510	Ntm	neurotrimin	XM_006510	RefSeq	PREDICTEI	FALSE	235106	Mm.283138	Q99PJ0	chr9	GO:000715	cell adhesio	GO:000561	extracellular	---	---	O
17360068	280.9461	313.8358	0.89520093	-0.1597166	0	main		NM_001177	Wbp1l	WW domain	NM_001177	RefSeq	Mus muscul	FALSE	226178	Mm.329895	Q8BGW2	chr19	GO:000815	biological_p	GO:001602	membrane	GO:000367	molecular_fu	O
17299288	83.87105	93.69013	0.89519622	-0.1597242	0	main		NM_001037	Samd4	sterile alpha	NM_001037	RefSeq	Mus muscul	FALSE	74480	Mm.214454	Q8CBY1	chr14	GO:000635	regulation o	GO:000573	cytoplasm /	GO:000372	RNA binding	O
17467608	148.615	166.2211	0.89408023	-0.1615238	0	main		NM_024288	Rmnd5a	required for	NM_024288	RefSeq	Mus muscul	FALSE	68477	Mm.28474	Q80YQ8	chr6	GO:000815	biological_p	GO:000557	cellular_com	GO:000367	molecular_fu	O
17328201	161.789	181.0224	0.89375127	-0.1620547	0	main		NM_001130	Gspt1	G1 to S pha	NM_001130	RefSeq	Mus muscul	FALSE	14852	Mm.325827	Q8R050	chr16	GO:000018	nuclear-tran	GO:000582	cytosol // tra	GO:000016	nucleotide b	O
17496253	456.9428	511.3192	0.89365469	-0.1622106	0	main		NM_183020	Atxn2l	ataxin 2-like	NM_183020	RefSeq	Mus muscul	FALSE	233871	Mm.231450	Q7TQH0	chr7	GO:001060	regulation o	GO:000573	cytoplasm /	GO:004482	poly(A) RNA	O
17345321	49.88082	55.86045	0.89295414	-0.163342	0	main		NM_030715	Polh	polymerase	NM_030715	RefSeq	Mus muscul	FALSE	80905	Mm.311585	Q9JJN0	chr17	GO:000073	DNA synthe	GO:000563	nucleus // n	GO:000367	DNA binding	O
17293237	62.98566	70.70172	0.89086461	-0.1667219	0	main		NM_001035	Golm1	golgi membr	NM_001035	RefSeq	Mus muscul	FALSE	105348	Mm.171335	Q91XA2	chr13	GO:000699	nucleus orga	GO:000561	extracellular	GO:000367	molecular_fu	O
17257405	91.37141	102.7492	0.88926639	-0.1693124	0	main		NM_181071	Tanc2	tetratricope	NM_181071	RefSeq	Mus muscul	FALSE	77097	Mm.22501	A2A690	chr11	GO:000170	in utero emb	GO:000557	cellular_com	GO:000367	molecular_fu	O
17537756	144.0379	162.38	0.88704212	-0.1729255	0	main		NM_027870	Armcx3	armadillo re	NM_027870	RefSeq	Mus muscul	FALSE	71703	Mm.67949	Q8BHS6	chrX	GO:003461	cellular prot	GO:001602	membrane	GO:000551	protein bindi	O
17241454	226.7472	256.1082	0.88535705	-0.1756687	0	main		NM_053183	Ddx50	DEAD (Asp-	NM_053183	RefSeq	Mus muscul	FALSE	94213	Mm.114116	Q99MJ9	chr10	GO:001050	RNA second	GO:000563	nucleus // n	GO:000016	nucleotide b	O
17323360	85.16828	96.2431	0.88492869	-0.1763669	0	main		NM_176833	Ppm1f	protein phos	NM_176833	RefSeq	Mus muscul	FALSE	68606	Mm.230296	Q8CGA0	chr16	GO:000646	negative reg	GO:000562	intracellular	GO:000382	catalytic acti	O
17260150	89.54873	101.2187	0.8847054	-0.176731	0	main		NM_001080	Znrf3	zinc and ring	NM_001080	RefSeq	Mus muscul	FALSE	407821	Mm.216313	Q5SSZ7	chr11	GO:000651	ubiquitin-de	GO:000588	plasma mem	GO:000484	ubiquitin-pro	O
17403681	133.3605	150.9372	0.88354958	-0.178617	0	main		NM_001080	Zzz3	zinc finger, Z	NM_001080	RefSeq	Mus muscul	FALSE	108946	Mm.379292	Q6KAQ7	chr3	GO:000635	transcription	GO:000563	nucleus // A	GO:000367	DNA binding	O
17430777	79.47932	90.25462	0.88061221	-0.1834213	0	main		NM_001122	Gmeb1	glucocortico	NM_001122	RefSeq	Mus muscul	FALSE	56809	Mm.103489	Q9JL60	chr4	GO:000635	transcription	GO:000563	nucleus // c	GO:000097	RNA polyme	O
17231188	89.03268	101.7966	0.87461349	-0.1932825	0	main		NM_145415	Diexf	digestive org	NM_145415	RefSeq	Mus muscul	FALSE	215193	Mm.173758	Q8BTT6	chr1	GO:000046	maturation o	GO:000563	nucleus // n	GO:001984	rRNA bindin	O
17417290	125.1987	143.1911	0.87434694	-0.1937222	0	main		NM_181040	Atpaf1	ATP syntha	NM_181040	RefSeq	Mus muscul	FALSE	230649	Mm.109600	Q811I0	chr4	GO:000646	protein com	GO:000573	mitochondri	GO:000367	molecular_fu	O
17349590	247.9713	283.639	0.87424966	-0.1938828	0	main		NM_019912	Ube2d2a	ubiquitin-con	NM_019912	RefSeq	Mus muscul	FALSE	56550	Mm.180052	P62838	chr18	GO:000020	protein poly	GO:000015	ubiquitin liga	GO:000016	nucleotide b	O
17376822	30.15712	34.53436	0.87324971	-0.1955338	0	main		NM_001291	Snap25	synaptosom	NM_001291	RefSeq	Mus muscul	FALSE	20614	Mm.45953	P60879	chr2	GO:000688	exocytosis	GO:000573	cytoplasm /	GO:000014	SNARE bind	O
17323532	37.334	42.75389	0.87323048	-0.1955656	0	main		NM_001159	P2rx6	purinergic re	NM_001159	RefSeq	Mus muscul	FALSE	18440	Mm.8032	O54803	chr16	GO:000681	transport //	GO:000563	integral com	GO:000161	purinergic nu	O
17214841	321.2827	369.881	0.86861099	-0.2032179	0	main		NM_001310	Agfg1	ArfGAP with	NM_001310	RefSeq	Mus muscul	FALSE	15463	Mm.433409	Q8K2K6	chr1	GO:000167	acrosome a	GO:000563	nucleus // c	GO:000367	DNA binding	O
17512846	148.5157	171.0928	0.86804179	-0.2041636	0	main		NM_001080	Atxn1l	ataxin 1-like	NM_001080	RefSeq	Mus muscul	FALSE	52335	Mm.276770	P0C7T6	chr8	GO:000012	negative reg	GO:000563	nucleus // n	GO:000367	DNA binding	O
17255402	315.3216	363.2942	0.8679511	-0.2043143	0	main		NM_025287	Spop	speckle-type	NM_025287	RefSeq	Mus muscul	FALSE	20747	Mm.285454	Q6ZWS8	chr11	GO:000012	negative reg	GO:000563	nucleus // n	GO:000108	RNA polyme	O
17397439	188.0045	216.8257	0.86707664	-0.2057686	0	main		NM_053089	Naa15	N(alpha)-ac	NM_053089	RefSeq	Mus muscul	FALSE	74838	Mm.275281	Q80UM3	chr3	GO:000152	angiogenesi	GO:000563	nucleus // tr	GO:000459	peptide alph	O
17526382	277.9572	321.4497	0.86469889	-0.2097303	0	main		NM_001081	Kmt2a	lysine (K)-sp	NM_001081	RefSeq	Mus muscul	FALSE	214162	Mm.2389	P55200	chr9	GO:000630	DNA methyl	GO:000563	nucleus // n	GO:000104	core promot	O
17264651	166.9523	193.7873	0.86152343	-0.2150381	0	main		NM_001017	Kdm6b	KDM1 lysine	NM_001017	RefSeq	Mus muscul	FALSE	216850	Mm.261201	Q5NCY0	chr11	GO:000695	inflammator	GO:000563	nucleus // n	GO:000090	sulfonate dio	O
17348202	123.8977	143.9555	0.86066666	-0.2164735	0	main		NM_011546	Zeb1	zinc finger E	NM_011546	RefSeq	Mus muscul	FALSE	21417	Mm.3929	Q64318	chr18	GO:000012	negative reg	GO:000563	nucleus // n	GO:000122	transcription	O
17496973	261.7639	304.6029	0.85936116	-0.2186635	0	main		NM_001029	Ate1	arginyltransf	NM_001029	RefSeq	Mus muscul	FALSE	11907	Mm.216321	Q9Z2A5	chr7	GO:001659	protein argir	GO:000563	nucleus // n	GO:000405	arginyltransf	O
17213321	1605.34	1870.064	0.85844121	-0.2202088	0	main		NM_007561	Bmpr2	bone morph	NM_007561	RefSeq	Mus muscul	FALSE	12168	Mm.7106	O35607	chr1	GO:000170	mesoderm f	GO:000561	extracellular	GO:000016	nucleotide b	O
17467823	536.0108	625.5967	0.85679928	-0.2229708	0	main		NM_019715	Kcmf1	potassium c	NM_019715	RefSeq	Mus muscul	FALSE	74287	Mm.29194	Q80UY2	chr6	GO:001656	protein ubiq	---	---	GO:000827	zinc ion bind	O
17287613	286.4705	334.6676	0.85598516	-0.2243423	0	main		NM_008739	Nsd1	nuclear rece	NM_008739	RefSeq	Mus muscul	FALSE	18193	Mm.168965	O88491	chr13	GO:000012	negative reg	GO:000563	nucleus // n	GO:000097	RNA polyme	O
17344472	62.14121	72.66191	0.85521025	-0.225649	0	main		NM_001142	Atat1	alpha tubulin	NM_001142	RefSeq	Mus muscul	FALSE	73242	Mm.273155	Q8K341	chr17	GO:000728	spermatoge	GO:000573	cytoplasm /	GO:001674	transferase	O
17303757	44.64601	52.31484	0.85341005	-0.228689	0	main		NM_134081	Dnajc9	DnaJ (Hsp4	NM_134081	RefSeq	Mus muscul	FALSE	108671	Mm.2871	Q91WN1	chr14	GO:003278	positive reg	GO:000561	extracellular	GO:003107	heat shock p	O
17267304	138.4841	162.8956	0.85014021	-0.2342273	0	main		NM_001114	Rps6kb1	ribosomal pr	NM_001114	RefSeq	Mus muscul	FALSE	72508	Mm.394280	Q8BSK8	chr11	GO:000008	G1/S transit	GO:000563	nucleus // n	GO:000016	nucleotide b	O
17472777	204.2106	240.4651	0.84923176	-0.2357698	0	main		NM_010586	Itpr2	inositol 1,4,5	NM_010586	RefSeq	Mus muscul	FALSE	16439	Mm.7800	Q9Z329	chr6	GO:000166	response to	GO:000573	cytoplasm /	GO:000521	ion channel	O
17535607	274.9536	323.8177	0.84909997	-0.2359937	0	main		NM_001142	Slc6a8	solute carrie	NM_001142	RefSeq	Mus muscul	FALSE	102857	Mm.274553	Q8VBW1	chrX	GO:000681	transport //	GO:000588	integral com	GO:000530	creatine tran	O
17424850	174.7722	205.8544	0.84900881	-0.2361486	0	main		NM_001038	Rnf38	ring finger p	NM_001038	RefSeq	Mus muscul	FALSE	73469	Mm.262859	Q8BI21	chr4	GO:001656	protein ubiq	GO:000563	nucleus // n	GO:000484	ubiquitin-pro	O
17248929	103.0886	121.44	0.84888505	-0.2363589	0	main		NM_001024	Zfp62	zinc finger p	NM_001024	RefSeq	Mus muscul	FALSE	22720	Mm.16650	Q8C827	chr11	GO:000635	transcription	GO:000563	nucleus	GO:000367	nucleic acid	O
17325014	262.105	309.1382	0.84785704	-0.2381071	0	main		NM_080557	Snx4	sorting nexin	NM_080557	RefSeq	Mus muscul	FALSE	69150	Mm.28196	Q91YJ2	chr16	GO:000681	transport //	GO:000573	cytoplasm /	GO:000515	epidermal g	O
17353143	153.0875	180.7326	0.84703866	-0.2395003	0	main		NM_144861	Rprd1a	regulation o	NM_144861	RefSeq	Mus muscul	FALSE	225283	Mm.28427	Q8VDS4	chr18	GO:007094	dephosphor	GO:001659	DNA-directe	GO:000367	molecular_fu	O
17223666	358.7776	424.8667	0.84444745	-0.2439205	0	main		NM_001081	Ino80d	INO80 comp	NM_001081	RefSeq	Mus muscul	FALSE	227195	Mm.74718	Q66JY2	chr1	GO:000628	DNA repair	GO:000563	nucleus // c	GO:000367	molecular_fu	O
17238449	155.9789	185.0954	0.84269463	-0.2469182	0	main		NM_172790	Ankrd52	ankyrin repe	NM_172790	RefSeq	Mus muscul	FALSE	237615	Mm.440177	Q8BTI7	chr10	GO:000815	biological_p	GO:000557	cellular_com	GO:000367	molecular_fu	O
17416897	150.6509	179.1626	0.84086132	-0.2500602	0	main		NM_007983	Faf1	Fas-associa	NM_007983	RefSeq	Mus muscul	FALSE	14084	Mm.318259	P54731	chr4	GO:000691	apoptotic pr	GO:000563	nucleus // n	GO:000551	protein bindi	O
17384787	112.2603	133.9064	0.83834903	-0.2543771	0	main		NM_178778	Scai	suppressor o	NM_178778	RefSeq	Mus muscul	FALSE	320271	Mm.204836	Q8C8N2	chr2	GO:000635	transcription	GO:000563	nucleus // n	GO:000371	transcription	O
17497153	105.1132	125.3863	0.83831487	-0.2544359	0	main		NM_001170	Ctbp2	C-terminal b	NM_001170	RefSeq	Mus muscul	FALSE	13017	Mm.246240	P56546	chr7	GO:000635	transcription	GO:000563	nucleus // n	GO:000368	chromatin bi	O
17436237	288.6224	344.4765	0.83785803	-0.2552223	0	main		NM_008037	Fosl2	fos-like antig	NM_008037	RefSeq	Mus muscul	FALSE	14284	Mm.24684	P47930	chr5	GO:000166	response to	GO:000563	nucleus // n	GO:000097	RNA polyme	O
17321365	223.0084	267.7094	0.83302417	-0.2635697	0	main		NM_001033	Kmt2d	lysine (K)-sp	NM_001033	RefSeq	Mus muscul	FALSE	381022	Mm.264889	Q6PDK2	chr15	GO:000155	oocyte grow	GO:000563	nucleus // h	GO:000551	protein bindi	O
17469097	112.7453	135.3484	0.83300061	-0.2636105	0	main		NM_030081	Rbsn	rabenosyn, I	NM_030081	RefSeq	Mus muscul	FALSE	78287	Mm.290734	Q80Y56	chr6	GO:000001	vacuole inhe	GO:000576	endosome /	GO:001713	Rab GTPas	O
17470235	109.6527	131.8461	0.83167193	-0.2659136	0	main		NM_001159	Cacna1c	calcium cha	NM_001159	RefSeq	Mus muscul	FALSE	12288	Mm.436656	Q01815	chr6	GO:000681	transport //	GO:000209	caveolar ma	GO:000521	ion channel	O
17371912	115.7305	140.2539	0.82514996	-0.2772718	0	main		NM_001164	Zak	sterile alpha	NM_001164	RefSeq	Mus muscul	FALSE	65964	Mm.314618	Q9ESL4	chr2	GO:000007	cell cycle ch	GO:000563	nucleus // c	GO:000016	nucleotide b	O
17373120	190.9029	232.1719	0.82224808	-0.2823544	0	main		NM_001244	Celf1	CUGBP, Ela	NM_001244	RefSeq	Mus muscul	FALSE	13046	Mm.29495	P28659	chr2	GO:000637	mRNA splic	GO:000563	nucleus // n	GO:000016	nucleotide b	O
17228906	99.36606	122.7104	0.8097607	-0.3044325	0	main		NM_001038	Dnm3	dynamin 3	NM_001038	RefSeq	Mus muscul	FALSE	103967	Mm.441620	Q8BZ98	chr1	GO:000689	endocytosis	GO:000191	photorecept	GO:000016	nucleotide b	O
17227536	84.13383	104.0114	0.80889047	-0.3059837	0	main		NM_001111	Ptprc	protein tyros	NM_001111	RefSeq	Mus muscul	FALSE	19264	Mm.391573	P06800	chr1	GO:000018	activation of	GO:000588	plasma mem	GO:000472	phosphopro	O
17322327	193.9375	239.9629	0.80819785	-0.3072196	0	main		NM_001076	Cbx5	chromobox 5	NM_001076	RefSeq	Mus muscul	FALSE	12419	Mm.262059	Q61686	chr15	GO:004589	negative reg	GO:000011	histone dea	GO:000368	chromatin bi	O
17527883	93.81408	116.4939	0.80531324	-0.312378	0	main		NM_001146	Lrrc49	leucine rich	NM_001146	RefSeq	Mus muscul	FALSE	102747	Mm.269413	Q91YK0	chr9	GO:000815	biological_p	GO:000573	cytoplasm /	GO:000367	molecular_fu	O
17266498	178.0527	221.2159	0.80488202	-0.3131508	0	main		NM_008702	Nlk	nemo like ki	NM_008702	RefSeq	Mus muscul	FALSE	18099	Mm.9001	O54949	chr11	GO:000635	transcription	GO:000563	nucleus // c	GO:000016	nucleotide b	O
17283487	188.5602	235.2411	0.80156146	-0.3191149	0	main		NM_172806	Btbd7	BTB (POZ) d	NM_172806	RefSeq	Mus muscul	FALSE	238386	Mm.440255	Q8CFE5	chr12	GO:000727	multicellular	GO:000563	nucleus // n	GO:000367	molecular_fu	O
17523597	185.0129	231.1964	0.80024127	-0.3214931	0	main		NM_013860	Limd1	LIM domains	NM_013860	RefSeq	Mus muscul	FALSE	29806	Mm.10987	Q9QXD8	chr9	GO:000166	response to	GO:000093	cytoplasmic	GO:000371	transcription	O
17224291	86.89172	108.7479	0.79901975	-0.3236969	0	main		NM_001310	Usp37	ubiquitin spe	NM_001310	RefSeq	Mus muscul	FALSE	319651	Mm.66568	Q8C0R0	chr1	GO:000008	G1/S transit	GO:000563	nucleus // n	GO:000419	cysteine-typ	O
17510685	245.6103	307.4504	0.79886154	-0.3239826	0	main		NM_010332	Ednra	endothelin re	NM_010332	RefSeq	Mus muscul	FALSE	13617	Mm.283168	Q61614	chr8	GO:000156	patterning o	GO:000582	cytosol // pl	GO:000471	protein tyros	O
17392991	142.0703	178.1121	0.79764542	-0.3261805	0	main		NM_001289	Bcl2l1	BCL2-like 1	NM_001289	RefSeq	Mus muscul	FALSE	12048	Mm.238213	Q64373	chr2	GO:000091	cytokinesis	GO:000562	intracellular	GO:000551	protein bindi	O
17270134	195.9595	245.9175	0.79685057	-0.3276189	0	main		NM_001044	Ubtf	upstream bi	NM_001044	RefSeq	Mus muscul	FALSE	21429	Mm.2845	P25976	chr11	GO:000018	chromatin si	GO:000165	fibrillar cente	GO:000367	DNA binding	O
17496251	44.81084	56.3889	0.79467484	-0.3315634	0	main		NM_001289	Sh2b1	SH2B adapt	NM_001289	RefSeq	Mus muscul	FALSE	20399	Mm.8538	Q91ZM2	chr7	GO:000692	movement o	GO:000172	ruffle // nucl	GO:000487	signal transd	O
17352493	173.7678	219.5239	0.79156666	-0.3372172	0	main		NM_001276	Epc1	enhancer of	NM_001276	RefSeq	Mus muscul	FALSE	13831	Mm.312133	Q8C9X6	chr18	GO:000012	negative reg	GO:000563	nucleus // n	GO:000440	histone acet	O
17440257	103.3728	130.8998	0.78970938	-0.3406063	0	main		NM_172716	Pcgf3	polycomb gr	NM_172716	RefSeq	Mus muscul	FALSE	69587	Mm.486543	Q8BTQ0	chr5	GO:000635	transcription	GO:000563	nucleus // n	GO:000827	zinc ion bind	O
17213336	231.0211	292.7206	0.78922051	-0.3414997	0	main		NM_007561	Bmpr2	bone morph	NM_007561	RefSeq	Mus muscul	FALSE	12168	Mm.7106	O35607	chr1	GO:000170	mesoderm f	GO:000561	extracellular	GO:000016	nucleotide b	O
17524087	140.3199	177.9557	0.78851029	-0.3427985	0	main		NM_019683	Ankrd49	ankyrin repe	NM_019683	RefSeq	Mus muscul	FALSE	56503	Mm.395005	Q8VE42	chr9	GO:000728	spermatoge	GO:000563	nucleus // c	GO:000367	molecular_fu	O
17289527	46.69418	59.23219	0.78832439	-0.3431387	0	main		NM_008533	Cd180	CD180 antig	NM_008533	RefSeq	Mus muscul	FALSE	17079	Mm.373974	Q62192	chr13	GO:000232	B cell prolife	GO:000588	plasma mem	---	---	O
17276732	147.6856	187.7219	0.78672547	-0.3460678	0	main		NM_026114	Eif2s1	eukaryotic tr	NM_026114	RefSeq	Mus muscul	FALSE	13665	Mm.196220	Q6ZWX6	chr12	GO:000223	positive reg	GO:000563	nucleus // n	GO:000367	nucleic acid	O
17229454	77.18172	98.45354	0.78394053	-0.3511839	0	main		NM_009062	Rgs4	regulator of	NM_009062	RefSeq	Mus muscul	FALSE	19736	Mm.41642	O08899	chr1	GO:000718	G-protein co	GO:000563	nucleus // c	GO:000196	G-protein al	O
17469121	61.30748	78.25469	0.78343522	-0.3521141	0	main		NM_001081	Prickle2	prickle hom	NM_001081	RefSeq	Mus muscul	FALSE	243548	Mm.248820	Q80Y24	chr6	GO:003117	neuron proje	GO:000563	nucleus // c	GO:000551	protein bindi	O
17363704	67.2994	86.41212	0.77881899	-0.36064	0	main		NM_001166	Rfx3	regulatory fa	NM_001166	RefSeq	Mus muscul	FALSE	19726	Mm.313321	P48381	chr19	GO:000635	transcription	GO:000079	nuclear chro	GO:000097	RNA polyme	O
17353765	116.0966	149.3508	0.77734167	-0.3633792	0	main		NM_008214	Hars	histidyl-tRN	NM_008214	RefSeq	Mus muscul	FALSE	15115	Mm.10528	Q61035	chr18	GO:000641	translation /	GO:000573	cytoplasm /	GO:000016	nucleotide b	O
17405397	36.14605	46.54868	0.77652148	-0.3649023	0	main		NM_019410	Pfn2	profilin 2	NM_019410	RefSeq	Mus muscul	FALSE	18645	Mm.271744	Q9JJV2	chr3	GO:001063	negative reg	GO:000573	cytoplasm /	GO:000077	adenyl-nucle	O
17510013	67.7812	87.37492	0.77575121	-0.3663341	0	main		NM_001122	Upf1	UPF1 regula	NM_001122	RefSeq	Mus muscul	FALSE	19704	Mm.390048	Q9EPU0	chr8	GO:000018	nuclear-tran	GO:000078	chromatin /	GO:000016	nucleotide b	O
17521405	57.68803	74.45363	0.77481823	-0.3680702	0	main		NM_001243	Rassf1	Ras associa	NM_001243	RefSeq	Mus muscul	FALSE	56289	Mm.12091	Q99MK9	chr9	GO:000697	cellular resp	GO:000563	nucleus // c	GO:000551	protein bindi	O
17527565	81.75107	105.8284	0.77248706	-0.3724173	0	main		NM_001304	Csk	c-src tyrosin	NM_001304	RefSeq	Mus muscul	FALSE	12988	Mm.21974	P41241	chr9	GO:000181	regulation o	GO:000573	cytoplasm /	GO:000016	nucleotide b	O
17287081	45.6678	59.50406	0.76747368	-0.3818108	0	main		NM_031166	Id4	inhibitor of D	NM_031166	RefSeq	Mus muscul	FALSE	15904	Mm.458006	P41139	chr13	GO:000008	G1/S transit	GO:000563	nucleus // c	GO:000108	RNA polyme	O
17533416	324.6757	427.4375	0.75958637	-0.3967141	0	main		NM_010028	Ddx3x	DEAD/H (As	NM_010028	RefSeq	Mus muscul	FALSE	13205	Mm.289662	Q62167	chrX	GO:000237	immune sys	GO:000563	nucleus // c	GO:000016	nucleotide b	O
17523736	60.1301	79.36652	0.75762551	-0.4004432	0	main		NM_029851	Dync2h1	dynein cytop	NM_029851	RefSeq	Mus muscul	FALSE	110350	Mm.237596	Q45VK7	chr9	GO:000701	microtubule-	GO:000573	cytoplasm /	GO:000016	nucleotide b	O
17348138	269.934	356.77	0.7566051	-0.4023876	0	main		NM_001081	9430020K01	RIKEN cDN	NM_001081	RefSeq	Mus muscul	FALSE	240185	Mm.33179	Q5DTX6	chr18	GO:000715	cell adhesio	GO:000591	cell-cell junc	GO:000367	molecular_fu	O
17315152	116.1495	153.5162	0.75659442	-0.402408	0	main		NM_007395	Acvr1b	activin A rec	NM_007395	RefSeq	Mus muscul	FALSE	11479	Mm.308467	Q61271	chr15	GO:000008	G1/S transit	GO:000588	plasma mem	GO:000016	nucleotide b	O
17543290	64.52078	86.55167	0.74545968	-0.4237978	0	main		NM_001033	Arhgef9	CDC42 guan	NM_001033	RefSeq	Mus muscul	FALSE	236915	Mm.44841	Q3UTH8	chrX	GO:000726	small GTPa	GO:000573	cytoplasm /	GO:000508	guanyl-nucle	O
17264424	158.5965	213.3341	0.74341842	-0.4277537	0	main		NM_023668	Ndel1	nuclear distr	NM_023668	RefSeq	Mus muscul	FALSE	83431	Mm.31979	Q9ERR1	chr11	GO:000013	establishme	GO:000077	chromosome	GO:000551	protein bindi	O
17317015	191.0211	257.2965	0.74241624	-0.4296998	0	main		NM_010162	Ext1	exostoses (m	NM_010162	RefSeq	Mus muscul	FALSE	14042	Mm.309395	P97464	chr15	GO:000150	ossification	GO:000578	endoplasmic	GO:000837	acetylglucos	O
17223766	187.874	254.5552	0.73804817	-0.4382131	0	main		NM_033563	Klf7	Kruppel-like	NM_033563	RefSeq	Mus muscul	FALSE	93691	Mm.29466	Q99JB0	chr1	GO:000635	transcription	GO:000563	nucleus	GO:000367	nucleic acid	O
17439164	247.1337	338.1787	0.73077843	-0.452494	0	main		NM_001009	Sept11	septin 11	NM_001009	RefSeq	Mus muscul	FALSE	52398	Mm.428571	Q8C1B7	chr5	GO:000704	cell cycle //	GO:000172	stress fiber	GO:000016	nucleotide b	O
17389861	126.2038	173.1994	0.72866188	-0.4566786	0	main		NM_026574	Ino80	INO80 homo	NM_026574	RefSeq	Mus muscul	FALSE	68142	Mm.330496	Q6ZPV2	chr2	GO:000007	mitotic siste	GO:000563	nucleus // m	GO:000016	nucleotide b	O
17357502	136.4818	188.9486	0.72232237	-0.4692853	0	main		NM_001164	Cpsf7	cleavage an	NM_001164	RefSeq	Mus muscul	FALSE	269061	Mm.490260	Q8BTV2	chr19	GO:000639	mRNA proc	GO:000563	nucleus // m	GO:000016	nucleotide b	O
17236754	187.6364	261.0039	0.71890267	-0.4761316	0	main		NM_001001	Eea1	early endos	NM_001001	RefSeq	Mus muscul	FALSE	216238	Mm.490373	Q8BL66	chr10	GO:000689	endocytosis	GO:000573	cytoplasm /	GO:000551	protein bindi	O
17297100	116.5668	162.9167	0.71549939	-0.4829776	0	main		NM_001305	Kctd6	potassium c	NM_001305	RefSeq	Mus muscul	FALSE	71393	Mm.292712	Q8BNL5	chr14	GO:005126	protein hom	GO:000557	cellular_com	GO:003050	ankyrin bind	O
17471541	48.27776	68.38022	0.70601937	-0.5022203	0	main		NM_001309	Clec7a	C-type lectin	NM_001309	RefSeq	Mus muscul	FALSE	56644	Mm.239516	Q6QLQ4	chr6	GO:000222	pattern reco	GO:000588	plasma mem	GO:000551	protein bindi	O
17451526	105.6323	151.2395	0.69844386	-0.5177839	0	main		NM_001159	Kctd10	potassium c	NM_001159	RefSeq	Mus muscul	FALSE	330171	Mm.490304	Q922M3	chr5	GO:005126	protein hom	GO:000563	nucleus // n	GO:000551	protein bindi	O
17346936	45.44438	65.23941	0.69657865	-0.5216418	0	main		NM_001164	Tgif1	TGFB-induc	NM_001164	RefSeq	Mus muscul	FALSE	21815	Mm.101034	P70284	chr17	GO:000012	negative reg	GO:000563	nucleus // n	GO:000097	RNA polyme	O
17219362	233.1581	338.966	0.68785099	-0.539832	0	main		NM_172647	F11r	F11 recepto	NM_172647	RefSeq	Mus muscul	FALSE	16456	Mm.294882	O88792	chr1	GO:000181	regulation o	GO:000588	plasma mem	GO:000551	protein bindi	O
17233194	36.71968	53.4034	0.68759068	-0.5403781	0	main		NM_011376	Sim1	single-mind	NM_011376	RefSeq	Mus muscul	FALSE	20464	Mm.4774	Q61045	chr10	GO:000635	transcription	GO:000563	nucleus // n	GO:000098	RNA polyme	O
17310004	151.8888	221.2115	0.68662253	-0.5424109	0	main		NM_030168	Rictor	RPTOR inde	NM_030168	RefSeq	Mus muscul	FALSE	78757	Mm.275811	Q6QI06	chr15	GO:000193	regulation o	GO:003193	TORC2 com	GO:000551	protein bindi	O
17541618	22.23969	32.47782	0.68476548	-0.5463181	0	main		NM_001310	Mbnl3	muscleblind	NM_001310	RefSeq	Mus muscul	FALSE	171170	Mm.295324	Q8R003	chrX	GO:000639	mRNA proc	GO:000563	nucleus // c	GO:004482	poly(A) RNA	O
17525894	39.92079	58.70789	0.6799902	-0.5564141	0	main		NM_011436	Sorl1	sortilin-relat	NM_011436	RefSeq	Mus muscul	FALSE	20660	Mm.121920	O88307	chr9	GO:000004	protein targe	GO:000557	extracellular	GO:000154	beta-amyloi	O
17401296	86.1347	127.7577	0.67420359	-0.5687438	0	main		NM_172684	Rsbn1	rosbin, roun	NM_172684	RefSeq	Mus muscul	FALSE	229675	Mm.29366	Q80T69	chr3	GO:000815	biological_p	GO:000563	nucleus // n	GO:000551	protein bindi	O
17460405	83.43053	126.2703	0.66072964	-0.597868	0	main		NM_001164	Tia1	cytotoxic gra	NM_001164	RefSeq	Mus muscul	FALSE	21841	Mm.333219	P52912	chr6	GO:000691	apoptotic pr	GO:000563	nucleus // n	GO:000016	nucleotide b	O
17214711	59.94259	91.23893	0.6569848	-0.6060681	0	main		NM_021342	Kcne4	potassium v	NM_021342	RefSeq	Mus muscul	FALSE	57814	Mm.24386	Q9WTW3	chr1	GO:000681	transport //	GO:000807	voltage-gate	GO:000524	voltage-gate	O
17313739	111.0322	169.9508	0.65331967	-0.614139	0	main		NM_146061	Prr5	proline rich 5	NM_146061	RefSeq	Mus muscul	FALSE	109270	Mm.291372	Q812A5	chr15	GO:000193	positive reg	GO:003193	TORC2 com	GO:000509	GTPase act	O
17523861	59.71745	91.97252	0.64929666	-0.6230503	0	main		NM_001291	Birc2	baculoviral I	NM_001291	RefSeq	Mus muscul	FALSE	11797	Mm.335659	Q62210	chr9	GO:000020	protein poly	GO:000174	XY body // r	GO:000371	transcription	O
17309041	41.99436	64.95107	0.64655378	-0.6291577	0	main		NM_001038	Dach1	dachshund 1	NM_001038	RefSeq	Mus muscul	FALSE	13134	Mm.320593	Q9QYB2	chr14	GO:000012	negative reg	GO:000563	nucleus // n	GO:000107	transcription	O
17233960	324.3363	507.3959	0.63921742	-0.6456214	0	main		NM_001242	Jmjd1c	jumonji dom	NM_001242	RefSeq	Mus muscul	FALSE	108829	Mm.23846	Q69ZK6	chr10	GO:000338	epithelial ce	GO:000563	nucleus	GO:000090	sulfonate dio	O
17405285	101.0246	158.7334	0.63644198	-0.6518991	0	main		NM_030595	Nbea	neurobeach	NM_030595	RefSeq	Mus muscul	FALSE	26422	Mm.384353	Q9EPN1	chr3	GO:000660	protein targe	GO:000580	trans-Golgi n	GO:000554	phospholipid	O
17366451	75.1636	119.4984	0.62899252	-0.6688852	0	main		NM_001177	Frmd4a	FERM doma	NM_001177	RefSeq	Mus muscul	FALSE	209630	Mm.37932	Q8BIE6	chr2	GO:009016	establishme	GO:000573	cytoplasm /	GO:003067	protein bindi	O
17289794	67.62027	108.8438	0.62125973	-0.6867315	0	main		NM_152804	Plk2	polo-like kin	NM_152804	RefSeq	Mus muscul	FALSE	20620	Mm.380	P53351	chr13	GO:000008	G1/S transit	GO:000078	chromatin /	GO:000016	nucleotide b	O
17255565	28.55392	50.12595	0.56964347	-0.8118689	0	main		NM_010461	Hoxb8	homeobox B	NM_010461	RefSeq	Mus muscul	FALSE	15416	Mm.4822	P09632	chr11	GO:000635	transcription	GO:000563	nucleus	GO:000367	DNA binding	O
17539271	732.77	1297.645	0.56469219	-0.8244634	0	main		NM_001079	Adgrg2	adhesion G	NM_001079	RefSeq	Mus muscul	FALSE	237175	Mm.213016	Q8CJ12	chrX	GO:000716	signal transd	GO:000588	plasma mem	GO:000487	signal transd	O
17410410	60.24154	123.7928	0.48663202	-1.0390969	0	main	2Down	NM_028943	Sgms2	sphingomye	NM_028943	RefSeq	Mus muscul	FALSE	74442	Mm.273360	Q9D4B1	chr3	GO:000662	lipid metabo	GO:000579	Golgi appar	GO:001630	kinase activi	O
17315718	57.88468	123.6243	0.4682306	-1.0947089	0	main	2Down	NM_001136	Ptger4	prostaglandi	NM_001136	RefSeq	Mus muscul	FALSE	19219	Mm.18509	P32240	chr15	GO:000193	positive reg	GO:000588	plasma mem	GO:000487	signal transd	O
17388774	12.59895	31.74073	0.39693321	-1.3330318	0	main	2Down	NM_007914	Ehf	ets homolog	NM_007914	RefSeq	Mus muscul	FALSE	13661	Mm.10724	O70273	chr2	GO:000635	transcription	GO:000563	nucleus // n	GO:000097	RNA polyme	O

Supplementary Table 4. The comparison of global mRNA expression in epididymal adipose tissues of *Mir342* (-/-) and *Mir342* (+/+) mice fed with high fat-high sucrose (HFHS) chow.

	Sample name	Negative control (average)
Test	HFHS *Mir342* (-/-) 682	41.96
Control	HFHS *Mir342* (+/+) 696	42.10

2Up	Ratio ≧ 2 and<br>Expression value of test sample ≧ Negative control (average)
2Down	Ratio ≦ 0.5 and<br>Expression value of test sample ≧ Negative control (average)

Feature ID	Normalized data				Flag			Annotation																	Keyword
Probe Set II	Expression v	Expression v	Ratio	Log2Ratio	Flag	category	Ratio flag	Gene Acces	Gene Symb	Gene Descr	mRNA Acce	mRNA Sou	mRna - Des	mRNA - xhy	EntrezGene	UniGene ID	SwissProt a	Chromosom	GO Biologic	GO Biologic	GO Cellular	GO Cellular	GO Molecul	GO Molecul	miR342 target gene
17376822	115.7549	38.63621	2.99602109	1.58304778	0	main	2Up	NM_001291	Snap25	synaptosom	NM_001291	RefSeq	Mus muscul	FALSE	20614	Mm.45953	P60879	chr2	GO:000688	exocytosis	GO:000573	cytoplasm /	GO:000014	SNARE bin	O
17262964	37.45129	23.3643	1.60292797	0.6807096	0	main		NM_001029	Fat2	FAT tumor s	NM_001029	RefSeq	Mus muscul	FALSE	245827	Mm.339363	Q5F226	chr11	GO:000715	cell adhesio	GO:000563	nucleus // p	GO:000550	calcium ion	O
17441134	16.16738	10.695	1.51167648	0.59614942	0	main		XM_006530	Msi1	musashi RN	XM_006530	RefSeq	PREDICTEI	FALSE	17690	Mm.5077	Q61474	chr5	GO:000972	response to	GO:000563	nucleus // c	GO:000016	nucleotide b	O
17395766	53.33025	35.9902	1.48179921	0.56734997	0	main		NM_007906	Eef1a2	eukaryotic tr	NM_007906	RefSeq	Mus muscul	FALSE	13628	Mm.2645	P62631	chr2	GO:000641	translation /	GO:000563	nucleus // c	GO:000016	nucleotide b	O
17399817	55.01883	37.35225	1.47297231	0.55873031	0	main		NM_199422	S100a7a	S100 calciu	NM_199422	RefSeq	Mus muscul	FALSE	381493	Mm.291525	Q6S5I3	chr3	GO:000695	inflammator	GO:000561	extracellular	GO:000550	calcium ion	O
17490995	35.71547	25.4631	1.40263636	0.48814104	0	main		NM_008172	Grin2d	glutamate re	NM_008172	RefSeq	Mus muscul	FALSE	14814	Mm.322594	Q03391	chr7	GO:000196	startle respo	GO:000588	plasma men	GO:000487	receptor act	O
17337919	33.33521	24.07984	1.38436177	0.46922101	0	main		NM_013688	Tcte1	t-complex-a	NM_013688	RefSeq	Mus muscul	FALSE	21645	Mm.491193	A6H639	chr17	GO:000815	biological_p	GO:000557	cellular_com	GO:000367	molecular_f	O
17377498	509.2241	371.3322	1.37134377	0.45559028	0	main		NM_153781	Pygb	brain glycog	NM_153781	RefSeq	Mus muscul	FALSE	110078	Mm.222584	Q8CI94	chr2	GO:000597	carbohydrat	GO:000573	cytoplasm /	GO:000382	catalytic act	O
17519649	200.4656	148.765	1.34753201	0.43031955	0	main		NM_010357	Gsta4	glutathione S	NM_010357	RefSeq	Mus muscul	FALSE	14860	Mm.2662	P24472	chr9	GO:000815	metabolic pr	GO:000573	cytoplasm /	GO:000436	glutathione	O
17280431	50.31095	37.39505	1.34539063	0.42802512	0	main		NM_009417	Tpo	thyroid pero	NM_009417	RefSeq	Mus muscul	FALSE	22018	Mm.4991	P35419	chr12	GO:000659	thyroid horm	GO:000561	extracellular	GO:000444	iodide pero	O
17372066	31.72515	23.66475	1.34060787	0.4228873	0	main		NM_013555	Hoxd9	homeobox D	NM_013555	RefSeq	Mus muscul	FALSE	15438	Mm.26544	P28357	chr2	GO:000012	negative reg	GO:000563	nucleus // n	GO:000097	RNA polym	O
17525198	35.91421	26.96492	1.33188639	0.41347103	0	main		NM_172290	Ntm	neurotrimin	NM_172290	RefSeq	Mus muscul	FALSE	235106	Mm.283138	Q99PJ0	chr9	GO:000715	cell adhesio	GO:000561	extracellular	---	---	O
17227384	28.00611	21.27916	1.31612855	0.39630041	0	main		NM_001313	Pkp1	plakophilin 1	NM_001313	RefSeq	Mus muscul	FALSE	18772	Mm.4494	P97350	chr1	GO:000715	cell adhesio	GO:000563	nucleus // n	GO:000551	protein bind	O
17431910	41.61295	31.9076	1.30417048	0.38313247	0	main		NM_011039	Pax7	paired box 7	NM_011039	RefSeq	Mus muscul	FALSE	18509	Mm.218760	P47239	chr4	GO:000633	chromatin re	GO:000563	nucleus // tr	GO:000098	transcription	O
17219127	22.30407	17.12559	1.30238257	0.3811533	0	main		NM_033652	Lmx1a	LIM homeob	NM_033652	RefSeq	Mus muscul	FALSE	110648	Mm.330081	Q9JKU8	chr1	GO:000155	regulation o	GO:000563	nucleus	GO:000367	DNA binding	O
17224551	32.20582	24.97091	1.28973353	0.36707303	0	main		NM_008985	Ptprn	protein tyros	NM_008985	RefSeq	Mus muscul	FALSE	19275	Mm.2902	Q60673	chr1	GO:000030	response to	GO:000576	endosome /	GO:000472	protein tyros	O
17400072	43.9936	34.16462	1.2876947	0.36479058	0	main		NM_028307	Tdrkh	tudor and KI	NM_028307	RefSeq	Mus muscul	FALSE	72634	Mm.331198	Q80VL1	chr3	GO:000714	male meiosi	GO:000573	cytoplasm /	GO:000367	nucleic acid	O
17494868	23.54035	18.42623	1.27754565	0.35337484	0	main		NM_001170	Trim66	tripartite mo	NM_001170	RefSeq	Mus muscul	FALSE	330627	Mm.183528	Q924W6	chr7	GO:004589	negative reg	GO:000562	intracellular	GO:000368	chromatin b	O
17452311	55.55238	43.6625	1.27231331	0.34745398	0	main		NM_001312	Cux2	cut-like hom	NM_001312	RefSeq	Mus muscul	FALSE	13048	Mm.332661	P70298	chr5	GO:000012	negative reg	GO:000563	nucleus // n	GO:000097	RNA polym	O
17438903	33.7138	26.51221	1.27163296	0.34668231	0	main		NM_007423	Afp	alpha fetopr	NM_007423	RefSeq	Mus muscul	FALSE	11576	Mm.358570	P02772	chr5	GO:000154	ovulation fro	GO:000557	extracellular	GO:000456	beta-glucur	O
17465332	13.43122	10.57571	1.27000646	0.34483583	0	main		NM_138682	Lrrc4	leucine rich	NM_138682	RefSeq	Mus muscul	FALSE	192198	Mm.443660	Q99PH1	chr6	GO:005080	regulation o	GO:000588	plasma men	GO:000551	protein bind	O
17349774	65.17008	53.43423	1.21963169	0.28644554	0	main		NM_001305	Hars2	histidyl-tRN	NM_001305	RefSeq	Mus muscul	FALSE	70791	Mm.282700	Q99KK9	chr18	GO:000641	translation /	GO:000573	cytoplasm /	GO:000016	nucleotide b	O
17392529	37.18504	30.51064	1.21875647	0.28540988	0	main		NM_001077	Nkx2-2	NK2 homeo	NM_001077	RefSeq	Mus muscul	FALSE	18088	Mm.330639	P42586	chr2	GO:000332	type B panc	GO:000562	intracellular	GO:000098	RNA polym	O
17424880	22.06554	18.12428	1.21745747	0.28387137	0	main		XM_006537	Pax5	paired box 5	XM_006537	RefSeq	PREDICTEI	FALSE	18507	Mm.439659	Q02650	chr4	GO:000012	negative reg	GO:000563	nucleus // tr	GO:000097	RNA polym	O
17414913	9.651809	7.986125	1.20857224	0.27330371	0	main		NM_001163	Frmd3	FERM doma	NM_001163	RefSeq	Mus muscul	FALSE	242506	Mm.221597	Q8BHD4	chr4	GO:000815	biological_p	GO:000573	cytoplasm /	GO:000809	cytoskeletal	O
17428501	31.41215	26.00614	1.20787437	0.27247041	0	main		NM_001025	Dmbx1	diencephalo	NM_001025	RefSeq	Mus muscul	FALSE	140477	Mm.213206	Q91ZK4	chr4	GO:000012	negative reg	GO:000563	nucleus // tr	GO:000097	RNA polym	O
17542047	26.49915	21.99744	1.204647	0.26861045	0	main		NM_178740	Slitrk4	SLIT and NT	NM_178740	RefSeq	Mus muscul	FALSE	245446	Mm.101707	Q810B8	chrX	GO:000740	axonogenes	GO:001602	membrane	GO:000367	molecular_f	O
17465296	10.88672	9.068828	1.20045501	0.26358134	0	main		NR_030420	Mir592	microRNA 5	NR_030420	RefSeq	Mus muscul	FALSE	735266	---	P47743	chr6	---	---	---	---	---	---	O
17400521	27.80565	23.16278	1.20044528	0.26356965	0	main		NM_022030	Sv2a	synaptic ves	NM_022030	RefSeq	Mus muscul	FALSE	64051	Mm.491135	Q9JIS5	chr3	GO:000681	transport //	GO:000573	cytoplasm /	GO:000487	receptor act	O
17284839	47.06473	39.28789	1.19794496	0.26056163	0	main		NM_177290	Itgb8	integrin beta	NM_177290	RefSeq	Mus muscul	FALSE	320910	Mm.217000	Q0VBD0	chr12	GO:000157	ganglioside	GO:000998	cell surface	GO:000510	receptor bin	O
17377126	83.47684	69.82295	1.19555017	0.25767468	0	main		NM_025314	Dtd1	D-tyrosyl-tR	NM_025314	RefSeq	Mus muscul	FALSE	66044	Mm.28109	Q9DD18	chr2	GO:000626	DNA replica	GO:000563	nucleus // c	GO:000216	aminoacyl-t	O
17323532	40.185	33.64933	1.19422883	0.2560793	0	main		NM_001159	P2rx6	purinergic re	NM_001159	RefSeq	Mus muscul	FALSE	18440	Mm.8032	O54803	chr16	GO:000681	transport //	GO:000563	integral com	GO:000161	purinergic n	O
17482021	1435.672	1209.146	1.18734379	0.24773773	0	main		NM_011055	Pde3b	phosphodie	NM_011055	RefSeq	Mus muscul	FALSE	18576	Mm.430730	Q61409	chr7	GO:000152	angiogenesi	GO:000578	endoplasmic	GO:000411	3',5'-cyclic-r	O
17392578	40.12033	33.94569	1.18189761	0.24110506	0	main		NM_019632	Napb	N-ethylmale	NM_019632	RefSeq	Mus muscul	FALSE	17957	Mm.274308	P28663	chr2	GO:000681	transport //	GO:000577	vacuolar me	GO:000548	soluble NSF	O
17417290	126.7323	107.5645	1.1781982	0.23658225	0	main		NM_181040	Atpaf1	ATP syntha	NM_181040	RefSeq	Mus muscul	FALSE	230649	Mm.109600	Q811I0	chr4	GO:000646	protein com	GO:000573	mitochondri	GO:000367	molecular_f	O
17290111	90.00959	76.51958	1.17629488	0.23424977	0	main		NM_008002	Fgf10	fibroblast gr	NM_008002	RefSeq	Mus muscul	FALSE	14165	Mm.317323	O35565	chr13	GO:000013	establishme	GO:000557	extracellular	GO:000510	receptor bin	O
17415943	96.70791	82.25693	1.175681	0.23349667	0	main		NM_001164	Dnajc6	DnaJ (Hsp4	NM_001164	RefSeq	Mus muscul	FALSE	72685	Mm.76494	Q80TZ3	chr4	GO:007231	clathrin coat	GO:004520	synapse //	GO:000472	phosphopro	O
17456627	82.29111	70.0621	1.1745453	0.23210235	0	main		NM_001081	Flnc	filamin C, ga	NM_001081	RefSeq	Mus muscul	FALSE	68794	Mm.39046	Q8VHX6	chr6	GO:003002	actin filamer	GO:000573	cytoplasm /	GO:000377	actin binding	O
17523861	66.82164	57.18039	1.16861113	0.22479493	0	main		NM_001291	Birc2	baculoviral I	NM_001291	RefSeq	Mus muscul	FALSE	11797	Mm.335659	Q62210	chr9	GO:000020	protein poly	GO:000174	XY body //	GO:000371	transcription	O
17523852	143.28	122.6337	1.16835747	0.22448175	0	main		NM_001291	Birc2	baculoviral I	NM_001291	RefSeq	Mus muscul	FALSE	11797	Mm.335659	Q62210	chr9	GO:000020	protein poly	GO:000174	XY body //	GO:000371	transcription	O
17402787	26.86041	23.20444	1.15755476	0.21108045	0	main		NM_021382	Tacr3	tachykinin re	NM_021382	RefSeq	Mus muscul	FALSE	21338	Mm.103810	P47937	chr3	GO:000716	signal trans	GO:000563	nucleus // c	GO:000487	signal trans	O
17339108	447.2206	388.2724	1.15182176	0.20391749	0	main		NM_008549	Man2a1	mannosidas	NM_008549	RefSeq	Mus muscul	FALSE	17158	Mm.2433	P27046	chr17	GO:000170	in utero emb	GO:000013	Golgi memb	GO:000382	catalytic act	O
17537643	34.36943	29.89347	1.14973036	0.20129555	0	main		NM_026239	Tmem35	transmembr	NM_026239	RefSeq	Mus muscul	FALSE	67564	Mm.41489	Q9D328	chrX	GO:000815	biological_p	GO:000577	peroxisome	GO:000367	molecular_f	O
17531154	33.77011	29.37538	1.14960589	0.20113936	0	main		NM_007567	Bsn	bassoon	NM_007567	RefSeq	Mus muscul	FALSE	12217	Mm.20425	O88737	chr9	GO:000741	synapse ass	GO:000573	cytoplasm /	GO:000551	protein bind	O
17388774	14.99036	13.06371	1.14748108	0.19847036	0	main		NM_007914	Ehf	ets homolog	NM_007914	RefSeq	Mus muscul	FALSE	13661	Mm.10724	O70273	chr2	GO:000635	transcription	GO:000563	nucleus // n	GO:000097	RNA polym	O
17394024	44.12214	38.52722	1.14521993	0.19562468	0	main		XM_006500	Oser1	oxidative str	XM_006500	RefSeq	PREDICTEI	FALSE	66680	Mm.25119	Q9D722	chr2	GO:007030	cellular resp	GO:000557	cellular_com	GO:000367	molecular_f	O
17289372	364.7119	318.5246	1.14500387	0.19535248	0	main		NM_007930	Enc1	ectodermal-	NM_007930	RefSeq	Mus muscul	FALSE	13803	Mm.241073	O35709	chr13	GO:000727	multicellular	GO:000079	nuclear chro	GO:000377	actin binding	O
17509315	36.70605	32.15852	1.1414098	0.19081686	0	main		NM_001145	Tenm3	teneurin trar	NM_001145	RefSeq	Mus muscul	FALSE	23965	Mm.42191	Q9WTS6	chr8	GO:000715	cell adhesio	GO:000588	integral com	GO:004280	protein hom	O
17321326	131.7579	116.0284	1.13556595	0.18341149	0	main		NM_007478	Arf3	ADP-ribosyl	NM_007478	RefSeq	Mus muscul	FALSE	11842	Mm.221298	P61205	chr15	GO:000681	transport //	GO:000562	intracellular	GO:000016	nucleotide b	O
17423701	16.44472	14.4964	1.13440026	0.18192977	0	main		NM_010243	Fut9	fucosyltrans	NM_010243	RefSeq	Mus muscul	FALSE	14348	Mm.39101	O88819	chr4	GO:000648	protein glyco	GO:000579	Golgi appara	GO:000000	alpha-1,6-m	O
17287081	56.04614	49.43338	1.13377115	0.18112946	0	main		NM_031166	Id4	inhibitor of D	NM_031166	RefSeq	Mus muscul	FALSE	15904	Mm.458006	P41139	chr13	GO:000008	G1/S transit	GO:000563	nucleus // c	GO:000108	RNA polym	O
17451536	123.9908	110.1686	1.12546406	0.17051999	0	main		NM_029956	Mmab	methylmalor	NM_029956	RefSeq	Mus muscul	FALSE	77697	Mm.105182	Q9D273	chr5	GO:000923	cobalamin m	GO:000573	mitochondri	GO:000016	nucleotide b	O
17427164	40.78498	36.25897	1.12482456	0.1697	0	main		NM_001177	Elavl2	ELAV (embr	NM_001177	RefSeq	Mus muscul	FALSE	15569	Mm.318042	Q60899	chr4	---	---	---	---	GO:000016	nucleotide b	O
17233960	284.576	253.8845	1.12088765	0.16464168	0	main		NM_001242	Jmjd1c	jumonji dom	NM_001242	RefSeq	Mus muscul	FALSE	108829	Mm.23846	Q69ZK6	chr10	GO:000338	epithelial ce	GO:000563	nucleus	GO:000090	sulfonate di	O
17492861	85.35543	76.19315	1.1202507	0.16382163	0	main		NM_013886	Hdgfrp3	hepatoma-d	NM_013886	RefSeq	Mus muscul	FALSE	29877	Mm.392934	Q9JMG7	chr7	GO:000828	cell prolifera	GO:000563	nucleus // n	GO:000368	chromatin b	O
17448693	22.4792	20.07113	1.1199768	0.16346885	0	main		NM_001122	Txk	TXK tyrosine	NM_001122	RefSeq	Mus muscul	FALSE	22165	Mm.3264	P42682	chr5	GO:000181	cytokine pro	GO:000563	nucleus // c	GO:000016	nucleotide b	O
17447510	217.7841	194.5101	1.11965445	0.16305356	0	main		NM_026242	Mrfap1	Morf4 family	NM_026242	RefSeq	Mus muscul	FALSE	67568	Mm.490257	Q9CQL7	chr5	GO:000815	biological_p	GO:000563	nucleus // n	GO:000551	protein bind	O
17454157	75.46747	67.59332	1.11649302	0.15897423	0	main		NM_144913	Mepce	methylphos	NM_144913	RefSeq	Mus muscul	FALSE	231803	Mm.61193	Q8K3A9	chr5	GO:000012	negative reg	GO:000557	cellular_com	GO:000017	rRNA (aden	O
17511868	222.1603	199.3775	1.11426966	0.15609842	0	main		NM_028221	Fam192a	family with s	NM_028221	RefSeq	Mus muscul	FALSE	102122	Mm.250425	Q91WE2	chr8	GO:000815	biological_p	GO:000563	nucleus // n	GO:000367	molecular_f	O
17466798	41.48066	37.22743	1.1142499	0.15607283	0	main		NM_001311	Hoxa1	homeobox A	NM_001311	RefSeq	Mus muscul	FALSE	15394	Mm.197	P09022	chr6	GO:000635	transcription	GO:000563	nucleus	GO:000367	DNA binding	O
17539271	373.0527	335.2821	1.1126532	0.15400399	0	main		NM_001079	Adgrg2	adhesion G	NM_001079	RefSeq	Mus muscul	FALSE	237175	Mm.213016	Q8CJ12	chrX	GO:000716	signal trans	GO:000588	plasma men	GO:000487	signal trans	O
17382496	54.55309	49.19826	1.10884186	0.14905362	0	main		NM_001177	Grin1	glutamate re	NM_001177	RefSeq	Mus muscul	FALSE	14810	Mm.278672	P35438	chr2	GO:000166	conditioned	GO:000573	cytoplasm /	GO:000487	receptor act	O
17328792	181.6631	163.925	1.10820863	0.14822951	0	main		NM_001109	Dgcr2	DiGeorge sy	NM_001109	RefSeq	Mus muscul	FALSE	13356	Mm.254515	P98154	chr16	GO:000715	cell adhesio	GO:001602	membrane	GO:003024	carbohydrat	O
17536501	51.28286	46.371	1.10592526	0.14525388	0	main		NM_001177	Eda	ectodysplas	NM_001177	RefSeq	Mus muscul	FALSE	13607	Mm.328086	O54693	chrX	GO:000194	hair follicle c	GO:000557	extracellular	GO:000516	tumor necro	O
17344472	88.49494	80.75047	1.09590619	0.13212431	0	main		NM_001142	Atat1	alpha tubulin	NM_001142	RefSeq	Mus muscul	FALSE	73242	Mm.273155	Q8K341	chr17	GO:000728	spermatoge	GO:000573	cytoplasm /	GO:001674	transferase	O
17349590	262.7926	240.1774	1.0941604	0.12982425	0	main		NM_019912	Ube2d2a	ubiquitin-cor	NM_019912	RefSeq	Mus muscul	FALSE	56550	Mm.180052	P62838	chr18	GO:000020	protein poly	GO:000015	ubiquitin liga	GO:000016	nucleotide b	O
17424884	22.16889	20.29303	1.09243864	0.12755224	0	main		NM_008782	Pax5	paired box 5	NM_008782	RefSeq	Mus muscul	FALSE	18507	Mm.439659	Q02650	chr4	GO:000012	negative reg	GO:000563	nucleus // tr	GO:000097	RNA polym	O
17466982	85.6805	78.46453	1.09196474	0.12692627	0	main		NM_027268	Scrn1	secernin 1	NM_027268	RefSeq	Mus muscul	FALSE	69938	Mm.41557	Q9CZC8	chr6	GO:000650	proteolysis	GO:000563	nucleus // c	GO:001680	dipeptidase	O
17495340	107.2381	98.28793	1.09106072	0.1257314	0	main		NM_001025	Sox6	SRY (sex de	NM_001025	RefSeq	Mus muscul	FALSE	20679	Mm.323365	P40645	chr7	GO:000012	negative reg	GO:000563	nucleus // n	GO:000367	DNA binding	O
17414802	41.45781	38.02921	1.09015701	0.12453594	0	main		NM_021362	Pappa	pregnancy-a	NM_021362	RefSeq	Mus muscul	FALSE	18491	Mm.317854	Q8R4K8	chr4	GO:000650	proteolysis	GO:000557	extracellular	GO:000417	endopeptida	O
17396765	24.35615	22.47008	1.08393695	0.11628084	0	main		NM_001013	Usp13	ubiquitin spe	NM_001013	RefSeq	Mus muscul	FALSE	72607	Mm.316153	Q5BKP2	chr3	GO:000635	regulation o	GO:000576	lysosome //	GO:000419	cysteine-typ	O
17518616	136.4556	125.8959	1.08387644	0.1162003	0	main		NM_028030	Rbpms2	RNA binding	NM_028030	RefSeq	Mus muscul	FALSE	71973	Mm.29148	Q8VC52	chr9	GO:000815	biological_p	GO:000557	cellular_com	GO:000016	nucleotide b	O
17363704	71.97082	66.40451	1.08382428	0.11613087	0	main		NM_001166	Rfx3	regulatory fa	NM_001166	RefSeq	Mus muscul	FALSE	19726	Mm.313321	P48381	chr19	GO:000635	transcription	GO:000079	nuclear chro	GO:000097	RNA polym	O
17223412	162.3734	149.894	1.08325483	0.11537267	0	main		NM_001310	Fam126b	family with s	NM_001310	RefSeq	Mus muscul	FALSE	213056	Mm.26272	Q8C729	chr1	GO:000815	biological_p	GO:000562	intracellular	GO:000367	molecular_f	O
17246577	62.48776	57.69034	1.08315812	0.11524386	0	main		NM_178149	Pik3ip1	phosphoinos	NM_178149	RefSeq	Mus muscul	FALSE	216505	Mm.390323	Q7TMJ8	chr11	GO:001406	negative reg	GO:000588	plasma men	GO:003631	phosphatidy	O
17417437	228.6117	211.4744	1.08103723	0.11241621	0	main		NM_029868	Gpbp1l1	GC-rich pro	NM_029868	RefSeq	Mus muscul	FALSE	77110	Mm.41715	Q6NZP2	chr4	GO:000635	transcription	GO:000563	nucleus // n	GO:000367	DNA binding	O
17448467	57.584	53.34187	1.07952721	0.11039961	0	main		NM_001164	Bend4	BEN domain	NM_001164	RefSeq	Mus muscul	FALSE	666938	Mm.101339	P86174	chr5	GO:000815	biological_p	GO:000557	cellular_com	GO:000367	molecular_f	O
17287613	331.9958	307.956	1.07806245	0.10844075	0	main		NM_008739	Nsd1	nuclear rece	NM_008739	RefSeq	Mus muscul	FALSE	18193	Mm.168965	O88491	chr13	GO:000012	negative reg	GO:000563	nucleus // n	GO:000097	RNA polym	O
17526382	285.2023	265.1712	1.07554025	0.10506152	0	main		NM_001081	Kmt2a	lysine (K)-sp	NM_001081	RefSeq	Mus muscul	FALSE	214162	Mm.2389	P55200	chr9	GO:000630	DNA methyl	GO:000563	nucleus // n	GO:000104	core promot	O
17253215	40.21035	37.5668	1.07036932	0.09810866	0	main		NM_010484	Slc6a4	solute carrie	NM_010484	RefSeq	Mus muscul	FALSE	15567	Mm.300318	Q60857	chr11	GO:000681	transport //	GO:000576	endosome /	GO:000532	neurotransm	O
17309001	28.28406	26.61485	1.06271724	0.08775779	0	main		NM_178685	Pcdh20	protocadher	NM_178685	RefSeq	Mus muscul	FALSE	219257	Mm.128861	Q8BIZ0	chr14	GO:000715	cell adhesio	GO:000588	plasma men	GO:000550	calcium ion	O
17223985	47.3078	44.56887	1.06145388	0.08604169	0	main		NM_011770	Ikzf2	IKAROS fan	NM_011770	RefSeq	Mus muscul	FALSE	22779	Mm.106343	P81183	chr1	GO:000635	transcription	GO:000563	nucleus // n	GO:000367	nucleic acid	O
17353765	149.9401	141.6387	1.05860969	0.08217076	0	main		NM_008214	Hars	histidyl-tRN	NM_008214	RefSeq	Mus muscul	FALSE	15115	Mm.10528	Q61035	chr18	GO:000641	translation /	GO:000573	cytoplasm /	GO:000016	nucleotide b	O
17545121	18.97492	17.9251	1.05856704	0.08211264	0	main		NM_001110	Dcx	doublecortin	NM_001110	RefSeq	Mus muscul	FALSE	13193	Mm.12871	O88809	chrX	GO:000176	neuron migr	GO:000573	cytoplasm /	GO:000468	calmodulin-	O
17457892	112.6965	106.5039	1.05814435	0.08153645	0	main		NM_007610	Casp2	caspase 2	NM_007610	RefSeq	Mus muscul	FALSE	12366	Mm.3921	P29594	chr6	GO:000650	proteolysis	GO:000563	nucleus // c	GO:000419	cysteine-typ	O
17371148	11.0231	10.41962	1.05791766	0.08122734	0	main		NM_009322	Tbr1	T-box brain	NM_009322	RefSeq	Mus muscul	FALSE	21375	Mm.308525	Q64336	chr2	GO:000166	conditioned	GO:000563	nucleus // n	GO:000097	RNA polym	O
17219612	53.80737	50.86245	1.05789969	0.08120284	0	main		NM_177723	Vsig8	V-set and im	NM_177723	RefSeq	Mus muscul	FALSE	240916	Mm.71940	Q6P3A4	chr1	GO:000815	biological_p	GO:000562	intracellular	GO:004482	poly(A) RN	O
17303757	51.99693	49.19018	1.05705915	0.08005611	0	main		NM_134081	Dnajc9	DnaJ (Hsp4	NM_134081	RefSeq	Mus muscul	FALSE	108671	Mm.2871	Q91WN1	chr14	GO:003278	positive reg	GO:000561	extracellular	GO:003107	heat shock	O
17543290	81.08113	76.80655	1.05565385	0.07813685	0	main		NM_001033	Arhgef9	CDC42 guar	NM_001033	RefSeq	Mus muscul	FALSE	236915	Mm.44841	Q3UTH8	chrX	GO:000726	small GTPa	GO:000573	cytoplasm /	GO:000508	guanyl-nucl	O
17343735	172.9278	163.8833	1.05518866	0.07750097	0	main		NM_013543	H2-Ke6	H2-K region	NM_013543	RefSeq	Mus muscul	FALSE	14979	Mm.275452	P50171	chr17	GO:000662	lipid metabo	GO:000573	mitochondri	GO:000025	C-3 sterol d	O
17217127	247.4617	234.5643	1.0549845	0.0772218	0	main		NM_001145	Nucks1	nuclear case	NM_001145	RefSeq	Mus muscul	FALSE	98415	Mm.246869	Q80XU3	chr1	GO:004259	glucose hon	GO:000563	nucleus // n	GO:000107	transcription	O
17325671	29.92644	28.37711	1.05459788	0.076693	0	main		NM_175548	Lsamp	limbic syster	NM_175548	RefSeq	Mus muscul	FALSE	268890	Mm.310524	Q8BLK3	chr16	GO:000715	cell adhesio	GO:000588	plasma men	---	---	O
17472089	32.27926	30.72753	1.05049967	0.0710757	0	main		NM_008171	Grin2b	glutamate re	NM_008171	RefSeq	Mus muscul	FALSE	14812	Mm.436649	Q01097	chr6	GO:000150	action poter	GO:000588	plasma men	GO:000487	receptor act	O
17281388	57.13224	54.38596	1.05049612	0.07107083	0	main		NM_001033	Fbxo33	F-box protei	NM_001033	RefSeq	Mus muscul	FALSE	70611	Mm.311026	Q8VE08	chr12	GO:000815	biological_p	GO:000557	cellular_com	GO:000367	molecular_f	O
17223666	370.1803	352.6698	1.04965126	0.06991008	0	main		NM_001081	Ino80d	INO80 comp	NM_001081	RefSeq	Mus muscul	FALSE	227195	Mm.74718	Q66JY2	chr1	GO:000628	DNA repair	GO:000563	nucleus // c	GO:000367	molecular_f	O
17248747	18.22918	17.36692	1.04964956	0.06990775	0	main		NM_173384	Sox30	SRY (sex de	NM_173384	RefSeq	Mus muscul	FALSE	214105	Mm.66708	Q8CGW4	chr11	GO:000635	transcription	GO:000563	nucleus // n	GO:000367	DNA binding	O
17265657	67.69852	64.65639	1.04705072	0.06633134	0	main		NM_177776	Smtnl2	smoothelin-l	NM_177776	RefSeq	Mus muscul	FALSE	276829	Mm.46732	Q8CI12	chr11	GO:000815	biological_p	GO:000557	cellular_com	GO:000367	molecular_f	O
17294267	25.37928	24.23967	1.04701425	0.06628108	0	main		NM_028878	Slc6a19	solute carrie	NM_028878	RefSeq	Mus muscul	FALSE	74338	Mm.271635	Q9D687	chr13	GO:000681	transport //	GO:000588	plasma men	GO:000532	neurotransm	O
17373120	199.2105	191.0578	1.04267138	0.06028453	0	main		NM_001244	Celf1	CUGBP, Ela	NM_001244	RefSeq	Mus muscul	FALSE	13046	Mm.29495	P28659	chr2	GO:000637	mRNA splic	GO:000563	nucleus // n	GO:000016	nucleotide b	O
17489707	234.0865	224.509	1.04265976	0.06026846	0	main		NM_009884	Cebpg	CCAAT/enh	NM_009884	RefSeq	Mus muscul	FALSE	12611	Mm.273090	P53568	chr7	GO:000633	nucleosome	GO:000563	nucleus // n	GO:000097	RNA polym	O
17405397	44.2464	42.52287	1.04053184	0.05732111	0	main		NM_019410	Pfn2	profilin 2	NM_019410	RefSeq	Mus muscul	FALSE	18645	Mm.271744	Q9JJV2	chr3	GO:001063	negative reg	GO:000573	cytoplasm /	GO:000077	adenyl-nucl	O
17441140	30.30204	29.12252	1.04050199	0.05727972	0	main		NM_008629	Msi1	musashi RN	NM_008629	RefSeq	Mus muscul	FALSE	17690	Mm.5077	Q61474	chr5	GO:000972	response to	GO:000563	nucleus // c	GO:000016	nucleotide b	O
17533416	341.3517	328.552	1.03895791	0.05513721	0	main		NM_010028	Ddx3x	DEAD/H (As	NM_010028	RefSeq	Mus muscul	FALSE	13205	Mm.289662	Q62167	chrX	GO:000237	immune sys	GO:000563	nucleus // c	GO:000016	nucleotide b	O
17297100	82.67797	79.65768	1.03791587	0.0536895	0	main		NM_001305	Kctd6	potassium c	NM_001305	RefSeq	Mus muscul	FALSE	71393	Mm.292712	Q8BNL5	chr14	GO:005126	protein hom	GO:000557	cellular_com	GO:003050	ankyrin binc	O
17401296	92.50517	89.3961	1.03477859	0.0493221	0	main		NM_172684	Rsbn1	rosbin, roun	NM_172684	RefSeq	Mus muscul	FALSE	229675	Mm.29366	Q80T69	chr3	GO:000815	biological_p	GO:000563	nucleus // n	GO:000551	protein bind	O
17502754	278.6718	269.5221	1.03394787	0.04816344	0	main		NM_001081	Otud4	OTU domain	NM_001081	RefSeq	Mus muscul	FALSE	73945	Mm.34348	B2RRE7	chr8	GO:000650	proteolysis	GO:000557	cellular_com	GO:000484	ubiquitin-sp	O
17539888	59.46204	57.53815	1.03343677	0.04745012	0	main		NM_018832	Magix	MAGI family	NM_018832	RefSeq	Mus muscul	FALSE	54634	Mm.67912	Q4KL35	chrX	GO:000815	biological_p	GO:000557	cellular_com	GO:000367	molecular_f	O
17525894	82.84209	80.42152	1.03009854	0.04278235	0	main		NM_011436	Sorl1	sortilin-relat	NM_011436	RefSeq	Mus muscul	FALSE	20660	Mm.121920	O88307	chr9	GO:000004	protein targe	GO:000557	extracellular	GO:000154	beta-amyloi	O
17430775	8.021109	7.78703	1.03006011	0.04272853	0	main		NR_036572	Rps15a-ps4	ribosomal pr	NR_036572	RefSeq	Mus muscul	FALSE	664903	Mm.490762	Q9JL60	chr4	---	---	---	---	---	---	O
17381475	231.4808	225.4937	1.02655107	0.03780541	0	main		NM_001110	Celf2	CUGBP, Ela	NM_001110	RefSeq	Mus muscul	FALSE	14007	Mm.398543	Q9Z0H4	chr2	GO:000637	mRNA splic	GO:000563	nucleus // n	GO:000016	nucleotide b	O
17510013	90.50267	88.38013	1.02401603	0.0342383	0	main		NM_001122	Upf1	UPF1 regula	NM_001122	RefSeq	Mus muscul	FALSE	19704	Mm.390048	Q9EPU0	chr8	GO:000018	nuclear-tran	GO:000078	chromatin /	GO:000016	nucleotide b	O
17224291	81.55163	79.75684	1.02250327	0.03210546	0	main		NM_001310	Usp37	ubiquitin spe	NM_001310	RefSeq	Mus muscul	FALSE	319651	Mm.66568	Q8C0R0	chr1	GO:000008	G1/S transit	GO:000563	nucleus // n	GO:000419	cysteine-typ	O
17442970	290.1595	283.8113	1.02236768	0.03191413	0	main		NM_001001	Vkorc1l1	vitamin K ep	NM_001001	RefSeq	Mus muscul	FALSE	69568	Mm.288718	Q6TEK5	chr5	GO:001718	peptidyl-glut	GO:000578	endoplasmic	GO:001649	oxidoreduct	O
17379582	156.3304	152.9911	1.02182676	0.03115062	0	main		NM_146129	Pcif1	PDX1 C-tern	NM_146129	RefSeq	Mus muscul	FALSE	228866	Mm.241134	P59114	chr2	GO:001092	negative reg	GO:000563	nucleus // n	GO:000367	molecular_f	O
17537756	169.9344	166.7148	1.01931202	0.02759574	0	main		NM_027870	Armcx3	armadillo re	NM_027870	RefSeq	Mus muscul	FALSE	71703	Mm.67949	Q8BHS6	chrX	GO:003461	cellular prot	GO:001602	membrane	GO:000551	protein bind	O
17323659	101.8101	100.0878	1.01720789	0.02461456	0	main		NM_001080	Trmt2a	TRM2 tRNA	NM_001080	RefSeq	Mus muscul	FALSE	15547	Mm.271798	Q8BNV1	chr16	GO:000639	RNA proces	---	---	GO:000016	nucleotide b	O
17287066	261.8475	257.7346	1.01595789	0.0228406	0	main		NM_001170	Rnf144b	ring finger p	NM_001170	RefSeq	Mus muscul	FALSE	218215	Mm.287609	Q8BKD6	chr13	GO:000020	protein poly	GO:000015	ubiquitin liga	GO:000484	ubiquitin-pr	O
17241454	198.1283	195.0712	1.01567171	0.02243417	0	main		NM_053183	Ddx50	DEAD (Asp-	NM_053183	RefSeq	Mus muscul	FALSE	94213	Mm.114116	Q99MJ9	chr10	GO:001050	RNA second	GO:000563	nucleus // n	GO:000016	nucleotide b	O
17392991	104.2315	103.0877	1.01109541	0.01591914	0	main		NM_001289	Bcl2l1	BCL2-like 1	NM_001289	RefSeq	Mus muscul	FALSE	12048	Mm.238213	Q64373	chr2	GO:000091	cytokinesis	GO:000562	intracellular	GO:000551	protein bind	O
17270648	455.5771	450.7456	1.01071891	0.01538182	0	main		NM_001285	Cdc27	cell division	NM_001285	RefSeq	Mus muscul	FALSE	217232	Mm.89845	A2A6Q5	chr11	GO:000709	metaphase/	GO:000563	nucleus // n	GO:001990	protein pho	O
17273700	71.98607	71.29449	1.00970033	0.01392718	0	main		NM_153082	Dnajc27	DnaJ (Hsp4	NM_153082	RefSeq	Mus muscul	FALSE	217378	Mm.271656	Q8CFP6	chr12	GO:000688	intracellular	GO:000563	nucleus // n	GO:000016	nucleotide b	O
17367216	707.927	701.1299	1.00969449	0.01391884	0	main		NM_026162	Plxdc2	plexin doma	NM_026162	RefSeq	Mus muscul	FALSE	67448	Mm.313938	Q9DC11	chr2	---	---	GO:000588	plasma men	---	---	O
17383858	138.9653	137.8552	1.00805265	0.011571	0	main		NM_001301	Dnm1	dynamin 1	NM_001301	RefSeq	Mus muscul	FALSE	13429	Mm.44736	P39053	chr2	GO:000203	G-protein co	GO:000191	photorecept	GO:000016	nucleotide b	O
17339630	430.0514	426.7055	1.00784124	0.0112684	0	main		NM_007566	Birc6	baculoviral I	NM_007566	RefSeq	Mus muscul	FALSE	12211	Mm.442564	O88738	chr17	GO:000189	placenta de	GO:000092	spindle pole	GO:000484	ubiquitin-pr	O
17211774	135.4472	134.5085	1.00697874	0.01003323	0	main		NM_145516	Plekhb2	pleckstrin ho	NM_145516	RefSeq	Mus muscul	FALSE	226971	Mm.292751	Q9QZC7	chr1	GO:000815	biological_p	GO:000576	endosome /	GO:000367	molecular_f	O
17313739	161.4951	160.6974	1.00496399	0.0071438	0	main		NM_146061	Prr5	proline rich	NM_146061	RefSeq	Mus muscul	FALSE	109270	Mm.291372	Q812A5	chr15	GO:000193	positive reg	GO:003193	TORC2 com	GO:000509	GTPase act	O
17320693	40.02792	39.87461	1.0038448	0.00553624	0	main		NM_001033	Gxylt1	glucoside xy	NM_001033	RefSeq	Mus muscul	FALSE	223827	Mm.22819	Q3UHH8	chr15	GO:001626	O-glycan pr	GO:001602	membrane	GO:001674	transferase	O
17472777	203.5953	202.8967	1.00344313	0.00495886	0	main		NM_010586	Itpr2	inositol 1,4,5	NM_010586	RefSeq	Mus muscul	FALSE	16439	Mm.7800	Q9Z329	chr6	GO:000166	response to	GO:000573	cytoplasm /	GO:000521	ion channel	O
17228906	96.06995	95.89493	1.00182512	0.0026307	0	main		NM_001038	Dnm3	dynamin 3	NM_001038	RefSeq	Mus muscul	FALSE	103967	Mm.441620	Q8BZ98	chr1	GO:000689	endocytosis	GO:000191	photorecept	GO:000016	nucleotide b	O
17322327	221.7817	221.4854	1.00133779	0.00192873	0	main		NM_001076	Cbx5	chromobox 5	NM_001076	RefSeq	Mus muscul	FALSE	12419	Mm.262059	Q61686	chr15	GO:004589	negative reg	GO:000011	histone dea	GO:000368	chromatin b	O
17349465	147.4406	147.2714	1.0011489	0.00165656	0	main		NM_175104	Fam53c	family with s	NM_175104	RefSeq	Mus muscul	FALSE	66306	Mm.29424	Q8BXQ8	chr18	GO:000815	biological_p	GO:000557	cellular_com	GO:000367	molecular_f	O
17365106	60.20166	60.20166	1	0	0	main		NM_001033	Sec31b	Sec31 homo	NM_001033	RefSeq	Mus muscul	FALSE	240667	Mm.329873	Q3TZ89	chr19	GO:000681	transport //	GO:000573	cytoplasm /	GO:000367	molecular_f	O
17520292	14.80348	14.8044	0.99993786	-8.966E-05	0	main		NM_009576	Zic4	zinc finger p	NM_009576	RefSeq	Mus muscul	FALSE	22774	Mm.8062 //	Q61467	chr9	---	---	GO:000563	nucleus // n	GO:000367	nucleic acid	O
17397439	189.1386	189.1699	0.99983454	-0.0002387	0	main		NM_053089	Naa15	N(alpha)-ac	NM_053089	RefSeq	Mus muscul	FALSE	74838	Mm.275281	Q80UM3	chr3	GO:000152	angiogenesi	GO:000563	nucleus // tr	GO:000459	peptide alph	O
17360260	45.78741	45.8485	0.99866757	-0.0019236	0	main		NM_025696	Sorcs3	sortilin-relat	NM_025696	RefSeq	Mus muscul	FALSE	66673	Mm.70980	Q8VI51	chr19	GO:000761	learning //	GO:001602	membrane	GO:000551	protein bind	O
17531939	77.19527	77.39584	0.99740852	-0.0037436	0	main		NM_175380	Gpd1l	glycerol-3-pl	NM_175380	RefSeq	Mus muscul	FALSE	333433	Mm.38198	Q3ULJ0	chr9	GO:000202	regulation o	GO:000573	cytoplasm /	GO:000436	glycerol-3-p	O
17401036	14.4135	14.48568	0.99501715	-0.0072067	0	main		NM_178777	Nhlh2	nescient hel	NM_178777	RefSeq	Mus muscul	FALSE	18072	Mm.137286	Q64221	chr3	GO:000635	transcription	GO:000563	nucleus // n	GO:000097	RNA polym	O
17248929	98.51282	99.2331	0.99274153	-0.0105099	0	main		NM_001024	Zfp62	zinc finger p	NM_001024	RefSeq	Mus muscul	FALSE	22720	Mm.16650	Q8C827	chr11	GO:000635	transcription	GO:000563	nucleus	GO:000367	nucleic acid	O
17226676	109.7982	110.6904	0.99193968	-0.0116757	0	main		NM_001162	Pfkfb2	6-phosphofr	NM_001162	RefSeq	Mus muscul	FALSE	18640	Mm.249861	P70265	chr1	GO:000600	glucose cata	GO:000573	cytoplasm	GO:000387	6-phosphofr	O
17255565	32.57258	32.86808	0.99100951	-0.0130292	0	main		NM_010461	Hoxb8	homeobox B	NM_010461	RefSeq	Mus muscul	FALSE	15416	Mm.4822	P09632	chr11	GO:000635	transcription	GO:000563	nucleus	GO:000367	DNA binding	O
17491788	101.3889	102.3853	0.99026813	-0.0141089	0	main		NM_021366	Klf13	Kruppel-like	NM_021366	RefSeq	Mus muscul	FALSE	50794	Mm.240473	Q9JJZ6	chr7	GO:000635	transcription	GO:000563	nucleus // n	GO:000097	RNA polym	O
17527735	199.1843	201.2736	0.9896196	-0.015054	0	main		NM_001042	Neo1	neogenin	NM_001042	RefSeq	Mus muscul	FALSE	18007	Mm.42249	P97798	chr9	GO:000635	regulation o	GO:000588	plasma men	GO:000487	receptor act	O
17310004	154.1932	155.8364	0.98945561	-0.0152931	0	main		NM_030168	Rictor	RPTOR inde	NM_030168	RefSeq	Mus muscul	FALSE	78757	Mm.275811	Q6QI06	chr15	GO:000193	regulation o	GO:003193	TORC2 com	GO:000551	protein bind	O
17331918	53.24339	53.82352	0.98922163	-0.0156343	0	main		NM_001145	Tiam1	T cell lymph	NM_001145	RefSeq	Mus muscul	FALSE	21844	Mm.310902	Q60610	chr16	GO:000330	cardiac mus	GO:000563	nucleus // c	GO:000505	receptor sig	O
17317015	265.5808	268.6235	0.98867299	-0.0164347	0	main		NM_010162	Ext1	exostoses (r	NM_010162	RefSeq	Mus muscul	FALSE	14042	Mm.309395	P97464	chr15	GO:000150	ossification	GO:000578	endoplasmic	GO:000837	acetylglucos	O
17293237	66.84004	67.60615	0.98866804	-0.0164419	0	main		NM_001035	Golm1	golgi memb	NM_001035	RefSeq	Mus muscul	FALSE	105348	Mm.171335	Q91XA2	chr13	GO:000699	nucleus orga	GO:000561	extracellular	GO:000367	molecular_f	O
17309161	215.4636	218.3356	0.98684594	-0.0191032	0	main		NM_015822	Fbxl3	F-box and le	NM_015822	RefSeq	Mus muscul	FALSE	50789	Mm.214746	Q8C4V4	chr14	GO:001656	protein ubiq	GO:000563	nucleus // n	GO:000484	ubiquitin-pr	O
17267304	126.9211	128.722	0.98600938	-0.0203267	0	main		NM_001114	Rps6kb1	ribosomal pr	NM_001114	RefSeq	Mus muscul	FALSE	72508	Mm.394280	Q8BSK8	chr11	GO:000008	G1/S transit	GO:000563	nucleus // n	GO:000016	nucleotide b	O
17321094	49.67124	50.50181	0.98355366	-0.0239243	0	main		NM_001113	Col2a1	collagen, typ	NM_001113	RefSeq	Mus muscul	FALSE	12824	Mm.2423	P28481	chr15	GO:000150	skeletal sys	GO:000557	extracellular	GO:000520	extracellula	O
17530782	33.96802	34.54357	0.98333843	-0.0242401	0	main		NM_029103	Manf	mesenceph	NM_029103	RefSeq	Mus muscul	FALSE	74840	Mm.29778	Q9CXI5	chr9	GO:000201	vasoconstric	GO:000557	extracellular	GO:000808	growth facto	O
17513297	85.18399	86.73388	0.98213051	-0.0260133	0	main		NM_029441	Cdyl2	chromodom	NM_029441	RefSeq	Mus muscul	FALSE	75796	Mm.159575	Q9D5D8	chr8	GO:000815	metabolic pr	GO:000563	nucleus // n	GO:000382	catalytic act	O
17213336	226.3219	230.5548	0.98164037	-0.0267335	0	main		NM_007561	Bmpr2	bone morph	NM_007561	RefSeq	Mus muscul	FALSE	12168	Mm.7106	O35607	chr1	GO:000170	mesoderm f	GO:000561	extracellular	GO:000016	nucleotide b	O
17255877	115.001	117.2174	0.98109154	-0.0275403	0	main		NM_010688	Lasp1	LIM and SH	NM_010688	RefSeq	Mus muscul	FALSE	16796	Mm.271967	Q61792	chr11	GO:000681	transport //	GO:000573	cytoplasm /	GO:000377	actin binding	O
17460405	94.06579	96.07951	0.97904111	-0.0305587	0	main		NM_001164	Tia1	cytotoxic gra	NM_001164	RefSeq	Mus muscul	FALSE	21841	Mm.333219	P52912	chr6	GO:000691	apoptotic pr	GO:000563	nucleus // n	GO:000016	nucleotide b	O
17252113	157.911	161.4595	0.97802235	-0.0320607	0	main		NM_001045	Mink1	misshapen-l	NM_001045	RefSeq	Mus muscul	FALSE	50932	Mm.42967	Q9JM52	chr11	GO:000016	MAPK casc	GO:000573	cytoplasm /	GO:000016	nucleotide b	O
17418268	18.18605	18.60526	0.9774682	-0.0328783	0	main		NM_011141	Pou3f1	POU domain	NM_011141	RefSeq	Mus muscul	FALSE	18991	Mm.297371	P21952	chr4	GO:000635	transcription	GO:000563	nucleus // tr	GO:000110	RNA polym	O
17496237	71.36223	73.04269	0.97699345	-0.0335792	0	main		NM_001081	Sh2b1	SH2B adapt	NM_001081	RefSeq	Mus muscul	FALSE	20399	Mm.8538	Q91ZM2	chr7	GO:000692	movement c	GO:000172	ruffle // nucl	GO:000487	signal trans	O
17328201	192.1141	196.641	0.97697886	-0.0336007	0	main		NM_001130	Gspt1	G1 to S pha	NM_001130	RefSeq	Mus muscul	FALSE	14852	Mm.325827	Q8R050	chr16	GO:000018	nuclear-tran	GO:000582	cytosol // tr	GO:000016	nucleotide b	O
17403681	129.7543	132.8888	0.97641261	-0.0344372	0	main		NM_001080	Zzz3	zinc finger, Z	NM_001080	RefSeq	Mus muscul	FALSE	108946	Mm.379292	Q6KAQ7	chr3	GO:000635	transcription	GO:000563	nucleus // A	GO:000367	DNA binding	O
17352493	162.6633	166.6056	0.97633753	-0.0345481	0	main		NM_001276	Epc1	enhancer of	NM_001276	RefSeq	Mus muscul	FALSE	13831	Mm.312133	Q8C9X6	chr18	GO:000012	negative reg	GO:000563	nucleus // n	GO:000440	histone ace	O
17321365	233.281	238.9829	0.97614097	-0.0348386	0	main		NM_001033	Kmt2d	lysine (K)-sp	NM_001033	RefSeq	Mus muscul	FALSE	381022	Mm.264889	Q6PDK2	chr15	GO:000155	oocyte grow	GO:000563	nucleus // h	GO:000551	protein bind	O
17286595	343.1549	351.7958	0.97543774	-0.0358783	0	main		NM_001039	Rreb1	ras responsi	NM_001039	RefSeq	Mus muscul	FALSE	68750	Mm.491109	Q3UH06	chr13	GO:000012	negative reg	GO:000563	nucleus // n	GO:000097	RNA polym	O
17416897	149.8432	153.7336	0.97469389	-0.0369789	0	main		NM_007983	Faf1	Fas-associa	NM_007983	RefSeq	Mus muscul	FALSE	14084	Mm.318259	P54731	chr4	GO:000691	apoptotic pr	GO:000563	nucleus // n	GO:000551	protein bind	O
17346936	49.84377	51.22615	0.97301417	-0.0394673	0	main		NM_001164	Tgif1	TGFB-induc	NM_001164	RefSeq	Mus muscul	FALSE	21815	Mm.101034	P70284	chr17	GO:000012	negative reg	GO:000563	nucleus // n	GO:000097	RNA polym	O
17220724	320.4922	329.8594	0.97160245	-0.041562	0	main		NM_008976	Ptpn14	protein tyros	NM_008976	RefSeq	Mus muscul	FALSE	19250	Mm.4498	Q62130	chr1	GO:000194	lymphangio	GO:000563	nucleus // n	GO:000371	transcription	O
17255402	388.1954	400.3978	0.96952431	-0.044651	0	main		NM_025287	Spop	speckle-type	NM_025287	RefSeq	Mus muscul	FALSE	20747	Mm.285454	Q6ZWS8	chr11	GO:000012	negative reg	GO:000563	nucleus // n	GO:000108	RNA polym	O
17410130	104.2192	107.5914	0.96865735	-0.0459417	0	main		NM_001034	Ank2	ankyrin 2, br	NM_001034	RefSeq	Mus muscul	FALSE	109676	Mm.220242	Q8C8R3	chr3	GO:000202	regulation o	GO:000562	intracellular	GO:000551	protein bind	O
17451526	129.9602	134.5998	0.96553041	-0.0506064	0	main		NM_001159	Kctd10	potassium c	NM_001159	RefSeq	Mus muscul	FALSE	330171	Mm.490304	Q922M3	chr5	GO:005126	protein hom	GO:000563	nucleus // n	GO:000551	protein bind	O
17415035	39.0703	40.47544	0.96528413	-0.0509744	0	main		NM_001081	Ccdc171	coiled-coil d	NM_001081	RefSeq	Mus muscul	FALSE	320226	Mm.180032	E9Q1U1	chr4	GO:000815	biological_p	GO:000557	cellular_com	GO:000367	molecular_f	O
17414301	329.8987	341.8171	0.96513223	-0.0512015	0	main		NM_009011	Rad23b	RAD23b hor	NM_009011	RefSeq	Mus muscul	FALSE	19359	Mm.196846	P54728	chr4	GO:000071	nucleotide-e	GO:000050	proteasome	GO:000368	damaged D	O
17221317	230.2351	238.6837	0.96460336	-0.0519923	0	main		NM_001302	Ncoa2	nuclear rece	NM_001302	RefSeq	Mus muscul	FALSE	17978	Mm.2537	Q61026	chr1	GO:000012	negative reg	GO:000563	nucleus // n	GO:000097	RNA polym	O
17389861	141.4602	147.0268	0.96213886	-0.0556829	0	main		NM_026574	Ino80	INO80 homo	NM_026574	RefSeq	Mus muscul	FALSE	68142	Mm.330496	Q6ZPV2	chr2	GO:000007	mitotic siste	GO:000563	nucleus // n	GO:000016	nucleotide b	O
17467608	156.8787	163.3382	0.96045322	-0.0582127	0	main		NM_024288	Rmnd5a	required for	NM_024288	RefSeq	Mus muscul	FALSE	68477	Mm.28474	Q80YQ8	chr6	GO:000815	biological_p	GO:000557	cellular_com	GO:000367	molecular_f	O
17483577	196.5946	204.9219	0.95936354	-0.0598505	0	main		NM_001082	Itgam	integrin alph	NM_001082	RefSeq	Mus muscul	FALSE	16409	Mm.262106	P05555	chr7	GO:000252	leukocyte m	GO:000561	extracellular	GO:000184	opsonin bin	O

17405285	132.8202	138.5756	0.95846744	-0.0611987	0	main	NM_030595	Nbea	neurobeach	NM_030595	RefSeq	Mus muscul	FALSE	26422	Mm.384353	Q9EPN1	chr3	GO:000660	protein targe	GO:000580	trans-Golgi	GO:000554	phospholipi	O
17378640	104.3902	109.0877	0.95693832	-0.0635022	0	main	NM_001042	Dlgap4	discs, large	NM_001042	RefSeq	Mus muscul	FALSE	228836	Mm.22094	B1AZP2	chr2	GO:002305	signaling //	GO:000588	plasma men	GO:000551	protein bind	O
17409075	202.4454	211.6944	0.95630966	-0.0644502	0	main	NM_001113	Csf1	colony stimu	NM_001113	RefSeq	Mus muscul	FALSE	12977	Mm.795	P07141	chr3	GO:000150	ossification	GO:000557	extracellular	GO:000512	cytokine act	O
17236754	200.4728	210.1691	0.95386429	-0.0681441	0	main	NM_001001	Eea1	early endos	NM_001001	RefSeq	Mus muscul	FALSE	216238	Mm.490373	Q8BL66	chr10	GO:000689	endocytosis	GO:000573	cytoplasm /	GO:000551	protein bind	O
17276642	317.4429	333.1916	0.9527338	-0.0698549	0	main	NM_001252	Fut8	fucosyltrans	NM_001252	RefSeq	Mus muscul	FALSE	53618	Mm.35628	Q9WTS2	chr12	GO:000648	protein N-lin	GO:000573	cytoplasm /	GO:000842	glycoprotein	O
17347281	231.3239	242.8026	0.95272415	-0.0698695	0	main	NM_011500	Strn	striatin, calm	NM_011500	RefSeq	Mus muscul	FALSE	268980	Mm.391038	O55106	chr17	GO:000762	locomotory	GO:000015	protein phos	GO:000551	calmodulin	O
17469097	115.7327	121.5071	0.95247685	-0.0702441	0	main	NM_030081	Rbsn	rabenosyn,	NM_030081	RefSeq	Mus muscul	FALSE	78287	Mm.290734	Q80Y56	chr6	GO:000001	vacuole inhe	GO:000576	endosome /	GO:001713	Rab GTPas	O
17544446	19.31515	20.29059	0.95192648	-0.0710779	0	main	NM_183319	Xkrx	Kell blood g	NM_183319	RefSeq	Mus muscul	FALSE	331524	Mm.235055	Q5GH68	chrX	GO:000815	biological_p	GO:001602	membrane	GO:000367	molecular_f	O
17268493	228.4234	240.0384	0.95161191	-0.0715548	0	main	NM_008942	Npepps	aminopeptid	NM_008942	RefSeq	Mus muscul	FALSE	19155	Mm.29824	Q11011	chr11	GO:000650	proteolysis	GO:000563	nucleus // c	GO:000417	aminopeptic	O
17430777	87.44078	91.94738	0.95098718	-0.0725022	0	main	NM_001122	Gmeb1	glucocortico	NM_001122	RefSeq	Mus muscul	FALSE	56809	Mm.103489	Q9JL60	chr4	GO:000635	transcription	GO:000563	nucleus // c	GO:000097	RNA polyme	O
17223793	60.94183	64.12533	0.95035503	-0.0734615	0	main	NM_001042	Fzd5	frizzled hom	NM_001042	RefSeq	Mus muscul	FALSE	14367	Mm.150813	Q9EQD0	chr1	GO:000057	embryonic a	GO:000576	early endos	GO:000487	signal trans	O
17214841	384.799	405.0603	0.94997955	-0.0740316	0	main	NM_001310	Agfg1	ArfGAP with	NM_001310	RefSeq	Mus muscul	FALSE	15463	Mm.433409	Q8K2K6	chr1	GO:000167	acrosome a	GO:000563	nucleus // c	GO:000367	DNA binding	O
17528714	188.1032	198.0108	0.94996435	-0.0740547	0	main	NM_001253	Tcf12	transcription	NM_001253	RefSeq	Mus muscul	FALSE	21406	Mm.171615	Q61286	chr9	GO:000635	transcription	GO:000079	nuclear chro	GO:000097	RNA polyme	O
17324774	200.1603	210.8045	0.94950677	-0.0747498	0	main	NM_177633	Ubxn7	UBX domain	NM_177633	RefSeq	Mus muscul	FALSE	224111	Mm.31275	Q6P5G6	chr16	GO:000815	biological_p	GO:000563	nucleus // n	GO:000813	transcription	O
17233194	33.28751	35.06404	0.9493347	-0.0750113	0	main	NM_011376	Sim1	single-minde	NM_011376	RefSeq	Mus muscul	FALSE	20464	Mm.4774	Q61045	chr10	GO:000635	transcription	GO:000563	nucleus // n	GO:000098	RNA polyme	O
17496253	424.8951	448.0515	0.94831755	-0.0765579	0	main	NM_183020	Atxn2l	ataxin 2-like	NM_183020	RefSeq	Mus muscul	FALSE	233871	Mm.231450	Q7TQH0	chr7	GO:001060	regulation o	GO:000573	cytoplasm /	GO:004482	poly(A) RN	O
17264424	198.7773	209.7572	0.94765424	-0.0775673	0	main	NM_023668	Ndel1	nuclear distr	NM_023668	RefSeq	Mus muscul	FALSE	83431	Mm.31979	Q9ERR1	chr11	GO:000013	establishme	GO:000077	chromosom	GO:000551	protein bind	O
17384951	292.5509	308.8128	0.94734059	-0.0780449	0	main	NM_001289	Zeb2	zinc finger E	NM_001289	RefSeq	Mus muscul	FALSE	24136	Mm.440702	Q9R0G7	chr2	GO:000012	negative reg	GO:000563	nucleus // n	GO:000120	transcription	O
17322642	96.01075	101.4861	0.94604828	-0.0800143	0	main	NM_026666	Ubn1	ubinuclein 1	NM_026666	RefSeq	Mus muscul	FALSE	170644	Mm.66356	Q4G0F8	chr16	GO:000633	DNA replica	GO:000563	nucleus // b	GO:000367	DNA binding	O
17470060	148.8099	157.8423	0.9427758	-0.0850134	0	main	NM_178045	Rassf4	Ras associa	NM_178045	RefSeq	Mus muscul	FALSE	213391	Mm.257931	Q8CB96	chr6	GO:000704	cell cycle //	GO:000557	cellular_com	GO:000367	molecular_f	O
17227536	176.1598	186.871	0.94268131	-0.085158	0	main	NM_001111	Ptprc	protein tyros	NM_001111	RefSeq	Mus muscul	FALSE	19264	Mm.391573	P06800	chr1	GO:000018	activation of	GO:000588	plasma men	GO:000472	phosphopro	O
17260150	84.38442	89.54873	0.94232961	-0.0856963	0	main	NM_001080	Znrf3	zinc and ring	NM_001080	RefSeq	Mus muscul	FALSE	407821	Mm.216313	Q5SSZ7	chr11	GO:000651	ubiquitin-de	GO:000588	plasma men	GO:000484	ubiquitin-pr	O
17512846	147.222	156.2486	0.94222924	-0.08585	0	main	NM_001080	Atxn1l	ataxin 1-like	NM_001080	RefSeq	Mus muscul	FALSE	52335	Mm.276770	P0C7T6	chr8	GO:000012	negative reg	GO:000563	nucleus // n	GO:000367	DNA binding	O
17527883	102.0302	108.4842	0.94050747	-0.0884887	0	main	NM_001146	Lrrc49	leucine rich	NM_001146	RefSeq	Mus muscul	FALSE	102747	Mm.269413	Q91YK0	chr9	GO:000815	biological_p	GO:000573	cytoplasm /	GO:000367	molecular_f	O
17240670	8.177396	8.699137	0.94002382	-0.0892308	0	main	NM_001031	Lin28b	lin-28 homol	NM_001031	RefSeq	Mus muscul	FALSE	380669	Mm.440328	Q45KJ6	chr10	GO:000635	regulation o	GO:000563	nucleus // n	GO:000367	nucleic acid	O
17397344	161.039	171.355	0.9397975	-0.0895782	0	main	NM_001130	Jade1	jade family F	NM_001130	RefSeq	Mus muscul	FALSE	269424	Mm.286285	Q6ZPI0	chr3	GO:000635	transcription	GO:000012	histone acet	GO:000110	RNA polyme	O
17442507	449.5289	479.1761	0.9381288	-0.0921421	0	main	NM_001310	Setd8	SET domain	NM_001310	RefSeq	Mus muscul	FALSE	67956	Mm.137966	Q2YDW7	chr5	GO:000012	negative reg	GO:000563	nucleus // c	GO:000017	rRNA (aden	O
17266452	24.00856	25.60396	0.93768933	-0.0928181	0	main	NM_001168	Sarm1	sterile alpha	NM_001168	RefSeq	Mus muscul	FALSE	237868	Mm.210332	Q6PDS3	chr11	GO:000237	immune sys	GO:000573	cytoplasm /	GO:000551	protein bind	O
17470235	103.0731	110.1043	0.93614055	-0.0952029	0	main	NM_001159	Cacna1c	calcium cha	NM_001159	RefSeq	Mus muscul	FALSE	12288	Mm.436656	Q01815	chr6	GO:000681	transport //	GO:000209	caveolar ma	GO:000521	ion channel	O
17357502	136.5337	146.1631	0.9341188	-0.098322	0	main	NM_001164	Cpsf7	cleavage an	NM_001164	RefSeq	Mus muscul	FALSE	269061	Mm.490260	Q8BTV2	chr19	GO:000639	mRNA proc	GO:000563	nucleus // m	GO:000016	nucleotide b	O
17261494	101.8866	109.2224	0.93283612	-0.1003044	0	main	NM_001284	Nprl3	nitrogen per	NM_001284	RefSeq	Mus muscul	FALSE	17168	---	Q8VIJ8	chr11	GO:000328	ventricular s	GO:199013	Iml1 comple	GO:000509	GTPase act	O
17548736	25.74745	27.64126	0.93148612	-0.1023938	0	main	XM_006510	Ntm	neurotrimin	XM_006510	RefSeq	PREDICTED	FALSE	235106	Mm.283138	Q99PJ0	chr9	GO:000715	cell adhesio	GO:000561	extracellular	---	---	O
17413384	164.9937	177.1373	0.93144527	-0.1024571	0	main	NM_172866	Rgp1	RGP1 retrog	NM_172866	RefSeq	Mus muscul	FALSE	242406	Mm.259311	Q8BHT7	chr4	GO:004214	retrograde t	GO:000573	cytoplasm /	GO:000508	guanyl-nucl	O
17416540	49.83544	53.59624	0.9298309	-0.1049597	0	main	NM_001080	Lrp8	low density	NM_001080	RefSeq	Mus muscul	FALSE	16975	Mm.442134	Q924X6	chr4	GO:000012	negative reg	GO:000557	extracellular	GO:000194	glycoprotein	O
17449526	483.5239	520.3524	0.92922393	-0.1059018	0	main	NM_030886	Ankrd17	ankyrin repe	NM_030886	RefSeq	Mus muscul	FALSE	81702	Mm.245522	Q99NH0	chr5	GO:000195	blood vesse	GO:000078	chromatin /	GO:000367	nucleic acid	O
17283487	207.7614	223.7074	0.92871939	-0.1066853	0	main	NM_172806	Btbd7	BTB (POZ)	NM_172806	RefSeq	Mus muscul	FALSE	238386	Mm.440255	Q8CFE5	chr12	GO:000727	multicellular	GO:000563	nucleus // n	GO:000367	molecular_f	O
17524087	147.4853	158.9219	0.92803635	-0.1077468	0	main	NM_019683	Ankrd49	ankyrin repe	NM_019683	RefSeq	Mus muscul	FALSE	56503	Mm.395005	Q8VE42	chr9	GO:000728	spermatoge	GO:000563	nucleus // c	GO:000367	molecular_f	O
17321202	64.5414	69.5806	0.92757751	-0.1084602	0	main	NM_173769	Zfp641	zinc finger p	NM_173769	RefSeq	Mus muscul	FALSE	239652	Mm.207453	Q8BZ34	chr15	GO:000635	transcription	GO:000562	intracellular	GO:000098	RNA polyme	O
17496973	254.7121	275.0791	0.92595948	-0.110979	0	main	NM_001029	Ate1	arginyltransf	NM_001029	RefSeq	Mus muscul	FALSE	11907	Mm.216321	Q9Z2A5	chr7	GO:001659	protein argi	GO:000563	nucleus // n	GO:000405	arginyltrans	O
17313284	376.4796	406.896	0.92524773	-0.1120884	0	main	NM_177821	Ep300	E1A binding	NM_177821	RefSeq	Mus muscul	FALSE	328572	Mm.258397	B2RWS6	chr15	GO:000012	negative reg	GO:000012	histone acet	GO:000097	RNA polyme	O
17268469	305.329	330.3497	0.92425996	-0.1136294	0	main	NM_008379	Kpnb1	karyopherin	NM_008379	RefSeq	Mus muscul	FALSE	16211	Mm.251013	P70168	chr11	GO:000005	protein impo	GO:000563	nucleus // n	GO:000551	protein bind	O
17461888	37.35178	40.41676	0.92416562	-0.1137767	0	main	NM_001252	Hrh1	histamine re	NM_001252	RefSeq	Mus muscul	FALSE	15465	Mm.333327	P70174	chr6	GO:000695	inflammatory	GO:000565	nucleoplasm	GO:000487	signal trans	O
17440974	44.2491	47.90542	0.92367628	-0.1145408	0	main	NM_001004	Fam222a	family with s	NM_001004	RefSeq	Mus muscul	FALSE	433940	Mm.194212	Q6PGH4	chr5	GO:000815	biological_p	GO:000557	cellular_com	GO:000367	molecular_f	O
17285000	467.4717	506.2545	0.92339268	-0.1149838	0	main	NM_011803	Klf6	Kruppel-like	NM_011803	RefSeq	Mus muscul	FALSE	23849	Mm.275036	O08584	chr13	GO:000635	transcription	GO:000563	nucleus // n	GO:000367	nucleic acid	O
17266498	214.8669	232.713	0.92331284	-0.1151086	0	main	NM_008702	Nlk	nemo like ki	NM_008702	RefSeq	Mus muscul	FALSE	18099	Mm.9001	O54949	chr11	GO:000635	transcription	GO:000563	nucleus // c	GO:000016	nucleotide b	O
17500263	51.29652	55.59924	0.92261189	-0.1162042	0	main	NM_199042	Thap1	THAP doma	NM_199042	RefSeq	Mus muscul	FALSE	73754	Mm.383241	Q8CHW1	chr8	GO:000193	endothelial	GO:000563	nucleus // n	GO:000367	nucleic acid	O
17438246	304.7455	330.4792	0.92213216	-0.1169546	0	main	NM_001083	Pdgfra	platelet deri	NM_001083	RefSeq	Mus muscul	FALSE	18595	Mm.221403	P26618	chr5	GO:000155	luteinization	GO:000563	nucleus // n	GO:000016	nucleotide b	O
17269921	19.1623	20.79368	0.92154443	-0.1178744	0	main	NM_009764	Brca1	breast canc	NM_009764	RefSeq	Mus muscul	FALSE	12189	Mm.244975	P48754	chr11	GO:000072	double-stran	GO:000079	condensed	GO:000367	DNA binding	O
17467823	479.8243	521.7062	0.91972129	-0.1207314	0	main	NM_019715	Kcmf1	potassium c	NM_019715	RefSeq	Mus muscul	FALSE	74287	Mm.29194	Q80UY2	chr6	GO:001656	protein ubiq	---	---	GO:000827	zinc ion bin	O
17497153	106.3439	115.8392	0.91803034	-0.1233863	0	main	NM_001170	Ctbp2	C-terminal b	NM_001170	RefSeq	Mus muscul	FALSE	13017	Mm.246240	P56546	chr7	GO:000635	transcription	GO:000563	nucleus // n	GO:000368	chromatin b	O
17214711	58.14585	63.64584	0.91358445	-0.13039	0	main	NM_021342	Kcne4	potassium v	NM_021342	RefSeq	Mus muscul	FALSE	57814	Mm.24386	Q9WTW3	chr1	GO:000681	transport //	GO:000807	voltage-gate	GO:000524	voltage-gate	O
17360068	234.2424	256.9593	0.91159339	-0.1335376	0	main	NM_001177	Wbp1l	WW domain	NM_001177	RefSeq	Mus muscul	FALSE	226178	Mm.329895	Q8BGW2	chr19	GO:000815	biological_p	GO:001602	membrane	GO:000367	molecular_f	O
17547634	481.5294	528.2668	0.9115269	-0.1336429	0	main	NM_008379	Kpnb1	karyopherin	NM_008379	RefSeq	Mus muscul	FALSE	16211	Mm.251013	P70168	chr11	GO:000005	protein impo	GO:000563	nucleus // n	GO:000551	protein bind	O
17213321	1606.535	1763.723	0.91087716	-0.1346716	0	main	NM_007561	Bmpr2	bone morph	NM_007561	RefSeq	Mus muscul	FALSE	12168	Mm.7106	O35607	chr1	GO:000170	mesoderm f	GO:000561	extracellular	GO:000016	nucleotide b	O
17355962	170.3138	187.259	0.90950929	-0.1368397	0	main	NM_001167	Suv420h1	suppressor	NM_001167	RefSeq	Mus muscul	FALSE	225888	Mm.278578	Q3U8K7	chr19	GO:000635	transcription	GO:000078	condensed	GO:000816	methyltrans	O
17414009	52.16828	57.36261	0.90944746	-0.1369378	0	main	NM_001145	Msantd3	Myb/SANT-l	NM_001145	RefSeq	Mus muscul	FALSE	66665	Mm.140749	Q9CR78	chr4	GO:000815	biological_p	GO:000557	cellular_com	GO:004280	identical pro	O
17297196	157.5717	173.3384	0.90904093	-0.1375828	0	main	NM_139227	Atxn7	ataxin 7	NM_139227	RefSeq	Mus muscul	FALSE	246103	Mm.133625	Q8R4I1	chr14	GO:000022	microtubule	GO:000563	nucleus // n	GO:000368	chromatin b	O
17429810	56.79457	62.55722	0.90788194	-0.1394234	0	main	NM_023423	Akirin1	akirin 1	NM_023423	RefSeq	Mus muscul	FALSE	68050	Mm.486258	Q99LF1	chr4	GO:000815	biological_p	GO:000563	nucleus // n	GO:000367	molecular_f	O
17439164	326.452	359.6101	0.9077943	-0.1395627	0	main	NM_001009	Sept11	septin 11	NM_001009	RefSeq	Mus muscul	FALSE	52398	Mm.428571	Q8C1B7	chr5	GO:000704	cell cycle //	GO:000172	stress fiber	GO:000016	nucleotide b	O
17261470	177.5007	196.0684	0.90529989	-0.1435323	0	main	NM_001291	Rhbdf1	rhomboid fa	NM_001291	RefSeq	Mus muscul	FALSE	13650	Mm.11545	Q6PIX5	chr11	GO:000650	proteolysis	GO:000013	Golgi memb	GO:000425	serine-type	O
17469121	74.36642	82.14896	0.90526307	-0.143591	0	main	NM_001081	Prickle2	prickle hom	NM_001081	RefSeq	Mus muscul	FALSE	243548	Mm.248820	Q80Y24	chr6	GO:003117	neuron proje	GO:000563	nucleus // c	GO:000551	protein bind	O
17270134	201.646	223.3068	0.90299982	-0.1472024	0	main	NM_001044	Ubtf	upstream bi	NM_001044	RefSeq	Mus muscul	FALSE	21429	Mm.2845	P25976	chr11	GO:000018	chromatin si	GO:000165	fibrillar cent	GO:000367	DNA binding	O
17323360	79.84156	88.59486	0.90119856	-0.1500831	0	main	NM_176833	Ppm1f	protein phos	NM_176833	RefSeq	Mus muscul	FALSE	68606	Mm.230296	Q8CGA0	chr16	GO:000646	negative reg	GO:000562	intracellular	GO:000382	catalytic act	O
17427746	139.3273	155.1076	0.89826224	-0.1547914	0	main	NM_178143	Prkaa2	protein kinas	NM_178143	RefSeq	Mus muscul	FALSE	108079	Mm.48638	Q8BRK8	chr4	GO:000635	transcription	GO:000563	nucleus // n	GO:000016	nucleotide b	O
17276732	147.6067	164.5118	0.89724081	-0.1564329	0	main	NM_026114	Eif2s1	eukaryotic tr	NM_026114	RefSeq	Mus muscul	FALSE	13665	Mm.196220	Q6ZWX6	chr12	GO:000223	positive reg	GO:000563	nucleus // n	GO:000367	nucleic acid	O
17291559	40.4289	45.05966	0.89723047	-0.1564495	0	main	NM_001289	E2f3	E2F transcri	NM_001289	RefSeq	Mus muscul	FALSE	13557	Mm.268356	O35261	chr13	GO:000635	transcription	GO:000563	nucleus // n	GO:000104	core promot	O
17247448	131.7222	147.461	0.89326805	-0.1628349	0	main	NM_024459	Ppp3r1	protein phos	NM_024459	RefSeq	Mus muscul	FALSE	19058	Mm.41840	Q63810	chr11	GO:000156	patterning o	GO:000573	cytoplasm /	GO:000472	phosphopro	O
17260786	37.44823	41.92485	0.89322275	-0.1629081	0	main	NM_026576	Etaa1	Ewing tumor	NM_026576	RefSeq	Mus muscul	FALSE	68145	Mm.178684	Q5SVT3	chr11	GO:000815	biological_p	GO:000573	cytoplasm	GO:000367	molecular_f	O
17315718	88.58265	99.21346	0.89284912	-0.1635117	0	main	NM_001136	Ptger4	prostaglandi	NM_001136	RefSeq	Mus muscul	FALSE	19219	Mm.18509	P32240	chr15	GO:000193	positive reg	GO:000588	plasma men	GO:000487	signal trans	O
17348202	118.3686	132.8309	0.89112247	-0.1663044	0	main	NM_011546	Zeb1	zinc finger E	NM_011546	RefSeq	Mus muscul	FALSE	21417	Mm.3929	Q64318	chr18	GO:000012	negative reg	GO:000563	nucleus // n	GO:000122	transcription	O
17348138	355.739	399.2838	0.89094273	-0.1665954	0	main	NM_001081	9430020K01	RIKEN cDN	NM_001081	RefSeq	Mus muscul	FALSE	240185	Mm.33179	Q5DTX6	chr18	GO:000715	cell adhesio	GO:000591	cell-cell junc	GO:000367	molecular_f	O
17439741	293.4853	330.0945	0.88909479	-0.1695909	0	main	NM_001080	Aff1	AF4/FMR2 f	NM_001080	RefSeq	Mus muscul	FALSE	17355	Mm.6949	O88573	chr5	GO:000635	transcription	GO:000563	nucleus // tr	GO:000370	transcription	O
17517780	104.5196	117.5948	0.88881141	-0.1700508	0	main	NM_153799	Edc3	enhancer of	NM_153799	RefSeq	Mus muscul	FALSE	353190	Mm.268104	Q8K2D3	chr9	GO:009050	RNA phosph	GO:000573	cytoplasm /	GO:000372	RNA binding	O
17484952	126.0133	142.3519	0.88522387	-0.1758857	0	main	NM_001252	Tspan4	tetraspanin	NM_001252	RefSeq	Mus muscul	FALSE	64540	Mm.489619	Q9DCK3	chr7	GO:000646	protein comp	GO:000588	plasma men	GO:000382	antigen bind	O
17309041	55.85724	63.29967	0.88242545	-0.1804537	0	main	NM_001038	Dach1	dachshund	NM_001038	RefSeq	Mus muscul	FALSE	13134	Mm.320593	Q9QYB2	chr14	GO:000012	negative reg	GO:000563	nucleus // n	GO:000107	transcription	O
17231188	73.24398	83.30973	0.87917678	-0.1857748	0	main	NM_145415	Diexf	digestive org	NM_145415	RefSeq	Mus muscul	FALSE	215193	Mm.173758	Q8BTT6	chr1	GO:000046	maturation c	GO:000563	nucleus // n	GO:001984	rRNA bindin	O
17523736	54.80596	62.54014	0.87633254	-0.1904497	0	main	NM_029851	Dync2h1	dynein cytop	NM_029851	RefSeq	Mus muscul	FALSE	110350	Mm.237596	Q45VK7	chr9	GO:000701	microtubule-	GO:000573	cytoplasm /	GO:000016	nucleotide b	O
17523597	166.288	189.8677	0.87580984	-0.1913104	0	main	NM_013860	Limd1	LIM domains	NM_013860	RefSeq	Mus muscul	FALSE	29806	Mm.10987	Q9QXD8	chr9	GO:000166	response to	GO:000093	cytoplasmic	GO:000371	transcription	O
17282570	76.9639	87.88165	0.87576758	-0.1913801	0	main	NM_013589	Ltbp2	latent transf	NM_013589	RefSeq	Mus muscul	FALSE	16997	Mm.3900	O08999	chr12	GO:004320	extracellular	GO:000557	extracellular	GO:000550	calcium ion	O
17420761	82.50122	94.36894	0.87424125	-0.1938966	0	main	NM_001205	Iffo2	intermediate	NM_001205	RefSeq	Mus muscul	FALSE	212632	Mm.30464	Q8R2V2	chr4	GO:000815	biological_p	GO:000588	intermediate	GO:000519	structural m	O
17479548	239.235	274.72	0.87083212	-0.1995335	0	main	NM_178070	Vps33b	vacuolar pro	NM_178070	RefSeq	Mus muscul	FALSE	233405	Mm.322870	P59016	chr7	GO:000681	transport //	GO:000573	cytoplasm /	GO:000551	protein bind	O
17424850	169.4346	194.827	0.86966694	-0.2014651	0	main	NM_001038	Rnf38	ring finger p	NM_001038	RefSeq	Mus muscul	FALSE	73469	Mm.262859	Q8BI21	chr4	GO:001656	protein ubiq	GO:000563	nucleus // n	GO:000484	ubiquitin-pr	O
17471541	58.46545	67.25922	0.86925555	-0.2021477	0	main	NM_001309	Clec7a	C-type lectin	NM_001309	RefSeq	Mus muscul	FALSE	56644	Mm.239516	Q6QLQ4	chr6	GO:000222	pattern reco	GO:000588	plasma men	GO:000551	protein bind	O
17439481	351.3684	406.1239	0.86517538	-0.2089355	0	main	NM_080708	Bmp2k	BMP2 induc	NM_080708	RefSeq	Mus muscul	FALSE	140780	Mm.281490	Q91Z96	chr5	GO:000646	protein phos	GO:000563	nucleus // n	GO:000016	nucleotide b	O
17243525	58.8191	68.13747	0.86324162	-0.2121637	0	main	NM_177388	Slc41a2	solute carrie	NM_177388	RefSeq	Mus muscul	FALSE	338365	Mm.67073	Q8BYR8	chr10	GO:000681	transport //	GO:000588	plasma men	GO:000832	cation trans	O
17453573	369.8721	428.659	0.86285859	-0.212804	0	main	NM_146001	Hip1	huntingtin in	NM_146001	RefSeq	Mus muscul	FALSE	215114	Mm.280805	Q8VD75	chr5	GO:000635	transcription	GO:000563	nucleus // c	GO:000377	actin binding	O
17325014	288.2719	334.1208	0.86277747	-0.2129396	0	main	NM_080557	Snx4	sorting nexin	NM_080557	RefSeq	Mus muscul	FALSE	69150	Mm.28196	Q91YJ2	chr16	GO:000681	transport //	GO:000573	cytoplasm /	GO:000515	epidermal g	O
17496251	52.3025	60.67113	0.8620657	-0.2141303	0	main	NM_001289	Sh2b1	SH2B adapt	NM_001289	RefSeq	Mus muscul	FALSE	20399	Mm.8538	Q91ZM2	chr7	GO:000692	movement c	GO:000172	ruffle // nucl	GO:000487	signal trans	O
17440257	97.41857	113.0726	0.86155771	-0.2149807	0	main	NM_172716	Pcgf3	polycomb gr	NM_172716	RefSeq	Mus muscul	FALSE	69587	Mm.486543	Q8BTQ0	chr5	GO:000635	transcription	GO:000563	nucleus // n	GO:000827	zinc ion bin	O
17521405	59.00763	68.67085	0.85928207	-0.2187963	0	main	NM_001243	Rassf1	Ras associa	NM_001243	RefSeq	Mus muscul	FALSE	56289	Mm.12091	Q99MK9	chr9	GO:000697	cellular resp	GO:000563	nucleus // c	GO:000551	protein bind	O
17405142	137.5441	160.3999	0.85750739	-0.221779	0	main	NM_080793	Setd7	SET domain	NM_080793	RefSeq	Mus muscul	FALSE	73251	Mm.192111	Q8VHL1	chr3	GO:000635	transcription	GO:000563	nucleus // c	GO:000203	p53 binding	O
17341681	77.30131	90.18329	0.85715779	-0.2223673	0	main	NM_027008	Kctd5	potassium c	NM_027008	RefSeq	Mus muscul	FALSE	69259	Mm.28171	Q8VC57	chr17	GO:005126	protein hom	GO:000573	cytoplasm /	GO:003240	protein com	O
17238449	157.545	183.9187	0.85660131	-0.2233042	0	main	NM_172790	Ankrd52	ankyrin repe	NM_172790	RefSeq	Mus muscul	FALSE	237615	Mm.440177	Q8BTI7	chr10	GO:000815	biological_p	GO:000557	cellular_com	GO:000367	molecular_f	O
17393160	79.98725	93.48403	0.85562475	-0.2249499	0	main	NM_001291	E2f1	E2F transcri	NM_001291	RefSeq	Mus muscul	FALSE	13555	Mm.18036	Q61501	chr2	GO:000007	DNA damag	GO:000079	nuclear chro	GO:000104	core promot	O
17353143	109.8206	128.448	0.854981	-0.2260357	0	main	NM_144861	Rprd1a	regulation o	NM_144861	RefSeq	Mus muscul	FALSE	225283	Mm.28427	Q8VDS4	chr18	GO:007094	dephosphor	GO:001659	DNA-directe	GO:000367	molecular_f	O
17370487	57.64075	67.76946	0.85054167	-0.2335462	0	main	NM_172854	Olfml2a	olfactomedin	NM_172854	RefSeq	Mus muscul	FALSE	241327	Mm.103491	Q8BHP7	chr2	GO:003019	extracellular	GO:000557	extracellular	GO:004280	protein hom	O
17269695	170.4522	200.6461	0.84951664	-0.2352859	0	main	NM_001113	Stat5b	signal trans	NM_001113	RefSeq	Mus muscul	FALSE	20851	Mm.34064	P42232	chr11	GO:000025	allantoin me	GO:000563	nucleus // n	GO:000097	RNA polyme	O
17239090	272.2291	321.2226	0.84747804	-0.2387521	0	main	NM_138667	Tab2	TGF-beta ac	NM_138667	RefSeq	Mus muscul	FALSE	68652	Mm.193041	Q99K90	chr10	GO:000750	heart develo	GO:000563	nucleus // c	GO:000551	protein bind	O
17384787	108.1278	128.3552	0.84241075	-0.2474043	0	main	NM_178778	Scai	suppressor	NM_178778	RefSeq	Mus muscul	FALSE	320271	Mm.204836	Q8C8N2	chr2	GO:000635	transcription	GO:000563	nucleus // n	GO:000371	transcription	O
17385840	28.90829	34.32003	0.84231541	-0.2475675	0	main	NM_001267	Fign	fidgetin	NM_001267	RefSeq	Mus muscul	FALSE	60344	Mm.130769	Q9ERZ6	chr2	GO:000704	cell cycle //	GO:000563	nucleus // c	GO:000016	nucleotide b	O
17359769	33.02648	39.39051	0.83843748	-0.2542249	0	main	NM_011976	Sema4g	sema domai	NM_011976	RefSeq	Mus muscul	FALSE	26456	Mm.34404	Q9WUH7	chr19	GO:000175	neural crest	GO:000561	extracellular	GO:000551	protein bind	O
17223766	214.3839	256.781	0.83489004	-0.2603419	0	main	NM_033563	Klf7	Kruppel-like	NM_033563	RefSeq	Mus muscul	FALSE	93691	Mm.29466	Q99JB0	chr1	GO:000635	transcription	GO:000563	nucleus	GO:000367	nucleic acid	O
17371912	117.7121	141.153	0.83393268	-0.2619972	0	main	NM_001164	Zak	sterile alpha	NM_001164	RefSeq	Mus muscul	FALSE	65964	Mm.314618	Q9ESL4	chr2	GO:000007	cell cycle ch	GO:000563	nucleus // c	GO:000016	nucleotide b	O
17529481	48.26484	58.60971	0.82349563	-0.2801671	0	main	NM_023814	Tbx18	T-box18	NM_023814	RefSeq	Mus muscul	FALSE	76365	Mm.158789	Q9EPZ6	chr9	GO:000012	negative reg	GO:000563	nucleus // T	GO:000097	transcription	O
17510685	324.9632	395.2768	0.82211554	-0.2825869	0	main	NM_010332	Ednra	endothelin r	NM_010332	RefSeq	Mus muscul	FALSE	13617	Mm.283168	Q61614	chr8	GO:000156	patterning o	GO:000582	cytosol // pl	GO:000471	protein tyros	O
17245334	143.8966	175.1239	0.82168453	-0.2833435	0	main	NM_001014	Dyrk2	dual-specific	NM_001014	RefSeq	Mus muscul	FALSE	69181	Mm.458174	Q5U4C9	chr10	GO:000646	protein phos	GO:000015	ubiquitin lig	GO:000016	nucleotide b	O
17452456	76.258	93.01814	0.81981859	-0.2866234	0	main	NM_001199	Camkk2	calcium/caln	NM_001199	RefSeq	Mus muscul	FALSE	207565	Mm.289237	Q8C078	chr5	GO:000646	protein phos	GO:000563	nucleus // c	GO:000016	nucleotide b	O
17218233	28.8106	35.34816	0.81505233	-0.2950354	0	main	NM_177756	Colgalt2	collagen bet	NM_177756	RefSeq	Mus muscul	FALSE	269132	Mm.23782	Q6NVG7	chr1	GO:000815	biological_p	GO:000578	endoplasmic	GO:001674	transferase	O
17219362	282.6627	347.3684	0.813726	-0.297385	0	main	NM_172647	F11r	F11 recepto	NM_172647	RefSeq	Mus muscul	FALSE	16456	Mm.294882	O88792	chr1	GO:000181	regulation o	GO:000588	plasma men	GO:000551	protein bind	O
17219079	59.14584	72.70006	0.81355971	-0.2976799	0	main	NM_001160	Fam78b	family with s	NM_001160	RefSeq	Mus muscul	FALSE	226610	Mm.389217	Q8BQN5	chr1	GO:000815	biological_p	GO:000557	cellular_com	GO:000367	molecular_f	O
17465212	283.8586	349.9285	0.81119029	-0.3018877	0	main	NM_001167	Wasl	Wiskott-Aldr	NM_001167	RefSeq	Mus muscul	FALSE	73178	Mm.1574	Q91YD9	chr6	GO:000635	transcription	GO:000013	Golgi memb	GO:000377	actin binding	O
17264651	188.0549	232.019	0.81051509	-0.3030891	0	main	NM_001017	Kdm6b	KDM1 lysine	NM_001017	RefSeq	Mus muscul	FALSE	216850	Mm.261201	Q5NCY0	chr11	GO:000695	inflammatory	GO:000563	nucleus // n	GO:000090	sulfonate di	O
17465272	9.531133	11.76178	0.81034784	-0.3033868	0	main	NM_001311	Grm8	glutamate re	NM_001311	RefSeq	Mus muscul	FALSE	14823	Mm.320732	P47743	chr6	GO:000716	signal trans	GO:000588	plasma men	GO:000164	group III me	O
17289794	111.5239	138.1331	0.8073655	-0.3087061	0	main	NM_152804	Plk2	polo-like kin	NM_152804	RefSeq	Mus muscul	FALSE	20620	Mm.380	P53351	chr13	GO:000008	G1/S transit	GO:000078	chromatin /	GO:000016	nucleotide b	O
17395985	81.38059	101.3153	0.80324087	-0.3160954	0	main	NM_030708	Zfhx4	zinc finger h	NM_030708	RefSeq	Mus muscul	FALSE	80892	Mm.41522	Q9JJN2	chr3	GO:000635	transcription	GO:000563	nucleus // n	GO:000367	nucleic acid	O
17527565	103.0535	129.0039	0.79884019	-0.3240212	0	main	NM_001304	Csk	c-src tyrosin	NM_001304	RefSeq	Mus muscul	FALSE	12988	Mm.21974	P41241	chr9	GO:000181	regulation o	GO:000573	cytoplasm /	GO:000016	nucleotide b	O
17345321	57.68089	72.42004	0.79647691	-0.3282955	0	main	NM_030715	Polh	polymerase	NM_030715	RefSeq	Mus muscul	FALSE	80905	Mm.311585	Q9JJN0	chr17	GO:000073	DNA synthe	GO:000563	nucleus // n	GO:000367	DNA binding	O
17333222	50.90661	64.18011	0.79318359	-0.3342733	0	main	NM_026644	Agpat4	1-acylglycer	NM_026644	RefSeq	Mus muscul	FALSE	68262	Mm.258300	Q8K4X7	chr17	GO:000662	lipid metabo	GO:001602	membrane	GO:000384	1-acylglycer	O
17338872	33.22202	42.13338	0.78849644	-0.3428239	0	main	NM_027244	Ndufa11	NADH dehy	NM_027244	RefSeq	Mus muscul	FALSE	69875	Mm.279823	Q9D8B4	chr17	GO:000681	transport //	GO:000573	mitochondri	GO:000367	molecular_f	O
17299288	106.3239	135.9234	0.78223396	-0.3543279	0	main	NM_001037	Samd4	sterile alpha	NM_001037	RefSeq	Mus muscul	FALSE	74480	Mm.214454	Q8CBY1	chr14	GO:000635	regulation o	GO:000573	cytoplasm /	GO:000372	RNA binding	O
17315152	133.9278	171.9612	0.77882569	-0.3606276	0	main	NM_007395	Acvr1b	activin A rec	NM_007395	RefSeq	Mus muscul	FALSE	11479	Mm.308467	Q61271	chr15	GO:000008	G1/S transit	GO:000588	plasma men	GO:000016	nucleotide b	O
17294187	39.74664	51.63868	0.76970674	-0.3776192	0	main	NM_172053	Adamts16	a disintegrin	NM_172053	RefSeq	Mus muscul	FALSE	271127	Mm.115970	Q69Z28	chr13	GO:000165	branching in	GO:000557	extracellular	GO:000422	metalloendo	O
17436237	207.721	272.6559	0.76184304	-0.3924343	0	main	NM_008037	Fosl2	fos-like antig	NM_008037	RefSeq	Mus muscul	FALSE	14284	Mm.24684	P47930	chr5	GO:000166	response to	GO:000563	nucleus // n	GO:000097	RNA polyme	O
17336086	122.7896	161.3051	0.76122578	-0.3936037	0	main	NM_172619	Adamts10	a disintegrin	NM_172619	RefSeq	Mus muscul	FALSE	224697	Mm.29304	P58459	chr17	GO:000650	proteolysis	GO:000152	microfibril //	GO:000422	metalloendo	O
17535607	227.0403	299.2179	0.75877914	-0.3982481	0	main	NM_001142	Slc6a8	solute carrie	NM_001142	RefSeq	Mus muscul	FALSE	102857	Mm.274553	Q8VBW1	chrX	GO:000681	transport //	GO:000588	integral com	GO:000530	creatine tra	O
17366451	88.71024	117.3319	0.75606242	-0.4034228	0	main	NM_001177	Frmd4a	FERM doma	NM_001177	RefSeq	Mus muscul	FALSE	209630	Mm.37932	Q8BIE6	chr2	GO:009016	establishme	GO:000573	cytoplasm /	GO:003067	protein bind	O
17285438	26.17583	35.38058	0.73983609	-0.4347224	0	main	NM_008380	Inhba	inhibin beta-	NM_008380	RefSeq	Mus muscul	FALSE	16323	Mm.8042	Q04998	chr13	GO:000008	G1/S transit	GO:000557	extracellular	GO:000510	receptor bin	O
17229454	107.3692	145.3002	0.73894737	-0.4364565	0	main	NM_009062	Rgs4	regulator of	NM_009062	RefSeq	Mus muscul	FALSE	19736	Mm.41642	O08899	chr1	GO:000718	G-protein co	GO:000563	nucleus // c	GO:000196	G-protein al	O
17410410	92.14751	124.8168	0.73826208	-0.437795	0	main	NM_028943	Sgms2	sphingomye	NM_028943	RefSeq	Mus muscul	FALSE	74442	Mm.273360	Q9D4B1	chr3	GO:000662	lipid metabo	GO:000579	Golgi appar	GO:001630	kinase activ	O
17257405	134.4901	182.3899	0.73737691	-0.4395259	0	main	NM_181071	Tanc2	tetratricopep	NM_181071	RefSeq	Mus muscul	FALSE	77097	Mm.22501	A2A690	chr11	GO:000170	in utero emb	GO:000557	cellular_com	GO:000367	molecular_f	O
17419305	16.84149	23.11808	0.72849865	-0.4570018	0	main	NM_010769	Matn1	matrilin 1, ca	NM_010769	RefSeq	Mus muscul	FALSE	17180	Mm.271796	P51942	chr4	GO:000206	chondrocyte	GO:000557	extracellular	GO:000551	protein bind	O
17541618	27.60691	38.11409	0.72432295	-0.465295	0	main	NM_001310	Mbnl3	muscleblind	NM_001310	RefSeq	Mus muscul	FALSE	171170	Mm.295324	Q8R003	chrX	GO:000639	mRNA proc	GO:000563	nucleus // c	GO:004482	poly(A) RN	O
17455801	545.3482	769.1423	0.70903421	-0.4960729	0	main	NM_007743	Col1a2	collagen, typ	NM_007743	RefSeq	Mus muscul	FALSE	12843	Mm.277792	Q01149	chr6	GO:000150	skeletal syst	GO:000557	extracellular	GO:000520	extracellular	O
17289527	89.56566	128.2702	0.69825774	-0.5181684	0	main	NM_008533	Cd180	CD180 antig	NM_008533	RefSeq	Mus muscul	FALSE	17079	Mm.373974	Q62192	chr13	GO:000232	B cell prolife	GO:000588	plasma men	---	---	O

Supplementary Table 5. The comparison of global mRNA expression in epididymal adipose tissues of *Mir342* (+/+) mice fed with high fat-high sucrose (HFHS) and standard (STD) chow.

	Sample name	Negative control (average)
Test	HFHS *Mir342* (+/+) 696	42.10
Control	STD *Mir342* (+/+) 669	50.72

2Up	Ratio ≧ 2 and<br>Expression value of test sample ≧ Negative control (average)
2Down	Ratio ≦ 0.5 and<br>Expression value of test sample ≧ Negative control (average)

Feature ID	Normalized data				Flag			Annotation																	Keyword
Probe Set ID	Expression v	Expression v	Ratio	Log2Ratio	解析対象 Fl	category	Ratio flag	Gene Acces	Gene Symb	Gene Descr	mRNA Acce	mRNA Sou	mRna - Des	mRNA - xhy	EntrezGene	UniGene ID	SwissProt a	Chromosom	GO Biologic	GO Biologic	GO Cellular	GO Cellular	GO Molecul	GO Molecul	miR342 target gene
17289527	128.2702	59.23219	2.16554883	1.11473271	0	main	2Up	NM_008533	Cd180	CD180 antig	NM_008533	RefSeq	Mus muscul	FALSE	17079	Mm.373974	Q62192	chr13	GO:000232	B cell prolife	GO:000588	plasma men	---	---	○
17331918	53.82352	27.3346	1.96906192	0.97750848	0	main		NM_001145	Tiam1	T cell lymph	NM_001145	RefSeq	Mus muscul	FALSE	21844	Mm.310902	Q60610	chr16	GO:000330	cardiac mus	GO:000563	nucleus // c	GO:000505	receptor sig	○
17336086	161.3051	84.18594	1.91605748	0.93814084	0	main		NM_172619	Adamts10	a disintegrin	NM_172619	RefSeq	Mus muscul	FALSE	224697	Mm.29304	P58459	chr17	GO:000650	proteolysis	GO:000152	microfibril //	GO:000422	metalloendo	○
17294187	51.63868	27.52689	1.87593586	0.9076105	0	main		NM_172053	Adamts16	a disintegrin	NM_172053	RefSeq	Mus muscul	FALSE	271127	Mm.115970	Q69Z28	chr13	GO:000165	branching in	GO:000557	extracellular	GO:000422	metalloendo	○
17227536	186.871	104.0114	1.7966396	0.84530104	0	main		NM_001111	Ptprc	protein tyros	NM_001111	RefSeq	Mus muscul	FALSE	19264	Mm.391573	P06800	chr1	GO:000018	activation of	GO:000588	plasma men	GO:000472	phosphopro	○
17455801	769.1423	431.3169	1.78324174	0.83450229	0	main		NM_007743	Col1a2	collagen, typ	NM_007743	RefSeq	Mus muscul	FALSE	12843	Mm.277792	Q01149	chr6	GO:000150	skeletal syst	GO:000557	extracellular	GO:000520	extracellular	○
17257405	182.3899	102.7492	1.77509801	0.82789868	0	main		NM_181071	Tanc2	tetratricopep	NM_181071	RefSeq	Mus muscul	FALSE	77097	Mm.22501	A2A690	chr11	GO:000170	in utero emb	GO:000557	cellular_com	GO:000367	molecular_f	○
17359769	39.39051	23.11076	1.70442296	0.76928339	0	main		NM_011976	Sema4g	sema domai	NM_011976	RefSeq	Mus muscul	FALSE	26456	Mm.34404	Q9WUH7	chr19	GO:000175	neural crest	GO:000561	extracellular	GO:000551	protein bind	○
17442507	479.1761	292.572	1.63780574	0.71176425	0	main		NM_001310	Setd8	SET domain	NM_001310	RefSeq	Mus muscul	FALSE	67956	Mm.137966	Q2YDW7	chr5	GO:000012	negative reg	GO:000563	nucleus // c	GO:000017	rRNA (aden	○
17483577	204.9219	126.2055	1.62371608	0.69929939	0	main		NM_001082	Itgam	integrin alph	NM_001082	RefSeq	Mus muscul	FALSE	16409	Mm.262106	P05555	chr7	GO:000252	leukocyte m	GO:000561	extracellular	GO:000184	opsonin bin	○
17453573	428.659	270.5324	1.58450152	0.66402905	0	main		NM_146001	Hip1	huntingtin in	NM_146001	RefSeq	Mus muscul	FALSE	215114	Mm.280805	Q8VD75	chr5	GO:000635	transcription	GO:000563	nucleus // c	GO:000377	actin binding	○
17383858	137.8552	88.12531	1.56430882	0.64552536	0	main		NM_001301	Dnm1	dynamin 1	NM_001301	RefSeq	Mus muscul	FALSE	13429	Mm.44736	P39053	chr2	GO:000203	G-protein cc	GO:000191	photorecept	GO:000016	nucleotide b	○
17410130	107.5914	69.52476	1.54752638	0.629964	0	main		NM_001034	Ank2	ankyrin 2, br	NM_001034	RefSeq	Mus muscul	FALSE	109676	Mm.220242	Q8C8R3	chr3	GO:000202	regulation o	GO:000562	intracellular	GO:000551	protein bind	○
17219079	72.70006	47.64585	1.52584244	0.609606	0	main		NM_001160	Fam78b	family with s	NM_001160	RefSeq	Mus muscul	FALSE	226610	Mm.389217	Q8BQN5	chr1	GO:000815	biological_p	GO:000557	cellular_com	GO:000367	molecular_f	○
17393160	93.48403	61.38258	1.52297329	0.60689064	0	main		NM_001291	E2f1	E2F transcri	NM_001291	RefSeq	Mus muscul	FALSE	13555	Mm.18036	Q61501	chr2	GO:000007	DNA damag	GO:000079	nuclear chrc	GO:000104	core promot	○
17377498	371.3322	246.0206	1.50935409	0.5939313	0	main		NM_153781	Pygb	brain glycog	NM_153781	RefSeq	Mus muscul	FALSE	110078	Mm.222584	Q8CI94	chr2	GO:000597	carbohydrat	GO:000573	cytoplasm /	GO:000382	catalytic act	○
17248747	17.36692	11.61673	1.49499214	0.5801379	0	main		NM_173384	Sox30	SRY (sex de	NM_173384	RefSeq	Mus muscul	FALSE	214105	Mm.66708	Q8CGW4	chr11	GO:000635	transcription	GO:000563	nucleus // n	GO:000367	DNA binding	○
17229454	145.3002	98.45354	1.47582504	0.5615217	0	main		NM_009062	Rgs4	regulator of	NM_009062	RefSeq	Mus muscul	FALSE	19736	Mm.41642	O08899	chr1	GO:000718	G-protein cc	GO:000563	nucleus // c	GO:000196	G-protein al	○
17299288	135.9234	93.69013	1.45077608	0.53682487	0	main		NM_001037	Samd4	sterile alpha	NM_001037	RefSeq	Mus muscul	FALSE	74480	Mm.214454	Q8CBY1	chr14	GO:000635	regulation o	GO:000573	cytoplasm /	GO:000372	RNA binding	○
17325671	28.37711	19.78366	1.43437109	0.52041832	0	main		NM_175548	Lsamp	limbic syster	NM_175548	RefSeq	Mus muscul	FALSE	268890	Mm.310524	Q8BLK3	chr16	GO:000715	cell adhesio	GO:000588	plasma men	---	---	○
17529481	58.60971	41.07187	1.42700369	0.51298906	0	main		NM_023814	Tbx18	T-box18	NM_023814	RefSeq	Mus muscul	FALSE	76365	Mm.158789	Q9EPZ6	chr9	GO:000012	negative reg	GO:000563	nucleus // T	GO:000097	transcription	○
17429810	62.55722	44.11662	1.41799666	0.50385414	0	main		NM_023423	Akirin1	akirin 1	NM_023423	RefSeq	Mus muscul	FALSE	68050	Mm.486258	Q99LF1	chr4	GO:000815	biological_p	GO:000563	nucleus // n	GO:000367	molecular_f	○
17282570	87.88165	62.56913	1.40455285	0.49011091	0	main		NM_013589	Ltbp2	latent transf	NM_013589	RefSeq	Mus muscul	FALSE	16997	Mm.3900	O08999	chr12	GO:004320	extracellular	GO:000557	extracellular	GO:000550	calcium ion	○
17419305	23.11808	16.80467	1.37569378	0.46015937	0	main		NM_010769	Matn1	matrilin 1, ca	NM_010769	RefSeq	Mus muscul	FALSE	17180	Mm.271796	P51942	chr4	GO:000206	chondrocyte	GO:000557	extracellular	GO:000551	protein bind	○
17525894	80.42152	58.70789	1.3698588	0.4540272	0	main		NM_011436	Sorl1	sortilin-relate	NM_011436	RefSeq	Mus muscul	FALSE	20660	Mm.121920	O88307	chr9	GO:000004	protein targe	GO:000557	extracellular	GO:000154	beta-amyloi	○
17484952	142.3519	104.0389	1.36825649	0.4523387	0	main		NM_001252	Tspan4	tetraspanin 4	NM_001252	RefSeq	Mus muscul	FALSE	64540	Mm.489619	Q9DCK3	chr7	GO:000646	protein com	GO:000588	plasma men	GO:000382	antigen binc	○
17370487	67.76946	50.10674	1.35250188	0.4356306	0	main		NM_172854	Olfml2a	olfactomedir	NM_172854	RefSeq	Mus muscul	FALSE	241327	Mm.103491	Q8BHP7	chr2	GO:003019	extracellular	GO:000557	extracellular	GO:004280	protein hom	○
17401036	14.48568	10.82743	1.33786873	0.41993657	0	main		NM_178777	Nhlh2	nescient hel	NM_178777	RefSeq	Mus muscul	FALSE	18072	Mm.137286	Q64221	chr3	GO:000635	transcription	GO:000563	nucleus // n	GO:000097	RNA polyme	○
17465272	11.76178	8.866076	1.32660492	0.40773878	0	main		NM_001311	Grm8	glutamate re	NM_001311	RefSeq	Mus muscul	FALSE	14823	Mm.320732	P47743	chr6	GO:000716	signal trans	GO:000588	plasma men	GO:000164	group III me	○
17269921	20.79368	15.69282	1.32504419	0.40604048	0	main		NM_009764	Brca1	breast canc	NM_009764	RefSeq	Mus muscul	FALSE	12189	Mm.244975	P48754	chr11	GO:000072	double-strar	GO:000079	condensed	GO:000367	DNA binding	○
17291559	45.05966	34.43858	1.30840644	0.38781077	0	main		NM_001289	E2f3	E2F transcri	NM_001289	RefSeq	Mus muscul	FALSE	13557	Mm.268356	O35261	chr13	GO:000635	transcription	GO:000563	nucleus // n	GO:000104	core promot	○
17338872	42.13338	32.30096	1.30440024	0.38338661	0	main		NM_027244	Ndufa11	NADH dehy	NM_027244	RefSeq	Mus muscul	FALSE	69875	Mm.279823	Q9D8B4	chr17	GO:000681	transport //	GO:000573	mitochondri	GO:000367	molecular_f	○
17439741	330.0945	253.4995	1.3021505	0.3808962	0	main		NM_001080	Aff1	AF4/FMR2 f	NM_001080	RefSeq	Mus muscul	FALSE	17355	Mm.6949	O88573	chr5	GO:000635	transcription	GO:000563	nucleus // tr	GO:000370	transcription	○
17409075	211.6944	162.8043	1.30029981	0.3788443	0	main		NM_001113	Csf1	colony stimu	NM_001113	RefSeq	Mus muscul	FALSE	12977	Mm.795	P07141	chr3	GO:000150	ossification	GO:000557	extracellular	GO:000512	cytokine act	○
17345321	72.42004	55.86045	1.2964457	0.37456178	0	main		NM_030715	Polh	polymerase	NM_030715	RefSeq	Mus muscul	FALSE	80905	Mm.311585	Q9JJN0	chr17	GO:000073	DNA synthe	GO:000563	nucleus // n	GO:000367	DNA binding	○
17510685	395.2768	307.4504	1.28566039	0.3625096	0	main		NM_010332	Ednra	endothelin r	NM_010332	RefSeq	Mus muscul	FALSE	13617	Mm.283168	Q61614	chr8	GO:000156	patterning o	GO:000582	cytosol // pl	GO:000471	protein tyros	○
17285438	35.38058	27.60077	1.28186931	0.35824918	0	main		NM_008380	Inhba	inhibin beta-	NM_008380	RefSeq	Mus muscul	FALSE	16323	Mm.8042	Q04998	chr13	GO:000008	G1/S transit	GO:000557	extracellular	GO:000510	receptor bin	○
17479548	274.72	214.7377	1.27932822	0.35538645	0	main		NM_178070	Vps33b	vacuolar pro	NM_178070	RefSeq	Mus muscul	FALSE	233405	Mm.322870	P59016	chr7	GO:000681	transport //	GO:000573	cytoplasm /	GO:000551	protein bind	○
17289794	138.1331	108.8438	1.26909479	0.34379983	0	main		NM_152804	Plk2	polo-like kin	NM_152804	RefSeq	Mus muscul	FALSE	20620	Mm.380	P53351	chr13	GO:000008	G1/S transit	GO:000078	chromatin /	GO:000016	nucleotide b	○
17385840	34.32003	27.06227	1.26818741	0.34276796	0	main		NM_001267	Fign	fidgetin	NM_001267	RefSeq	Mus muscul	FALSE	60344	Mm.130769	Q9ERZ6	chr2	GO:000704	cell cycle //	GO:000563	nucleus // c	GO:000016	nucleotide b	○
17269695	200.6461	158.7477	1.26393075	0.33791742	0	main		NM_001113	Stat5b	signal transc	NM_001113	RefSeq	Mus muscul	FALSE	20851	Mm.34064	P42232	chr11	GO:000025	allantoin me	GO:000563	nucleus // n	GO:000097	RNA polyme	○
17224551	24.97091	19.93151	1.25283584	0.32519739	0	main		NM_008985	Ptprn	protein tyros	NM_008985	RefSeq	Mus muscul	FALSE	19275	Mm.2902	Q60673	chr1	GO:000030	response to	GO:000576	endosome /	GO:000472	protein tyros	○
17536501	46.371	37.42121	1.23916357	0.30936663	0	main		NM_001177	Eda	ectodysplas	NM_001177	RefSeq	Mus muscul	FALSE	13607	Mm.328086	O54693	chrX	GO:000194	hair follicle	GO:000557	extracellular	GO:000516	tumor necro	○
17397344	171.355	139.1975	1.23102067	0.29985499	0	main		NM_001130	Jade1	jade family F	NM_001130	RefSeq	Mus muscul	FALSE	269424	Mm.286285	Q6ZPI0	chr3	GO:000635	transcription	GO:000012	histone acet	GO:000110	RNA polyme	○
17367216	701.1299	570.6381	1.22867698	0.29710569	0	main		NM_026162	Plxdc2	plexin doma	NM_026162	RefSeq	Mus muscul	FALSE	67448	Mm.313938	Q9DC11	chr2	---	---	GO:000588	plasma men	---	---	○
17333222	64.18011	52.51259	1.22218519	0.28946291	0	main		NM_026644	Agpat4	1-acylglycer	NM_026644	RefSeq	Mus muscul	FALSE	68262	Mm.258300	Q8K4X7	chr17	GO:000662	lipid metabo	GO:001602	membrane	GO:000384	1-acylglycer	○
17384951	308.8128	252.7078	1.22201531	0.28926236	0	main		NM_001289	Zeb2	zinc finger E	NM_001289	RefSeq	Mus muscul	FALSE	24136	Mm.440702	Q9R0G7	chr2	GO:000012	negative reg	GO:000563	nucleus // n	GO:000120	transcription	○
17527565	129.0039	105.8284	1.21899131	0.28568784	0	main		NM_001304	Csk	c-src tyrosin	NM_001304	RefSeq	Mus muscul	FALSE	12988	Mm.21974	P41241	chr9	GO:000181	regulation o	GO:000573	cytoplasm /	GO:000016	nucleotide b	○
17211774	134.5085	110.7301	1.21474197	0.2806499	0	main		NM_145516	Plekhb2	pleckstrin hc	NM_145516	RefSeq	Mus muscul	FALSE	226971	Mm.292751	Q9QZC7	chr1	GO:000815	biological_p	GO:000576	endosome /	GO:000367	molecular_f	○
17509315	32.15852	26.58824	1.20950164	0.27441273	0	main		NM_001145	Tenm3	teneurin trar	NM_001145	RefSeq	Mus muscul	FALSE	23965	Mm.42191	Q9WTS6	chr8	GO:000715	cell adhesio	GO:000588	integral com	GO:004280	protein hom	○
17519649	148.765	123.3637	1.20590579	0.2701172	0	main		NM_010357	Gsta4	glutathione	NM_010357	RefSeq	Mus muscul	FALSE	14860	Mm.2662	P24472	chr9	GO:000815	metabolic pr	GO:000573	cytoplasm /	GO:000436	glutathione	○
17220724	329.8594	273.7484	1.20497289	0.26900069	0	main		NM_008976	Ptpn14	protein tyros	NM_008976	RefSeq	Mus muscul	FALSE	19250	Mm.4498	Q62130	chr1	GO:000194	lymphangiog	GO:000563	nucleus // n	GO:000371	transcription	○
17264651	232.019	193.7873	1.19728692	0.25976893	0	main		NM_001017	Kdm6b	KDM1 lysine	NM_001017	RefSeq	Mus muscul	FALSE	216850	Mm.261201	Q5NCY0	chr11	GO:000695	inflammator	GO:000563	nucleus // n	GO:000090	sulfonate di	○
17547634	528.2668	441.4713	1.19660508	0.25894709	0	main		NM_008379	Kpnb1	karyopherin	NM_008379	RefSeq	Mus muscul	FALSE	16211	Mm.251013	P70168	chr11	GO:000005	protein impo	GO:000563	nucleus // n	GO:000551	protein bind	○
17243525	68.13747	57.12748	1.19272669	0.25426349	0	main		NM_177388	Slc41a2	solute carrie	NM_177388	RefSeq	Mus muscul	FALSE	338365	Mm.67073	Q8BYR8	chr10	GO:000681	transport //	GO:000588	plasma men	GO:000832	cation trans	○
17218233	35.34816	29.63811	1.19265905	0.25418167	0	main		NM_177756	Colgalt2	collagen bet	NM_177756	RefSeq	Mus muscul	FALSE	269132	Mm.23782	Q6NVG7	chr1	GO:000815	biological_p	GO:000578	endoplasmic	GO:001674	transferase	○
17416540	53.59624	45.24218	1.18465202	0.24446335	0	main		NM_001080	Lrp8	low density l	NM_001080	RefSeq	Mus muscul	FALSE	16975	Mm.442134	Q924X6	chr4	GO:000012	negative reg	GO:000557	extracellular	GO:000194	glycoprotein	○
17531939	77.39584	65.39586	1.18349755	0.24305672	0	main		NM_175380	Gpd1l	glycerol-3-pl	NM_175380	RefSeq	Mus muscul	FALSE	333433	Mm.38198	Q3ULJ0	chr9	GO:000202	regulation o	GO:000573	cytoplasm /	GO:000436	glycerol-3-p	○
17402787	23.20444	19.70089	1.17783714	0.23614008	0	main		NM_021382	Tacr3	tachykinin re	NM_021382	RefSeq	Mus muscul	FALSE	21338	Mm.103810	P47937	chr3	GO:000716	signal trans	GO:000563	nucleus // c	GO:000487	signal trans	○
17420761	94.36894	80.15652	1.17730835	0.23549222	0	main		NM_001205	Iffo2	intermediate	NM_001205	RefSeq	Mus muscul	FALSE	212632	Mm.30464	Q8R2V2	chr4	GO:000815	biological_p	GO:000588	intermediate	GO:000519	structural m	○
17520292	14.8044	12.60399	1.17458043	0.23214551	0	main		NM_009576	Zic4	zinc finger p	NM_009576	RefSeq	Mus muscul	FALSE	22774	Mm.8062 //	Q61467	chr9	---	---	GO:000563	nucleus // n	GO:000367	nucleic acid	○
17541618	38.11409	32.47782	1.17354213	0.23086963	0	main		NM_001310	Mbnl3	muscleblind	NM_001310	RefSeq	Mus muscul	FALSE	171170	Mm.295324	Q8R003	chrX	GO:000639	mRNA proc	GO:000563	nucleus // c	GO:004482	poly(A) RN	○
17452456	93.01814	79.79966	1.16564582	0.22112949	0	main		NM_001199	Camkk2	calcium/caln	NM_001199	RefSeq	Mus muscul	FALSE	207565	Mm.289237	Q8C078	chr5	GO:000646	protein phos	GO:000563	nucleus // c	GO:000016	nucleotide b	○
17414009	57.36261	49.41135	1.16091971	0.2152682	0	main		NM_001145	Msantd3	Myb/SANT-l	NM_001145	RefSeq	Mus muscul	FALSE	66665	Mm.140749	Q9CR78	chr4	GO:000815	biological_p	GO:000557	cellular_com	GO:004280	identical pro	○
17439481	406.1239	350.084	1.16007558	0.2142188	0	main		NM_080708	Bmp2k	BMP2 induc	NM_080708	RefSeq	Mus muscul	FALSE	140780	Mm.281490	Q91Z96	chr5	GO:000646	protein phos	GO:000563	nucleus // n	GO:000016	nucleotide b	○
17451536	110.1686	95.00095	1.15965788	0.21369924	0	main		NM_029956	Mmab	methylmalon	NM_029956	RefSeq	Mus muscul	FALSE	77697	Mm.105182	Q9D273	chr5	GO:000923	cobalamin n	GO:000573	mitochondri	GO:000016	nucleotide b	○
17360260	45.8485	39.65516	1.15617993	0.20936593	0	main		NM_025696	Sorcs3	sortilin-relate	NM_025696	RefSeq	Mus muscul	FALSE	66673	Mm.70980	Q8VI51	chr19	GO:000761	learning // r	GO:001602	membrane	GO:000551	protein bind	○
17253215	37.5668	32.62972	1.15130623	0.20327162	0	main		NM_010484	Slc6a4	solute carrie	NM_010484	RefSeq	Mus muscul	FALSE	15567	Mm.300318	Q60857	chr11	GO:000681	transport //	GO:000576	endosome /	GO:000532	neurotransm	○
17221317	238.6837	207.8641	1.14826803	0.19945943	0	main		NM_001302	Ncoa2	nuclear rece	NM_001302	RefSeq	Mus muscul	FALSE	17978	Mm.2537	Q61026	chr1	GO:000012	negative reg	GO:000563	nucleus // n	GO:000097	RNA polyme	○
17470060	157.8423	137.5229	1.14775285	0.19881201	0	main		NM_178045	Rassf4	Ras associa	NM_178045	RefSeq	Mus muscul	FALSE	213391	Mm.257931	Q8CB96	chr6	GO:000704	cell cycle //	GO:000557	cellular_com	GO:000367	molecular_f	○
17461888	40.41676	35.33884	1.14369232	0.19369899	0	main		NM_001252	Hrh1	histamine re	NM_001252	RefSeq	Mus muscul	FALSE	15465	Mm.333327	P70174	chr6	GO:000695	inflammator	GO:000565	nucleoplasm	GO:000487	signal trans	○
17518616	125.8959	110.4689	1.13965016	0.18859103	0	main		NM_028030	Rbpms2	RNA binding	NM_028030	RefSeq	Mus muscul	FALSE	71973	Mm.29148	Q9VC52	chr9	GO:000815	biological_p	GO:000557	cellular_com	GO:000016	nucleotide b	○
17527735	201.2736	177.1278	1.13631852	0.18436729	0	main		NM_001042	Neo1	neogenin	NM_001042	RefSeq	Mus muscul	FALSE	18007	Mm.42249	P97798	chr9	GO:000635	regulation o	GO:000588	plasma men	GO:000487	receptor act	○
17268469	330.3497	291.0156	1.13516148	0.18289754	0	main		NM_008379	Kpnb1	karyopherin	NM_008379	RefSeq	Mus muscul	FALSE	16211	Mm.251013	P70168	chr11	GO:000005	protein impo	GO:000563	nucleus // n	GO:000551	protein bind	○
17255877	117.2174	103.8069	1.12918698	0.1752844	0	main		NM_010688	Lasp1	LIM and SH	NM_010688	RefSeq	Mus muscul	FALSE	16796	Mm.271967	Q61792	chr11	GO:000681	transport //	GO:000573	cytoplasm /	GO:000377	actin binding	○
17392529	30.51064	27.02555	1.12895538	0.17498846	0	main		NM_001077	Nkx2-2	NK2 homeo	NM_001077	RefSeq	Mus muscul	FALSE	18088	Mm.330639	P42586	chr2	GO:000332	type B panc	GO:000562	intracellular	GO:000098	RNA polyme	○
17517780	117.5948	104.2109	1.1284309	0.17431808	0	main		NM_153799	Edc3	enhancer of	NM_153799	RefSeq	Mus muscul	FALSE	353190	Mm.268104	Q8K2D3	chr9	GO:000950	RNA phosph	GO:000573	cytoplasm /	GO:000372	RNA binding	○
17240670	8.699137	7.713967	1.1277125	0.17339931	0	main		NM_001031	Lin28b	lin-28 homol	NM_001031	RefSeq	Mus muscul	FALSE	380669	Mm.440328	Q45KJ6	chr10	GO:000635	regulation o	GO:000563	nucleus // n	GO:000367	nucleic acid	○
17372066	23.66475	21.01071	1.12631843	0.17161477	0	main		NM_013555	Hoxd9	homeobox D	NM_013555	RefSeq	Mus muscul	FALSE	15438	Mm.26544	P28357	chr2	GO:000012	negative reg	GO:000563	nucleus // n	GO:000097	RNA polyme	○
17315152	171.9612	153.5162	1.12015019	0.16369218	0	main		NM_007395	Acvr1b	activin A rec	NM_007395	RefSeq	Mus muscul	FALSE	11479	Mm.308467	Q61271	chr15	GO:000008	G1/S transit	GO:000588	plasma men	GO:000016	nucleotide b	○
17500263	55.59924	49.6639	1.11951015	0.1628676	0	main		NM_199042	Thap1	THAP doma	NM_199042	RefSeq	Mus muscul	FALSE	73754	Mm.383241	Q8CHW1	chr8	GO:000193	endothelial	GO:000563	nucleus // n	GO:000367	nucleic acid	○
17348138	399.2838	356.77	1.11916305	0.16242023	0	main		NM_001081	9430020K0	RIKEN cDN	NM_001081	RefSeq	Mus muscul	FALSE	240185	Mm.33179	Q5DTX6	chr18	GO:000715	cell adhesio	GO:000591	cell-cell junc	GO:000367	molecular_f	○
17376822	38.63621	34.53436	1.11877591	0.16192109	0	main		NM_001291	Snap25	synaptosom	NM_001291	RefSeq	Mus muscul	FALSE	20614	Mm.45953	P60879	chr2	GO:000688	exocytosis	GO:000573	cytoplasm /	GO:000014	SNARE bin	○
17396765	22.47008	20.128	1.1163593	0.15880143	0	main		NM_001013	Usp13	ubiquitin spe	NM_001013	RefSeq	Mus muscul	FALSE	72607	Mm.316153	Q5BKP2	chr3	GO:000635	regulation o	GO:000576	lysosome //	GO:000419	cysteine-typ	○
17490995	25.4631	22.82328	1.11566348	0.15790193	0	main		NM_008172	Grin2d	glutamate re	NM_008172	RefSeq	Mus muscul	FALSE	14814	Mm.322594	Q03391	chr7	GO:000196	startle respo	GO:000588	plasma men	GO:000487	receptor act	○
17428501	26.00614	23.34089	1.11418802	0.15599271	0	main		NM_001025	Dmbx1	diencephalo	NM_001025	RefSeq	Mus muscul	FALSE	140477	Mm.213206	Q91ZK4	chr4	GO:000012	negative reg	GO:000563	nucleus // tr	GO:000097	RNA polyme	○
17276642	333.1916	299.0916	1.11401189	0.15576464	0	main		NM_001252	Fut8	fucosyltrans	NM_001252	RefSeq	Mus muscul	FALSE	53618	Mm.35628	Q9WTS2	chr12	GO:000648	protein N-lin	GO:000573	cytoplasm /	GO:000842	glycoprotein	○
17511868	199.3775	179.3222	1.11183947	0.1529485	0	main		NM_028221	Fam192a	family with s	NM_028221	RefSeq	Mus muscul	FALSE	102122	Mm.250425	Q91WE2	chr8	GO:000815	biological_p	GO:000563	nucleus // n	GO:000367	molecular_f	○
17344472	80.75047	72.66191	1.11131775	0.15227137	0	main		NM_001142	Atat1	alpha tubulir	NM_001142	RefSeq	Mus muscul	FALSE	73242	Mm.273155	Q8K341	chr17	GO:000728	spermatoge	GO:000573	cytoplasm /	GO:001674	transferase	○
17321094	50.50181	45.45639	1.11099474	0.15185198	0	main		NM_001113	Col2a1	collagen, typ	NM_001113	RefSeq	Mus muscul	FALSE	12824	Mm.2423	P28481	chr15	GO:000150	skeletal syst	GO:000557	extracellular	GO:000520	extracellular	○
17539888	57.53815	51.92461	1.10810943	0.14810036	0	main		NM_018832	Magix	MAGI family	NM_018832	RefSeq	Mus muscul	FALSE	54634	Mm.67912	Q4KL35	chrX	GO:000815	biological_p	GO:000557	cellular_com	GO:000367	molecular_f	○
17227384	21.27916	19.21104	1.10765268	0.14750558	0	main		NM_001313	Pkp1	plakophilin 1	NM_001313	RefSeq	Mus muscul	FALSE	18772	Mm.4494	P97350	chr1	GO:000715	cell adhesio	GO:000563	nucleus // n	GO:000551	protein bind	○
17382496	49.19826	44.44615	1.10691837	0.14654884	0	main		NM_001177	Grin1	glutamate re	NM_001177	RefSeq	Mus muscul	FALSE	14810	Mm.278672	P35438	chr2	GO:000166	conditioned	GO:000573	cytoplasm /	GO:000487	receptor act	○
17261470	196.0684	177.5807	1.10410872	0.14288224	0	main		NM_001291	Rhbdf1	rhomboid fa	NM_001291	RefSeq	Mus muscul	FALSE	13650	Mm.11545	Q6PIX5	chr11	GO:000650	proteolysis	GO:000013	Golgi memb	GO:000425	serine-type	○
17544446	20.29059	18.3815	1.10385932	0.14255631	0	main		NM_183319	Xkrx	Kell blood gr	NM_183319	RefSeq	Mus muscul	FALSE	331524	Mm.235055	Q5GH68	chrX	GO:000815	biological_p	GO:001602	membrane	GO:000367	molecular_f	○
17255402	400.3978	363.2942	1.102131	0.14029571	0	main		NM_025287	Spop	speckle-type	NM_025287	RefSeq	Mus muscul	FALSE	20747	Mm.285454	Q6ZWS8	chr11	GO:000012	negative reg	GO:000563	nucleus // n	GO:000108	RNA polyme	○
17448693	20.07113	18.25478	1.09949997	0.13684756	0	main		NM_001122	Txk	TXK tyrosine	NM_001122	RefSeq	Mus muscul	FALSE	22165	Mm.3264	P42682	chr5	GO:000181	cytokine pro	GO:000563	nucleus // c	GO:000016	nucleotide b	○
17413384	177.1373	161.1458	1.09923622	0.13650145	0	main		NM_172866	Rgp1	RGP1 retrog	NM_172866	RefSeq	Mus muscul	FALSE	242406	Mm.259311	Q8BHT7	chr4	GO:004214	retrograde t	GO:000573	cytoplasm /	GO:000508	guanyl-nucl	○
17394024	38.52722	35.08982	1.09796004	0.13482554	0	main		XM_006500	Oser1	oxidative str	XM_006500	RefSeq	PREDICTED	FALSE	66680	Mm.25119	Q9D722	chr2	GO:007030	cellular resp	GO:000557	cellular_com	GO:000367	molecular_f	○
17214841	405.0603	369.881	1.09510978	0.1310755	0	main		NM_001310	Agfg1	ArfGAP with	NM_001310	RefSeq	Mus muscul	FALSE	15463	Mm.433409	Q8K2K6	chr1	GO:000167	acrosome a	GO:000563	nucleus // c	GO:000367	DNA binding	○
17262964	23.3643	21.34819	1.09443939	0.13019206	0	main		NM_001029	Fat2	FAT tumor s	NM_001029	RefSeq	Mus muscul	FALSE	245827	Mm.339363	Q5F226	chr11	GO:000715	cell adhesio	GO:000563	nucleus // p	GO:000550	calcium ion	○
17466798	37.22743	34.13578	1.09056919	0.12508131	0	main		NM_001311	Hoxa1	homeobox A	NM_001311	RefSeq	Mus muscul	FALSE	15394	Mm.197	P09022	chr6	GO:000635	transcription	GO:000563	nucleus	GO:000367	DNA binding	○
17261494	109.2224	100.2062	1.08997647	0.12429699	0	main		NM_001284	Nprl3	nitrogen per	NM_001284	RefSeq	Mus muscul	FALSE	17168	---	Q8VIJ8	chr11	GO:000328	ventricular s	GO:199013	Iml1 comple	GO:000509	GTPase act	○
17328201	196.641	181.0224	1.08627993	0.11939593	0	main		NM_001130	Gspt1	G1 to S pha	NM_001130	RefSeq	Mus muscul	FALSE	14852	Mm.325827	Q8R050	chr16	GO:000018	nuclear-tran	GO:000582	cytosol // tra	GO:000016	nucleotide b	○
17347281	242.8026	223.8336	1.08474599	0.11735725	0	main		NM_011500	Strn	striatin, calm	NM_011500	RefSeq	Mus muscul	FALSE	268980	Mm.391038	O55106	chr17	GO:000762	locomotory	GO:000015	protein phos	GO:000551	calmodulin l	○
17456627	70.0621	64.621	1.08420018	0.11663116	0	main		NM_001081	Flnc	filamin C, ga	NM_001081	RefSeq	Mus muscul	FALSE	68794	Mm.39046	Q8VHX6	chr6	GO:003002	actin filamer	GO:000573	cytoplasm /	GO:000377	actin binding	○
17528714	198.0108	183.0559	1.08169581	0.11329485	0	main		NM_001253	Tcf12	transcription	NM_001253	RefSeq	Mus muscul	FALSE	21406	Mm.171615	Q61286	chr9	GO:000635	transcription	GO:000079	nuclear chrc	GO:000097	RNA polyme	○
17325014	334.1208	309.1382	1.08081369	0.11211786	0	main		NM_080557	Snx4	sorting nexin	NM_080557	RefSeq	Mus muscul	FALSE	69150	Mm.28196	Q91YJ2	chr16	GO:000681	transport //	GO:000573	cytoplasm /	GO:000515	epidermal g	○
17219612	50.86245	47.09312	1.08003993	0.11108465	0	main		NM_177723	Vsig8	V-set and im	NM_177723	RefSeq	Mus muscul	FALSE	240916	Mm.71940	Q6P3A4	chr1	GO:000815	biological_p	GO:000562	intracellular	GO:004482	poly(A) RNA	○
17442970	283.8113	262.8956	1.07955896	0.11044204	0	main		NM_001001	Vkorc1l1	vitamin K ep	NM_001001	RefSeq	Mus muscul	FALSE	69568	Mm.288718	Q6TEK5	chr5	GO:001718	peptidyl-glut	GO:000578	endoplasmic	GO:001649	oxidoreduct	○
17405142	160.3999	148.8133	1.07785998	0.10816977	0	main		NM_080793	Setd7	SET domain	NM_080793	RefSeq	Mus muscul	FALSE	73251	Mm.192111	Q8VHL1	chr3	GO:000635	transcription	GO:000563	nucleus // c	GO:000203	p53 binding	○
17496251	60.67113	56.3889	1.07594101	0.10559898	0	main		NM_001289	Sh2b1	SH2B adapt	NM_001289	RefSeq	Mus muscul	FALSE	20399	Mm.8538	Q91ZM2	chr7	GO:000692	movement c	GO:000172	ruffle // nucl	GO:000487	signal trans	○
17525198	26.96492	25.15817	1.07181564	0.10005677	0	main		NM_172290	Ntm	neurotrimin	NM_172290	RefSeq	Mus muscul	FALSE	235106	Mm.283138	Q99PJ0	chr9	GO:000715	cell adhesio	GO:000561	extracellular	---	---	○
17439164	359.6101	338.1787	1.063373	0.08864775	0	main		NM_001009	Sept11	septin 11	NM_001009	RefSeq	Mus muscul	FALSE	52398	Mm.428571	Q8C1B7	chr5	GO:000704	cell cycle //	GO:000172	stress fiber	GO:000016	nucleotide b	○
17542047	21.99744	20.70273	1.06253813	0.08751461	0	main		NM_178740	Slitrk4	SLIT and NT	NM_178740	RefSeq	Mus muscul	FALSE	245446	Mm.101707	Q810B8	chrX	GO:000740	axonogenes	GO:001602	membrane	GO:000367	molecular_f	○
17321326	116.0284	109.3113	1.06144927	0.08603543	0	main		NM_007478	Arf3	ADP-ribosyl	NM_007478	RefSeq	Mus muscul	FALSE	11842	Mm.221298	P61205	chr15	GO:000681	transport //	GO:000562	intracellular	GO:000016	nucleotide b	○
17424884	20.29303	19.1464	1.0598875	0.08391114	0	main		NM_008782	Pax5	paired box 5	NM_008782	RefSeq	Mus muscul	FALSE	18507	Mm.439659	Q02650	chr4	GO:000012	negative reg	GO:000563	nucleus // tr	GO:000097	RNA polyme	○
17341681	90.18329	85.18712	1.05864936	0.08222483	0	main		NM_027008	Kctd5	potassium c	NM_027008	RefSeq	Mus muscul	FALSE	69259	Mm.28171	Q8VC57	chr17	GO:005126	protein hom	GO:000573	cytoplasm /	GO:003240	protein com	○
17400521	23.16278	21.90077	1.057624	0.08082682	0	main		NM_022030	Sv2a	synaptic ves	NM_022030	RefSeq	Mus muscul	FALSE	64051	Mm.491135	Q9JIS5	chr3	GO:000681	transport //	GO:000573	cytoplasm /	GO:000487	receptor act	○
17530782	34.54357	32.76868	1.05416422	0.07609963	0	main		NM_029103	Manf	mesenceph	NM_029103	RefSeq	Mus muscul	FALSE	74840	Mm.29778	Q9CXI5	chr9	GO:000201	vasoconstric	GO:000557	extracellular	GO:000808	growth facto	○
17266498	232.713	221.2159	1.0519723	0.07309672	0	main		NM_008702	Nlk	nemo like ki	NM_008702	RefSeq	Mus muscul	FALSE	18099	Mm.9001	O54949	chr11	GO:000635	transcription	GO:000563	nucleus // c	GO:000016	nucleotide b	○
17219127	17.12559	16.3053	1.05030818	0.07081271	0	main		NM_033652	Lmx1a	LIM homeob	NM_033652	RefSeq	Mus muscul	FALSE	110648	Mm.330081	Q9JKU8	chr1	GO:000155	regulation o	GO:000563	nucleus	GO:000367	DNA binding	○
17223793	64.12533	61.05568	1.05027624	0.07076883	0	main		NM_001042	Fzd5	frizzled hom	NM_001042	RefSeq	Mus muscul	FALSE	14367	Mm.150813	Q9EQD0	chr1	GO:000057	embryonic a	GO:000576	early endos	GO:000487	signal trans	○
17465296	9.068828	8.636144	1.05010153	0.07052882	0	main		NR_030420	Mir592	microRNA 5	NR_030420	RefSeq	Mus muscul	FALSE	735266	---	P47743	chr6	---	---	---	---	---	---	○
17469121	82.14896	78.25469	1.04976405	0.07006509	0	main		NM_001081	Prickle2	prickle homo	NM_001081	RefSeq	Mus muscul	FALSE	243548	Mm.248820	Q80Y24	chr6	GO:003117	neuron proje	GO:000563	nucleus // c	GO:000551	protein bind	○
17491788	102.3853	97.62957	1.04871198	0.06861851	0	main		NM_021366	Klf13	Kruppel-like	NM_021366	RefSeq	Mus muscul	FALSE	50794	Mm.240473	Q9JJZ6	chr7	GO:000635	transcription	GO:000563	nucleus // n	GO:000097	RNA polyme	○
17415943	82.25693	78.60956	1.04639856	0.06543245	0	main		NM_001164	Dnajc6	DnaJ (Hsp4	NM_001164	RefSeq	Mus muscul	FALSE	72685	Mm.76494	Q80TZ3	chr4	GO:007231	clathrin coat	GO:004520	synapse // n	GO:000472	phosphopro	○
17395766	35.9902	34.39535	1.04636819	0.06539058	0	main		NM_007906	Eef1a2	eukaryotic tr	NM_007906	RefSeq	Mus muscul	FALSE	13628	Mm.2645	P62631	chr2	GO:000641	translation	GO:000563	nucleus // c	GO:000016	nucleotide b	○
17317015	268.6235	257.2965	1.04402314	0.06215369	0	main		NM_010162	Ext1	exostoses (r	NM_010162	RefSeq	Mus muscul	FALSE	14042	Mm.309395	P97464	chr15	GO:000150	ossification	GO:000578	endoplasmic	GO:000837	acetylglucos	○
17328792	163.925	157.7459	1.03917122	0.05543339	0	main		NM_001109	Dgcr2	DiGeorge sy	NM_001109	RefSeq	Mus muscul	FALSE	13356	Mm.254515	P98154	chr16	GO:000715	cell adhesio	GO:001602	membrane	GO:003024	carbohydrat	○
17427164	36.25897	34.92921	1.03807014	0.05390393	0	main		NM_001177	Elavl2	ELAV (embr	NM_001177	RefSeq	Mus muscul	FALSE	15569	Mm.318042	Q60899	chr4	---	---	---	---	GO:000016	nucleotide b	○
17365106	60.20166	58.36703	1.03143264	0.04464961	0	main		NM_001033	Sec31b	Sec31 homo	NM_001033	RefSeq	Mus muscul	FALSE	240667	Mm.329873	Q3TZ89	chr19	GO:000681	transport //	GO:000573	cytoplasm /	GO:000367	molecular_f	○
17270648	450.7456	437.1094	1.03119631	0.04431901	0	main		NM_001285	Cdc27	cell division	NM_001285	RefSeq	Mus muscul	FALSE	217232	Mm.89845	A2A6Q5	chr11	GO:000709	metaphase/	GO:000563	nucleus // n	GO:001990	protein phos	○
17449526	520.3524	504.9713	1.03045935	0.0432876	0	main		NM_030886	Ankrd17	ankyrin repe	NM_030886	RefSeq	Mus muscul	FALSE	81702	Mm.245522	Q99NH0	chr5	GO:000195	blood vesse	GO:000078	chromatin /	GO:000367	nucleic acid	○
17290111	76.51958	74.27445	1.03022749	0.04296294	0	main		NM_008002	Fgf10	fibroblast gr	NM_008002	RefSeq	Mus muscul	FALSE	14165	Mm.317323	O35565	chr13	GO:000013	establishme	GO:000557	extracellular	GO:000510	receptor bin	○
17313284	406.896	395.5622	1.02865238	0.04075553	0	main		NM_177821	Ep300	E1A binding	NM_177821	RefSeq	Mus muscul	FALSE	328572	Mm.258397	B2RWS6	chr15	GO:000012	negative reg	GO:000012	histone acet	GO:000097	RNA polyme	○
17252113	161.4595	157.0524	1.02806133	0.03992634	0	main		NM_001045	Mink1	misshapen-l	NM_001045	RefSeq	Mus muscul	FALSE	50932	Mm.42967	Q9JM52	chr11	GO:000016	MAPK casc	GO:000573	cytoplasm /	GO:000016	nucleotide b	○
17537756	166.7148	162.38	1.02669541	0.03800823	0	main		NM_027870	Armcx3	armadillo re	NM_027870	RefSeq	Mus muscul	FALSE	71703	Mm.67949	Q8BHS6	chrX	GO:003461	cellular prot	GO:001602	membrane	GO:000551	protein bind	○
17219362	347.3684	338.966	1.02478833	0.03532595	0	main		NM_172647	F11r	F11 recepto	NM_172647	RefSeq	Mus muscul	FALSE	16456	Mm.294882	O88792	chr1	GO:000181	regulation o	GO:000588	plasma men	GO:000551	protein bind	○
17414301	341.8171	334.686	1.02130684	0.03041637	0	main		NM_009011	Rad23b	RAD23b hor	NM_009011	RefSeq	Mus muscul	FALSE	19359	Mm.196846	P54728	chr4	GO:000071	nucleotide-e	GO:000050	proteasome	GO:000368	damaged D	○
17430777	91.94738	90.25462	1.01875538	0.02680768	0	main		NM_001122	Gmeb1	glucocortico	NM_001122	RefSeq	Mus muscul	FALSE	56809	Mm.103489	Q9JL60	chr4	GO:000635	transcription	GO:000563	nucleus // c	GO:000097	RNA polyme	○
17466982	78.46453	77.23427	1.01592894	0.02279949	0	main		NM_027268	Scrn1	secernin 1	NM_027268	RefSeq	Mus muscul	FALSE	69938	Mm.41557	Q9CZC8	chr6	GO:000650	proteolysis	GO:000563	nucleus // c	GO:001680	dipeptidase	○
17452311	43.6625	42.98427	1.01577856	0.02258593	0	main		NM_001312	Cux2	cut-like hom	NM_001312	RefSeq	Mus muscul	FALSE	13048	Mm.332661	P70298	chr5	GO:000012	negative reg	GO:000563	nucleus // n	GO:000097	RNA polyme	○
17510013	88.38013	87.37492	1.01150456	0.01650282	0	main		NM_001122	Upf1	UPF1 regula	NM_001122	RefSeq	Mus muscul	FALSE	19704	Mm.390048	Q9EPU0	chr8	GO:000018	nuclear-tran	GO:000078	chromatin /	GO:000016	nucleotide b	○
17441140	29.12252	28.80253	1.01110979	0.01593966	0	main		NM_008629	Msi1	musashi RN	NM_008629	RefSeq	Mus muscul	FALSE	17690	Mm.5077	Q61474	chr5	GO:000972	response to	GO:000563	nucleus // c	GO:000016	nucleotide b	○
17247448	147.461	145.9767	1.01016806	0.01459533	0	main		NM_024459	Ppp3r1	protein phos	NM_024459	RefSeq	Mus muscul	FALSE	19058	Mm.41840	Q63810	chr11	GO:000156	patterning o	GO:000573	cytoplasm /	GO:000472	phosphopro	○
17245334	175.1239	173.4036	1.00992079	0.01424214	0	main		NM_001014	Dyrk2	dual-specific	NM_001014	RefSeq	Mus muscul	FALSE	69181	Mm.458174	Q5U4C9	chr10	GO:000646	protein phos	GO:000015	ubiquitin liga	GO:000016	nucleotide b	○
17537643	29.89347	29.60971	1.00958334	0.01376001	0	main		NM_026239	Tmem35	transmembr	NM_026239	RefSeq	Mus muscul	FALSE	67564	Mm.41489	Q9D328	chrX	GO:000815	biological_p	GO:000577	peroxisome	GO:000367	molecular_f	○
17223766	256.781	254.5552	1.00874388	0.01255992	0	main		NM_033563	Klf7	Kruppel-like	NM_033563	RefSeq	Mus muscul	FALSE	93691	Mm.29466	Q99JB0	chr1	GO:000635	transcription	GO:000563	nucleus	GO:000367	nucleic acid	○
17495340	98.28793	97.46153	1.00847924	0.01218139	0	main		NM_001025	Sox6	SRY (sex de	NM_001025	RefSeq	Mus muscul	FALSE	20679	Mm.323365	P40645	chr7	GO:000012	negative reg	GO:000563	nucleus // n	GO:000367	DNA binding	○
17410410	124.8168	123.7928	1.00827189	0.01188472	0	main		NM_028943	Sgms2	sphingomye	NM_028943	RefSeq	Mus muscul	FALSE	74442	Mm.273360	Q9D4B1	chr3	GO:000662	lipid metabo	GO:000579	Golgi appara	GO:001630	kinase activ	○
17438903	26.51221	26.33271	1.00681662	0.00980093	0	main		NM_007423	Afp	alpha fetopr	NM_007423	RefSeq	Mus muscul	FALSE	11576	Mm.358570	P02772	chr5	GO:000154	ovulation fro	GO:000557	extracellular	GO:000456	beta-glucuro	○
17371912	141.153	140.2539	1.00641052	0.0092189	0	main		NM_001164	Zak	sterile alpha	NM_001164	RefSeq	Mus muscul	FALSE	65964	Mm.314618	Q9ESL4	chr2	GO:000007	cell cycle ch	GO:000563	nucleus // c	GO:000016	nucleotide b	○
17378640	109.0877	108.5014	1.00540362	0.00777478	0	main		NM_001042	Dlgap4	discs, large	NM_001042	RefSeq	Mus muscul	FALSE	228836	Mm.22094	B1AZP2	chr2	GO:002305	signaling //	GO:000588	plasma men	GO:000551	protein bind	○
17496237	73.04269	72.6756	1.00505108	0.00726882	0	main		NM_001081	Sh2b1	SH2B adapt	NM_001081	RefSeq	Mus muscul	FALSE	20399	Mm.8538	Q91ZM2	chr7	GO:000692	movement c	GO:000172	ruffle // nucl	GO:000487	signal trans	○
17355962	187.259	186.7519	1.00271537	0.00391214	0	main		NM_001167	Suv420h1	suppressor	NM_001167	RefSeq	Mus muscul	FALSE	225888	Mm.278578	Q3U8K7	chr19	GO:000635	transcription	GO:000078	condensed	GO:000816	methyltrans	○
17323659	100.0878	99.98786	1.00099952	0.00144128	0	main		NM_001080	Trmt2a	TRM2 tRNA	NM_001080	RefSeq	Mus muscul	FALSE	15547	Mm.271798	Q8BNV1	chr16	GO:000639	RNA proces	---	---	GO:000016	nucleotide b	○
17239090	321.2226	321.9822	0.99764086	-0.0034075	0	main		NM_138667	Tab2	TGF-beta ac	NM_138667	RefSeq	Mus muscul	FALSE	68652	Mm.193041	Q99K90	chr10	GO:000750	heart develo	GO:000563	nucleus // c	GO:000551	protein bind	○
17226676	110.6904	111.1392	0.99596182	-0.0058377	0	main		NM_001162	Pfkfb2	6-phosphofr	NM_001162	RefSeq	Mus muscul	FALSE	18640	Mm.249861	P70265	chr1	GO:000600	glucose cata	GO:000573	cytoplasm	GO:000387	6-phosphofr	○
17531154	29.37538	29.54562	0.99423806	-0.0083368	0	main		NM_007567	Bsn	bassoon	NM_007567	RefSeq	Mus muscul	FALSE	12217	Mm.20425	O88737	chr9	GO:000741	synapse ass	GO:000573	cytoplasm /	GO:000551	protein bind	○
17238449	183.9187	185.0954	0.99364274	-0.0092009	0	main		NM_172790	Ankrd52	ankyrin repe	NM_172790	RefSeq	Mus muscul	FALSE	237615	Mm.440177	Q8BTI7	chr10	GO:000815	biological_p	GO:000557	cellular_com	GO:000367	molecular_f	○
17349465	147.2714	148.6045	0.99102921	-0.0130005	0	main		NM_175104	Fam53c	family with s	NM_175104	RefSeq	Mus muscul	FALSE	66306	Mm.29424	Q8BXQ8	chr18	GO:000815	biological_p	GO:000557	cellular_com	GO:000367	molecular_f	○
17513297	86.73388	87.64886	0.98956085	-0.0151397	0	main		NM_029441	Cdyl2	chromodom	NM_029441	RefSeq	Mus muscul	FALSE	75796	Mm.159575	Q9D5D8	chr8	GO:000815	metabolic pr	GO:000563	nucleus // n	GO:000382	catalytic act	○
17281388	54.38596	55.14585	0.98622036	-0.0200181	0	main		NM_001033	Fbxo33	F-box protei	NM_001033	RefSeq	Mus muscul	FALSE	70611	Mm.311026	Q8VE08	chr12	GO:000815	biological_p	GO:000557	cellular_com	GO:000367	molecular_f	○
17424880	18.12428	18.37914	0.98613319	-0.0201456	0	main		XM_006537	Pax5	paired box 5	XM_006537	RefSeq	PREDICTED	FALSE	18507	Mm.439659	Q02650	chr4	GO:000012	negative reg	GO:000563	nucleus // tr	GO:000097	RNA polyme	○
17273700	71.29449	72.34348	0.98549987	-0.0210724	0	main		NM_153082	Dnajc27	DnaJ (Hsp4	NM_153082	RefSeq	Mus muscul	FALSE	217378	Mm.271656	Q8CFP6	chr12	GO:000688	intracellular	GO:000563	nucleus // n	GO:000016	nucleotide b	○
17395985	101.3153	102.8577	0.98500453	-0.0217977	0	main		NM_030708	Zfhx4	zinc finger h	NM_030708	RefSeq	Mus muscul	FALSE	80892	Mm.41522	Q9JJN2	chr3	GO:000635	transcription	GO:000563	nucleus // n	GO:000367	nucleic acid	○
17440974	47.90542	48.6448	0.98480043	-0.0220967	0	main		NM_001004	Fam222a	family with s	NM_001004	RefSeq	Mus muscul	FALSE	433940	Mm.194212	Q6PGH4	chr5	GO:000815	biological_p	GO:000557	cellular_com	GO:000367	molecular_f	○
17471541	67.25922	68.38022	0.98360637	-0.023847	0	main		NM_001309	Clec7a	C-type lectir	NM_001309	RefSeq	Mus muscul	FALSE	56644	Mm.239516	Q6QLQ4	chr6	GO:000222	pattern reco	GO:000588	plasma men	GO:000551	protein bind	○
17264424	209.7572	213.3341	0.98323334	-0.0243943	0	main		NM_023668	Ndel1	nuclear distr	NM_023668	RefSeq	Mus muscul	FALSE	83431	Mm.31979	Q9ERR1	chr11	GO:000013	establishme	GO:000077	chromosom	GO:000551	protein bind	○
17467608	163.3382	166.2211	0.98265623	-0.0252413	0	main		NM_024288	Rmnd5a	required for	NM_024288	RefSeq	Mus muscul	FALSE	68477	Mm.28474	Q80YQ8	chr6	GO:000815	biological_p	GO:000557	cellular_com	GO:000367	molecular_f	○
17265657	64.65639	65.81596	0.98238163	-0.0256445	0	main		NM_177776	Smtnl2	smoothelin-l	NM_177776	RefSeq	Mus muscul	FALSE	276829	Mm.46732	Q8CI12	chr11	GO:000815	biological_p	GO:000557	cellular_com	GO:000367	molecular_f	○
17366451	117.3319	119.4984	0.98187005	-0.026396	0	main		NM_001177	Frmd4a	FERM doma	NM_001177	RefSeq	Mus muscul	FALSE	209630	Mm.37932	Q8BIE6	chr2	GO:009016	establishme	GO:000573	cytoplasm /	GO:003067	protein bind	○
17392578	33.94569	34.67097	0.97908106	-0.0304998	0	main		NM_019632	Napb	N-ethylmale	NM_019632	RefSeq	Mus muscul	FALSE	17957	Mm.274308	P28663	chr2	GO:000681	transport //	GO:000577	vacuolar me	GO:000548	soluble NSF	○

17285000	506.2545	517.7437	0.9778091	-0.0323753	0	main		NM_011803	Klf6	Kruppel-like	NM_011803	RefSeq	Mus muscul	FALSE	23849	Mm.275036	O08584	chr13	GO:000635	transcription	GO:000563	nucleus // n	GO:000367	nucleic acid	O
17223412	149.894	153.7217	0.97509981	-0.0363782	0	main		NM_001310	Fam126b	family with s	NM_001310	RefSeq	Mus muscul	FALSE	213056	Mm.26272	Q8C729	chr1	GO:000815	biological_p	GO:000562	intracellular	GO:000367	molecular_f	O
17309041	63.29967	64.95107	0.97457471	-0.0371553	0	main		NM_001038	Dach1	dachshund	NM_001038	RefSeq	Mus muscul	FALSE	13134	Mm.320593	Q9QYB2	chr14	GO:000012	negative reg	GO:000563	nucleus // n	GO:000107	transcription	O
17266452	25.60396	26.29961	0.97354904	-0.0386744	0	main		NM_001168	Sarm1	sterile alpha	NM_001168	RefSeq	Mus muscul	FALSE	237868	Mm.210332	Q6PDS3	chr11	GO:000237	immune sys	GO:000573	cytoplasm /	GO:000551	protein bind	O
17400072	34.16462	35.14649	0.9720635	-0.0408775	0	main		NM_028307	Tdrkh	tudor and K	NM_028307	RefSeq	Mus muscul	FALSE	72634	Mm.331198	Q80VL1	chr3	GO:000714	male meiosi	GO:000573	cytoplasm /	GO:000367	nucleic acid	O
17286595	351.7958	362.054	0.97166666	-0.0414666	0	main		NM_001039	Rreb1	ras responsi	NM_001039	RefSeq	Mus muscul	FALSE	68750	Mm.491109	Q3UH06	chr13	GO:000012	negative reg	GO:000563	nucleus // n	GO:000097	RNA polyme	O
17472089	30.72753	31.64303	0.97106788	-0.042356	0	main		NM_008171	Grin2b	glutamate re	NM_008171	RefSeq	Mus muscul	FALSE	14812	Mm.436649	Q01097	chr6	GO:000150	action poten	GO:000588	plasma men	GO:000487	receptor act	O
17297196	173.3384	178.9966	0.96838934	-0.0463409	0	main		NM_139227	Atxn7	ataxin 7	NM_139227	RefSeq	Mus muscul	FALSE	246103	Mm.133625	Q8R4I1	chr14	GO:000022	microtubule	GO:000563	nucleus // n	GO:000368	chromatin b	O
17260786	41.92485	43.36451	0.96680096	-0.0487092	0	main		NM_026576	Etaa1	Ewing tumor	NM_026576	RefSeq	Mus muscul	FALSE	68145	Mm.178684	Q5SVT3	chr11	GO:000815	biological_p	GO:000573	cytoplasm	GO:000367	molecular_f	O
17321202	69.5806	72.38771	0.96122118	-0.0570597	0	main		NM_173769	Zfp641	zinc finger p	NM_173769	RefSeq	Mus muscul	FALSE	239652	Mm.207453	Q8BZ34	chr15	GO:000635	transcription	GO:000562	intracellular	GO:000098	RNA polyme	O
17465212	349.9285	364.1455	0.96095791	-0.0574548	0	main		NM_001167	Wasl	Wiskott-Aldr	NM_001167	RefSeq	Mus muscul	FALSE	73178	Mm.1574	Q91YD9	chr6	GO:000635	transcription	GO:000013	Golgi memb	GO:000377	actin binding	O
17415035	40.47544	42.14399	0.96040835	-0.0582801	0	main		NM_001081	Ccdc171	coiled-coil d	NM_001081	RefSeq	Mus muscul	FALSE	320226	Mm.180032	E9Q1U1	chr4	GO:000815	biological_p	GO:000557	cellular_com	GO:000367	molecular_f	O
17447510	194.5101	202.6133	0.96000657	-0.0588838	0	main		NM_026242	Mrfap1	Morf4 family	NM_026242	RefSeq	Mus muscul	FALSE	67568	Mm.490257	Q9CQL7	chr5	GO:000815	biological_p	GO:000563	nucleus // n	GO:000551	protein bind	O
17427746	155.1076	161.5786	0.95995138	-0.0589668	0	main		NM_178143	Prkaa2	protein kinas	NM_178143	RefSeq	Mus muscul	FALSE	108079	Mm.48638	Q8BRK8	chr4	GO:000635	transcription	GO:000563	nucleus // n	GO:000016	nucleotide b	O
17399817	37.35225	38.92988	0.95947509	-0.0596827	0	main		NM_199422	S100a7a	S100 calciu	NM_199422	RefSeq	Mus muscul	FALSE	381493	Mm.291525	Q6S5I3	chr3	GO:000695	inflammatory	GO:000561	extracellular	GO:000550	calcium ion	O
17384787	128.3552	133.9064	0.95854418	-0.0610832	0	main		NM_178778	Scai	suppressor	NM_178778	RefSeq	Mus muscul	FALSE	320271	Mm.204836	Q8C8N2	chr2	GO:000635	transcription	GO:000563	nucleus // n	GO:000371	transcription	O
17343735	163.8833	171.1077	0.95777864	-0.0622358	0	main		NM_013543	H2-Ke6	H2-K region	NM_013543	RefSeq	Mus muscul	FALSE	14979	Mm.275452	P50171	chr17	GO:000662	lipid metabo	GO:000573	mitochondri	GO:000025	C-3 sterol d	O
17423701	14.4964	15.14345	0.95727196	-0.0629992	0	main		NM_010243	Fut9	fucosyltrans	NM_010243	RefSeq	Mus muscul	FALSE	14348	Mm.39101	O88819	chr4	GO:000648	protein glyc	GO:000579	Golgi appara	GO:000000	alpha-1,6-m	O
17293237	67.60615	70.70172	0.95621648	-0.0645908	0	main		NM_001035	Golm1	golgi membr	NM_001035	RefSeq	Mus muscul	FALSE	105348	Mm.171335	Q91XA2	chr13	GO:000699	nucleus orga	GO:000561	extracellular	GO:000367	molecular_f	O
17294267	24.23967	25.37928	0.95509683	-0.0662811	0	main		NM_028878	Slc6a19	solute carrie	NM_028878	RefSeq	Mus muscul	FALSE	74338	Mm.271635	Q9D687	chr13	GO:000681	transport //	GO:000588	plasma men	GO:000532	neurotransm	O
17430775	7.78703	8.16085	0.9541935	-0.0676462	0	main		NR_036572	Rps15a-ps4	ribosomal pr	NR_036572	RefSeq	Mus muscul	FALSE	664903	Mm.490762	Q9JL60	chr4	---	---	---	---	---	---	O
17283487	223.7074	235.2411	0.95097073	-0.0725272	0	main		NM_172806	Btbd7	BTB (POZ)	NM_172806	RefSeq	Mus muscul	FALSE	238386	Mm.440255	Q8CFE5	chr12	GO:000727	multicellular	GO:000563	nucleus // n	GO:000367	molecular_f	O
17353765	141.6387	149.3508	0.94836251	-0.0764895	0	main		NM_008214	Hars	histidyl-tRN	NM_008214	RefSeq	Mus muscul	FALSE	15115	Mm.10528	Q61035	chr18	GO:000641	translation /	GO:000573	cytoplasm /	GO:000016	nucleotide b	O
17280431	37.39505	39.46811	0.94747506	-0.0778401	0	main		NM_009417	Tpo	thyroid pero	NM_009417	RefSeq	Mus muscul	FALSE	22018	Mm.4991	P35419	chr12	GO:000659	thyroid horm	GO:000561	extracellular	GO:000444	iodide perox	O
17424850	194.827	205.8544	0.94643107	-0.0794307	0	main		NM_001038	Rnf38	ring finger p	NM_001038	RefSeq	Mus muscul	FALSE	73469	Mm.262859	Q8BI21	chr4	GO:001656	protein ubiq	GO:000563	nucleus // n	GO:000484	ubiquitin-pro	O
17313739	160.6974	169.9508	0.94555248	-0.0807706	0	main		NM_146061	Prr5	proline rich	NM_146061	RefSeq	Mus muscul	FALSE	109270	Mm.291372	Q812A5	chr15	GO:000193	positive regu	GO:003193	TORC2 com	GO:000509	GTPase act	O
17418268	18.60526	19.6862	0.94509149	-0.0814741	0	main		NM_011141	Pou3f1	POU domain	NM_011141	RefSeq	Mus muscul	FALSE	18991	Mm.297371	P21952	chr4	GO:000635	transcription	GO:000563	nucleus // tr	GO:000110	RNA polyme	O
17213321	1763.723	1870.064	0.9431351	-0.0844636	0	main		NM_007561	Bmpr2	bone morph	NM_007561	RefSeq	Mus muscul	FALSE	12168	Mm.7106	O35607	chr1	GO:000170	mesoderm f	GO:000561	extracellular	GO:000016	nucleotide b	O
17502754	269.5221	286.0241	0.94230556	-0.0857331	0	main		NM_001081	Otud4	OTU domain	NM_001081	RefSeq	Mus muscul	FALSE	73945	Mm.34348	B2RRE7	chr8	GO:000650	proteolysis	GO:000557	cellular_com	GO:000484	ubiquitin-sp	O
17309001	26.61485	28.25089	0.9420889	-0.0860649	0	main		NM_178685	Pcdh20	protocadher	NM_178685	RefSeq	Mus muscul	FALSE	219257	Mm.128861	Q8BIZ0	chr14	GO:000715	cell adhesio	GO:000588	plasma men	GO:000550	calcium ion	O
17489707	224.509	238.7547	0.94033332	-0.0887559	0	main		NM_009884	Cebpg	CCAAT/enh	NM_009884	RefSeq	Mus muscul	FALSE	12611	Mm.273090	P53568	chr7	GO:000633	nucleosome	GO:000563	nucleus // n	GO:000097	RNA polyme	O
17303757	49.19018	52.31484	0.94027201	-0.0888499	0	main		NM_134081	Dnajc9	DnaJ (Hsp4	NM_134081	RefSeq	Mus muscul	FALSE	108671	Mm.2871	Q91WN1	chr14	GO:003278	positive regu	GO:000561	extracellular	GO:003107	heat shock	O
17527883	108.4842	116.4939	0.93124361	-0.1027695	0	main		NM_001146	Lrrc49	leucine rich	NM_001146	RefSeq	Mus muscul	FALSE	102747	Mm.269413	Q91YK0	chr9	GO:000815	biological_p	GO:000573	cytoplasm /	GO:000367	molecular_f	O
17535607	299.2179	323.8177	0.92403195	-0.1139854	0	main		NM_001142	Slc6a8	solute carrie	NM_001142	RefSeq	Mus muscul	FALSE	102857	Mm.274553	Q8VBW1	chrX	GO:000681	transport //	GO:000588	integral com	GO:000530	creatine tran	O
17497153	115.8392	125.3863	0.92385851	-0.1142562	0	main		NM_001170	Ctbp2	C-terminal b	NM_001170	RefSeq	Mus muscul	FALSE	13017	Mm.246240	P56546	chr7	GO:000635	transcription	GO:000563	nucleus // n	GO:000368	chromatin b	O
17322327	221.4854	239.9629	0.92299851	-0.1155998	0	main		NM_001076	Cbx5	chromobox	NM_001076	RefSeq	Mus muscul	FALSE	12419	Mm.262059	Q61686	chr15	GO:004589	negative reg	GO:000011	histone deac	GO:000368	chromatin b	O
17348202	132.8309	143.9555	0.92272195	-0.1160321	0	main		NM_011546	Zeb1	zinc finger E	NM_011546	RefSeq	Mus muscul	FALSE	21417	Mm.3929	Q64318	chr18	GO:000012	negative reg	GO:000563	nucleus // n	GO:000122	transcription	O
17521405	68.67085	74.45363	0.92233045	-0.1166444	0	main		NM_001243	Rassf1	Ras associa	NM_001243	RefSeq	Mus muscul	FALSE	56289	Mm.12091	Q99MK9	chr9	GO:000697	cellular resp	GO:000563	nucleus // c	GO:000551	protein bind	O
17323360	88.59486	96.2431	0.92053207	-0.1194601	0	main		NM_176833	Ppm1f	protein phos	NM_176833	RefSeq	Mus muscul	FALSE	68606	Mm.230296	Q8CGA0	chr16	GO:000646	negative reg	GO:000562	intracellular	GO:000382	catalytic act	O
17287613	307.956	334.6676	0.92018468	-0.1200046	0	main		NM_008739	Nsd1	nuclear rece	NM_008739	RefSeq	Mus muscul	FALSE	18193	Mm.168965	O88491	chr13	GO:000012	negative reg	GO:000563	nucleus // n	GO:000097	RNA polyme	O
17320693	39.87461	43.58567	0.91485596	-0.1283835	0	main		NM_001033	Gxylt1	glucoside xy	NM_001033	RefSeq	Mus muscul	FALSE	223827	Mm.22819	Q3UHH8	chr15	GO:001626	O-glycan pr	GO:001602	membrane	GO:001674	transferase	O
17405397	42.52287	46.54868	0.91351398	-0.1305013	0	main		NM_019410	Pfn2	profilin 2	NM_019410	RefSeq	Mus muscul	FALSE	18645	Mm.271744	Q9JJV2	chr3	GO:001063	negative reg	GO:000573	cytoplasm /	GO:000077	adenyl-nucl	O
17512846	156.2486	171.0928	0.9132389	-0.1309358	0	main		NM_001080	Atxn1l	ataxin 1-like	NM_001080	RefSeq	Mus muscul	FALSE	52335	Mm.276770	P0C7T6	chr8	GO:000012	negative reg	GO:000563	nucleus // n	GO:000367	DNA binding	O
17548736	27.64126	30.27794	0.91291746	-0.1314437	0	main		XM_006510	Ntm	neurotrimin	XM_006510	RefSeq	PREDICTED	FALSE	235106	Mm.283138	Q99PJ0	chr9	GO:000715	cell adhesio	GO:000561	extracellular	---	---	O
17438246	330.4792	363.4018	0.90940441	-0.1370061	0	main		NM_001083	Pdgfra	platelet deri	NM_001083	RefSeq	Mus muscul	FALSE	18595	Mm.221403	P26618	chr5	GO:000155	luteinization	GO:000563	nucleus // n	GO:000016	nucleotide b	O
17270134	223.3068	245.9175	0.90805575	-0.1391472	0	main		NM_001044	Ubtf	upstream bi	NM_001044	RefSeq	Mus muscul	FALSE	21429	Mm.2845	P25976	chr11	GO:000018	chromatin si	GO:000165	fibrillar cente	GO:000367	DNA binding	O
17339630	426.7055	471.4305	0.90512918	-0.1438044	0	main		NM_007566	Birc6	baculoviral	NM_007566	RefSeq	Mus muscul	FALSE	12211	Mm.442564	O88738	chr17	GO:000189	placenta dev	GO:000092	spindle pole	GO:000484	ubiquitin-pro	O
17379582	152.9911	169.101	0.90473208	-0.1444375	0	main		NM_146129	Pcif1	PDX1 C-term	NM_146129	RefSeq	Mus muscul	FALSE	228866	Mm.241134	P59114	chr2	GO:001092	negative reg	GO:000563	nucleus // n	GO:000367	molecular_f	O
17496973	275.0791	304.6029	0.90307446	-0.1470831	0	main		NM_001029	Ate1	arginyltransf	NM_001029	RefSeq	Mus muscul	FALSE	11907	Mm.216321	Q9Z2A5	chr7	GO:001659	protein argir	GO:000563	nucleus // n	GO:000405	arginyltrans	O
17322642	101.4861	112.5266	0.90188542	-0.1489839	0	main		NM_026666	Ubn1	ubinuclein 1	NM_026666	RefSeq	Mus muscul	FALSE	170644	Mm.66356	Q4G0F8	chr16	GO:000633	DNA replica	GO:000563	nucleus // b	GO:000367	DNA binding	O
17469097	121.5071	135.3484	0.89773577	-0.1556372	0	main		NM_030081	Rbsn	rabenosyn,	NM_030081	RefSeq	Mus muscul	FALSE	78287	Mm.290734	Q80Y56	chr6	GO:000001	vacuole inhe	GO:000576	endosome /	GO:001713	Rab GTPas	O
17414913	7.986125	8.924139	0.89489025	-0.1602173	0	main		NM_001163	Frmd3	FERM doma	NM_001163	RefSeq	Mus muscul	FALSE	242506	Mm.221597	Q8BHD4	chr4	GO:000815	biological_p	GO:000573	cytoplasm /	GO:000809	cytoskeletal	O
17524087	158.9219	177.9557	0.89304192	-0.1632002	0	main		NM_019683	Ankrd49	ankyrin repe	NM_019683	RefSeq	Mus muscul	FALSE	56503	Mm.395005	Q8VE42	chr9	GO:000728	spermatoge	GO:000563	nucleus // c	GO:000367	molecular_f	O
17321365	238.9829	267.7094	0.89269521	-0.1637604	0	main		NM_001033	Kmt2d	lysine (K)-sp	NM_001033	RefSeq	Mus muscul	FALSE	381022	Mm.264889	Q6PDK2	chr15	GO:000155	oocyte grow	GO:000563	nucleus // h	GO:000551	protein bind	O
17451526	134.5998	151.2395	0.88997782	-0.1681587	0	main		NM_001159	Kctd10	potassium c	NM_001159	RefSeq	Mus muscul	FALSE	330171	Mm.490304	Q922M3	chr5	GO:005126	protein hom	GO:000563	nucleus // n	GO:000551	protein bind	O
17543290	76.80655	86.55167	0.88740691	-0.1723323	0	main		NM_001033	Arhgef9	CDC42 guai	NM_001033	RefSeq	Mus muscul	FALSE	236915	Mm.44841	Q3UTH8	chrX	GO:000726	small GTPa	GO:000573	cytoplasm /	GO:000508	guanyl-nucl	O
17260150	89.54873	101.2187	0.8847054	-0.176731	0	main		NM_001080	Znrf3	zinc and ring	NM_001080	RefSeq	Mus muscul	FALSE	407821	Mm.216313	Q5SSZ7	chr11	GO:000651	ubiquitin-de	GO:000588	plasma men	GO:000484	ubiquitin-pro	O
17403681	132.8888	150.9372	0.88042444	-0.1837289	0	main		NM_001080	Zzz3	zinc finger,	NM_001080	RefSeq	Mus muscul	FALSE	108946	Mm.379292	Q6KAQ7	chr3	GO:000635	transcription	GO:000563	nucleus // A	GO:000367	DNA binding	O
17276732	164.5118	187.7219	0.87635912	-0.1904059	0	main		NM_026114	Eif2s1	eukaryotic tr	NM_026114	RefSeq	Mus muscul	FALSE	13665	Mm.196220	Q6ZWX6	chr12	GO:000223	positive regu	GO:000563	nucleus // n	GO:000367	nucleic acid	O
17496253	448.0515	511.3192	0.87626575	-0.1905596	0	main		NM_183020	Atxn2l	ataxin 2-like	NM_183020	RefSeq	Mus muscul	FALSE	233871	Mm.231450	Q7TQH0	chr7	GO:001060	regulation of	GO:000573	cytoplasm /	GO:004482	poly(A) RNA	O
17441134	10.695	12.20723	0.87612014	-0.1907994	0	main		XM_006530	Msi1	musashi RN	XM_006530	RefSeq	PREDICTED	FALSE	17690	Mm.5077	Q61474	chr5	GO:000972	response to	GO:000563	nucleus // c	GO:000016	nucleotide b	O
17405285	138.5756	158.7334	0.87300845	-0.1959325	0	main		NM_030595	Nbea	neurobeach	NM_030595	RefSeq	Mus muscul	FALSE	26422	Mm.384353	Q9EPN1	chr3	GO:000660	protein targe	GO:000580	trans-Golgi	GO:000554	phospholipi	O
17223985	44.56887	51.07743	0.87257464	-0.1966496	0	main		NM_011770	Ikzf2	IKAROS fan	NM_011770	RefSeq	Mus muscul	FALSE	22779	Mm.106343	P81183	chr1	GO:000635	transcription	GO:000563	nucleus // n	GO:000367	nucleic acid	O
17397439	189.1699	216.8257	0.87245147	-0.1968532	0	main		NM_053089	Naa15	N(alpha)-ac	NM_053089	RefSeq	Mus muscul	FALSE	74838	Mm.275281	Q80UM3	chr3	GO:000152	angiogenesi	GO:000563	nucleus // tr	GO:000459	peptide alph	O
17377126	69.82295	80.23766	0.87020172	-0.2005782	0	main		NM_025314	Dtd1	D-tyrosyl-tR	NM_025314	RefSeq	Mus muscul	FALSE	66044	Mm.28109	Q9DD18	chr2	GO:000626	DNA replica	GO:000563	nucleus // c	GO:000216	aminoacyl-tl	O
17268493	240.0384	277.6746	0.86445933	-0.21013	0	main		NM_008942	Npepps	aminopeptid	NM_008942	RefSeq	Mus muscul	FALSE	19155	Mm.29824	Q11011	chr11	GO:000650	proteolysis	GO:000563	nucleus // c	GO:000417	aminopeptic	O
17440257	113.0726	130.8998	0.86381033	-0.2112135	0	main		NM_172716	Pcgf3	polycomb gr	NM_172716	RefSeq	Mus muscul	FALSE	69587	Mm.486543	Q8BTQ0	chr5	GO:000635	transcription	GO:000563	nucleus // n	GO:000827	zinc ion binc	O
17465332	10.57571	12.25005	0.86331974	-0.2120331	0	main		NM_138682	Lrrc4	leucine rich	NM_138682	RefSeq	Mus muscul	FALSE	192198	Mm.443660	Q99PH1	chr6	GO:005080	regulation of	GO:000588	plasma men	GO:000551	protein bind	O
17416897	153.7336	179.1626	0.85806748	-0.220837	0	main		NM_007983	Faf1	Fas-associa	NM_007983	RefSeq	Mus muscul	FALSE	14084	Mm.318259	P54731	chr4	GO:000691	apoptotic pr	GO:000563	nucleus // n	GO:000551	protein bind	O
17457892	106.5039	124.1933	0.85756559	-0.2216811	0	main		NM_007610	Casp2	caspase 2	NM_007610	RefSeq	Mus muscul	FALSE	12366	Mm.3921	P29594	chr6	GO:000650	proteolysis	GO:000563	nucleus // c	GO:000419	cysteine-typ	O
17389861	147.0268	173.1994	0.84888747	-0.2363548	0	main		NM_026574	Ino80	INO80 hom	NM_026574	RefSeq	Mus muscul	FALSE	68142	Mm.330496	Q6ZPV2	chr2	GO:000007	mitotic siste	GO:000563	nucleus // m	GO:000016	nucleotide b	O
17545121	17.9251	21.15036	0.84750803	-0.2387011	0	main		NM_001110	Dcx	doublecortin	NM_001110	RefSeq	Mus muscul	FALSE	13193	Mm.12871	O88809	chrX	GO:000176	neuron migr	GO:000573	cytoplasm /	GO:000468	calmodulin-	O
17349590	240.1774	283.639	0.84677142	-0.2399555	0	main		NM_019912	Ube2d2a	ubiquitin-cor	NM_019912	RefSeq	Mus muscul	FALSE	56550	Mm.180052	P62838	chr18	GO:000020	protein poly	GO:000015	ubiquitin liga	GO:000016	nucleotide b	O
17494868	18.42623	21.79678	0.84536477	-0.2423541	0	main		NM_001170	Trim66	tripartite mo	NM_001170	RefSeq	Mus muscul	FALSE	330627	Mm.183528	Q924W6	chr7	GO:004589	negative reg	GO:000562	intracellular	GO:000368	chromatin b	O
17217127	234.5643	277.89	0.84409047	-0.2445305	0	main		NM_001145	Nucks1	nuclear case	NM_001145	RefSeq	Mus muscul	FALSE	98415	Mm.246869	Q80XU3	chr1	GO:004259	glucose hon	GO:000563	nucleus // n	GO:000107	transcription	O
17472777	202.8967	240.4651	0.84376777	-0.2450821	0	main		NM_010586	Itpr2	inositol 1,4,5	NM_010586	RefSeq	Mus muscul	FALSE	16439	Mm.7800	Q9Z329	chr6	GO:000166	response to	GO:000573	cytoplasm /	GO:000521	ion channel	O
17246577	57.69034	68.46188	0.84266368	-0.2469711	0	main		NM_178149	Pik3ip1	phosphoinos	NM_178149	RefSeq	Mus muscul	FALSE	216505	Mm.390323	Q7TMJ8	chr11	GO:001406	negative reg	GO:000588	plasma men	GO:003631	phosphatidy	O
17417437	211.4744	251.1756	0.84193847	-0.2482133	0	main		NM_029868	Gpbp1l1	GC-rich pror	NM_029868	RefSeq	Mus muscul	FALSE	77110	Mm.41715	Q6NZP2	chr4	GO:000635	transcription	GO:000563	nucleus // n	GO:000367	DNA binding	O
17470235	110.1043	131.8461	0.83509713	-0.2599841	0	main		NM_001159	Cacna1c	calcium cha	NM_001159	RefSeq	Mus muscul	FALSE	12288	Mm.436656	Q01815	chr6	GO:000681	transport //	GO:000209	caveolar ma	GO:000521	ion channel	O
17467823	521.7062	625.5967	0.83393375	-0.2619953	0	main		NM_019715	Kcmf1	potassium c	NM_019715	RefSeq	Mus muscul	FALSE	74287	Mm.29194	Q80UY2	chr6	GO:001656	protein ubiq	---	---	GO:000827	zinc ion binc	O
17448467	53.34187	64.08509	0.83236007	-0.2647203	0	main		NM_001164	Bend4	BEN domain	NM_001164	RefSeq	Mus muscul	FALSE	666938	Mm.101339	P86174	chr5	GO:000815	biological_p	GO:000557	cellular_com	GO:000367	molecular_f	O
17287081	49.43338	59.50406	0.83075642	-0.2675026	0	main		NM_031166	Id4	inhibitor of D	NM_031166	RefSeq	Mus muscul	FALSE	15904	Mm.458006	P41139	chr13	GO:000008	G1/S transit	GO:000563	nucleus // c	GO:000108	RNA polyme	O
17223666	352.6698	424.8667	0.83007164	-0.2686922	0	main		NM_001081	Ino80d	INO80 comp	NM_001081	RefSeq	Mus muscul	FALSE	227195	Mm.74718	Q66JY2	chr1	GO:000628	DNA repair	GO:000563	nucleus // c	GO:000367	molecular_f	O
17526382	265.1712	321.4497	0.82492284	-0.2776689	0	main		NM_001081	Kmt2a	lysine (K)-sp	NM_001081	RefSeq	Mus muscul	FALSE	214162	Mm.2389	P55200	chr9	GO:000630	DNA methyl	GO:000563	nucleus // n	GO:000104	core promot	O
17373120	191.0578	232.1719	0.82291526	-0.2811842	0	main		NM_001244	Celf1	CUGBP, Ela	NM_001244	RefSeq	Mus muscul	FALSE	13046	Mm.29495	P28659	chr2	GO:000637	mRNA splic	GO:000563	nucleus // n	GO:000016	nucleotide b	O
17324774	210.8045	256.5869	0.82157156	-0.2835419	0	main		NM_177633	Ubxn7	UBX domain	NM_177633	RefSeq	Mus muscul	FALSE	224111	Mm.31275	Q6P5G6	chr16	GO:000815	biological_p	GO:000563	nucleus // n	GO:000813	transcription	O
17523597	189.8677	231.1964	0.82123986	-0.2841244	0	main		NM_013860	Limd1	LIM domains	NM_013860	RefSeq	Mus muscul	FALSE	29806	Mm.10987	Q9QXD8	chr9	GO:000166	response to	GO:000093	cytoplasmic	GO:000371	transcription	O
17360068	256.9593	313.8358	0.81876988	-0.2884701	0	main		NM_001177	Wbp1l	WW domain	NM_001177	RefSeq	Mus muscul	FALSE	226178	Mm.329895	Q8BGW2	chr19	GO:000815	biological_p	GO:001602	membrane	GO:000367	molecular_f	O
17231188	83.30973	101.7966	0.81839403	-0.2891325	0	main		NM_145415	Diexf	digestive org	NM_145415	RefSeq	Mus muscul	FALSE	215193	Mm.173758	Q8BTT6	chr1	GO:000046	maturation c	GO:000563	nucleus // n	GO:001984	rRNA bindin	O
17289372	318.5246	389.7773	0.81719638	-0.2912453	0	main		NM_007930	Enc1	ectodermal-	NM_007930	RefSeq	Mus muscul	FALSE	13803	Mm.241073	O35709	chr13	GO:000727	multicellular	GO:000079	nuclear chro	GO:000377	actin binding	O
17248929	99.2331	121.44	0.81713686	-0.2913504	0	main		NM_001024	Zfp62	zinc finger p	NM_001024	RefSeq	Mus muscul	FALSE	22720	Mm.16650	Q8C827	chr11	GO:000635	transcription	GO:000563	nucleus	GO:000367	nucleic acid	O
17492861	76.19315	93.37978	0.81594913	-0.2934489	0	main		NM_013886	Hdgfrp3	hepatoma-d	NM_013886	RefSeq	Mus muscul	FALSE	29877	Mm.392934	Q9JMG7	chr7	GO:000828	cell prolifera	GO:000563	nucleus // n	GO:000368	chromatin b	O
17381475	225.4937	276.614	0.81519265	-0.294787	0	main		NM_001110	Celf2	CUGBP, Ela	NM_001110	RefSeq	Mus muscul	FALSE	14007	Mm.398543	Q9Z0H4	chr2	GO:000637	mRNA splic	GO:000563	nucleus // n	GO:000016	nucleotide b	O
17309161	218.3356	270.8407	0.80614029	-0.3108972	0	main		NM_015822	Fbxl3	F-box and le	NM_015822	RefSeq	Mus muscul	FALSE	50789	Mm.214746	Q8C4V4	chr14	GO:001656	protein ubiq	GO:000563	nucleus // n	GO:000484	ubiquitin-pro	O
17236754	210.1691	261.0039	0.80523356	-0.3125208	0	main		NM_001001	Eea1	early endos	NM_001001	RefSeq	Mus muscul	FALSE	216238	Mm.490373	Q8BL66	chr10	GO:000689	endocytosis	GO:000573	cytoplasm /	GO:000551	protein bind	O
17454157	67.59332	83.99852	0.80469656	-0.3134832	0	main		NM_144913	Mepce	methylphosp	NM_144913	RefSeq	Mus muscul	FALSE	231803	Mm.61193	Q8K3A9	chr5	GO:000012	negative reg	GO:000557	cellular_com	GO:000017	rRNA (aden	O
17371148	10.41962	12.96029	0.80396503	-0.3147953	0	main		NM_009322	Tbr1	T-box brain	NM_009322	RefSeq	Mus muscul	FALSE	21375	Mm.308525	Q64336	chr2	GO:000166	conditioned	GO:000563	nucleus // n	GO:000097	RNA polyme	O
17315718	99.21346	123.6243	0.80254012	-0.3173546	0	main		NM_001136	Ptger4	prostagland	NM_001136	RefSeq	Mus muscul	FALSE	19219	Mm.18509	P32240	chr15	GO:000193	positive regu	GO:000588	plasma men	GO:000487	signal trans	O
17436237	272.6559	344.4765	0.79150798	-0.3373242	0	main		NM_008037	Fosl2	fos-like antig	NM_008037	RefSeq	Mus muscul	FALSE	14284	Mm.24684	P47930	chr5	GO:000166	response to	GO:000563	nucleus // n	GO:000097	RNA polyme	O
17267304	128.722	162.8956	0.79021164	-0.339689	0	main		NM_001114	Rps6kb1	ribosomal pr	NM_001114	RefSeq	Mus muscul	FALSE	72508	Mm.394280	Q8BSK8	chr11	GO:000008	G1/S transit	GO:000563	nucleus // n	GO:000016	nucleotide b	O
17523736	62.54014	79.36652	0.78799146	-0.3437481	0	main		NM_029851	Dync2h1	dynein cytop	NM_029851	RefSeq	Mus muscul	FALSE	110350	Mm.237596	Q45VK7	chr9	GO:000701	microtubule-	GO:000573	cytoplasm /	GO:000016	nucleotide b	O
17213336	230.5548	292.7206	0.78762752	-0.3444146	0	main		NM_007561	Bmpr2	bone morph	NM_007561	RefSeq	Mus muscul	FALSE	12168	Mm.7106	O35607	chr1	GO:000170	mesoderm f	GO:000561	extracellular	GO:000016	nucleotide b	O
17323532	33.64933	42.75389	0.78704721	-0.3454779	0	main		NM_001159	P2rx6	purinergic re	NM_001159	RefSeq	Mus muscul	FALSE	18440	Mm.8032	O54803	chr16	GO:000681	transport //	GO:000563	integral com	GO:000161	purinergic n	O
17346936	51.22615	65.23941	0.78520253	-0.3488633	0	main		NM_001164	Tgif1	TGFB-induc	NM_001164	RefSeq	Mus muscul	FALSE	21815	Mm.101034	P70284	chr17	GO:000012	negative reg	GO:000563	nucleus // n	GO:000097	RNA polyme	O
17228906	95.89493	122.7104	0.78147353	-0.3557311	0	main		NM_001038	Dnm3	dynamin 3	NM_001038	RefSeq	Mus muscul	FALSE	103967	Mm.441620	Q8BZ98	chr1	GO:000689	endocytosis	GO:000191	photorecept	GO:000016	nucleotide b	O
17431910	31.9076	41.01265	0.77799411	-0.3621689	0	main		NM_011039	Pax7	paired box 7	NM_011039	RefSeq	Mus muscul	FALSE	18509	Mm.218760	P47239	chr4	GO:000633	chromatin re	GO:000563	nucleus // tr	GO:000098	transcription	O
17357502	146.1631	188.9486	0.77356011	-0.3704147	0	main		NM_001164	Cpsf7	cleavage an	NM_001164	RefSeq	Mus muscul	FALSE	269061	Mm.490260	Q8BTV2	chr19	GO:000639	mRNA proc	GO:000563	nucleus // m	GO:000016	nucleotide b	O
17533416	328.552	427.4375	0.76865507	-0.3795918	0	main		NM_010028	Ddx3x	DEAD/H (As	NM_010028	RefSeq	Mus muscul	FALSE	13205	Mm.289662	Q62167	chrX	GO:000237	immune sys	GO:000563	nucleus // c	GO:000016	nucleotide b	O
17363704	66.40451	86.41212	0.76846292	-0.3799524	0	main		NM_001166	Rfx3	regulatory fa	NM_001166	RefSeq	Mus muscul	FALSE	19726	Mm.313321	P48381	chr19	GO:000635	transcription	GO:000079	nuclear chro	GO:000097	RNA polyme	O
17349774	53.43423	69.77926	0.76576092	-0.3850341	0	main		NM_001305	Hars2	histidyl-tRN	NM_001305	RefSeq	Mus muscul	FALSE	70791	Mm.282700	Q99KK9	chr18	GO:000641	translation /	GO:000573	cytoplasm /	GO:000016	nucleotide b	O
17241454	195.0712	256.1082	0.76167495	-0.3927526	0	main		NM_053183	Ddx50	DEAD (Asp-	NM_053183	RefSeq	Mus muscul	FALSE	94213	Mm.114116	Q99MJ9	chr10	GO:001050	RNA second	GO:000563	nucleus // n	GO:000016	nucleotide b	O
17460405	96.07951	126.2703	0.76090347	-0.3942146	0	main		NM_001164	Tia1	cytotoxic gra	NM_001164	RefSeq	Mus muscul	FALSE	21841	Mm.333219	P52912	chr6	GO:000691	apoptotic pr	GO:000563	nucleus // n	GO:000016	nucleotide b	O
17414802	38.02921	50.01162	0.76040748	-0.3951554	0	main		NM_021362	Pappa	pregnancy-a	NM_021362	RefSeq	Mus muscul	FALSE	18491	Mm.317854	Q8R4K8	chr4	GO:000650	proteolysis	GO:000557	extracellular	GO:000417	endopeptida	O
17352493	166.6056	219.5239	0.7589406	-0.3979411	0	main		NM_001276	Epc1	enhancer of	NM_001276	RefSeq	Mus muscul	FALSE	13831	Mm.312133	Q8C9X6	chr18	GO:000012	negative reg	GO:000563	nucleus // n	GO:000440	histone acet	O
17417290	107.5645	143.1911	0.75119543	-0.4127398	0	main		NM_181040	Atpaf1	ATP synthas	NM_181040	RefSeq	Mus muscul	FALSE	230649	Mm.109600	Q811I0	chr4	GO:000646	protein com	GO:000573	mitochondri	GO:000367	molecular_f	O
17224291	79.75684	108.7479	0.73341039	-0.4473074	0	main		NM_001310	Usp37	ubiquitin spe	NM_001310	RefSeq	Mus muscul	FALSE	319651	Mm.66568	Q8C0R0	chr1	GO:000008	G1/S transit	GO:000563	nucleus // n	GO:000419	cysteine-typ	O
17284839	39.28789	53.58744	0.73315482	-0.4478102	0	main		NM_177290	Itgb8	integrin beta	NM_177290	RefSeq	Mus muscul	FALSE	320910	Mm.217000	Q0VBD0	chr12	GO:000157	ganglioside	GO:000998	cell surface	GO:000510	receptor bin	O
17353143	128.448	180.7326	0.71070742	-0.4926723	0	main		NM_144861	Rprd1a	regulation of	NM_144861	RefSeq	Mus muscul	FALSE	225283	Mm.28427	Q8VDS4	chr18	GO:007094	dephosphor	GO:001659	DNA-directe	GO:000367	molecular_f	O
17310004	155.8364	221.2115	0.7044679	-0.5053941	0	main		NM_030168	Rictor	RPTOR inde	NM_030168	RefSeq	Mus muscul	FALSE	78757	Mm.275811	Q6QI06	chr15	GO:000193	regulation of	GO:003193	TORC2 com	GO:000551	protein bind	O
17401296	89.3961	127.7577	0.6997316	-0.5151264	0	main		NM_172684	Rsbn1	rosbin, roun	NM_172684	RefSeq	Mus muscul	FALSE	229675	Mm.29366	Q80T69	chr3	GO:000815	biological_p	GO:000563	nucleus // n	GO:000551	protein bind	O
17523852	122.6337	175.4357	0.69902363	-0.5165869	0	main		NM_001291	Birc2	baculoviral	NM_001291	RefSeq	Mus muscul	FALSE	11797	Mm.335659	Q62210	chr9	GO:000020	protein poly	GO:000174	XY body //	GO:000371	transcription	O
17214711	63.64584	91.23893	0.69757328	-0.5195833	0	main		NM_021342	Kcne4	potassium v	NM_021342	RefSeq	Mus muscul	FALSE	57814	Mm.24386	Q9WTW3	chr1	GO:000681	transport //	GO:000807	voltage-gate	GO:000524	voltage-gate	O
17337919	24.07984	35.74555	0.67364581	-0.5699378	0	main		NM_013688	Tcte1	t-complex-as	NM_013688	RefSeq	Mus muscul	FALSE	21645	Mm.491193	A6H639	chr17	GO:000815	biological_p	GO:000557	cellular_com	GO:000367	molecular_f	O
17233194	35.06404	53.4034	0.65658816	-0.6069394	0	main		NM_011376	Sim1	single-minde	NM_011376	RefSeq	Mus muscul	FALSE	20464	Mm.4774	Q61045	chr10	GO:000635	transcription	GO:000563	nucleus // n	GO:000098	RNA polyme	O
17255565	32.86808	50.12595	0.65570987	-0.6088705	0	main		NM_010461	Hoxb8	homeobox B	NM_010461	RefSeq	Mus muscul	FALSE	15416	Mm.4822	P09632	chr11	GO:000635	transcription	GO:000563	nucleus	GO:000367	DNA binding	O
17482021	1209.146	1920.469	0.62960975	-0.6674702	0	main		NM_011055	Pde3b	phosphodies	NM_011055	RefSeq	Mus muscul	FALSE	18576	Mm.430730	Q61409	chr7	GO:000152	angiogenesi	GO:000578	endoplasmic	GO:000411	3',5'-cyclic-r	O
17523861	57.18039	91.97252	0.62171168	-0.6856824	0	main		NM_001291	Birc2	baculoviral	NM_001291	RefSeq	Mus muscul	FALSE	11797	Mm.335659	Q62210	chr9	GO:000020	protein poly	GO:000174	XY body //	GO:000371	transcription	O
17287066	257.7346	427.7617	0.60251911	-0.7309211	0	main		NM_001170	Rnf144b	ring finger p	NM_001170	RefSeq	Mus muscul	FALSE	218215	Mm.287609	Q8BKD6	chr13	GO:000020	protein poly	GO:000015	ubiquitin liga	GO:000484	ubiquitin-pro	O
17339108	388.2724	648.3241	0.59888627	-0.739646	0	main		NM_008549	Man2a1	mannosidas	NM_008549	RefSeq	Mus muscul	FALSE	17158	Mm.2433	P27046	chr17	GO:000170	in utero emb	GO:000013	Golgi memb	GO:000382	catalytic act	O
17392991	103.0877	178.1121	0.57877988	-0.7889133	0	main		NM_001289	Bcl2l1	BCL2-like 1	NM_001289	RefSeq	Mus muscul	FALSE	12048	Mm.238213	Q64373	chr2	GO:000091	cytokinesis	GO:000562	intracellular	GO:000551	protein bind	O
17233960	253.8845	507.3959	0.50036766	-0.9989395	0	main		NM_001242	Jmjd1c	jumonji dom	NM_001242	RefSeq	Mus muscul	FALSE	108829	Mm.23846	Q69ZK6	chr10	GO:000338	epithelial ce	GO:000563	nucleus	GO:000090	sulfonate di	O
17297100	79.65768	162.9167	0.4889473	-1.0322491	0	main	2Down	NM_001305	Kctd6	potassium c	NM_001305	RefSeq	Mus muscul	FALSE	71393	Mm.292712	Q8BNL5	chr14	GO:005126	protein hom	GO:000557	cellular_com	GO:003050	ankyrin binc	O
17388774	13.06371	31.74073	0.4115756	-1.2807706	0	main	2Down	NM_007914	Ehf	ets homolog	NM_007914	RefSeq	Mus muscul	FALSE	13661	Mm.10724	O70273	chr2	GO:000635	transcription	GO:000563	nucleus // n	GO:000097	RNA polyme	O
17539271	335.2821	1297.645	0.25837737	-1.9524484	0	main	2Down	NM_001079	Adgrg2	adhesion G	NM_001079	RefSeq	Mus muscul	FALSE	237175	Mm.213016	Q8CJ12	chrX	GO:000716	signal trans	GO:000588	plasma men	GO:000487	signal trans	O

Supplementary Table 6. Predicted target genes of miR-342-3p by TargetScan, miRDB, Pictar and DIANA-microT v5.0.

Acvr1b	*Ankrd17*	*Atxn2l*	*Brca1*	*Cdyl2*	*Csk*	*Dmbx1*	*E2f3*	*Ep300*	*Fam78b*
Adamts10	*Ankrd49*	*Atxn7*	*Bsn*	*Cebpg*	*Ctbp2*	*Dnajc27*	*Eda*	*Epc1*	*Fat2*
Adamts16	*Ankrd52*	*Bcl2l1*	*Btbd7*	*Celf1*	*Cux2*	*Dnajc6*	*Edc3*	*Etaa1*	*Fbxl3*
Adgrg2	*Arf3*	*Bend4*	*Cacna1c*	*Celf2*	*Dach1*	*Dnajc9*	*Ednra*	*Ext1*	*Fbxo33*
Aff1	*Arhgef9*	*Birc2*	*Camkk2*	*Clec7a*	*Dcx*	*Dnm1*	*Eea1*	*F11r*	*Fgf10*
Afp	*Armcx3*	*Birc2*	*Casp2*	*Col1a2*	*Ddx3x*	*Dnm3*	*Eef1a2*	*Faf1*	*Fign*
Agfg1	*Atat1*	*Birc6*	*Cbx5*	*Col2a1*	*Ddx50*	*Dtd1*	*Ehf*	*Fam126b*	*Flnc*
Agpat4	*Ate1*	*Bmp2k*	*Ccdc171*	*Colgalt2*	*Dgcr2*	*Dync2h1*	*Eif2s1*	*Fam192a*	*Fosl2*
Akirin1	*Atpaf1*	*Bmpr2*	*Cd180*	*Cpsf7*	*Diexf*	*Dyrk2*	*Elavl2*	*Fam222a*	*Frmd3*
Ank2	*Atxn1l*	*Bmpr2*	*Cdc27*	*Csf1*	*Dlgap4*	*E2f1*	*Enc1*	*Fam53c*	*Frmd4a*
Fut8	*Grin2d*	*Hoxa1*	*Itgam*	*Kdm6b*	*Lin28b*	*Matn1*	*Naa15*	*Npepps*	*Pappa*
Fut9	*Grm8*	*Hoxb8*	*Itgb8*	*Klf13*	*Lmx1a*	*Mbnl3*	*Napb*	*Nprl3*	*Pax5*
Fzd5	*Gspt1*	*Hoxd9*	*Itpr2*	*Klf6*	*Lrp8*	*Mepce*	*Nbea*	*Nsd1*	*Pax5*
Gm328	*Gsta4*	*Hrh1*	*Jade1*	*Klf7*	*Lrrc4*	*Mink1*	*Ncoa2*	*Ntm*	*Pax7*
Gmeb1	*Gxylt1*	*Id4*	*Jmjd1c*	*Kmt2a*	*Lrrc49*	*Mir592*	*Ndel1*	*Ntm*	*Pcdh20*
Golm1	*H2-Ke6*	*Iffo2*	*Kcmf1*	*Kmt2d*	*Lsamp*	*Mmab*	*Ndufa11*	*Nucks1*	*Pcgf3*
Gpbp1l1	*Hars*	*Ikzf2*	*Kcne4*	*Kpnb1*	*Ltbp2*	*Mrfap1*	*Neo1*	*Olfml2a*	*Pcif1*
Gpd1l	*Hars2*	*Inhba*	*Kctd10*	*Kpnb1*	*Magix*	*Msantd3*	*Nhlh2*	*Oser1*	*Pde3b*
Grin1	*Hdgfrp3*	*Ino80*	*Kctd5*	*Lasp1*	*Man2a1*	*Msi1*	*Nkx2-2*	*Otud4*	*Pdgfra*
Grin2b	*Hip1*	*Ino80d*	*Kctd6*	*Limd1*	*Manf*	*Msi1*	*Nlk*	*P2rx6*	*Pfkfb2*
Pfn2	*Prickle2*	*Rassf4*	*Rnf38*	*Scrn1*	*Slc41a2*	*Sox30*	*Tbr1*	*Tmem35*	*Upf1*
Pik3ip1	*Prkaa2*	*Rbpms2*	*Rprd1a*	*Sec31b*	*Slc6a19*	*Sox6*	*Tbx18*	*Tpo*	*Usp13*
Pkp1	*Prr5*	*Rbsn*	*Rps15a-ps4*	*Sema4g*	*Slc6a4*	*Spop*	*Tcf12*	*Trim66*	*Usp37*
Plekhb2	*Ptger4*	*Rfx3*	*Rps6kb1*	*Sept11*	*Slc6a8*	*Stat5b*	*Tcte1*	*Trmt2a*	*Vkorc1l1*
Plk2	*Ptpn14*	*Rgp1*	*Rreb1*	*Setd7*	*Slitrk4*	*Strn*	*Tdrkh*	*Tspan4*	*Vps33b*
Plxdc2	*Ptprc*	*Rgs4*	*Rsbn1*	*Setd8*	*Smtnl2*	*Suv420h1*	*Tenm3*	*Txk*	*Vsig8*
Polh	*Ptprn*	*Rhbdf1*	*S100a7a*	*Sgms2*	*Snap25*	*Sv2a*	*Tgif1*	*Ube2d2a*	*Wasl*
Pou3f1	*Pygb*	*Rictor*	*Samd4*	*Sh2b1*	*Snx4*	*Tab2*	*Thap1*	*Ubn1*	*Wbp1l*
Ppm1f	*Rad23b*	*Rmnd5a*	*Sarm1*	*Sh2b1*	*Sorcs3*	*Tacr3*	*Tia1*	*Ubtf*	*Xkrx*
Ppp3r1	*Rassf1*	*Rnf144b*	*Scai*	*Sim1*	*Sorl1*	*Tanc2*	*Tiam1*	*Ubxn7*	*Zak*
Zeb1	*Zeb2*	*Zfhx4*	*Zfp62*	*Zfp641*	*Zic4*	*Znrf3*	*Zzz3*		

Supplementary Table 7. Candidate genes of miR-342-3p sorted by the ratio of HFHS *Mir342* (-/-) / HFHS *Mir342* (+/+) expression values in epididymal adipose tissues.

	Expression values				Ratio			
Gene Symbol	STD (-/-)	STD (+/+)	HFHS (-/-)	HFHS (+/+)	STD (-/-) / STD (+/+)	HFHS (-/-) / HFHS (+/+)	HFHS (+/+) / STD (+/+)	HFHS (-/-) / STD (-/-)
Snap25	30.157	34.5344	115.75	38.636	0.8732497	2.9960211	1.11877591	3.83839372
Fat2	31.146	21.3482	37.451	23.364	1.4589654	1.602928	1.09443939	1.20243259
Msi1	14.485	12.2072	16.167	10.695	1.1866197	1.5116765	0.87612014	1.11612016
Eef1a2	49.677	34.3954	53.33	35.99	1.4442798	1.4817992	1.04636819	1.07355066
S100a7a	55.009	38.9299	55.019	37.352	1.4130339	1.4729723	0.95947509	1.00017433
Grin2d	28.194	22.8233	35.715	25.463	1.2353373	1.4026364	1.11566348	1.26675534
Tcte1	32.741	35.7456	33.335	24.08	0.9159509	1.3843618	0.67364581	1.01814352
Gsta4	167.26	123.364	200.47	148.77	1.3558178	1.347532	1.20590579	1.19853616
Tpo	56.762	39.4681	50.311	37.395	1.4381621	1.3453906	0.94747506	0.88635633
Hoxd9	22.193	21.0107	31.725	23.665	1.0562794	1.3406079	1.12631843	1.42949996
Ntm	32.716	25.1582	35.914	26.965	1.3003986	1.3318864	1.07181564	1.0977685
Pkp1	27.623	19.211	28.006	21.279	1.4378847	1.3161286	1.10765268	1.0138597
Pax7	50.596	41.0127	41.613	31.908	1.2336728	1.3041705	0.77799411	0.82245224
Lmx1a	22.078	16.3053	22.304	17.126	1.3540186	1.3023826	1.05030818	1.01025425

HFHS, high fat-high sucrose chow; STD, standard chow.
